# Quantitative proteomic analysis for high-throughput screening of differential glycoproteins in hepatocellular carcinoma serum

**DOI:** 10.7497/j.issn.2095-3941.2015.0010

**Published:** 2015-09

**Authors:** Hua-Jun Gao, Ya-Jing Chen, Duo Zuo, Ming-Ming Xiao, Ying Li, Hua Guo, Ning Zhang, Rui-Bing Chen

**Affiliations:** ^1^Research Center of Basic Medical Sciences & School of Medical Laboratory, Tianjin Medical University, Tianjin 300070, China; ^2^Laboratory of Cancer Cell Biology, Tianjin Medical University Cancer Institute and Hospital, National Clinical Research Center for Cancer, Tianjin Key Laboratory of Cancer Prevention and Therapy, Tianjin 300060, China

**Keywords:** Glycoprotein, hepatocellular carcinoma (HCC), mass spectrometry, proteomics, serum

## Abstract

**Objective:**

Hepatocellular carcinoma (HCC) is a leading cause of cancer-related deaths. Novel serum biomarkers are required to increase the sensitivity and specificity of serum screening for early HCC diagnosis. This study employed a quantitative proteomic strategy to analyze the differential expression of serum glycoproteins between HCC and normal control serum samples.

**Methods:**

Lectin affinity chromatography (LAC) was used to enrich glycoproteins from the serum samples. Quantitative mass spectrometric analysis combined with stable isotope dimethyl labeling and 2D liquid chromatography (LC) separations were performed to examine the differential levels of the detected proteins between HCC and control serum samples. Western blot was used to analyze the differential expression levels of the three serum proteins.

**Results:**

A total of 2,280 protein groups were identified in the serum samples from HCC patients by using the 2D LC-MS/MS method. Up to 36 proteins were up-regulated in the HCC serum, whereas 19 proteins were down-regulated. Three differential glycoproteins, namely, fibrinogen gamma chain (FGG), FOS-like antigen 2 (FOSL2), and α-1,6-mannosylglycoprotein 6-β-N-acetylglucosaminyltransferase B (MGAT5B) were validated by Western blot. All these three proteins were up-regulated in the HCC serum samples.

**Conclusion:**

A quantitative glycoproteomic method was established and proven useful to determine potential novel biomarkers for HCC.

## Introduction

Hepatocellular carcinoma (HCC) ranks the second leading cause of cancer-related deaths worldwide^1^. HCC is usually associated with viral infections, such as hepatitis C and hepatitis B. HCC diagnosis is generally based on ultrasound scanning and serum alpha-fetoprotein (AFP) estimation every 6 months^2^. However, ultrasound can only detect tumors larger than 3 cm, and this method cannot distinguish malignant tumors from benign ones. Other methods such as computed tomography and nuclear magnetic resonance are usually very expensive for routine screening^3^. Serum is an ideal sample source for HCC detection. The known serum biomarkers for HCC include AFP, des-γ-carboxyprothrombin (DCP), fucosylated AFP (AFP-L3), Golgi protein 73 (GP73), isozyme of alkaline phosphatase (variant ALP), and isozyme of gamma-glutamyl transpeptidase (novel γ-GTP)^4^^,^^5^. AFP is a classical biomarker, and an increase in AFP serum level correlates with tumor size^6^. However, in patients with chronic liver diseases, AFP concentration is also slightly increased. DCP is an abnormal prothrombin that has been used for clinical HCC screening. High DCP level reflects a poor prognosis^7^. DCP sensitivity for early and small HCC (<2 cm) is only 56.5%^8^. AFP-L3 is a fucosylated species of AFP, and the serum concentration of AFP-L3 is associated with poor HCC differentiation^9^. Novel serum biomarkers are urgently required to increase the sensitivity and specificity of serum biomarker screening for early HCC diagnosis.

Mass spectrometry-based proteomic approaches have evolved as powerful tools to discover novel biomarkers^10^^,^^11^. However, identification of potential protein biomarkers from biofluid samples, such as serum and plasma, remains challenging because of their large protein concentration range. Efforts have been made to simplify serum samples via affinity chromatography, either by removing abundant proteins from the serum or enriching a subproteome with a common chemical structural feature^12^^-^^14^, e.g., affinity depletion using antibody-conjugated materials^12^^,^^13^. The interest in abnormal protein glycosylation research is increasing. The currently known HCC biomarkers, namely, AFP, AFP-L3, DCP, and GP73, are all glycoproteins. Many biomarkers clinically used for cancer diagnosis are also glycoproteins, such as prostate-specific antigen in prostate cancer^15^; Her2/neu in breast cancer^16^; CA-125 in ovarian cancer^17^; and CEA in colorectal, breast, pancreatic, and lung cancer^18^. Therefore, targeting glycoproteins in the serum can enrich the potential biomarkers while reducing the serum sample complexity for in-depth proteome analysis.

In this study, lectin affinity chromatography (LAC) was used to enrich glycoproteins from blood samples of 40 healthy volunteers and 40 HCC patients. Stable isotope dimethyl labeling and 2D liquid chromatography (LC) separation were used for quantitative mass spectrometric analysis to examine the differential levels of the detected proteins. More than 2,000 proteins were characterized in the serum. A panel of proteins exhibited significant changes in relative abundances between the HCC and control samples. The expression patterns of fibrinogen gamma chain (FGG), FOS-like antigen 2 (FOSL2), and α-1,6-mannosylglycoprotein 6-β-N-acetylglucosaminyltransferase B (MGAT5B) were validated by Western blot.

## Materials and methods

### Serum collection

The study was approved by the Ethics Committee of Tianjin Medical University. Serum samples were processed from each individual by using a 12G BD Vacutainer Safety-Lok^TM^ blood collection system. After collection, samples were immediately placed on ice and allowed to stand for 30 min. Samples were then centrifuged at 3,000 rpm for 15 min and stored at −80 °C until analysis. A total of 40 HCC serum samples were collected and divided into two groups. Each serum cohort, which consisted of 20 HCC samples and 20 cases of age- and gender-matched normal control cohort, was pooled for quantitative glycoproteomic analysis. HCC diagnoses were confirmed through histopathologic study.

### Reagents and materials

Iodoacetamide, N-acetyl-D-glucosamine, methyl-R-D-mannopyranoside, methyl-R-D-glucopyranoside, manganese chloride tetrahydrate, formaldehyde, deuterated formaldehyde, and sodium cyanoborohydride were purchased from Sigma-Aldrich (St. Louis, MO). Agarose-bound Concanavalin A (Con A, 6 mg lectin/mL gel) and wheat germ agglutinin (WGA, 7 mg lectin/mL gel) were purchased from Vector Laboratories (Burlingame, CA). Dithiothreitol (DTT) and sequencing grade modified trypsin were purchased from Promega (Madison, WI). Antibodies used in Western blot were all obtained from Santa Cruz Biotechnology (Dallas, TX).

### Lectin affinity chromatography (LAC)

Lectin affinity columns were prepared by adding 400 μL each of Con A and WGA slurry to empty Micro Bio-Spin columns (Bio-Rad Laboratories, Hercules, CA), as reported by Wei *et al*.^19^. Con A exhibited a high affinity to high-mannose type N-glycans, whereas WGA was selective for N-acetyl-glucosamine (GlcNAc). Up to 40 mL of pooled serum was diluted 10 times with the binding buffer (20 mM Tris, 0.15 M NaCl, 1 mM CaCl_2_, 1 mM MnCl_2_, pH 7.4) and loaded onto the lectin affinity columns. After shaking for 6 h, unretained proteins were discarded, and lectin beads were washed with 2.5 mL of binding buffer. The captured glycoproteins were eluted with 2 mL of elution buffer (10 mM Tris, 0.075 M NaCl, 0.25 M Nacetyl-D-glucosamine, 0.17 M methyl-R-D-mannopyranoside, and 0.17 M methyl-R-D-glucopyranoside). The eluted fraction was concentrated using a 10 kDa Centricon Ultracel YM-10 filter (Millipore, Billerica, MA). BCA assays were performed to measure the protein concentration.

### Protein digest and stable isotopic labeling

Concentrated samples were denatured with 6 M urea in 0.2 M sodium acetate buffer (pH 8) and reduced by incubation with 10 mM DTT at 37 °C for 1 h. Reduced proteins were alkylated for 1 h in darkness with 40 mM iodoacetamide. Alkylation reaction was quenched by adding DTT to a final concentration of 50 mM. Samples were diluted to a final concentration of 1 M urea. Trypsin was added at a 50:1 protein-to-trypsin mass ratio, and samples were incubated at 37 °C overnight. Sodium cyanoborohydride was added to the protein digest to a final concentration of 50 mM. Samples were labeled with either 0.2 mM formaldehyde or 0.2 mM deuterated-formaldehyde. The mixed peptides were vortexed and incubated at 37 °C for 1 h. Up to 2 M NH_4_OH was added to quench the reaction, and the mixture was immediately dried using SpeedVac. Finally, samples were reconstituted in water.

### High-pH reversed-phase liquid chromatography (RPLC)

Equal amounts of light- and heavy-labeled samples were combined and separated using Waters HPLC C18 columns with high pH stability at a flow rate of 150 μL/min. The peptides were eluted with a 40 min gradient 5%-45% buffer B (buffer A: 100 mM ammonium formate, pH 10; buffer B: acetonitrile). Fractions were collected every 3 min for 60 min. Collected fractions were dried using SpeedVac and reconstituted in 20 μL of 0.1% formic acid. Up to 2 μL of each of the 10 fractions containing peptides was subjected to LC-MS/MS.

### LC-MS/MS and data analysis

A nanoUPLC system (Waters, Milford, MA) was used to separate the tryptic peptides. Samples were loaded on a C18 trap column and flushed with mobile phase A (0.1% formic acid in H_2_O) at 5 μL/min for 10 min before being delivered to a nanoUPLC column (C18, 150 mm × 0.075 mm × 1.7 μm). The peptides were eluted using a 7%-45% B gradient (0.1% formic acid in acetonitrile) over 90 min into a nano-electrospray ionization (nESI) LTQ Orbitrap mass spectrometer (ThermoFisher Scientific, Waltham, MA). The mass spectrometer was operated in data-dependent mode, in which an initial FT scan recorded the mass range of m/z 350-2,000. The spray voltage was set between 1.8 and 2.0 kV, and the mass resolution used for MS scan was 30,000. For tandem mass analysis, the eight most abundant ions were automatically selected for collisionally activated dissociation. The mass window for precursor ion selection was m/z ±1, and the normalized collision energy was set at 35% for MS/MS. Dynamic exclusion parameters were set as follows: exclusion duration was 60 s, and exclusion mass width was 0.01% relative to reference mass.

Raw data were searched through UniProt human protein database containing 98,778 sequence entries via SEQUEST algorithm embedded in the Protein Discoverer version 1.3 software (ThermoFisher Scientific, Waltham, MA). The following parameters were applied during the database search: 10 ppm precursor mass error tolerance, 1 Da fragment mass error tolerance, static modifications of carbamidomethylation for all cysteine residues, dimethylation for formaldehyde-labeled sample (+28 Da) or deuterated-formaldehyde-labeled (+32 Da) lysine, and N-terminus. False discovery rate of <0.05 was used as filtering criteria for all identified peptides. Proteins identified with the same set of peptides were grouped and treated as one. Protein Discoverer was used for relative quantification. Two groups of pooled serum samples were analyzed, each of which contained serum collected from 20 patients or healthy donors. Each sample was analyzed thrice. Protein identification information was imported to PANTHER database for gene ontology analysis (http://www.pantherdb.org).

## Results

### Enrichment of glycoproteins from serum

**Figure 1:**
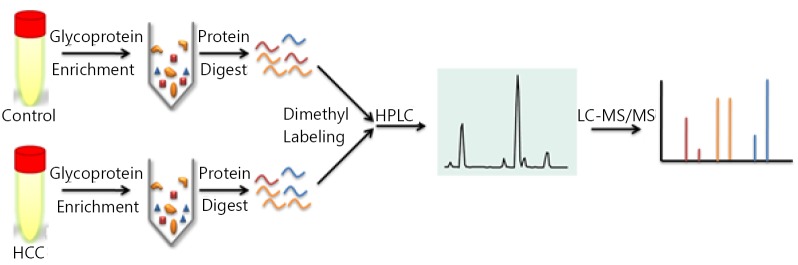
Quantitative proteomic schematic of serum glycoproteins. Glycoproteins in serum samples were enriched using lectin affinity chromatography and digested by trypsin then labeled using isotopic formaldehyde, followed by 2D RP-RP LC-MS/MS.

**Figure 2:**
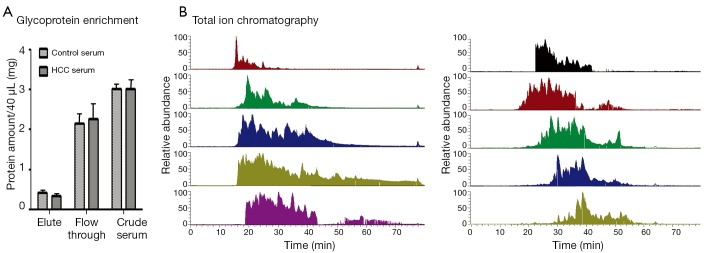
Sample serum glycoprotein preparation. (A) The protein content in the crude serum, flow-through from LAC column, and the eluted fraction that contained glycoproteins, as measured using BCA assay. (B) LC-MS/MS total ion chromatogram from 10 fractions separated by high pH RP-LC separation.

Direct proteome analysis in serum was challenging because of the presence of highly abundant proteins. The analyzing strategy employed in this study is shown in **Figure 1**. A lectin affinity column with a broad sugar selection spectrum was used to purify the glycoproteins from the serum. Two lectin types were combined to extend the coverage of glycoprotein enrichment. Con A showed a high affinity to high-mannose type N-glycans, whereas WGA was selective for GlcNAc. Majority of the serum albumin and other abundant proteins were removed from the serum, which reduced the sample complexity and increased the detection sensitivity for proteins with low abundance. Approximately 80% of the total serum proteins were removed after LAC purification (**Figure 2A**). Glycoprotein enrichment effectiveness was further demonstrated by the mass spectrometry results. With lectin selection, albumin no longer appeared on the top of the protein identification list, although a moderate number of albumin peptides can still be detected probably because of nonspecific and secondary binding events.

### Relative glycoprotein quantification between HCC and control serum

A total of 40 HCC and control samples were analyzed to investigate the differential serum glycoprotein expression induced by HCC. Patient information is provided in **Table 1**. Multidimensional separations were extensively applied in proteomic studies to reduce the complexity of samples. In the present study, RPLC was used in both dimensions of the separation under significantly different pH conditions. Moreover, 2D RP-RP LC-MS/MS demonstrated great orthogonality in peptide separations because of charge changes in acidic and basic amino acid side chains under different pH conditions^20^. The use of RP as first dimension provided higher separation resolution and higher peptide recovery than strong cation exchange. Different peptide total ion chromatogram profiles were observed between the fractions collected from the first dimension of RPLC separation **(****Figure 2B**). The widespread of peaks in all 10 fractions in the second dimension provided great resolution to enhance proteomic detection.

**Table 1:** Clinical information of HCC samples and normal controls

Items	HCC	Normal control
Sample No.	40	40
Age	57.5±10.8	55.3±14.3
Gender		
Female	10	21
Male	30	19
HBsAg		
Positive	23	
Negative	17	
HCV		
Positive	4	
Negative	36	
ALT (U/L)	60.4±71.8	
AST (U/L)	60.0±63.3	
ALB (g/L)	40.8±4.6	
TBIL (μmol/L)	31.1±59.5	
BCLC stages		
A, B	23	
C, D	17	
Metastasis		
Positive	11	
Negative	29	

**Figure 3:**
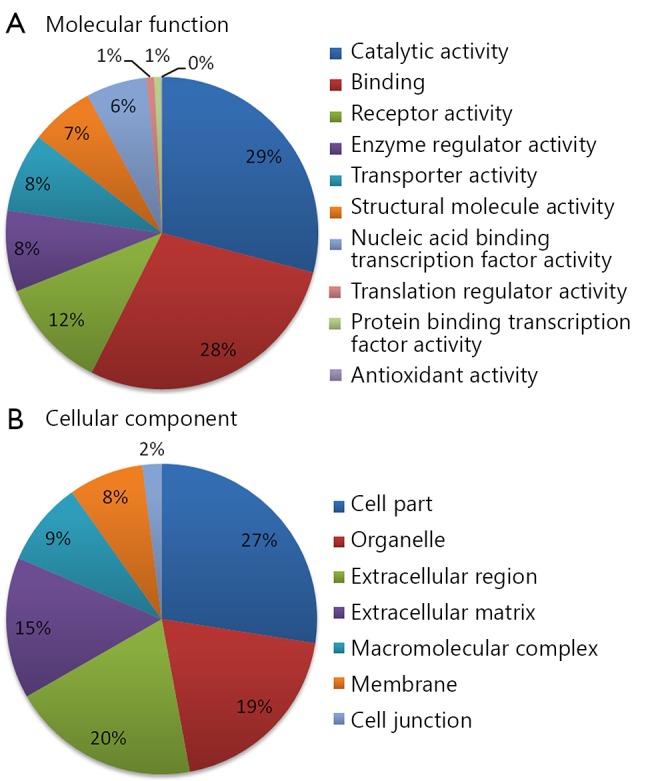
Gene ontology analysis of identified serum proteins. (A) Diagram showing the molecular functions of the identified proteins. (B) Diagram showing the cellular components of the identified proteins. Annotation information was acquired through PANTHER database (http://www.pantherdb.org).

Using 2D RP-RP LC-MS/MS method, 2,280 protein groups were identified from the two groups of pooled serum samples (**Table S1** in the supplementary materials, available with the full text of this article at www.cancerbiomed.org). Systematic gene ontology analysis was performed using PANTHER database. Molecular function analysis revealed that the majority of the identified serum proteins demonstrated catalytic (29%), binding (28%), and receptor activities (12%) (**Figure 3**). Cellular component analysis showed that most of the identified serum proteins probably originated from tissue leakage, including cell parts (27%) and organelles (19%). Another large portion resulted from the extracellular region (20%) and extracellular matrix (15%).

Quantitative proteomic analysis was performed to calculate the ratios of the identified serum glycoproteins between HCC and control. Given the low glycoprotein concentration, proteins identified with only one unique peptide were kept in the list. However, further validation is required. Twofold cutoff threshold showed that 36 proteins were up-regulated in the HCC serum, whereas 19 proteins were down-regulated (**Table 2**). AFP, the clinically used marker for HCC diagnosis, was detected in HCC serum only.

**Table 2:** List of differently expressed glycoproteins in human serum from patients with hepatocellular carcinoma

UniProt accession	Protein description	Unique peptides	PSMs	Ratio HCC/control	Stdev
Up-regulated in HCC					
B4DJF2	14-3-3 protein epsilon (14-3-3E)	1	5	HCC	NA
B4DMW9	Alpha-fetoprotein	6	12	HCC	NA
B3KXI1	Homo sapiens papilin, proteoglycan-like sulfated glycoprotein (PAPLN), mRNA	2	2	HCC	NA
C9JEU5	Fibrinogen gamma chain	2	4	HCC	NA
Q96JS0	Tubby homologue	1	6	HCC	NA
Q59HA1	Golgin 97 variant (Fragment)	1	2	HCC	NA
Q3V5L5	α-1,6-mannosylglycoprotein 6-β-N-acetylglucosaminyltransferase B	1	6	HCC	NA
B9EJA8	Mannose receptor, C type 1-like 1	4	12	33.79	20.94
E9PDD2	Vascular cell adhesion protein 1	3	5	19.04	NA
Q2LD37	KIAA1109	1	2	13.04	2.18
D3DP13	Fibrinogen β chain, isoform CRA_e	4	10	12.88	8.97
D6CHE9	Proteinase 3	1	4	12.41	3.12
Q5XTR9	Hemoglobin delta-beta fusion protein	2	24	7.30	2.38
P01833	Polymeric immunoglobulin receptor	8	22	6.02	2.46
P04275	von Willebrand factor	16	60	5.68	2.81
C9JCN8	Fos-related antigen 2	1	11	5.60	0.82
Q00532	Cyclin-dependent kinase-like 1	2	3	4.95	0.19
E7EU22	cytoskeleton-associated proteins	3	14	3.76	1.81
Q2L9S7	Alpha-1-antitrypsin MBrescia variant	8	3,460	3.57	1.56
P02786	Transferrin receptor protein 1	5	8	3.52	1.15
A6NKL6	Transmembrane protein 200C	1	6	3.45	1.29
B7Z461	Serine/threonine-protein kinase PCTAIRE-1	2	6	3.38	0.36
O75128	Protein cordon-bleu	1	2	3.34	0.69
P20061	Transcobalamin-1	1	5	3.29	0.14
P02042	Hemoglobin subunit δ	8	278	2.94	1.35
Q5KTC1	SSF-TRPM2	2	4	2.81	0.84
Q5T619	Zinc finger protein 648	1	4	2.81	0.23
B4DVE1	Galectin-3-binding protein	3	12	2.71	0.73
Q59FC6	Tumor rejection antigen (Gp96) 1 variant	4	7	2.54	0.91
Q96JG9	Zinc finger protein 469	3	8	2.51	0.53
E5FY30	Glycoprotein Ib (Fragment)	4	12	2.51	0.44
P02750	Leucine-rich alpha-2-glycoprotein	10	89	2.41	0.65
Q68CK4	Leucine-rich alpha-2-glycoprotein	10	74	2.09	0.38
B4E0R8	LOC100499484	1	5	2.08	0.63
E9PGL6	The fibulin family of extracellular matrix glycoproteins	4	11	2.08	0.57
P15144	Aminopeptidase N	5	9	2.02	0.47
Down-regulated in HCC					
Q9UGM5	Fetuin-B	5	16	0.49	0.15
P27169	Serum paraoxonase/arylesterase 1	10	126	0.47	0.09
B3KPF0	Insulin-like growth factor-binding protein 3	3	5	0.46	0.04
P06276	Cholinesterase	13	49	0.46	0.14
P02743	Serum amyloid P-component	8	67	0.45	0.12
B0AZL7	Insulin-like growth factor-binding protein complex acid labile chain	10	26	0.43	0.10
P02656	Apolipoprotein C-III	4	50	0.39	0.23
C9JPG5	Semaphorin-3F	1	19	0.37	0.08
B4DFJ1	TERF1-interacting nuclear factor 2	2	3	0.30	NA
Q63ZY3	KN motif and ankyrin repeat domain-containing protein 2	2	2	0.26	0.11
A5YAK2	Apolipoprotein C-IV	2	6	0.24	0.12
A6XMH1	Transthyretin	2	8	0.24	0.11
Q16473	Putative tenascin-XA	1	2	0.22	0.005
Q3ZB85	PCDHB9 protein (Fragment)	1	2	0.20	0.007
B7ZLP8	TARSL2 protein	1	5	0.04	0.007
B3KU02	Homo sapiens sorting nexin 26 (SNX26)	1	45	0.03	0.008
B4DHJ6	Ankyrin repeat domain-containing protein 50	2	3	0.02	0.003
D9ZGF8	Rho-associated, coiled-coil containing protein kinase 1	2	4	Control	NA
Q7M4S4	Granulocyte inhibitory protein	1	2	Control	NA

HCC, the protein was only detected in the serum from HCC patient; Control, the protein was only detected in the control serum; NA, not available.

### Validation of three differently expressed proteins

**Figure 4:**
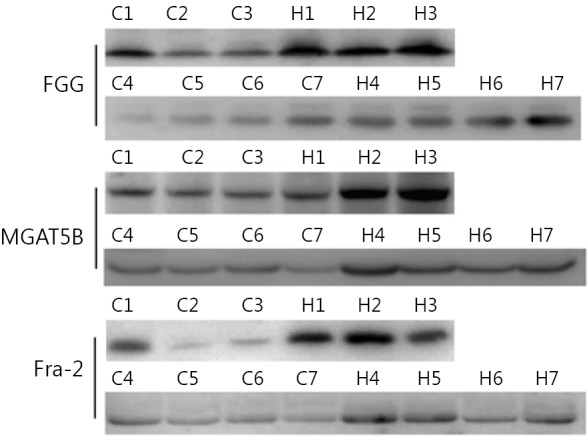
Western blot analysis of identified differential serum proteins. Serum samples were first clarified by depletion of highly abundant proteins. Seven groups of HCC and control serum samples were analyzed. H, HCC; C, control.

Besides AFP, other glycoproteins with differential expression levels were also found: FGG, FOSL2, and MGAT5B. To validate this observation in quantitative MS experiments, Western blot analysis was performed to test the level of the three up-regulated glycoproteins in seven groups of serum samples. Abundant protein depletion was performed to remove albumin and IgG prior to Western blot analysis. FGG, MGAT5B, and FOSL2 were detected using Western blot (**Figure 4**). Consistent with the mass spectrometry analysis results, the three proteins were up-regulated in the seven HCC serum samples compared with the normal control. Further studies must be performed on large numbers of clinical samples by using ELISA to evaluate the potential usage of FGG, FOSL2, and MGAT5B as serum biomarkers.

## Discussion

Novel biomarker discovery using traditional biological assays is time consuming. With the great technical development of mass spectrometry, quantitative proteomics has become an essential tool to determine biomarkers. A large effort has been devoted to mining novel serum HCC biomarkers over the last decade^3^^,^^21^^,^^22^. The technology used in these studies evolved from 2D electrophoresis to multidimensional LC-MS/MS. Different analytical methods can provide complementary information, which can validate previous reports and provide new opportunities to discover novel biomarkers. For example, Pan *et al*.^23^ reported that two commonly used glycoprotein enrichment methods, namely, LAC and hydrazide coupling enrichment, can purify different glycoprotein pools from serum. Hydrazide coupling and label-free quantification were used in a previous study to examine the glycoprotein expression in HCC serum. In the present study, a different strategy was employed by combining LAC and dimethylation isotopic labeling. Several proteins, such as AFP, fibrinogen beta chain, polymeric-immunoglobulin receptor, and insulin-like growth factor-binding protein 3, were detected and quantified by both LAC and dimethylation isotopic labeling. However, majority of the detected proteins were not reported in previous research.

Three differential glycoproteins were validated using Western blot. FGG role was suggested for other tumor types^24^^,^^25^. Zhu *et al*.^26^ demonstrated that FGG was significantly up-regulated at the mRNA level in the HCC cell lines and HCC tissues. Plasma fibrinogen progressively increased with the tumor clinical stage of HCC patients^26^^,^^27^. The higher FGG serum concentration in HCC patients than in healthy people may be attributed to the higher FGG expression in HCC and increased fibrinogen degradation^28^.

MGAT5B (also reported as GnT-Vb or GnT-IX), an MGAT5 isozyme, demonstrated a broad transfer activity toward GlcNAc β 1,2-Man α 1-Ser/Thr, which formed a 2,6-branched structure in brain O-mannosyl glycan^29^. MGAT5B was exclusively detected in neural tissues and testes^30^^,^^31^. Lange *et al*.^32^ detected an MGAT5B expression in prostate cancer cells and xenografts, whereas MGAT5B was absent in the primary prostate epithelial cells and normal human prostate. Liu *et al*.^33^ observed that MGAT5B was up-regulated in metastatic HCC clinical cancer specimens, and the trend was the same in human HCC cell lines and orthotopic xenograft tumors. The current results also revealed increased MGAT5B levels in the serum of HCC patients compared with normal controls. Additionally, MGAT5B was extensively expressed in different HCC cell lines (**Figure S1** in the supplementary materials, available with the full text of this article at www.cancerbiomed.org). More studies must be conducted in the future to understand the functions of MGAT5B in HCC development.

FOSL2 belongs to the activator protein 1 (Jun and Fos family) transcription factors, which regulate gene expression in cell proliferation, differentiation, inflammation, and malignant transformation^34^. FOSL2 overexpression was associated with the progression of various human tumor types, including breast cancer^35^, ovarian carcinoma^36^, salivary gland tumors^37^, colorectal cancer^36^^-^^38^, and adult T-cell leukaemia^39^. FOSL2 is one of the genes affected by the characteristic t(2;5) translocation in tumor cells^40^. The present study suggests for the first time that FOSL2 is up-regulated in HCC serum. However, additional studies are needed to confirm the findings and determine whether such up-regulation is caused by FOSL2 overexpression in HCC tumor tissues.

## Figures and Tables

**Figure S1:**
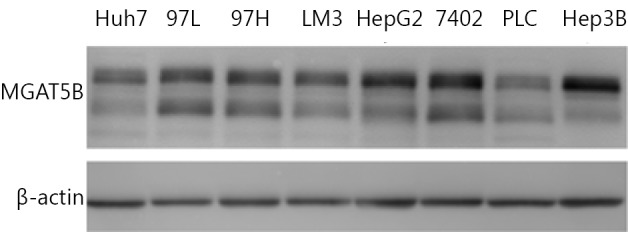
Western blotting analysis of MGAT5B from different HCC cell lines. The same amount of protein was loaded for each cell lystae, and β-actin was used as internal standard.

## Supplementary materials

This has been provided by the authors to give readers additional information about their work. Supplement to: Gao HJ, Chen YJ, Zuo D, Xiao MM, Li Y, Guo H, Zhang N, Chen RB. Quantitative proteomic analysis for high-throughput screening of differential glycoproteins in hepatocellular carcinoma serum. Cancer Biol Med 2015;12:246-254. doi: 10.7497/j.issn.2095-3941.2015.0010

**Table S1:** A total of 2,280 protein groups identified from the two groups of pooled serum samples

Accession	Description	Σ Coverage	Σ# Proteins	Σ# Unique peptides	Σ# Peptides	# AAs	MW (kDa)	calc. pI
C0JYY2	Apolipoprotein B (Including Ag(X) antigen) OS=Homo sapiens GN=APOB PE=4 SV=1 - [C0JYY2_HUMAN]	38.07	15	149	151	4,563	515.2	7.05
P01024	Complement C3 OS=Homo sapiens GN=C3 PE=1 SV=2 - [CO3_HUMAN]	77.87	5	118	118	1,663	187.0	6.40
P01023	Alpha-2-macroglobulin OS=Homo sapiens GN=A2M PE=1 SV=3 - [A2MG_HUMAN]	77.95	5	86	101	1,474	163.2	6.46
P01031	Complement C5 OS=Homo sapiens GN=C5 PE=1 SV=4 - [CO5_HUMAN]	36.99	2	48	48	1,676	188.2	6.52
P00450	Ceruloplasmin OS=Homo sapiens GN=CP PE=1 SV=1 - [CERU_HUMAN]	50.23	8	44	44	1,065	122.1	5.72
A7E2V2	Complement component 4A (Rodgers blood group) OS=Homo sapiens GN=C4A PE=2 SV=1 - [A7E2V2_HUMAN]	53.78	22	36	70	1,744	192.7	7.14
P02768	Serum albumin OS=Homo sapiens GN=ALB PE=1 SV=2 - [ALBU_HUMAN]	53.20	13	30	30	609	69.3	6.28
A2RTY6	Inter-alpha (Globulin) inhibitor H2 OS=Homo sapiens GN=ITIH2 PE=2 SV=1 - [A2RTY6_HUMAN]	44.40	7	29	29	946	106.4	6.86
B4E1Z4	cDNA FLJ55673, highly similar to Complement factor B (EC 3.4.21.47) OS=Homo sapiens PE=2 SV=1 - [B4E1Z4_HUMAN]	29.38	14	28	36	1,266	140.9	7.18
P01008	Antithrombin-III OS=Homo sapiens GN=SERPINC1 PE=1 SV=1 - [ANT3_HUMAN]	59.91	8	24	24	464	52.6	6.71
P02790	Hemopexin OS=Homo sapiens GN=HPX PE=1 SV=2 - [HEMO_HUMAN]	68.18	7	23	23	462	51.6	7.02
P01011	Alpha-1-antichymotrypsin OS=Homo sapiens GN=SERPINA3 PE=1 SV=2 - [AACT_HUMAN]	58.87	2	21	21	423	47.6	5.52
P43652	Afamin OS=Homo sapiens GN=AFM PE=1 SV=1 - [AFAM_HUMAN]	39.40	2	21	21	599	69.0	5.90
P03952	Plasma kallikrein OS=Homo sapiens GN=KLKB1 PE=1 SV=1 - [KLKB1_HUMAN]	32.60	10	21	21	638	71.3	8.22
B2R7F8	cDNA, FLJ93426, highly similar to Homo sapiens plasminogen (PLG), mRNA OS=Homo sapiens PE=2 SV=1 - [B2R7F8_HUMAN]	31.73	30	20	21	810	90.5	7.24
B4E1D8	cDNA FLJ51597, highly similar to C4b-binding protein alpha chain OS=Homo sapiens PE=2 SV=1 - [B4E1D8_HUMAN]	40.86	5	19	19	536	60.4	6.65
P05156	Complement factor I OS=Homo sapiens GN=CFI PE=1 SV=2 - [CFAI_HUMAN]	34.13	8	17	17	583	65.7	7.50
P00734	Prothrombin OS=Homo sapiens GN=F2 PE=1 SV=2 - [THRB_HUMAN]	33.92	8	17	17	622	70.0	5.90
B2R950	cDNA, FLJ94213, highly similar to Homo sapiens pregnancy-zone protein (PZP), mRNA OS=Homo sapiens PE=2 SV=1 - [B2R950_HUMAN]	22.13	5	16	30	1,482	163.8	6.38
P02647	Apolipoprotein A-I OS=Homo sapiens GN=APOA1 PE=1 SV=1 - [APOA1_HUMAN]	63.67	7	16	17	267	30.8	5.76
B7Z550	Complement component 8, beta polypeptide, isoform CRA_b OS=Homo sapiens GN=C8B PE=2 SV=1 - [B7Z550_HUMAN]	32.70	6	16	17	529	60.1	7.77
A8K2N0	cDNA FLJ77835, highly similar to Homo sapiens complement component 1, s subcomponent (C1S), transcript variant 2, mRNA OS=Homo sapiens PE=2 SV=1 - [A8K2N0_HUMAN]	30.38	5	16	17	688	76.6	4.98
P05546	Heparin cofactor 2 OS=Homo sapiens GN=SERPIND1 PE=1 SV=3 - [HEP2_HUMAN]	36.27	2	16	16	499	57.0	6.90
P04275	von Willebrand factor OS=Homo sapiens GN=VWF PE=1 SV=4 - [VWF_HUMAN]	6.51	3	16	16	2,813	309.1	5.48
B4E1H2	cDNA FLJ58564, highly similar to Plasma protease C1 inhibitor OS=Homo sapiens PE=2 SV=1 - [B4E1H2_HUMAN]	36.16	5	16	16	448	49.7	6.54
A8MXF4	Uncharacterized protein OS=Homo sapiens GN=ITIH3 PE=4 SV=1 - [A8MXF4_HUMAN]	33.88	5	16	16	670	75.0	5.86
P02649	Apolipoprotein E OS=Homo sapiens GN=APOE PE=1 SV=1 - [APOE_HUMAN]	58.99	8	15	16	317	36.1	5.73
P02748	Complement component C9 OS=Homo sapiens GN=C9 PE=1 SV=2 - [CO9_HUMAN]	32.56	1	15	15	559	63.1	5.59
P07225	Vitamin K-dependent protein S OS=Homo sapiens GN=PROS1 PE=1 SV=1 - [PROS_HUMAN]	25.89	17	15	15	676	75.1	5.67
B2R8I2	cDNA, FLJ93914, highly similar to Homo sapiens histidine-rich glycoprotein (HRG), mRNA OS=Homo sapiens PE=2 SV=1 - [B2R8I2_HUMAN]	30.10	3	15	15	525	59.5	7.44
P00738	Haptoglobin OS=Homo sapiens GN=HP PE=1 SV=1 - [HPT_HUMAN]	64.78	6	14	26	406	45.2	6.58
Q53HT9	Complement component 1, r subcomponent variant (Fragment) OS=Homo sapiens PE=2 SV=1 - [Q53HT9_HUMAN]	32.62	7	14	17	705	80.2	6.44
P80108	Phosphatidylinositol-glycan-specific phospholipase D OS=Homo sapiens GN=GPLD1 PE=1 SV=3 - [PHLD_HUMAN]	20.83	1	14	14	840	92.3	6.37
B2R815	cDNA, FLJ93695, highly similar to Homo sapiens serpin peptidase inhibitor, clade A (alpha-1 antiproteinase, antitrypsin), member 4 (SERPINA4), mRNA OS=Homo sapiens PE=2 SV=1 - [B2R815_HUMAN]	37.24	2	14	14	427	48.5	7.75
B4DZ36	cDNA FLJ58441, highly similar to Attractin OS=Homo sapiens PE=2 SV=1 - [B4DZ36_HUMAN]	13.49	4	14	14	1,156	129.7	6.87
P12259	Coagulation factor V OS=Homo sapiens GN=F5 PE=1 SV=4 - [FA5_HUMAN]	7.69	3	13	14	2,224	251.5	6.05
O43866	CD5 antigen-like OS=Homo sapiens GN=CD5L PE=1 SV=1 - [CD5L_HUMAN]	42.94	1	13	13	347	38.1	5.47
P10909	Clusterin OS=Homo sapiens GN=CLU PE=1 SV=1 - [CLUS_HUMAN]	30.96	13	13	13	449	52.5	6.27
P02765	Alpha-2-HS-glycoprotein OS=Homo sapiens GN=AHSG PE=1 SV=1 - [FETUA_HUMAN]	51.77	5	13	13	367	39.3	5.72
P25311	Zinc-alpha-2-glycoprotein OS=Homo sapiens GN=AZGP1 PE=1 SV=2 - [ZA2G_HUMAN]	44.30	5	13	13	298	34.2	6.05
P06276	Cholinesterase OS=Homo sapiens GN=BCHE PE=1 SV=1 - [CHLE_HUMAN]	26.91	11	13	13	602	68.4	7.42
P22792	Carboxypeptidase N subunit 2 OS=Homo sapiens GN=CPN2 PE=1 SV=2 - [CPN2_HUMAN]	30.83	1	12	12	545	60.6	5.99
B4E1B3	cDNA FLJ53950, highly similar to Angiotensinogen OS=Homo sapiens PE=2 SV=1 - [B4E1B3_HUMAN]	37.55	14	12	12	466	51.0	6.16
P15169	Carboxypeptidase N catalytic chain OS=Homo sapiens GN=CPN1 PE=1 SV=1 - [CBPN_HUMAN]	31.44	2	11	11	458	52.3	7.34
D9IWP9	Beta-2-glycoprotein I (Fragment) OS=Homo sapiens PE=2 SV=1 - [D9IWP9_HUMAN]	47.55	3	11	11	326	36.2	8.00
A8K050	cDNA FLJ75376, highly similar to Homo sapiens peptidoglycan recognition protein L (PGLYRP) mRNA OS=Homo sapiens PE=2 SV=1 - [A8K050_HUMAN]	37.67	3	11	11	576	62.1	7.37
B4E1B2	cDNA FLJ53691, highly similar to Serotransferrin OS=Homo sapiens PE=2 SV=1 - [B4E1B2_HUMAN]	67.55	3	10	42	678	74.8	7.12
P01009	Alpha-1-antitrypsin OS=Homo sapiens GN=SERPINA1 PE=1 SV=3 - [A1AT_HUMAN]	68.66	10	10	31	418	46.7	5.59
P08697	Alpha-2-antiplasmin OS=Homo sapiens GN=SERPINF2 PE=1 SV=3 - [A2AP_HUMAN]	30.14	5	10	10	491	54.5	6.29
P07357	Complement component C8 alpha chain OS=Homo sapiens GN=C8A PE=1 SV=2 - [CO8A_HUMAN]	20.55	2	10	10	584	65.1	6.47
P00742	Coagulation factor X OS=Homo sapiens GN=F10 PE=1 SV=2 - [FA10_HUMAN]	24.59	3	10	10	488	54.7	5.94
P51884	Lumican OS=Homo sapiens GN=LUM PE=1 SV=2 - [LUM_HUMAN]	32.84	2	10	10	338	38.4	6.61
P27169	Serum paraoxonase/arylesterase 1 OS=Homo sapiens GN=PON1 PE=1 SV=3 - [PON1_HUMAN]	36.06	10	10	10	355	39.7	5.22
P04004	Vitronectin OS=Homo sapiens GN=VTN PE=1 SV=1 - [VTNC_HUMAN]	29.92	2	10	10	478	54.3	5.80
E9PF24	Uncharacterized protein OS=Homo sapiens GN=APOL1 PE=4 SV=1 - [E9PF24_HUMAN]	30.53	11	10	10	380	42.1	5.80
E7EX03	Uncharacterized protein OS=Homo sapiens GN=CNDP1 PE=4 SV=1 - [E7EX03_HUMAN]	26.54	4	10	10	486	54.5	5.24
B0AZL7	cDNA, FLJ79457, highly similar to Insulin-like growth factor-binding proteincomplex acid labile chain OS=Homo sapiens PE=2 SV=1 - [B0AZL7_HUMAN]	20.50	5	10	10	605	66.0	6.79
P07996	Thrombospondin-1 OS=Homo sapiens GN=THBS1 PE=1 SV=2 - [TSP1_HUMAN]	10.85	5	10	10	1,170	129.3	4.94
P04217	Alpha-1B-glycoprotein OS=Homo sapiens GN=A1BG PE=1 SV=4 - [A1BG_HUMAN]	54.14	4	9	17	495	54.2	5.86
P02760	Protein AMBP OS=Homo sapiens GN=AMBP PE=1 SV=1 - [AMBP_HUMAN]	31.53	2	9	10	352	39.0	6.25
P36955	Pigment epithelium-derived factor OS=Homo sapiens GN=SERPINF1 PE=1 SV=4 - [PEDF_HUMAN]	26.08	1	9	9	418	46.3	6.38
P55058	Phospholipid transfer protein OS=Homo sapiens GN=PLTP PE=1 SV=1 - [PLTP_HUMAN]	23.12	6	9	9	493	54.7	7.01
P05543	Thyroxine-binding globulin OS=Homo sapiens GN=SERPINA7 PE=1 SV=2 - [THBG_HUMAN]	27.71	1	9	9	415	46.3	6.30
B7Z2X4	cDNA FLJ53327, highly similar to Gelsolin OS=Homo sapiens PE=2 SV=1 - [B7Z2X4_HUMAN]	16.60	16	9	9	705	77.7	5.69
P02751	Fibronectin OS=Homo sapiens GN=FN1 PE=1 SV=4 - [FINC_HUMAN]	33.61	24	8	55	2,386	262.5	5.71
D3DPF9	Titin, isoform CRA_b OS=Homo sapiens GN=TTN PE=4 SV=1 - [D3DPF9_HUMAN]	0.50	21	8	11	26,926	2,991.2	6.74
P06727	Apolipoprotein A-IV OS=Homo sapiens GN=APOA4 PE=1 SV=3 - [APOA4_HUMAN]	24.49	35	8	9	396	45.4	5.38
P69905	Hemoglobin subunit alpha OS=Homo sapiens GN=HBA1 PE=1 SV=2 - [HBA_HUMAN]	82.39	17	8	8	142	15.2	8.68
Q04756	Hepatocyte growth factor activator OS=Homo sapiens GN=HGFAC PE=1 SV=1 - [HGFA_HUMAN]	16.79	2	8	8	655	70.6	7.24
Q92954	Proteoglycan 4 OS=Homo sapiens GN=PRG4 PE=1 SV=2 - [PRG4_HUMAN]	7.34	6	8	8	1,404	151.0	9.50
P02743	Serum amyloid P-component OS=Homo sapiens GN=APCS PE=1 SV=2 - [SAMP_HUMAN]	32.29	1	8	8	223	25.4	6.54
B2R9F2	cDNA, FLJ94361, highly similar to Homo sapiens serine (or cysteine) proteinase inhibitor, clade A(alpha-1 antiproteinase, antitrypsin), member 6 (SERPINA6), mRNA OS=Homo sapiens PE=2 SV=1 - [B2R9F2_HUMAN]	24.94	2	8	8	405	45.1	6.04
O75636	Ficolin-3 OS=Homo sapiens GN=FCN3 PE=1 SV=2 - [FCN3_HUMAN]	29.10	8	8	8	299	32.9	6.67
P01833	Polymeric immunoglobulin receptor OS=Homo sapiens GN=PIGR PE=1 SV=4 - [PIGR_HUMAN]	14.01	1	8	8	764	83.2	5.74
B4E1C2	Kininogen 1, isoform CRA_b OS=Homo sapiens GN=KNG1 PE=2 SV=1 - [B4E1C2_HUMAN]	40.06	2	7	25	644	71.9	6.81
B7Z549	cDNA FLJ56821, highly similar to Inter-alpha-trypsin inhibitor heavy chain H1 OS=Homo sapiens PE=2 SV=1 - [B7Z549_HUMAN]	46.82	2	7	22	677	75.5	6.96
P01591	Immunoglobulin J chain OS=Homo sapiens GN=IGJ PE=1 SV=4 - [IGJ_HUMAN]	64.15	4	7	7	159	18.1	5.24
P01880	Ig delta chain C region OS=Homo sapiens GN=IGHD PE=1 SV=2 - [IGHD_HUMAN]	27.86	2	7	7	384	42.2	7.93
O00391	Sulfhydryl oxidase 1 OS=Homo sapiens GN=QSOX1 PE=1 SV=3 - [QSOX1_HUMAN]	12.99	5	7	7	747	82.5	8.92
B2RBZ5	cDNA, FLJ95778, highly similar to Homo sapiens serpin peptidase inhibitor, clade A (alpha-1 antiproteinase, antitrypsin), member 10 (SERPINA10), mRNA OS=Homo sapiens PE=2 SV=1 - [B2RBZ5_HUMAN]	17.34	2	7	7	444	50.6	8.51
P02763	Alpha-1-acid glycoprotein 1 OS=Homo sapiens GN=ORM1 PE=1 SV=1 - [A1AG1_HUMAN]	51.24	2	6	11	201	23.5	5.02
P19652	Alpha-1-acid glycoprotein 2 OS=Homo sapiens GN=ORM2 PE=1 SV=2 - [A1AG2_HUMAN]	50.25	3	6	11	201	23.6	5.11
Q8TCF0	LBP protein OS=Homo sapiens GN=LBP PE=2 SV=1 - [Q8TCF0_HUMAN]	16.56	2	6	6	477	52.9	6.76
P20851	C4b-binding protein beta chain OS=Homo sapiens GN=C4BPB PE=1 SV=1 - [C4BPB_HUMAN]	27.38	4	6	6	252	28.3	5.14
Q16610	Extracellular matrix protein 1 OS=Homo sapiens GN=ECM1 PE=1 SV=2 - [ECM1_HUMAN]	12.96	3	6	6	540	60.6	6.71
P00748	Coagulation factor XII OS=Homo sapiens GN=F12 PE=1 SV=3 - [FA12_HUMAN]	10.89	3	6	6	615	67.7	7.74
B7Z8U5	cDNA FLJ51606, highly similar to Hyaluronan-binding protein 2 (EC 3.4.21.-) OS=Homo sapiens PE=2 SV=1 - [B7Z8U5_HUMAN]	11.05	2	6	6	534	59.8	6.43
B4DMW9	cDNA FLJ51509, highly similar to Alpha-fetoprotein OS=Homo sapiens PE=2 SV=1 - [B4DMW9_HUMAN]	15.38	3	6	6	481	54.2	7.44
P00739	Haptoglobin-related protein OS=Homo sapiens GN=HPR PE=2 SV=2 - [HPTR_HUMAN]	49.14	2	5	17	348	39.0	7.09
Q6N041	Putative uncharacterized protein DKFZp686O16217 (Fragment) OS=Homo sapiens GN=DKFZp686O16217 PE=2 SV=1 - [Q6N041_HUMAN]	42.37	2	5	13	498	54.1	7.18
P04264	Keratin, type II cytoskeletal 1 OS=Homo sapiens GN=KRT1 PE=1 SV=6 - [K2C1_HUMAN]	11.80	3	5	7	644	66.0	8.12
P20929	Nebulin OS=Homo sapiens GN=NEB PE=1 SV=4 - [NEBU_HUMAN]	1.42	7	5	7	6,669	772.4	9.07
P02652	Apolipoprotein A-II OS=Homo sapiens GN=APOA2 PE=1 SV=1 - [APOA2_HUMAN]	61.00	1	5	5	100	11.2	6.62
P05090	Apolipoprotein D OS=Homo sapiens GN=APOD PE=1 SV=1 - [APOD_HUMAN]	29.63	4	5	5	189	21.3	5.15
P43251	Biotinidase OS=Homo sapiens GN=BTD PE=1 SV=2 - [BTD_HUMAN]	11.97	5	5	5	543	61.1	6.25
P07360	Complement component C8 gamma chain OS=Homo sapiens GN=C8G PE=1 SV=3 - [CO8G_HUMAN]	39.60	2	5	5	202	22.3	8.31
P00740	Coagulation factor IX OS=Homo sapiens GN=F9 PE=1 SV=2 - [FA9_HUMAN]	13.02	25	5	5	461	51.7	5.47
Q9UGM5	Fetuin-B OS=Homo sapiens GN=FETUB PE=1 SV=2 - [FETUB_HUMAN]	17.80	7	5	5	382	42.0	6.83
P04278	Sex hormone-binding globulin OS=Homo sapiens GN=SHBG PE=1 SV=2 - [SHBG_HUMAN]	18.66	9	5	5	402	43.8	6.71
P02786	Transferrin receptor protein 1 OS=Homo sapiens GN=TFRC PE=1 SV=2 - [TFR1_HUMAN]	7.50	3	5	5	760	84.8	6.61
B2R888	Monocyte differentiation antigen CD14 OS=Homo sapiens PE=2 SV=1 - [B2R888_HUMAN]	20.00	4	5	5	375	40.0	6.23
P09172	Dopamine beta-hydroxylase OS=Homo sapiens GN=DBH PE=1 SV=3 - [DOPO_HUMAN]	8.91	2	5	5	617	69.0	6.42
P05160	Coagulation factor XIII B chain OS=Homo sapiens GN=F13B PE=1 SV=3 - [F13B_HUMAN]	7.87	1	5	5	661	75.5	6.39
P15144	Aminopeptidase N OS=Homo sapiens GN=ANPEP PE=1 SV=4 - [AMPN_HUMAN]	4.65	6	5	5	967	109.5	5.48
B4DV20	cDNA FLJ54076, highly similar to Complement C2 (EC 3.4.21.43) OS=Homo sapiens PE=2 SV=1 - [B4DV20_HUMAN]	24.52	19	4	13	620	69.3	7.34
P48740	Mannan-binding lectin serine protease 1 OS=Homo sapiens GN=MASP1 PE=1 SV=3 - [MASP1_HUMAN]	16.31	5	4	10	699	79.2	5.49
Q9NZP8	Complement C1r subcomponent-like protein OS=Homo sapiens GN=C1RL PE=1 SV=2 - [C1RL_HUMAN]	12.53	4	4	6	487	53.5	7.20
P26927	Hepatocyte growth factor-like protein OS=Homo sapiens GN=MST1 PE=1 SV=2 - [HGFL_HUMAN]	8.86	6	4	5	711	80.3	7.68
P02655	Apolipoprotein C-II OS=Homo sapiens GN=APOC2 PE=1 SV=1 - [APOC2_HUMAN]	49.50	2	4	4	101	11.3	4.72
P02656	Apolipoprotein C-III OS=Homo sapiens GN=APOC3 PE=1 SV=1 - [APOC3_HUMAN]	53.54	2	4	4	99	10.8	5.41
P01625	Ig kappa chain V-IV region Len OS=Homo sapiens PE=1 SV=2 - [KV402_HUMAN]	42.98	6	4	4	114	12.6	7.93
P21817	Ryanodine receptor 1 OS=Homo sapiens GN=RYR1 PE=1 SV=3 - [RYR1_HUMAN]	1.55	3	4	4	5,038	564.8	5.30
Q5SRP4	Apolipoprotein M OS=Homo sapiens GN=APOM PE=4 SV=1 - [Q5SRP4_HUMAN]	31.03	3	4	4	116	13.0	7.75
B4DQA0	cDNA FLJ57560, highly similar to L-selectin OS=Homo sapiens PE=2 SV=1 - [B4DQA0_HUMAN]	13.54	5	4	4	325	36.3	6.23
D6REX5	Uncharacterized protein OS=Homo sapiens GN=SEPP1 PE=4 SV=1 - [D6REX5_HUMAN]	16.45	3	4	4	310	35.1	7.66
B4DPC7	cDNA FLJ55124, highly similar to Plasma serine protease inhibitor OS=Homo sapiens PE=2 SV=1 - [B4DPC7_HUMAN]	11.59	12	4	4	328	37.2	8.98
Q8NBH6	cDNA PSEC0266 fis, clone NT2RP3003649, highly similar to Homo sapiens fibulin-1D mRNA OS=Homo sapiens PE=2 SV=1 - [Q8NBH6_HUMAN]	7.68	8	4	4	638	70.5	5.91
P02747	Complement C1q subcomponent subunit C OS=Homo sapiens GN=C1QC PE=1 SV=3 - [C1QC_HUMAN]	20.00	1	4	4	245	25.8	8.41
D3DP13	Fibrinogen beta chain, isoform CRA_e OS=Homo sapiens GN=FGB PE=4 SV=1 - [D3DP13_HUMAN]	13.08	5	4	4	344	39.7	7.30
D6RA08	Uncharacterized protein OS=Homo sapiens GN=C1QB PE=4 SV=1 - [D6RA08_HUMAN]	21.83	5	4	4	229	24.1	9.16
E9PGL6	Uncharacterized protein OS=Homo sapiens GN=EFEMP1 PE=4 SV=1 - [E9PGL6_HUMAN]	11.68	14	4	4	411	46.4	4.92
Q59FC6	Tumor rejection antigen (Gp96) 1 variant (Fragment) OS=Homo sapiens PE=2 SV=1 - [Q59FC6_HUMAN]	10.07	7	4	4	576	65.9	5.19
B9ZVW9	Transforming growth factor, beta-induced, 68kDa, isoform CRA_a OS=Homo sapiens GN=TGFBI PE=4 SV=1 - [B9ZVW9_HUMAN]	12.26	5	4	4	416	45.4	6.70
Q149P0	GBF1 protein OS=Homo sapiens GN=GBF1 PE=2 SV=1 - [Q149P0_HUMAN]	1.35	39	4	4	1,855	205.9	5.76
B9EJA8	Mannose receptor, C type 1-like 1 OS=Homo sapiens GN=MRC1L1 PE=2 SV=1 - [B9EJA8_HUMAN]	3.57	5	4	4	1,456	165.9	6.51
E9PJL5	Uncharacterized protein OS=Homo sapiens GN=C12orf63 PE=4 SV=1 - [E9PJL5_HUMAN]	2.36	2	4	4	3,096	351.7	8.10
P13645	Keratin, type I cytoskeletal 10 OS=Homo sapiens GN=KRT10 PE=1 SV=6 - [K1C10_HUMAN]	7.88	1	4	4	584	58.8	5.21
Q6EMK4	Vasorin OS=Homo sapiens GN=VASN PE=1 SV=1 - [VASN_HUMAN]	8.92	1	4	4	673	71.7	7.39
B4DU18	cDNA FLJ51093, highly similar to Cadherin-5 OS=Homo sapiens PE=2 SV=1 - [B4DU18_HUMAN]	5.87	8	4	4	750	83.9	5.50
P01871	Ig mu chain C region OS=Homo sapiens GN=IGHM PE=1 SV=3 - [IGHM_HUMAN]	57.30	1	3	22	452	49.3	6.77
P02042	Hemoglobin subunit delta OS=Homo sapiens GN=HBD PE=1 SV=2 - [HBD_HUMAN]	62.59	21	3	8	147	16.0	8.05
P01860	Ig gamma-3 chain C region OS=Homo sapiens GN=IGHG3 PE=1 SV=2 - [IGHG3_HUMAN]	31.30	5	3	8	377	41.3	7.90
P36980	Complement factor H-related protein 2 OS=Homo sapiens GN=CFHR2 PE=1 SV=1 - [FHR2_HUMAN]	27.04	2	3	7	270	30.6	6.38
Q4TZM4	Hemoglobin beta chain (Fragment) OS=Homo sapiens GN=HBB PE=2 SV=1 - [Q4TZM4_HUMAN]	88.12	32	3	7	101	11.0	6.52
Q5CZ94	Putative uncharacterized protein DKFZp781M0386 OS=Homo sapiens GN=DKFZp781M0386 PE=2 SV=1 - [Q5CZ94_HUMAN]	40.60	5	3	7	234	25.0	7.27
P04206	Ig kappa chain V-III region GOL OS=Homo sapiens PE=1 SV=1 - [KV307_HUMAN]	52.29	3	3	5	109	11.8	9.25
Q7RTM4	Spectrin-like protein of the nuclear envelope and Golgi OS=Homo sapiens GN=SYNE1 PE=2 SV=1 - [Q7RTM4_HUMAN]	0.70	16	3	4	8,407	965.7	5.55
E5FY30	Glycoprotein Ib (Fragment) OS=Homo sapiens PE=4 SV=1 - [E5FY30_HUMAN]	25.99	9	3	4	177	19.7	5.85
Q8WXI7	Mucin-16 OS=Homo sapiens GN=MUC16 PE=1 SV=2 - [MUC16_HUMAN]	0.30	2	3	4	22,152	2,351.2	6.00
E9PCB7	Uncharacterized protein OS=Homo sapiens GN=RIMS1 PE=4 SV=1 - [E9PCB7_HUMAN]	6.97	26	3	4	760	85.1	9.42
Q7Z485	ATPase, Class I, type 8B, member 3, isoform CRA_b OS=Homo sapiens GN=ATP8B3 PE=2 SV=1 - [Q7Z485_HUMAN]	4.28	8	3	4	1,263	143.6	7.83
P02654	Apolipoprotein C-I OS=Homo sapiens GN=APOC1 PE=1 SV=1 - [APOC1_HUMAN]	24.10	1	3	3	83	9.3	8.47
B3KPF0	cDNA FLJ31712 fis, clone NT2RI2006445, highly similar to Insulin-like growth factor-binding protein 3 OS=Homo sapiens PE=4 SV=1 - [B3KPF0_HUMAN]	37.33	18	3	3	75	8.7	9.58
Q9UL88	Myosin-reactive immunoglobulin heavy chain variable region (Fragment) OS=Homo sapiens PE=2 SV=1 - [Q9UL88_HUMAN]	29.77	9	3	3	131	14.1	9.63
A0N5G1	Rheumatoid factor C6 light chain (Fragment) OS=Homo sapiens GN=V<kappa>1 PE=2 SV=1 - [A0N5G1_HUMAN]	40.52	4	3	3	116	12.5	8.46
Q13790	Apolipoprotein F OS=Homo sapiens GN=APOF PE=1 SV=2 - [APOF_HUMAN]	11.66	2	3	3	326	35.4	5.64
P01597	Ig kappa chain V-I region DEE OS=Homo sapiens PE=1 SV=1 - [KV105_HUMAN]	65.74	1	3	3	108	11.7	9.36
Q8TDY2	RB1-inducible coiled-coil protein 1 OS=Homo sapiens GN=RB1CC1 PE=1 SV=3 - [RBCC1_HUMAN]	1.63	5	3	3	1,594	183.0	5.41
Q96JG9	Zinc finger protein 469 OS=Homo sapiens GN=ZNF469 PE=1 SV=3 - [ZN469_HUMAN]	0.92	1	3	3	3,925	409.9	7.72
D3DQX7	Amyloid protein A OS=Homo sapiens GN=SAA1 PE=3 SV=1 - [D3DQX7_HUMAN]	35.25	5	3	3	122	13.6	6.79
A8K3E4	cDNA FLJ78367, highly similar to Homo sapiens fibrinogen, A alpha polypeptide (FGA), transcriptvariant alpha, mRNA OS=Homo sapiens PE=2 SV=1 - [A8K3E4_HUMAN]	6.52	4	3	3	644	69.7	8.06
B4DMZ5	cDNA FLJ57826, moderately similar to Cholesteryl ester transfer protein OS=Homo sapiens PE=2 SV=1 - [B4DMZ5_HUMAN]	9.01	4	3	3	433	47.8	6.01
Q9Y5Y7	Lymphatic vessel endothelial hyaluronic acid receptor 1 OS=Homo sapiens GN=LYVE1 PE=1 SV=2 - [LYVE1_HUMAN]	8.07	1	3	3	322	35.2	8.28
P58107	Epiplakin OS=Homo sapiens GN=EPPK1 PE=1 SV=2 - [EPIPL_HUMAN]	0.79	2	3	3	5,090	555.3	5.60
Q9NZJ4	Sacsin OS=Homo sapiens GN=SACS PE=1 SV=2 - [SACS_HUMAN]	0.59	1	3	3	4,579	520.8	7.05
Q01118	Sodium channel protein type 7 subunit alpha OS=Homo sapiens GN=SCN7A PE=2 SV=2 - [SCN7A_HUMAN]	1.72	5	3	3	1,682	193.4	7.96
Q9C0G0	Zinc finger protein 407 OS=Homo sapiens GN=ZNF407 PE=2 SV=2 - [ZN407_HUMAN]	2.09	1	3	3	2,248	247.2	6.49
B4DVE1	cDNA FLJ53478, highly similar to Galectin-3-binding protein OS=Homo sapiens PE=2 SV=1 - [B4DVE1_HUMAN]	8.38	8	3	3	573	64.1	5.47
Q59HC6	DNA cytosine methyltransferase 3 alpha isoform a variant (Fragment) OS=Homo sapiens PE=2 SV=1 - [Q59HC6_HUMAN]	3.21	4	3	3	811	89.6	6.04
Q71S06	Beta spectrin IV OS=Homo sapiens PE=2 SV=1 - [Q71S06_HUMAN]	2.60	8	3	3	2,002	225.4	5.67
B3KX75	cDNA FLJ44930 fis, clone BRAMY3015549, highly similar to Neural cell adhesion molecule L1-like protein (Fragment) OS=Homo sapiens PE=2 SV=1 - [B3KX75_HUMAN]	3.50	2	3	3	1,113	124.7	6.16
A8K335	cDNA FLJ76254, highly similar to Homo sapiens gamma-glutamyl hydrolase (GGH), mRNA OS=Homo sapiens PE=2 SV=1 - [A8K335_HUMAN]	7.86	4	3	3	318	36.0	7.42
E9PGS4	Uncharacterized protein OS=Homo sapiens GN=PNPLA5 PE=4 SV=1 - [E9PGS4_HUMAN]	12.46	5	3	3	337	37.7	7.90
B2R773	cDNA, FLJ93312, highly similar to Homo sapiens adipose most abundant gene transcript 1 (APM1), mRNA OS=Homo sapiens PE=2 SV=1 - [B2R773_HUMAN]	15.98	2	3	3	244	26.4	5.74
B4E3I4	cDNA FLJ53432, highly similar to DNA mismatch repair protein MSH6 OS=Homo sapiens PE=2 SV=1 - [B4E3I4_HUMAN]	3.78	5	3	3	1,058	119.7	6.84
E9PDD2	Uncharacterized protein OS=Homo sapiens GN=VCAM1 PE=4 SV=1 - [E9PDD2_HUMAN]	6.48	5	3	3	540	59.2	5.63
B2R701	cDNA, FLJ93202, Homo sapiens protease inhibitor 16 (PI16), mRNA OS=Homo sapiens PE=2 SV=1 - [B2R701_HUMAN]	6.78	2	3	3	428	45.7	5.38
B3KXZ9	cDNA FLJ46477 fis, clone THYMU3025118, highly similar to Cell surface glycoprotein MUC18 OS=Homo sapiens PE=2 SV=1 - [B3KXZ9_HUMAN]	9.60	4	3	3	531	59.0	5.19
P35527	Keratin, type I cytoskeletal 9 OS=Homo sapiens GN=KRT9 PE=1 SV=3 - [K1C9_HUMAN]	10.11	1	3	3	623	62.0	5.24
B3KUS4	cDNA FLJ40508 fis, clone TESTI2045850, highly similar to AP-3 complex subunit beta-2 OS=Homo sapiens PE=2 SV=1 - [B3KUS4_HUMAN]	5.71	6	3	3	718	78.8	5.34
Q07954	Prolow-density lipoprotein receptor-related protein 1 OS=Homo sapiens GN=LRP1 PE=1 SV=2 - [LRP1_HUMAN]	0.86	3	3	3	4,544	504.3	5.39
Q5T011	Protein SZT2 OS=Homo sapiens GN=SZT2 PE=2 SV=3 - [SZT2_HUMAN]	1.05	1	3	3	3,432	377.8	6.27
P08603	Complement factor H OS=Homo sapiens GN=CFH PE=1 SV=4 - [CFAH_HUMAN]	50.77	8	2	53	1,231	139.0	6.61
B4DI57	cDNA FLJ54111, highly similar to Serotransferrin OS=Homo sapiens PE=2 SV=1 - [B4DI57_HUMAN]	64.27	3	2	34	571	63.4	7.21
B2RMS9	Inter-alpha (Globulin) inhibitor H4 (Plasma Kallikrein-sensitive glycoprotein) OS=Homo sapiens GN=ITIH4 PE=2 SV=1 - [B2RMS9_HUMAN]	46.67	6	2	31	930	103.3	6.98
B4DPP8	cDNA FLJ53075, highly similar to Kininogen-1 OS=Homo sapiens PE=2 SV=1 - [B4DPP8_HUMAN]	50.12	5	2	20	415	46.5	6.43
Q6N092	Putative uncharacterized protein DKFZp686K18196 (Fragment) OS=Homo sapiens GN=DKFZp686K18196 PE=1 SV=1 - [Q6N092_HUMAN]	42.39	3	2	17	519	56.4	6.93
Q9NPP6	Immunoglobulin heavy chain variant (Fragment) OS=Homo sapiens PE=2 SV=1 - [Q9NPP6_HUMAN]	58.41	5	2	17	416	44.8	6.13
B7Z1F8	cDNA FLJ53025, highly similar to Complement C4-B OS=Homo sapiens PE=2 SV=1 - [B7Z1F8_HUMAN]	57.41	2	2	15	270	30.4	7.99
Q6GMX6	IGH@ protein OS=Homo sapiens GN=IGH@ PE=1 SV=1 - [Q6GMX6_HUMAN]	43.23	21	2	15	465	51.1	8.69
Q6MZQ6	Putative uncharacterized protein DKFZp686G11190 OS=Homo sapiens GN=DKFZp686G11190 PE=1 SV=1 - [Q6MZQ6_HUMAN]	39.58	10	2	13	475	52.0	8.06
B1AKG0	Complement factor H-related 1 OS=Homo sapiens GN=CFHR1 PE=4 SV=1 - [B1AKG0_HUMAN]	36.16	7	2	10	271	30.8	7.81
Q9UK54	Hemoglobin beta subunit variant (Fragment) OS=Homo sapiens GN=HBB PE=2 SV=1 - [Q9UK54_HUMAN]	81.25	20	2	9	128	14.0	6.95
Q6MZX7	Putative uncharacterized protein DKFZp686M24218 OS=Homo sapiens GN=DKFZp686M24218 PE=1 SV=1 - [Q6MZX7_HUMAN]	20.38	1	2	8	476	52.4	7.77
Q6N093	Putative uncharacterized protein DKFZp686I04196 (Fragment) OS=Homo sapiens GN=DKFZp686I04196 PE=1 SV=1 - [Q6N093_HUMAN]	23.26	4	2	8	417	46.0	7.59
Q2L9S7	Alpha-1-antitrypsin MBrescia variant (Fragment) OS=Homo sapiens GN=AAT PE=2 SV=1 - [Q2L9S7_HUMAN]	73.03	1	2	8	89	10.7	6.16
A2KBC6	Anti-FactorVIII scFv (Fragment) OS=Homo sapiens PE=2 SV=1 - [A2KBC6_HUMAN]	28.99	37	2	6	238	25.0	8.41
A0M8Q6	Ig lambda-7 chain C region OS=Homo sapiens GN=IGLC7 PE=1 SV=2 - [LAC7_HUMAN]	57.55	1	2	5	106	11.3	8.28
P35908	Keratin, type II cytoskeletal 2 epidermal OS=Homo sapiens GN=KRT2 PE=1 SV=2 - [K22E_HUMAN]	6.89	21	2	4	639	65.4	8.00
P80748	Ig lambda chain V-III region LOI OS=Homo sapiens PE=1 SV=1 - [LV302_HUMAN]	45.95	3	2	4	111	11.9	5.08
P20848	Putative alpha-1-antitrypsin-related protein OS=Homo sapiens GN=SERPINA2 PE=5 SV=1 - [A1ATR_HUMAN]	4.76	1	2	3	420	47.9	7.90
P01621	Ig kappa chain V-III region NG9 (Fragment) OS=Homo sapiens PE=1 SV=1 - [KV303_HUMAN]	32.00	2	2	3	100	10.7	6.52
E7EU22	Uncharacterized protein OS=Homo sapiens GN=CEP350 PE=4 SV=1 - [E7EU22_HUMAN]	1.12	7	2	3	3,117	350.8	6.33
B4DPR0	cDNA FLJ60136, moderately similar to Complement factor H-related protein 3 OS=Homo sapiens PE=2 SV=1 - [B4DPR0_HUMAN]	12.64	10	2	3	269	30.7	7.78
Q0ZCH9	Immunglobulin heavy chain variable region (Fragment) OS=Homo sapiens PE=4 SV=1 - [Q0ZCH9_HUMAN]	32.23	33	2	3	121	13.4	8.46
A0N5G5	Rheumatoid factor D5 light chain (Fragment) OS=Homo sapiens GN=V<kappa>3 PE=2 SV=1 - [A0N5G5_HUMAN]	28.81	3	2	3	118	12.8	8.97
Q8WYP5	Protein ELYS OS=Homo sapiens GN=AHCTF1 PE=1 SV=3 - [ELYS_HUMAN]	1.99	4	2	3	2,266	252.3	6.60
D7UT47	SH3 and multiple ankyrin repeat domains 3 OS=Homo sapiens GN=SHANK3 PE=2 SV=1 - [D7UT47_HUMAN]	1.56	2	2	3	1,731	184.6	8.88
B4E2L8	cDNA FLJ53487, highly similar to Coagulation factor XIII A chain (EC 2.3.2.13) OS=Homo sapiens PE=2 SV=1 - [B4E2L8_HUMAN]	7.03	5	2	3	626	70.3	5.34
Q9ULE0	Protein WWC3 OS=Homo sapiens GN=WWC3 PE=2 SV=3 - [WWC3_HUMAN]	2.38	15	2	3	1,092	122.6	6.37
Q9H319	Mutant desmin OS=Homo sapiens PE=2 SV=1 - [Q9H319_HUMAN]	6.81	21	2	3	470	53.5	5.24
B3KNM3	cDNA FLJ14951 fis, clone PLACE3000020, highly similar to Adenylate cyclase type 3 (EC 4.6.1.1) OS=Homo sapiens PE=2 SV=1 - [B3KNM3_HUMAN]	4.95	9	2	3	1,090	123.0	6.55
C9J3J9	Uncharacterized protein OS=Homo sapiens GN=AMOTL1 PE=4 SV=1 - [C9J3J9_HUMAN]	2.51	17	2	3	957	106.6	7.25
Q9NRY4	Glucocorticoid receptor DNA-binding factor 1 OS=Homo sapiens GN=GRLF1 PE=1 SV=2 - [GRLF1_HUMAN]	3.44	2	2	3	1,513	172.1	6.79
P81605	Dermcidin OS=Homo sapiens GN=DCD PE=1 SV=2 - [DCD_HUMAN]	20.00	2	2	2	110	11.3	6.54
Q7L5Y6	DET1 homolog OS=Homo sapiens GN=DET1 PE=1 SV=2 - [DET1_HUMAN]	5.82	2	2	2	550	63.8	7.56
Q9NYQ8	Protocadherin Fat 2 OS=Homo sapiens GN=FAT2 PE=1 SV=2 - [FAT2_HUMAN]	0.62	4	2	2	4,349	479.0	5.16
Q6ZU52	Uncharacterized protein KIAA0408 OS=Homo sapiens GN=KIAA0408 PE=1 SV=1 - [K0408_HUMAN]	3.17	1	2	2	694	79.1	8.35
P02533	Keratin, type I cytoskeletal 14 OS=Homo sapiens GN=KRT14 PE=1 SV=4 - [K1C14_HUMAN]	4.24	1	2	2	472	51.5	5.16
Q09028	Histone-binding protein RBBP4 OS=Homo sapiens GN=RBBP4 PE=1 SV=3 - [RBBP4_HUMAN]	4.47	12	2	2	425	47.6	4.89
O60293	Zinc finger C3H1 domain-containing protein OS=Homo sapiens GN=ZFC3H1 PE=1 SV=3 - [ZC3H1_HUMAN]	2.66	1	2	2	1,989	226.2	8.13
B2RMV5	ADAM metallopeptidase domain 28 OS=Homo sapiens GN=ADAM28 PE=2 SV=1 - [B2RMV5_HUMAN]	3.74	3	2	2	775	87.1	6.95
A6XMH1	Transthyretin OS=Homo sapiens PE=2 SV=1 - [A6XMH1_HUMAN]	25.36	6	2	2	138	14.9	5.76
B4DPC8	cDNA FLJ51023, highly similar to Vitamin K-dependent protein C (EC 3.4.21.69) OS=Homo sapiens PE=2 SV=1 - [B4DPC8_HUMAN]	7.35	14	2	2	272	30.8	6.60
Q9NTG0	Putative uncharacterized protein DKFZp434G2016 (Fragment) OS=Homo sapiens GN=DKFZp434G2016 PE=2 SV=1 - [Q9NTG0_HUMAN]	5.24	5	2	2	496	57.7	7.65
B2R5G8	Amyloid protein A OS=Homo sapiens PE=2 SV=1 - [B2R5G8_HUMAN]	14.62	2	2	2	130	14.8	8.98
Q5NV83	V3-3 protein (Fragment) OS=Homo sapiens GN=IGLV7-46 PE=2 SV=1 - [Q5NV83_HUMAN]	18.37	4	2	2	98	10.4	7.28
B4DXN1	cDNA FLJ53236, highly similar to Homo sapiens IQ motif containing E (IQCE), mRNA OS=Homo sapiens PE=2 SV=1 - [B4DXN1_HUMAN]	2.20	5	2	2	637	71.1	9.38
P08519	Apolipoprotein(a) OS=Homo sapiens GN=LPA PE=1 SV=1 - [APOA_HUMAN]	7.12	2	2	2	4,548	501.0	5.88
Q9Y5P4	Collagen type IV alpha-3-binding protein OS=Homo sapiens GN=COL4A3BP PE=1 SV=1 - [C43BP_HUMAN]	2.56	1	2	2	624	70.8	5.48
P16383	GC-rich sequence DNA-binding factor OS=Homo sapiens GN=TCF9 PE=1 SV=2 - [GCF_HUMAN]	4.74	5	2	2	781	89.3	5.99
P06213	Insulin receptor OS=Homo sapiens GN=INSR PE=1 SV=4 - [INSR_HUMAN]	1.01	3	2	2	1,382	156.2	6.20
Q86VQ0	Lebercilin OS=Homo sapiens GN=LCA5 PE=1 SV=2 - [LCA5_HUMAN]	2.01	10	2	2	697	80.5	7.68
P11226	Mannose-binding protein C OS=Homo sapiens GN=MBL2 PE=1 SV=2 - [MBL2_HUMAN]	8.06	1	2	2	248	26.1	5.49
Q12769	Nuclear pore complex protein Nup160 OS=Homo sapiens GN=NUP160 PE=1 SV=3 - [NU160_HUMAN]	1.60	3	2	2	1,436	162.0	5.50
Q13258	Prostaglandin D2 receptor OS=Homo sapiens GN=PTGDR PE=2 SV=2 - [PD2R_HUMAN]	4.18	1	2	2	359	40.2	9.16
Q04912	Macrophage-stimulating protein receptor OS=Homo sapiens GN=MST1R PE=1 SV=2 - [RON_HUMAN]	1.14	11	2	2	1,400	152.2	6.55
Q9NQW1	Protein transport protein Sec31B OS=Homo sapiens GN=SEC31B PE=1 SV=1 - [SC31B_HUMAN]	2.46	6	2	2	1,179	128.6	8.29
Q92623	Tetratricopeptide repeat protein 9A OS=Homo sapiens GN=TTC9 PE=2 SV=3 - [TTC9A_HUMAN]	5.41	9	2	2	222	24.4	8.98
Q8IYT8	Serine/threonine-protein kinase ULK2 OS=Homo sapiens GN=ULK2 PE=1 SV=3 - [ULK2_HUMAN]	2.41	2	2	2	1,036	112.6	8.53
D6RA70	Uncharacterized protein OS=Homo sapiens GN=YTHDC2 PE=4 SV=1 - [D6RA70_HUMAN]	1.89	12	2	2	739	82.9	7.39
E9PKE8	Uncharacterized protein OS=Homo sapiens GN=CNTN5 PE=4 SV=1 - [E9PKE8_HUMAN]	3.07	4	2	2	911	100.3	6.68
Q5KTC1	Transient receptor potential cation channel, subfamily M, member 2, striatum short form (SSF-TRPM2) OS=Homo sapiens GN=TRPM2 PE=2 SV=1 - [Q5KTC1_HUMAN]	1.40	6	2	2	1,289	147.2	7.09
B3KSN9	cDNA FLJ36718 fis, clone UTERU2010747, highly similar to Heparanase-2 (EC 3.2.-.-) OS=Homo sapiens PE=2 SV=1 - [B3KSN9_HUMAN]	6.37	4	2	2	534	60.1	9.99
B4DN75	cDNA FLJ60724, highly similar to Cartilage oligomeric matrix protein OS=Homo sapiens PE=2 SV=1 - [B4DN75_HUMAN]	3.95	9	2	2	684	75.1	4.63
B7ZKL5	AXIN2 protein OS=Homo sapiens GN=AXIN2 PE=2 SV=1 - [B7ZKL5_HUMAN]	3.60	3	2	2	778	86.6	7.58
B7Z514	cDNA FLJ50601, highly similar to Glutathione synthetase (EC 6.3.2.3) OS=Homo sapiens PE=2 SV=1 - [B7Z514_HUMAN]	9.25	4	2	2	346	38.2	7.17
Q59H62	IPO4 protein variant (Fragment) OS=Homo sapiens PE=2 SV=1 - [Q59H62_HUMAN]	4.88	12	2	2	758	81.8	4.79
Q59FP8	Neogenin homolog 1 variant (Fragment) OS=Homo sapiens PE=2 SV=1 - [Q59FP8_HUMAN]	2.92	5	2	2	1,130	123.9	7.03
A5YKK6	CCR4-NOT transcription complex subunit 1 OS=Homo sapiens GN=CNOT1 PE=1 SV=2 - [CNOT1_HUMAN]	0.59	3	2	2	2,376	266.8	7.11
Q6ZUT9	DENN domain-containing protein 5B OS=Homo sapiens GN=DENND5B PE=1 SV=2 - [DEN5B_HUMAN]	1.96	2	2	2	1,274	144.9	6.73
Q9Y2I6	Ninein-like protein OS=Homo sapiens GN=NINL PE=1 SV=2 - [NINL_HUMAN]	2.10	2	2	2	1,382	156.2	5.06
Q9H7B4	SET and MYND domain-containing protein 3 OS=Homo sapiens GN=SMYD3 PE=1 SV=4 - [SMYD3_HUMAN]	7.94	6	2	2	428	49.1	7.25
Q76NI1	Protein very KIND OS=Homo sapiens GN=KNDC1 PE=2 SV=2 - [VKIND_HUMAN]	2.23	5	2	2	1,749	191.3	6.16
D3DN94	Poly (ADP-ribose) polymerase family, member 14, isoform CRA_a OS=Homo sapiens GN=PARP14 PE=4 SV=1 - [D3DN94_HUMAN]	2.46	2	2	2	1,424	160.1	8.35
B3KRS5	Histone deacetylase OS=Homo sapiens GN=HDAC2 PE=2 SV=1 - [B3KRS5_HUMAN]	8.95	8	2	2	458	52.0	5.74
Q2M2Y9	Uncharacterized protein C3orf48. (Fragment) OS=Homo sapiens GN=C3orf48 PE=2 SV=1 - [Q2M2Y9_HUMAN]	6.23	2	2	2	369	42.7	9.31
E9PPV2	Uncharacterized protein OS=Homo sapiens GN=RNPC3 PE=4 SV=1 - [E9PPV2_HUMAN]	23.91	1	2	2	138	15.8	6.54
B4DFJ1	cDNA FLJ53728, highly similar to TERF1-interacting nuclear factor 2 OS=Homo sapiens PE=2 SV=1 - [B4DFJ1_HUMAN]	6.25	4	2	2	416	46.1	7.78
D6RBJ7	Uncharacterized protein OS=Homo sapiens GN=GC PE=4 SV=1 - [D6RBJ7_HUMAN]	9.48	4	2	2	348	38.8	5.50
C9JVG2	Uncharacterized protein OS=Homo sapiens GN=ZNF775 PE=4 SV=1 - [C9JVG2_HUMAN]	30.56	3	2	2	144	15.5	9.25
B9A6L3	TBC1 domain family, member 12 OS=Homo sapiens GN=TBC1D12 PE=2 SV=1 - [B9A6L3_HUMAN]	4.55	2	2	2	769	84.9	6.16
Q59G05	Poly(A) polymerase gamma variant (Fragment) OS=Homo sapiens PE=2 SV=1 - [Q59G05_HUMAN]	6.81	8	2	2	382	42.3	9.32
B3KSE0	cDNA FLJ36069 fis, clone TESTI2019406, highly similar to HEME OXYGENASE 2 (EC 1.14.99.3) OS=Homo sapiens PE=2 SV=1 - [B3KSE0_HUMAN]	7.28	5	2	2	316	35.9	5.50
Q496Y0	LON peptidase N-terminal domain and RING finger protein 3 OS=Homo sapiens GN=LONRF3 PE=1 SV=1 - [LONF3_HUMAN]	3.56	3	2	2	759	84.4	7.25
Q13395	Probable methyltransferase TARBP1 OS=Homo sapiens GN=TARBP1 PE=1 SV=1 - [TARB1_HUMAN]	0.93	1	2	2	1,621	181.6	7.05
Q9NQZ8	Endothelial zinc finger protein induced by tumor necrosis factor alpha OS=Homo sapiens GN=ZNF71 PE=2 SV=1 - [ZNF71_HUMAN]	5.11	1	2	2	489	54.5	8.68
B3KU01	cDNA FLJ39018 fis, clone NT2RP7002594, highly similar to Semaphorin-6A OS=Homo sapiens PE=2 SV=1 - [B3KU01_HUMAN]	8.53	11	2	2	457	49.7	9.38
E7EPC0	Uncharacterized protein OS=Homo sapiens GN=RYR2 PE=4 SV=1 - [E7EPC0_HUMAN]	0.83	3	2	2	4,959	563.7	6.28
Q8TA90	Similar to Elongation factor 2b (Fragment) OS=Homo sapiens PE=2 SV=1 - [Q8TA90_HUMAN]	2.51	6	2	2	517	57.5	6.93
Q6ZMN5	cDNA FLJ16798 fis, clone TESTI2053667, weakly similar to PAB-dependent poly(A)-specific ribonuclease subunit PAN3 (EC 3.1.13.4) OS=Homo sapiens PE=2 SV=1 - [Q6ZMN5_HUMAN]	4.24	3	2	2	472	53.6	7.55
B9ZVS0	Uncharacterized protein OS=Homo sapiens GN=CEP152 PE=4 SV=2 - [B9ZVS0_HUMAN]	1.34	5	2	2	1,271	146.8	5.40
Q6IEH8	Transcriptional regulator OS=Homo sapiens GN=NIPBL PE=2 SV=1 - [Q6IEH8_HUMAN]	0.57	2	2	2	2,804	315.9	7.91
E9PN18	Uncharacterized protein OS=Homo sapiens GN=PUF60 PE=4 SV=1 - [E9PN18_HUMAN]	9.89	5	2	2	263	28.7	7.85
Q8WXX0	Dynein heavy chain 7, axonemal OS=Homo sapiens GN=DNAH7 PE=1 SV=2 - [DYH7_HUMAN]	0.47	1	2	2	4,024	460.9	6.00
Q15262	Receptor-type tyrosine-protein phosphatase kappa OS=Homo sapiens GN=PTPRK PE=1 SV=2 - [PTPRK_HUMAN]	3.47	10	2	2	1,439	162.0	5.90
Q6ZRS2	Helicase SRCAP OS=Homo sapiens GN=SRCAP PE=1 SV=3 - [SRCAP_HUMAN]	0.74	1	2	2	3,230	343.3	5.96
Q8N6N2	Tetratricopeptide repeat protein 9B OS=Homo sapiens GN=TTC9B PE=2 SV=1 - [TTC9B_HUMAN]	6.28	3	2	2	239	25.9	9.48
A8K337	cDNA FLJ77862, highly similar to Homo sapiens catechol-O-methyltransferase domain containing 1 (COMTD1), mRNA OS=Homo sapiens PE=2 SV=1 - [A8K337_HUMAN]	19.85	2	2	2	262	28.8	8.38
A5YAK2	Apolipoprotein C-IV OS=Homo sapiens GN=APOC4 PE=2 SV=1 - [A5YAK2_HUMAN]	12.60	2	2	2	127	14.6	9.13
B7ZLE7	DAPK1 protein OS=Homo sapiens GN=DAPK1 PE=2 SV=1 - [B7ZLE7_HUMAN]	1.47	7	2	2	1,364	152.4	7.17
Q8WY19	Down syndrome cell adhesion molecule splice variant (Fragment) OS=Homo sapiens GN=DSCAM PE=2 SV=1 - [Q8WY19_HUMAN]	1.72	3	2	2	1,746	192.4	7.77
Q9UID3	Protein fat-free homolog OS=Homo sapiens GN=FFR PE=1 SV=2 - [FFR_HUMAN]	2.30	5	2	2	782	86.0	6.47
Q9NS87	Kinesin-like protein KIF15 OS=Homo sapiens GN=KIF15 PE=1 SV=1 - [KIF15_HUMAN]	1.80	1	2	2	1,388	160.1	6.00
Q8IYB3	Serine/arginine repetitive matrix protein 1 OS=Homo sapiens GN=SRRM1 PE=1 SV=2 - [SRRM1_HUMAN]	2.99	5	2	2	904	102.3	11.84
Q13428	Treacle protein OS=Homo sapiens GN=TCOF1 PE=1 SV=3 - [TCOF_HUMAN]	2.49	5	2	2	1,488	152.0	9.04
D3DSU3	Kinesin family member 13B, isoform CRA_a OS=Homo sapiens GN=KIF13B PE=3 SV=1 - [D3DSU3_HUMAN]	1.60	4	2	2	1,562	174.7	5.91
B4DSA9	cDNA FLJ51068, highly similar to Synaptotagmin-4 OS=Homo sapiens PE=2 SV=1 - [B4DSA9_HUMAN]	13.97	6	2	2	179	20.1	8.94
B3KMT7	cDNA FLJ12561 fis, clone NT2RM4000798, highly similar to Brefeldin A-inhibited guanine nucleotide-exchange protein 2 OS=Homo sapiens PE=2 SV=1 - [B3KMT7_HUMAN]	4.00	4	2	2	750	86.5	6.43
A8K5S8	cDNA FLJ78047 OS=Homo sapiens PE=2 SV=1 - [A8K5S8_HUMAN]	3.14	11	2	2	700	77.1	7.87
C9JGV8	Uncharacterized protein OS=Homo sapiens GN=BDP1 PE=4 SV=1 - [C9JGV8_HUMAN]	3.93	4	2	2	865	97.4	7.56
E7EUY0	Uncharacterized protein OS=Homo sapiens GN=PRKDC PE=4 SV=1 - [E7EUY0_HUMAN]	0.90	3	2	2	4,096	465.1	7.17
A8K941	cDNA FLJ77618 OS=Homo sapiens PE=2 SV=1 - [A8K941_HUMAN]	4.66	2	2	2	708	79.4	7.83
B4DLW4	cDNA FLJ60300, highly similar to Homo sapiens protein phosphatase 1, regulatory (inhibitor) subunit 12C (PPP1R12C), mRNA OS=Homo sapiens PE=2 SV=1 - [B4DLW4_HUMAN]	3.72	4	2	2	538	59.3	6.07
A8K3L6	cDNA FLJ76119 OS=Homo sapiens PE=2 SV=1 - [A8K3L6_HUMAN]	3.00	5	2	2	567	64.2	6.07
B4DV74	cDNA FLJ53855, highly similar to Kanadaptin OS=Homo sapiens PE=2 SV=1 - [B4DV74_HUMAN]	3.06	6	2	2	490	55.7	4.93
A2IPI4	HRV Fab 025-VL (Fragment) OS=Homo sapiens PE=2 SV=1 - [A2IPI4_HUMAN]	23.01	4	2	2	113	12.1	9.20
O95996	Adenomatous polyposis coli protein 2 OS=Homo sapiens GN=APC2 PE=1 SV=1 - [APC2_HUMAN]	1.52	1	2	2	2,303	243.8	8.82
Q8WXK1	Ankyrin repeat and SOCS box protein 15 OS=Homo sapiens GN=ASB15 PE=2 SV=3 - [ASB15_HUMAN]	2.04	4	2	2	588	65.8	5.83
Q76LX8	A disintegrin and metalloproteinase with thrombospondin motifs 13 OS=Homo sapiens GN=ADAMTS13 PE=1 SV=1 - [ATS13_HUMAN]	1.75	6	2	2	1,427	153.5	7.17
Q9UPV0	Centrosomal protein of 164 kDa OS=Homo sapiens GN=CEP164 PE=1 SV=3 - [CE164_HUMAN]	2.12	8	2	2	1,460	164.2	5.36
Q14789	Golgin subfamily B member 1 OS=Homo sapiens GN=GOLGB1 PE=1 SV=2 - [GOGB1_HUMAN]	0.46	9	2	2	3,259	375.8	5.00
Q6P0N0	Mis18-binding protein 1 OS=Homo sapiens GN=MIS18BP1 PE=1 SV=1 - [M18BP_HUMAN]	1.94	1	2	2	1,132	129.0	9.25
Q96JQ0	Protocadherin-16 OS=Homo sapiens GN=DCHS1 PE=2 SV=1 - [PCD16_HUMAN]	0.85	3	2	2	3,298	346.0	4.94
P22891	Vitamin K-dependent protein Z OS=Homo sapiens GN=PROZ PE=1 SV=2 - [PROZ_HUMAN]	4.75	2	2	2	400	44.7	5.97
Q96AY4	Tetratricopeptide repeat protein 28 OS=Homo sapiens GN=TTC28 PE=1 SV=4 - [TTC28_HUMAN]	1.29	1	2	2	2,481	270.7	6.89
B1ANB7	Mucolipin 3 OS=Homo sapiens GN=MCOLN3 PE=4 SV=1 - [B1ANB7_HUMAN]	5.30	7	2	2	321	37.2	6.23
Q5T4F6	Cartilage acidic protein 1 (Fragment) OS=Homo sapiens GN=CRTAC1 PE=2 SV=1 - [Q5T4F6_HUMAN]	4.39	2	2	2	524	56.5	5.55
A1L3U4	PCDH24 protein OS=Homo sapiens GN=PCDH24 PE=2 SV=1 - [A1L3U4_HUMAN]	2.21	2	2	2	1,310	141.5	4.50
D3DWB6	Ubiquitin carboxyl-terminal hydrolase OS=Homo sapiens GN=USP9X PE=3 SV=1 - [D3DWB6_HUMAN]	1.05	6	2	2	2,379	271.1	5.97
E7ERU0	Uncharacterized protein OS=Homo sapiens GN=DST PE=4 SV=1 - [E7ERU0_HUMAN]	0.28	8	2	2	5,375	615.3	5.74
B4DK53	cDNA FLJ60618, weakly similar to Zinc finger protein 3 OS=Homo sapiens PE=2 SV=1 - [B4DK53_HUMAN]	4.77	4	2	2	461	53.4	8.92
Q9BR60	OS9 protein (Fragment) OS=Homo sapiens GN=OS9 PE=2 SV=2 - [Q9BR60_HUMAN]	6.97	15	2	2	373	42.5	4.73
C9K055	Uncharacterized protein OS=Homo sapiens GN=PCYOX1 PE=4 SV=1 - [C9K055_HUMAN]	9.09	9	2	2	209	23.4	6.68
D9ZGF8	Rho-associated, coiled-coil containing protein kinase 1 OS=Homo sapiens GN=ROCK1 PE=4 SV=1 - [D9ZGF8_HUMAN]	1.62	2	2	2	1,354	158.1	5.94
A6NP16	Uncharacterized protein OS=Homo sapiens GN=AMOT PE=4 SV=1 - [A6NP16_HUMAN]	5.70	6	2	2	597	68.4	8.06
Q9P225	Dynein heavy chain 2, axonemal OS=Homo sapiens GN=DNAH2 PE=1 SV=3 - [DYH2_HUMAN]	0.36	1	2	2	4,427	507.4	6.37
Q9P2X3	Protein IMPACT OS=Homo sapiens GN=IMPACT PE=1 SV=2 - [IMPCT_HUMAN]	8.75	1	2	2	320	36.5	4.97
O60333	Kinesin-like protein KIF1B OS=Homo sapiens GN=KIF1B PE=1 SV=5 - [KIF1B_HUMAN]	1.98	6	2	2	1,816	204.3	5.60
Q96L50	Leucine-rich repeat protein 1 OS=Homo sapiens GN=LRR1 PE=1 SV=2 - [LLR1_HUMAN]	2.90	5	2	2	414	46.7	9.09
P26599	Polypyrimidine tract-binding protein 1 OS=Homo sapiens GN=PTBP1 PE=1 SV=1 - [PTBP1_HUMAN]	4.90	12	2	2	531	57.2	9.17
D3DRJ1	HCG27250, isoform CRA_a OS=Homo sapiens GN=hCG_27250 PE=4 SV=1 - [D3DRJ1_HUMAN]	1.97	12	2	2	864	95.4	6.52
E7EV48	Uncharacterized protein OS=Homo sapiens GN=ITPR1 PE=4 SV=1 - [E7EV48_HUMAN]	0.95	9	2	2	2,732	310.8	6.23
B9EGR5	MGA protein OS=Homo sapiens GN=MGA PE=2 SV=1 - [B9EGR5_HUMAN]	0.98	3	2	2	2,856	314.9	6.81
E7ETY1	Uncharacterized protein OS=Homo sapiens GN=RBBP8 PE=4 SV=1 - [E7ETY1_HUMAN]	2.33	6	2	2	902	102.5	6.24
B7Z461	cDNA FLJ60122, highly similar to Serine/threonine-protein kinase PCTAIRE-1 (EC 2.7.11.22) OS=Homo sapiens PE=2 SV=1 - [B7Z461_HUMAN]	5.97	9	2	2	335	36.2	8.91
A5PLM6	SEC14L1 protein OS=Homo sapiens GN=SEC14L1 PE=2 SV=1 - [A5PLM6_HUMAN]	2.92	7	2	2	719	81.8	6.51
B7ZAU8	cDNA, FLJ79312, highly similar to HEAT repeat-containing protein 1 OS=Homo sapiens PE=2 SV=1 - [B7ZAU8_HUMAN]	2.93	5	2	2	1,126	127.5	6.52
B4DT84	cDNA FLJ60348, highly similar to Atrial natriuretic peptide clearance receptor OS=Homo sapiens PE=2 SV=1 - [B4DT84_HUMAN]	5.25	7	2	2	324	36.6	6.55
C9JEU5	Uncharacterized protein OS=Homo sapiens GN=FGG PE=4 SV=1 - [C9JEU5_HUMAN]	4.94	7	2	2	445	50.3	6.09
B4DG75	cDNA FLJ59875, highly similar to DNA polymerase mu (EC 2.7.7.7) OS=Homo sapiens PE=2 SV=1 - [B4DG75_HUMAN]	9.28	8	2	2	237	25.6	8.06
Q14689	Disco-interacting protein 2 homolog A OS=Homo sapiens GN=DIP2A PE=1 SV=2 - [DIP2A_HUMAN]	1.34	7	2	2	1,571	170.3	8.03
O43281	Embryonal Fyn-associated substrate OS=Homo sapiens GN=EFS PE=1 SV=1 - [EFS_HUMAN]	4.81	1	2	2	561	58.8	5.11
A9UHW6	MIF4G domain-containing protein OS=Homo sapiens GN=MIF4GD PE=1 SV=1 - [MI4GD_HUMAN]	6.76	5	2	2	222	25.4	5.33
Q96MW7	Tigger transposable element-derived protein 1 OS=Homo sapiens GN=TIGD1 PE=2 SV=1 - [TIGD1_HUMAN]	5.92	2	2	2	591	67.3	8.46
Q5T321	Neurobeachin OS=Homo sapiens GN=NBEA PE=2 SV=1 - [Q5T321_HUMAN]	1.22	9	2	2	2,943	327.3	6.16
B4DZR3	cDNA FLJ59826, highly similar to Zinc finger protein ZFPM2 OS=Homo sapiens PE=2 SV=1 - [B4DZR3_HUMAN]	2.27	6	2	2	882	98.0	7.62
Q99989	Putative uncharacterized protein CFTR (Fragment) OS=Homo sapiens GN=CFTR PE=2 SV=1 - [Q99989_HUMAN]	3.61	14	2	2	996	113.3	8.60
Q6ZR19	cDNA FLJ46721 fis, clone TRACH3018524, highly similar to Protein-tyrosine phosphatase beta (EC 3.1.3.48) (Fragment) OS=Homo sapiens PE=2 SV=1 - [Q6ZR19_HUMAN]	2.42	7	2	2	1,407	156.9	7.05
A7E1W7	SIGLEC-15 OS=Homo sapiens GN=SIGLEC15 PE=2 SV=1 - [A7E1W7_HUMAN]	7.93	3	2	2	328	35.6	8.51
B4DN96	cDNA FLJ54474, highly similar to Ubiquitin carboxyl-terminal hydrolase 40 (EC 3.1.2.15) (Fragment) OS=Homo sapiens PE=2 SV=1 - [B4DN96_HUMAN]	2.76	2	2	2	798	89.7	5.52
Q6ZP06	CDNA FLJ26768 fis, clone PRS02994 OS=Homo sapiens PE=2 SV=1 - [Q6ZP06_HUMAN]	28.20	1	2	2	539	59.0	8.76
Q59EZ2	Telomerase protein component 1 variant (Fragment) OS=Homo sapiens PE=2 SV=1 - [Q59EZ2_HUMAN]	1.16	4	2	2	1,903	207.7	6.99
D4YW74	Polytrophin OS=Homo sapiens GN=TROPH PE=2 SV=1 - [D4YW74_HUMAN]	0.37	2	2	2	6,825	788.0	5.47
Q00532	Cyclin-dependent kinase-like 1 OS=Homo sapiens GN=CDKL1 PE=2 SV=5 - [CDKL1_HUMAN]	6.44	1	2	2	357	41.6	8.85
Q8TC99	Fibronectin type III domain-containing protein 8 OS=Homo sapiens GN=FNDC8 PE=2 SV=2 - [FNDC8_HUMAN]	5.25	1	2	2	324	35.9	5.08
Q6ZW33	MICAL C-terminal-like protein OS=Homo sapiens GN=MICALCL PE=2 SV=3 - [MICLK_HUMAN]	5.18	1	2	2	695	77.2	8.50
P35579	Myosin-9 OS=Homo sapiens GN=MYH9 PE=1 SV=4 - [MYH9_HUMAN]	1.63	4	2	2	1,960	226.4	5.60
Q14957	Glutamate [NMDA] receptor subunit epsilon-3 OS=Homo sapiens GN=GRIN2C PE=2 SV=3 - [NMDE3_HUMAN]	2.76	5	2	2	1,233	134.1	8.48
Q86UU1	Pleckstrin homology-like domain family B member 1 OS=Homo sapiens GN=PHLDB1 PE=1 SV=1 - [PHLB1_HUMAN]	2.47	4	2	2	1,377	151.1	8.63
Q5T0A1	Dehydrodolichyl diphosphate synthase OS=Homo sapiens GN=DHDDS PE=2 SV=1 - [Q5T0A1_HUMAN]	13.88	16	2	2	209	24.1	8.35
B4DXJ6	cDNA FLJ61453, highly similar to Adapter-relatedprotein complex 2 alpha- 1 subunit OS=Homo sapiens PE=2 SV=1 - [B4DXJ6_HUMAN]	4.45	15	2	2	494	54.7	9.04
B4E0H8	cDNA FLJ60385, highly similar to Integrin alpha-3 OS=Homo sapiens PE=2 SV=1 - [B4E0H8_HUMAN]	3.18	5	2	2	1,037	115.1	6.57
B3KXI1	cDNA FLJ45428 fis, clone BRHIP3038735, highly similar to Homo sapiens papilin, proteoglycan-like sulfated glycoprotein (PAPLN), mRNA OS=Homo sapiens PE=2 SV=1 - [B3KXI1_HUMAN]	7.32	3	2	2	478	51.2	6.52
B3KWZ6	cDNA FLJ44343 fis, clone TRACH3005479, highly similar to Homo sapiens sperm associated antigen 17 (SPAG17), mRNA OS=Homo sapiens PE=2 SV=1 - [B3KWZ6_HUMAN]	1.85	3	2	2	1,082	120.9	6.21
Q8N3K9	Cardiomyopathy-associated protein 5 OS=Homo sapiens GN=CMYA5 PE=1 SV=3 - [CMYA5_HUMAN]	1.20	1	2	2	4,069	448.9	4.78
O43909	Exostosin-like 3 OS=Homo sapiens GN=EXTL3 PE=1 SV=1 - [EXTL3_HUMAN]	1.85	1	2	2	919	104.7	6.51
Q92945	Far upstream element-binding protein 2 OS=Homo sapiens GN=KHSRP PE=1 SV=4 - [FUBP2_HUMAN]	3.80	14	2	2	711	73.1	7.30
Q63ZY3	KN motif and ankyrin repeat domain-containing protein 2 OS=Homo sapiens GN=KANK2 PE=1 SV=1 - [KANK2_HUMAN]	2.23	1	2	2	851	91.1	5.63
Q8NA61	Spermatid-associated protein OS=Homo sapiens GN=SPERT PE=2 SV=1 - [SPERT_HUMAN]	6.03	2	2	2	448	51.5	7.15
Q5VUA4	Zinc finger protein 318 OS=Homo sapiens GN=ZNF318 PE=1 SV=2 - [ZN318_HUMAN]	0.79	1	2	2	2,279	251.0	7.20
B1AM31	Chromosome 1 open reading frame 125 (Fragment) OS=Homo sapiens GN=C1orf125 PE=4 SV=1 - [B1AM31_HUMAN]	1.95	4	2	2	872	101.8	5.97
B4DSN8	cDNA FLJ60863, highly similar to High mobility group protein 2-like 1 OS=Homo sapiens PE=2 SV=1 - [B4DSN8_HUMAN]	7.11	6	2	2	492	53.1	9.42
C9JV64	Ubiquitin carboxyl-terminal hydrolase OS=Homo sapiens GN=USP34 PE=3 SV=1 - [C9JV64_HUMAN]	1.15	4	2	2	3,311	377.2	5.92
A8MSY9	Uncharacterized protein OS=Homo sapiens GN=EFCAB5 PE=4 SV=1 - [A8MSY9_HUMAN]	3.35	4	2	2	926	106.8	5.38
B3KN48	cDNA FLJ13551 fis, clone PLACE1007140, weakly similar to Homo sapiens myosin, heavy polypeptide 10, non-muscle (MYH10), mRNA OS=Homo sapiens PE=2 SV=1 - [B3KN48_HUMAN]	2.03	4	2	2	789	92.9	6.51
E7EQ88	Uncharacterized protein OS=Homo sapiens GN=CDK20 PE=4 SV=1 - [E7EQ88_HUMAN]	7.78	4	2	2	450	49.5	6.90
A8K9U1	cDNA FLJ76468, highly similar to Homo sapiens cullin 7 (CUL7), mRNA OS=Homo sapiens PE=2 SV=1 - [A8K9U1_HUMAN]	1.47	5	2	2	1,698	191.0	5.96
C9J879	Uncharacterized protein OS=Homo sapiens GN=KDM4C PE=4 SV=1 - [C9J879_HUMAN]	3.32	7	2	2	573	64.4	5.31
B3KQH5	Integrator complex subunit 6, isoform CRA_b OS=Homo sapiens GN=INTS6 PE=2 SV=1 - [B3KQH5_HUMAN]	2.54	5	2	2	709	80.5	8.72
B4DLC8	cDNA FLJ59331, highly similar to Signal transducer and activator of transcription 2 OS=Homo sapiens PE=2 SV=1 - [B4DLC8_HUMAN]	4.13	4	2	2	460	53.0	7.50
B7Z6H4	DNA-directed RNA polymerase OS=Homo sapiens PE=2 SV=1 - [B7Z6H4_HUMAN]	3.21	2	2	2	1,369	153.4	8.48
Q5THM6	Endothelin converting enzyme 1 (Fragment) OS=Homo sapiens GN=ECE1 PE=2 SV=1 - [Q5THM6_HUMAN]	17.26	5	2	2	197	22.2	7.87
E9PGG4	Uncharacterized protein OS=Homo sapiens GN=ALS2CR11 PE=4 SV=1 - [E9PGG4_HUMAN]	1.87	3	2	2	1,820	208.9	7.62
D7RF68	AGTRAP-BRAF fusion protein OS=Homo sapiens PE=2 SV=1 - [D7RF68_HUMAN]	4.19	7	2	2	597	66.2	8.90
B4DK16	cDNA FLJ57882, highly similar to Pre-mRNA-processing-splicing factor 8 OS=Homo sapiens PE=2 SV=1 - [B4DK16_HUMAN]	3.76	3	2	2	1,065	123.4	8.16
Q6ZRK5	cDNA FLJ46283 fis, clone TESTI4031173 OS=Homo sapiens PE=2 SV=1 - [Q6ZRK5_HUMAN]	2.22	2	2	2	632	68.9	9.26
Q02952	A-kinase anchor protein 12 OS=Homo sapiens GN=AKAP12 PE=1 SV=4 - [AKA12_HUMAN]	1.80	2	2	2	1,782	191.4	4.41
O60673	DNA polymerase zeta catalytic subunit OS=Homo sapiens GN=REV3L PE=1 SV=2 - [DPOLZ_HUMAN]	0.58	3	2	2	3,130	352.6	8.47
B4E2M2	cDNA FLJ54903 OS=Homo sapiens PE=2 SV=1 - [B4E2M2_HUMAN]	4.96	2	2	2	383	44.4	9.06
A8K5T0	cDNA FLJ75416, highly similar to Homo sapiens complement factor H (CFH), mRNA OS=Homo sapiens PE=2 SV=1 - [A8K5T0_HUMAN]	49.96	5	1	52	1,231	138.9	6.71
E9PGN5	Uncharacterized protein OS=Homo sapiens GN=ITIH4 PE=4 SV=1 - [E9PGN5_HUMAN]	47.56	4	1	30	900	99.8	6.47
A8K2T4	cDNA FLJ78207, highly similar to Human complement protein component C7 mRNA OS=Homo sapiens PE=2 SV=1 - [A8K2T4_HUMAN]	42.94	7	1	28	843	93.3	6.51
P13671	Complement component C6 OS=Homo sapiens GN=C6 PE=1 SV=3 - [CO6_HUMAN]	29.34	9	1	26	934	104.7	6.76
A8K8Z4	cDNA FLJ78071, highly similar to Human MHC class III complement component C6 mRNA OS=Homo sapiens PE=2 SV=1 - [A8K8Z4_HUMAN]	31.26	9	1	26	934	104.6	6.62
Q6U2G1	C4B1 (Fragment) OS=Homo sapiens GN=C4B PE=4 SV=1 - [Q6U2G1_HUMAN]	61.05	15	1	23	534	58.3	6.42
Q13747	Alpha-1 antitrypsin (Fragment) OS=Homo sapiens PE=2 SV=1 - [Q13747_HUMAN]	74.11	7	1	20	197	22.8	6.55
Q86TT1	Full-length cDNA clone CS0DD006YL02 of Neuroblastoma of Homo sapiens (human) OS=Homo sapiens PE=2 SV=1 - [Q86TT1_HUMAN]	69.07	1	1	20	375	41.2	6.79
Q6MZW0	Putative uncharacterized protein DKFZp686J11235 (Fragment) OS=Homo sapiens GN=DKFZp686J11235 PE=1 SV=1 - [Q6MZW0_HUMAN]	46.05	7	1	17	506	54.4	6.77
P04220	Ig mu heavy chain disease protein OS=Homo sapiens PE=1 SV=1 - [MUCB_HUMAN]	54.73	1	1	16	391	43.0	5.24
B4E3S6	cDNA FLJ58413, highly similar to Complement component C7 OS=Homo sapiens PE=2 SV=1 - [B4E3S6_HUMAN]	41.98	5	1	16	486	53.7	5.40
Q6P089	IGH@ protein OS=Homo sapiens GN=IGH@ PE=1 SV=1 - [Q6P089_HUMAN]	49.17	5	1	16	480	52.0	6.32
Q6N096	Putative uncharacterized protein DKFZp686I15196 OS=Homo sapiens GN=DKFZp686I15196 PE=2 SV=1 - [Q6N096_HUMAN]	40.99	28	1	13	466	50.9	8.06
Q7Z351	Putative uncharacterized protein DKFZp686N02209 OS=Homo sapiens GN=DKFZp686N02209 PE=1 SV=1 - [Q7Z351_HUMAN]	36.72	9	1	12	482	52.8	8.48
P02750	Leucine-rich alpha-2-glycoprotein OS=Homo sapiens GN=LRG1 PE=1 SV=2 - [A2GL_HUMAN]	38.90	13	1	10	347	38.2	6.95
Q68CK4	Leucine-rich alpha-2-glycoprotein OS=Homo sapiens GN=HMFT1766 PE=2 SV=1 - [Q68CK4_HUMAN]	38.90	13	1	10	347	38.1	6.95
Q6GMV8	Putative uncharacterized protein OS=Homo sapiens PE=2 SV=1 - [Q6GMV8_HUMAN]	50.43	12	1	9	234	24.9	6.67
B3KWB5	cDNA FLJ42722 fis, clone BRAMY4000277, highly similar to Alpha-1B-glycoprotein OS=Homo sapiens PE=2 SV=1 - [B3KWB5_HUMAN]	43.48	1	1	9	345	37.0	5.62
Q6PJG0	Putative uncharacterized protein OS=Homo sapiens PE=1 SV=1 - [Q6PJG0_HUMAN]	43.40	13	1	8	235	24.6	7.30
Q6PIL8	IGK@ protein OS=Homo sapiens GN=IGK@ PE=1 SV=1 - [Q6PIL8_HUMAN]	49.58	8	1	8	236	25.8	6.55
A2NUT2	Lambda-chain (AA -20 to 215) OS=Homo sapiens PE=2 SV=1 - [A2NUT2_HUMAN]	43.40	20	1	8	235	24.6	7.62
A0A5E4	Putative uncharacterized protein OS=Homo sapiens PE=2 SV=1 - [A0A5E4_HUMAN]	43.83	6	1	8	235	24.7	5.94
Q8NEJ1	Putative uncharacterized protein OS=Homo sapiens PE=2 SV=1 - [Q8NEJ1_HUMAN]	45.34	13	1	8	236	25.0	7.69
Q9P1C5	PRO2769 OS=Homo sapiens PE=2 SV=1 - [Q9P1C5_HUMAN]	20.85	1	1	7	494	55.3	6.38
Q0KKI6	Immunoblobulin light chain (Fragment) OS=Homo sapiens PE=2 SV=1 - [Q0KKI6_HUMAN]	47.95	10	1	7	219	24.0	8.06
Q5HYM1	Putative uncharacterized protein DKFZp686O1553 (Fragment) OS=Homo sapiens GN=DKFZp686O1553 PE=1 SV=1 - [Q5HYM1_HUMAN]	17.33	6	1	7	479	54.0	5.86
B4DN21	cDNA FLJ53365, highly similar to Homo sapiens fibronectin 1 (FN1), transcript variant 4, mRNA OS=Homo sapiens PE=2 SV=1 - [B4DN21_HUMAN]	23.86	6	1	6	306	34.5	5.64
P01593	Ig kappa chain V-I region AG OS=Homo sapiens PE=1 SV=1 - [KV101_HUMAN]	37.96	12	1	3	108	12.0	5.99
Q9UL85	Myosin-reactive immunoglobulin kappa chain variable region (Fragment) OS=Homo sapiens PE=1 SV=1 - [Q9UL85_HUMAN]	31.19	5	1	3	109	11.8	8.51
Q0ZCG9	Immunglobulin heavy chain variable region (Fragment) OS=Homo sapiens PE=4 SV=1 - [Q0ZCG9_HUMAN]	30.58	31	1	3	121	13.3	5.99
A2MYD4	V2-7 protein (Fragment) OS=Homo sapiens GN=V2-7 PE=4 SV=1 - [A2MYD4_HUMAN]	44.79	3	1	3	96	10.3	5.01
Q65ZC9	Single-chain Fv (Fragment) OS=Homo sapiens GN=scFv PE=1 SV=1 - [Q65ZC9_HUMAN]	12.08	31	1	3	240	25.6	9.11
Q5NV90	V2-17 protein (Fragment) OS=Homo sapiens GN=IGLV3-25 PE=4 SV=1 - [Q5NV90_HUMAN]	41.24	4	1	3	97	10.4	4.59
Q14215	Nebulin (Fragment) OS=Homo sapiens PE=2 SV=1 - [Q14215_HUMAN]	1.13	3	1	3	3,007	348.8	9.10
Q9P0X4	Voltage-dependent T-type calcium channel subunit alpha-1I OS=Homo sapiens GN=CACNA1I PE=1 SV=1 - [CAC1I_HUMAN]	2.34	3	1	2	2,223	244.9	6.54
P01743	Ig heavy chain V-I region HG3 OS=Homo sapiens PE=4 SV=1 - [HV102_HUMAN]	22.22	4	1	2	117	12.9	8.92
P01608	Ig kappa chain V-I region Roy OS=Homo sapiens PE=1 SV=1 - [KV116_HUMAN]	24.07	10	1	2	108	11.8	5.36
P01619	Ig kappa chain V-III region B6 OS=Homo sapiens PE=1 SV=1 - [KV301_HUMAN]	21.30	2	1	2	108	11.6	9.25
P04434	Ig kappa chain V-III region VH (Fragment) OS=Homo sapiens PE=4 SV=1 - [KV310_HUMAN]	23.28	2	1	2	116	12.7	5.94
P01699	Ig lambda chain V-I region VOR OS=Homo sapiens PE=1 SV=1 - [LV101_HUMAN]	14.41	1	1	2	111	11.5	5.29
P01714	Ig lambda chain V-III region SH OS=Homo sapiens PE=1 SV=1 - [LV301_HUMAN]	25.00	5	1	2	108	11.4	6.52
Q96HE9	Proline-rich protein 11 OS=Homo sapiens GN=PRR11 PE=1 SV=1 - [PRR11_HUMAN]	6.94	1	1	2	360	40.1	10.11
A6NK21	Putative zinc finger protein LOC730110 OS=Homo sapiens PE=5 SV=3 - [YI017_HUMAN]	4.02	1	1	2	547	62.9	9.07
A2NKM6	NANUC-1 heavy chain (Fragment) OS=Homo sapiens PE=2 SV=1 - [A2NKM6_HUMAN]	14.05	16	1	2	121	13.1	9.95
A2IPI6	HRV Fab 027-VL (Fragment) OS=Homo sapiens PE=2 SV=1 - [A2IPI6_HUMAN]	20.35	2	1	2	113	12.4	9.41
A2NB44	Cold agglutinin FS-2 H-chain (Fragment) OS=Homo sapiens GN=IGH@ PE=2 SV=1 - [A2NB44_HUMAN]	13.16	7	1	2	114	12.8	7.11
A2JA19	Anti-mucin1 light chain variable region (Fragment) OS=Homo sapiens PE=2 SV=1 - [A2JA19_HUMAN]	21.50	2	1	2	107	11.7	8.91
A2NJV5	Kappa light chain variable region (Fragment) OS=Homo sapiens GN=IGKV A18 PE=4 SV=1 - [A2NJV5_HUMAN]	19.83	4	1	2	121	13.2	7.28
Q9UL86	Myosin-reactive immunoglobulin kappa chain variable region (Fragment) OS=Homo sapiens PE=1 SV=1 - [Q9UL86_HUMAN]	14.68	2	1	2	109	11.9	7.96
Q8TCZ8	Apolipoprotein E (Fragment) OS=Homo sapiens GN=APOE PE=2 SV=1 - [Q8TCZ8_HUMAN]	42.11	2	1	2	57	6.7	9.98
Q5XTR9	Hemoglobin delta-beta fusion protein (Fragment) OS=Homo sapiens GN=HBD/HBB PE=2 SV=1 - [Q5XTR9_HUMAN]	61.76	1	1	2	34	3.9	5.62
Q8TDB0	Apolipoprotein A-1 A175P variant (Fragment) OS=Homo sapiens PE=2 SV=1 - [Q8TDB0_HUMAN]	34.00	1	1	2	50	5.6	7.59
D3GKD8	A-gamma globin Osilo variant OS=Homo sapiens GN=HBG1 PE=3 SV=1 - [D3GKD8_HUMAN]	14.97	17	1	2	147	16.2	7.20
A2N7P4	Immunoglobulin mu-chain D-J4-region (Fragment) OS=Homo sapiens GN=IGHM PE=4 SV=1 - [A2N7P4_HUMAN]	21.67	3	1	2	120	13.3	9.26
B7ZMG8	Putative uncharacterized protein OS=Homo sapiens PE=4 SV=1 - [B7ZMG8_HUMAN]	24.10	9	1	2	83	9.1	4.59
P78492	Inter-alpha-trypsin inhibitor (Fragment) OS=Homo sapiens GN=ITIL PE=2 SV=1 - [P78492_HUMAN]	45.10	1	1	2	51	5.7	4.89
Q16671	Anti-Muellerian hormone type-2 receptor OS=Homo sapiens GN=AMHR2 PE=1 SV=1 - [AMHR2_HUMAN]	5.41	3	1	2	573	62.7	5.81
P98198	Probable phospholipid-transporting ATPase ID OS=Homo sapiens GN=ATP8B2 PE=1 SV=2 - [AT8B2_HUMAN]	1.41	5	1	2	1,209	137.4	7.01
Q9Y4D8	Probable E3 ubiquitin-protein ligase C12orf51 OS=Homo sapiens GN=C12orf51 PE=1 SV=5 - [K0614_HUMAN]	0.60	5	1	2	3,996	439.1	6.19
Q9C0D2	Protein KIAA1731 OS=Homo sapiens GN=KIAA1731 PE=2 SV=4 - [K1731_HUMAN]	0.77	1	1	2	2,601	295.0	6.00
P01610	Ig kappa chain V-I region WEA OS=Homo sapiens PE=1 SV=1 - [KV118_HUMAN]	30.56	10	1	2	108	11.8	8.91
Q9BT92	Trichoplein keratin filament-binding protein OS=Homo sapiens GN=TCHP PE=1 SV=1 - [TCHP_HUMAN]	2.81	34	1	2	498	61.0	6.54
E9PEW5	Uncharacterized protein OS=Homo sapiens GN=MYO10 PE=4 SV=1 - [E9PEW5_HUMAN]	1.00	9	1	2	1,397	161.4	6.25
Q0ZCH0	Immunglobulin heavy chain variable region (Fragment) OS=Homo sapiens PE=4 SV=1 - [Q0ZCH0_HUMAN]	15.13	7	1	2	119	13.0	8.81
A2J1N0	Rheumatoid factor RF-IP14 (Fragment) OS=Homo sapiens PE=2 SV=1 - [A2J1N0_HUMAN]	18.75	19	1	2	96	10.5	8.97
Q9HCC1	Single chain Fv (Fragment) OS=Homo sapiens PE=1 SV=1 - [Q9HCC1_HUMAN]	26.79	18	1	2	112	12.2	8.47
B2RDY3	cDNA, FLJ96827 OS=Homo sapiens PE=2 SV=1 - [B2RDY3_HUMAN]	6.33	3	1	2	490	55.4	7.02
A2N0T4	VH6DJ protein (Fragment) OS=Homo sapiens GN=VH6DJ PE=2 SV=1 - [A2N0T4_HUMAN]	16.84	21	1	2	95	10.6	8.47
A2NYV1	Heavy chain Fab (Fragment) OS=Homo sapiens PE=2 SV=1 - [A2NYV1_HUMAN]	14.06	7	1	2	128	14.3	6.54
A2MYE1	A30 (Fragment) OS=Homo sapiens PE=4 SV=1 - [A2MYE1_HUMAN]	34.38	11	1	2	96	10.4	8.50
E7ERQ2	Uncharacterized protein OS=Homo sapiens GN=PTPRD PE=4 SV=1 - [E7ERQ2_HUMAN]	3.33	8	1	2	1,383	156.0	6.55
Q9UL70	Myosin-reactive immunoglobulin light chain variable region (Fragment) OS=Homo sapiens PE=1 SV=1 - [Q9UL70_HUMAN]	31.48	10	1	2	108	11.6	9.36
Q4ZG57	Putative uncharacterized protein MCM6 (Fragment) OS=Homo sapiens GN=MCM6 PE=2 SV=1 - [Q4ZG57_HUMAN]	2.55	2	1	2	785	88.9	5.60
Q2PZI1	Protein dpy-19 homolog 1 OS=Homo sapiens GN=DPY19L1 PE=2 SV=1 - [D19L1_HUMAN]	2.67	1	1	2	675	77.3	8.95
Q13257	Mitotic spindle assembly checkpoint protein MAD2A OS=Homo sapiens GN=MAD2L1 PE=1 SV=1 - [MD2L1_HUMAN]	11.22	1	1	2	205	23.5	5.08
Q70Z35	Phosphatidylinositol 3,4,5-trisphosphate-dependent Rac exchanger 2 protein OS=Homo sapiens GN=PREX2 PE=2 SV=1 - [PREX2_HUMAN]	1.74	1	1	2	1,606	182.5	7.44
Q8N720	Zinc finger protein 655 OS=Homo sapiens GN=ZNF655 PE=1 SV=3 - [ZN655_HUMAN]	5.50	4	1	2	491	57.4	7.14
A1A5C4	RRBP1 protein OS=Homo sapiens GN=RRBP1 PE=2 SV=1 - [A1A5C4_HUMAN]	2.68	1	1	2	934	102.8	5.38
A0JP11	Phosphoinositide-3-kinase, regulatory subunit 4 OS=Homo sapiens GN=PIK3R4 PE=2 SV=1 - [A0JP11_HUMAN]	1.77	3	1	2	1,358	153.1	7.23
Q9HB03	Elongation of very long chain fatty acids protein 3 OS=Homo sapiens GN=ELOVL3 PE=1 SV=2 - [ELOV3_HUMAN]	14.44	1	1	2	270	31.5	9.51
Q92558	Wiskott-Aldrich syndrome protein family member 1 OS=Homo sapiens GN=WASF1 PE=1 SV=1 - [WASF1_HUMAN]	5.37	1	1	2	559	61.6	6.46
D2JYH6	Fibrillin 1 OS=Homo sapiens GN=FBN1 PE=4 SV=1 - [D2JYH6_HUMAN]	0.87	5	1	2	2,871	312.1	4.93
E5RJU0	Uncharacterized protein OS=Homo sapiens GN=UQCRB PE=4 SV=1 - [E5RJU0_HUMAN]	18.57	5	1	2	140	16.3	8.97
B7Z707	cDNA FLJ54182, highly similar to Deoxyribonuclease gamma (EC 3.1.21.-) OS=Homo sapiens PE=2 SV=1 - [B7Z707_HUMAN]	5.09	6	1	2	275	31.8	8.91
Q12982	BCL2/adenovirus E1B 19 kDa protein-interacting protein 2 OS=Homo sapiens GN=BNIP2 PE=1 SV=1 - [BNIP2_HUMAN]	4.14	1	1	2	314	36.0	4.81
Q9H6A9	Pecanex-like protein 3 OS=Homo sapiens GN=PCNXL3 PE=1 SV=2 - [PCX3_HUMAN]	0.93	4	1	2	2,034	221.9	6.64
B2RD46	cDNA, FLJ96449 OS=Homo sapiens PE=2 SV=1 - [B2RD46_HUMAN]	2.21	3	1	2	860	96.9	6.43
B4E1V0	cDNA FLJ54839, highly similar to Lactotransferrin (EC 3.4.21.-) OS=Homo sapiens PE=2 SV=1 - [B4E1V0_HUMAN]	8.33	16	1	2	300	33.0	7.64
Q5M9N0	Coiled-coil domain-containing protein 158 OS=Homo sapiens GN=CCDC158 PE=2 SV=2 - [CD158_HUMAN]	1.26	1	1	2	1,113	127.1	6.46
Q8TB92	Probable 3-hydroxymethyl-3-methylglutaryl-CoA lyase 2 OS=Homo sapiens GN=HMGCLL1 PE=2 SV=3 - [HMGC2_HUMAN]	4.05	4	1	2	370	39.5	6.58
Q5VU43	Myomegalin OS=Homo sapiens GN=PDE4DIP PE=1 SV=1 - [MYOME_HUMAN]	0.90	6	1	2	2,346	264.9	5.44
Q15149	Plectin OS=Homo sapiens GN=PLEC PE=1 SV=3 - [PLEC_HUMAN]	0.34	19	1	2	4,684	531.5	5.96
Q6N021	Methylcytosine dioxygenase TET2 OS=Homo sapiens GN=TET2 PE=1 SV=3 - [TET2_HUMAN]	2.60	2	1	2	2,002	223.7	7.99
B2RNA7	HCG1982192, isoform CRA_y OS=Homo sapiens GN=PCDHAC1 PE=2 SV=1 - [B2RNA7_HUMAN]	4.40	2	1	2	818	88.2	4.97
B4DMX3	cDNA FLJ56575, highly similar to cGMP-dependent protein kinase 2 (EC 2.7.11.12) OS=Homo sapiens PE=2 SV=1 - [B4DMX3_HUMAN]	2.05	6	1	2	733	84.3	8.40
Q7Z3M0	Putative uncharacterized protein DKFZp686M1993 (Fragment) OS=Homo sapiens GN=DKFZp686M1993 PE=2 SV=1 - [Q7Z3M0_HUMAN]	1.31	12	1	2	1,605	183.0	5.59
Q4ZG20	Putative uncharacterized protein TTN OS=Homo sapiens GN=TTN PE=2 SV=1 - [Q4ZG20_HUMAN]	0.66	3	1	2	5,604	631.2	5.73
Q8TF62	Probable phospholipid-transporting ATPase IM OS=Homo sapiens GN=ATP8B4 PE=2 SV=3 - [AT8B4_HUMAN]	2.18	6	1	2	1,192	135.8	6.99
Q8N2F8	cDNA PSEC0195 fis, clone HEMBA1001322, highly similar to ALPHA-ADAPTIN C OS=Homo sapiens PE=2 SV=1 - [Q8N2F8_HUMAN]	10.49	2	1	2	467	51.2	7.43
D6RBW0	Uncharacterized protein OS=Homo sapiens GN=CENPE PE=3 SV=1 - [D6RBW0_HUMAN]	2.66	2	1	2	1,126	130.4	5.14
Q6LDG4	Complement protein (Fragment) OS=Homo sapiens GN=C2 PE=4 SV=1 - [Q6LDG4_HUMAN]	30.16	2	1	2	63	7.2	7.18
B7ZLC9	GEMIN5 protein OS=Homo sapiens GN=GEMIN5 PE=2 SV=1 - [B7ZLC9_HUMAN]	1.86	3	1	2	1,507	168.3	6.62
Q9UL71	Myosin-reactive immunoglobulin heavy chain variable region (Fragment) OS=Homo sapiens PE=1 SV=1 - [Q9UL71_HUMAN]	24.79	4	1	2	121	13.1	7.12
B4E0B2	cDNA FLJ51426, highly similar to Adapter-related protein complex 3 beta-1 subunit OS=Homo sapiens PE=2 SV=1 - [B4E0B2_HUMAN]	3.89	5	1	2	565	63.7	8.28
Q6ZU25	cDNA FLJ44043 fis, clone TESTI4029836, highly similar to Potential phospholipid-transporting ATPase IB (EC 3.6.3.13) OS=Homo sapiens PE=2 SV=1 - [Q6ZU25_HUMAN]	2.69	5	1	2	968	109.0	7.71
Q6P461	Acyl-coenzyme A synthetase ACSM6, mitochondrial OS=Homo sapiens GN=ACSM6 PE=2 SV=3 - [ACSM6_HUMAN]	5.21	1	1	2	480	53.5	8.41
B7Z753	cDNA FLJ53221, highly similar to Ral guanine nucleotide dissociation stimulator OS=Homo sapiens PE=2 SV=1 - [B7Z753_HUMAN]	3.62	10	1	2	885	97.3	6.07
Q5H9P4	Putative uncharacterized protein DKFZp686M19106 (Fragment) OS=Homo sapiens GN=DKFZp686M19106 PE=2 SV=1 - [Q5H9P4_HUMAN]	3.57	8	1	2	924	99.8	6.32
B4DHJ6	cDNA FLJ58180, highly similar to Ankyrin repeat domain-containing protein 50 OS=Homo sapiens PE=2 SV=1 - [B4DHJ6_HUMAN]	1.84	3	1	2	1,250	136.4	6.35
B7Z250	cDNA FLJ56324, highly similar to Hepatocyte growth factor-like protein OS=Homo sapiens PE=2 SV=1 - [B7Z250_HUMAN]	5.60	1	1	2	339	38.4	7.34
A2N2F1	VL4 protein (Fragment) OS=Homo sapiens GN=VL4 PE=2 SV=1 - [A2N2F1_HUMAN]	19.05	5	1	2	105	11.3	8.94
Q12955	Ankyrin-3 OS=Homo sapiens GN=ANK3 PE=1 SV=3 - [ANK3_HUMAN]	0.41	12	1	2	4,377	480.1	6.49
B4DVX3	cDNA FLJ50105, highly similar to Jumonji/ARID domain-containing protein 1A (Fragment) OS=Homo sapiens PE=2 SV=1 - [B4DVX3_HUMAN]	3.92	5	1	2	715	81.4	6.28
B7Z9A3	cDNA FLJ56215, moderately similar to Homo sapiens murine retrovirus integration site 1 homolog (MRVI1), transcript variant 2, mRNA OS=Homo sapiens PE=2 SV=1 - [B7Z9A3_HUMAN]	3.40	6	1	2	706	76.9	5.26
Q6NUT2	Protein dpy-19 homolog 2 OS=Homo sapiens GN=DPY19L2 PE=1 SV=2 - [D19L2_HUMAN]	2.11	2	1	2	758	87.3	9.10
Q14204	Cytoplasmic dynein 1 heavy chain 1 OS=Homo sapiens GN=DYNC1H1 PE=1 SV=5 - [DYHC1_HUMAN]	0.67	1	1	2	4,646	532.1	6.40
Q9HC58	Sodium/potassium/calcium exchanger 3 OS=Homo sapiens GN=SLC24A3 PE=2 SV=4 - [NCKX3_HUMAN]	3.73	1	1	2	644	71.9	5.35
Q12879	Glutamate [NMDA] receptor subunit epsilon-1 OS=Homo sapiens GN=GRIN2A PE=1 SV=1 - [NMDE1_HUMAN]	1.71	2	1	2	1,464	165.2	7.11
Q5VU65	Nuclear pore membrane glycoprotein 210-like OS=Homo sapiens GN=NUP210L PE=2 SV=1 - [P210L_HUMAN]	1.32	1	1	2	1,888	210.5	7.50
B7Z2A7	cDNA FLJ53837, moderately similar to Homo sapiens acyl-Coenzyme A binding domain containing 5 (ACBD5), mRNA OS=Homo sapiens PE=2 SV=1 - [B7Z2A7_HUMAN]	6.90	5	1	2	348	38.7	4.92
Q6ZU67	BEN domain-containing protein 4 OS=Homo sapiens GN=BEND4 PE=1 SV=3 - [BEND4_HUMAN]	2.06	1	1	1	534	58.3	6.13
Q9Y6R9	Coiled-coil domain-containing protein 61 OS=Homo sapiens GN=CCDC61 PE=1 SV=2 - [CCD61_HUMAN]	2.95	1	1	1	474	53.2	9.95
P02462	Collagen alpha-1(IV) chain OS=Homo sapiens GN=COL4A1 PE=1 SV=3 - [CO4A1_HUMAN]	1.56	1	1	1	1,669	160.5	8.28
Q8N4Y2	EF-hand calcium-binding domain-containing protein 4A OS=Homo sapiens GN=EFCAB4A PE=2 SV=3 - [EFC4A_HUMAN]	4.01	2	1	1	399	44.9	5.34
Q96CN4	EVI5-like protein OS=Homo sapiens GN=EVI5L PE=1 SV=1 - [EVI5L_HUMAN]	1.89	2	1	1	794	91.3	5.34
Q6V0I7	Protocadherin Fat 4 OS=Homo sapiens GN=FAT4 PE=2 SV=2 - [FAT4_HUMAN]	0.34	1	1	1	4,981	542.4	4.94
Q9NS66	Probable G-protein coupled receptor 173 OS=Homo sapiens GN=GPR173 PE=2 SV=1 - [GP173_HUMAN]	1.88	1	1	1	373	41.5	9.16
P42701	Interleukin-12 receptor subunit beta-1 OS=Homo sapiens GN=IL12RB1 PE=1 SV=1 - [I12R1_HUMAN]	1.51	1	1	1	662	73.1	5.44
P01612	Ig kappa chain V-I region Mev OS=Homo sapiens PE=1 SV=1 - [KV120_HUMAN]	16.51	1	1	1	109	11.9	6.57
O95907	Monocarboxylate transporter 3 OS=Homo sapiens GN=SLC16A8 PE=2 SV=1 - [MOT3_HUMAN]	1.19	1	1	1	504	52.3	5.67
Q6NZ67	Mitotic-spindle organizing protein 2B OS=Homo sapiens GN=MZT2B PE=1 SV=1 - [MZT2B_HUMAN]	13.92	3	1	1	158	16.2	10.15
Q9HC10	Otoferlin OS=Homo sapiens GN=OTOF PE=1 SV=3 - [OTOF_HUMAN]	1.55	1	1	1	1,997	226.6	5.69
Q9UN70	Protocadherin gamma-C3 OS=Homo sapiens GN=PCDHGC3 PE=1 SV=1 - [PCDGK_HUMAN]	0.75	1	1	1	934	101.0	5.21
Q7LBE3	Solute carrier family 26 member 9 OS=Homo sapiens GN=SLC26A9 PE=2 SV=1 - [S26A9_HUMAN]	0.88	2	1	1	791	86.9	8.22
Q9Y6X3	MAU2 chromatid cohesion factor homolog OS=Homo sapiens GN=MAU2 PE=1 SV=2 - [SCC4_HUMAN]	2.45	1	1	1	613	69.0	7.25
Q6ZMT1	SH3 and cysteine-rich domain-containing protein 2 OS=Homo sapiens GN=STAC2 PE=2 SV=1 - [STAC2_HUMAN]	1.95	1	1	1	411	45.0	7.31
Q96Q05	Trafficking protein particle complex subunit 9 OS=Homo sapiens GN=TRAPPC9 PE=1 SV=2 - [TPPC9_HUMAN]	0.87	2	1	1	1,148	128.4	6.62
Q2M2E5	Uncharacterized protein FLJ37543 OS=Homo sapiens PE=2 SV=2 - [YE017_HUMAN]	9.23	1	1	1	130	14.8	9.47
B1ANM7	Fas (TNFRSF6) associated factor 1 OS=Homo sapiens GN=FAF1 PE=4 SV=1 - [B1ANM7_HUMAN]	1.22	3	1	1	490	56.0	5.08
Q5VV23	Chromosome 10 open reading frame 63 OS=Homo sapiens GN=C10orf63 PE=2 SV=1 - [Q5VV23_HUMAN]	6.27	2	1	1	255	29.4	9.35
C9JE89	Motile sperm domain containing 3, isoform CRA_b OS=Homo sapiens GN=MOSPD3 PE=4 SV=1 - [C9JE89_HUMAN]	3.11	3	1	1	225	24.4	8.88
D3DWC9	Placenta-specific 9, isoform CRA_a OS=Homo sapiens GN=PLAC9 PE=4 SV=1 - [D3DWC9_HUMAN]	17.14	1	1	1	140	14.9	12.21
B1ALL0	Testis expressed 11 (Fragment) OS=Homo sapiens GN=TEX11 PE=4 SV=1 - [B1ALL0_HUMAN]	1.72	3	1	1	754	86.8	5.16
B7Z9J8	cDNA, FLJ78862, highly similar to Isocitrate dehydrogenase OS=Homo sapiens PE=2 SV=1 - [B7Z9J8_HUMAN]	6.61	6	1	1	257	28.1	5.66
B3KTT7	Pannexin 2, isoform CRA_a OS=Homo sapiens GN=PANX2 PE=2 SV=1 - [B3KTT7_HUMAN]	4.05	2	1	1	667	73.2	8.27
Q6ZNT1	CDNA FLJ27213 fis, clone SYN03652 OS=Homo sapiens PE=2 SV=1 - [Q6ZNT1_HUMAN]	17.42	1	1	1	155	17.0	7.93
B4DMI2	cDNA FLJ57533, highly similar to Proprotein convertase subtilisin/kexin type 9 (EC 3.4.21.-) OS=Homo sapiens PE=2 SV=1 - [B4DMI2_HUMAN]	5.64	1	1	1	266	27.8	6.84
B7Z240	cDNA FLJ55050, highly similar to Epidermal growth factor receptor substrate 15 OS=Homo sapiens PE=2 SV=1 - [B7Z240_HUMAN]	1.05	1	1	1	573	63.6	4.49
D2KTB3	Helicase-like protein (Fragment) OS=Homo sapiens GN=Si-11-6 PE=2 SV=1 - [D2KTB3_HUMAN]	8.09	7	1	1	309	35.0	8.13
A2NW98	Rheumatoid factor light chain variable region (Fragment) OS=Homo sapiens PE=2 SV=1 - [A2NW98_HUMAN]	6.72	1	1	1	134	14.8	7.12
B4DU38	cDNA FLJ57795, highly similar to Isocitrate dehydrogenase OS=Homo sapiens PE=2 SV=1 - [B4DU38_HUMAN]	24.81	3	1	1	133	14.4	6.68
Q9Y417	Putative uncharacterized protein DKFZp586N041 (Fragment) OS=Homo sapiens GN=DKFZp586N041 PE=2 SV=1 - [Q9Y417_HUMAN]	2.17	2	1	1	276	31.7	5.54
B3KWQ0	cDNA FLJ43557 fis, clone PROST2018588, highly similar to Rap guanine nucleotide exchange factor 3 OS=Homo sapiens PE=2 SV=1 - [B3KWQ0_HUMAN]	1.23	7	1	1	570	64.2	8.51
Q59F65	G protein pathway suppressor 1 isoform 2 variant (Fragment) OS=Homo sapiens PE=2 SV=1 - [Q59F65_HUMAN]	3.87	1	1	1	284	30.4	8.37
C9JVG1	Uncharacterized protein OS=Homo sapiens GN=CPB1 PE=4 SV=1 - [C9JVG1_HUMAN]	7.41	3	1	1	189	21.9	7.12
Q5NV84	V1-3 protein (Fragment) OS=Homo sapiens GN=IGLV2-11 PE=4 SV=1 - [Q5NV84_HUMAN]	8.08	1	1	1	99	10.5	7.06
Q05DN2	IFIT2 protein (Fragment) OS=Homo sapiens GN=IFIT2 PE=2 SV=1 - [Q05DN2_HUMAN]	1.46	18	1	1	412	48.0	8.16
Q5T266	Zinc finger, DHHC-type containing 12 (Fragment) OS=Homo sapiens GN=ZDHHC12 PE=2 SV=1 - [Q5T266_HUMAN]	6.83	3	1	1	205	23.1	8.34
A2MYD6	V1-2 protein (Fragment) OS=Homo sapiens GN=V1-2 PE=4 SV=1 - [A2MYD6_HUMAN]	8.08	2	1	1	99	10.3	5.91
B4DEL8	cDNA FLJ59128, highly similar to Neurogenic locus notch homolog protein 4 OS=Homo sapiens PE=2 SV=1 - [B4DEL8_HUMAN]	4.00	1	1	1	200	21.3	7.34
Q96DY3	SLC27A1 protein (Fragment) OS=Homo sapiens GN=SLC27A1 PE=2 SV=1 - [Q96DY3_HUMAN]	8.75	3	1	1	240	26.0	7.91
B4DNL0	cDNA FLJ52959, highly similar to Growth hormone-inducible transmembrane protein OS=Homo sapiens PE=2 SV=1 - [B4DNL0_HUMAN]	7.61	7	1	1	276	30.1	9.73
B5MDL6	Uncharacterized protein OS=Homo sapiens GN=KIAA1409 PE=4 SV=1 - [B5MDL6_HUMAN]	0.62	3	1	1	2,419	270.7	6.24
B4DS48	cDNA FLJ54434, highly similar to Myelin transcription factor 1-like protein (Fragment) OS=Homo sapiens PE=2 SV=1 - [B4DS48_HUMAN]	1.91	3	1	1	942	105.9	4.89
Q59F23	MHC class II antigen variant (Fragment) OS=Homo sapiens PE=2 SV=1 - [Q59F23_HUMAN]	6.06	1	1	1	165	19.2	9.66
E9PMM3	Uncharacterized protein OS=Homo sapiens GN=PEX16 PE=4 SV=1 - [E9PMM3_HUMAN]	5.46	4	1	1	183	21.2	10.18
O00439	Putative uncharacterized protein OS=Homo sapiens GN=FBXO46 PE=2 SV=1 - [O00439_HUMAN]	3.96	1	1	1	202	21.7	9.60
Q9BTR6	Putative uncharacterized protein (Fragment) OS=Homo sapiens PE=2 SV=1 - [Q9BTR6_HUMAN]	3.39	5	1	1	295	32.5	6.84
A2A4F6	Centrosomal protein 350kDa (Fragment) OS=Homo sapiens GN=CEP350 PE=4 SV=1 - [A2A4F6_HUMAN]	13.08	1	1	1	130	14.6	10.04
A8K383	cDNA FLJ75428, highly similar to Homo sapiens activating transcription factor 6 (ATF6), mRNA OS=Homo sapiens PE=2 SV=1 - [A8K383_HUMAN]	1.79	2	1	1	670	74.5	8.22
Q32Q26	ANTXR2 protein OS=Homo sapiens GN=ANTXR2 PE=2 SV=1 - [Q32Q26_HUMAN]	2.43	3	1	1	411	44.8	8.18
E9PG49	Uncharacterized protein OS=Homo sapiens GN=SCN1A PE=4 SV=1 - [E9PG49_HUMAN]	0.71	3	1	1	1,981	226.0	5.92
C9J8Q1	Uncharacterized protein OS=Homo sapiens GN=NFU1 PE=4 SV=1 - [C9J8Q1_HUMAN]	6.25	4	1	1	96	10.4	4.12
Q5T045	Novel protein (Fragment) OS=Homo sapiens GN=RP11-480N24.2-002 PE=2 SV=1 - [Q5T045_HUMAN]	21.05	4	1	1	38	4.4	8.79
B9EK47	HEAT repeat containing 5B OS=Homo sapiens GN=HEATR5B PE=2 SV=1 - [B9EK47_HUMAN]	0.68	2	1	1	2,071	224.1	7.18
B4DQ88	cDNA FLJ52014, highly similar to EGF-like module-containing mucin-like hormone receptor-like 2 OS=Homo sapiens PE=2 SV=1 - [B4DQ88_HUMAN]	8.44	1	1	1	225	25.0	6.83
Q16333	FLT (Fragment) OS=Homo sapiens GN=flt PE=2 SV=1 - [Q16333_HUMAN]	100.00	1	1	1	24	2.7	5.48
B4DT66	cDNA FLJ55367, highly similar to Rattus norvegicus SDA1 domain containing 1 (Sdad1), mRNA OS=Homo sapiens PE=2 SV=1 - [B4DT66_HUMAN]	2.92	3	1	1	650	75.5	9.22
B4E0E2	cDNA FLJ60205, highly similar to Tyrosine-protein kinase ZAP-70 (EC 2.7.10.2) OS=Homo sapiens PE=2 SV=1 - [B4E0E2_HUMAN]	10.27	2	1	1	185	20.7	6.52
Q59GS2	Chemokine (C-X3-C motif) ligand 1 variant (Fragment) OS=Homo sapiens PE=2 SV=1 - [Q59GS2_HUMAN]	2.62	4	1	1	381	40.3	6.29
B3KUN4	cDNA FLJ40302 fis, clone TESTI2029196, highly similar to Rhomboid-related protein 2 (EC 3.4.21.105) OS=Homo sapiens PE=2 SV=1 - [B3KUN4_HUMAN]	5.14	2	1	1	370	41.5	6.54
Q29RY0	SNTA1 protein (Fragment) OS=Homo sapiens GN=SNTA1 PE=2 SV=1 - [Q29RY0_HUMAN]	8.49	4	1	1	212	22.8	7.83
A4ZI32	Fanconi anemia core complex 100 kDa subunit OS=Homo sapiens PE=2 SV=1 - [A4ZI32_HUMAN]	1.02	2	1	1	881	93.3	5.25
B7Z628	cDNA FLJ59632, highly similar to Epidermis-type lipoxygenase 3 (EC 1.13.11.-) OS=Homo sapiens PE=2 SV=1 - [B7Z628_HUMAN]	5.36	5	1	1	504	57.0	8.48
C9J7I8	Uncharacterized protein OS=Homo sapiens GN=HNF4A PE=3 SV=1 - [C9J7I8_HUMAN]	2.62	7	1	1	382	42.7	8.13
B4E124	cDNA FLJ53264, highly similar to Ankyrin repeat domain-containing protein 41 OS=Homo sapiens PE=2 SV=1 - [B4E124_HUMAN]	5.01	6	1	1	439	46.6	6.07
B4DZ15	cDNA FLJ57285 OS=Homo sapiens PE=2 SV=1 - [B4DZ15_HUMAN]	0.90	3	1	1	781	91.0	8.91
Q59GF3	Aquaporin 4 isoform a variant (Fragment) OS=Homo sapiens PE=2 SV=1 - [Q59GF3_HUMAN]	6.85	1	1	1	219	23.8	7.59
B4E2F3	cDNA FLJ53277, highly similar to Conserved oligomeric Golgi complex component 3 OS=Homo sapiens PE=2 SV=1 - [B4E2F3_HUMAN]	0.90	2	1	1	665	75.7	6.62
Q3ZB85	PCDHB9 protein (Fragment) OS=Homo sapiens GN=PCDHB9 PE=2 SV=1 - [Q3ZB85_HUMAN]	0.75	2	1	1	796	86.9	5.02
Q05BP9	OLIG2 protein (Fragment) OS=Homo sapiens GN=OLIG2 PE=2 SV=1 - [Q05BP9_HUMAN]	8.89	4	1	1	270	27.3	9.41
C9JXH6	Uncharacterized protein OS=Homo sapiens GN=DNAH1 PE=4 SV=1 - [C9JXH6_HUMAN]	0.33	2	1	1	4,265	487.2	5.83
B2RDI7	cDNA, FLJ96629, highly similar to Homo sapiens ring finger protein 10 (RNF10), mRNA OS=Homo sapiens PE=2 SV=1 - [B2RDI7_HUMAN]	2.10	4	1	1	811	89.9	6.92
C9JPG5	Uncharacterized protein OS=Homo sapiens GN=SEMA3F PE=4 SV=1 - [C9JPG5_HUMAN]	0.87	4	1	1	686	77.3	7.96
Q658Z0	Putative uncharacterized protein DKFZp666F111 (Fragment) OS=Homo sapiens GN=DKFZp666F111 PE=2 SV=1 - [Q658Z0_HUMAN]	11.11	3	1	1	117	13.9	10.11
Q86VZ9	AngRem52 OS=Homo sapiens PE=2 SV=1 - [Q86VZ9_HUMAN]	4.71	1	1	1	340	37.4	9.01
B4DH82	cDNA FLJ54437, highly similar to RRP5 protein homolog (Fragment) OS=Homo sapiens PE=2 SV=1 - [B4DH82_HUMAN]	1.46	2	1	1	1,299	143.1	8.32
Q6ZPD0	cDNA FLJ26027 fis, clone PNC04328, highly similar to Homo sapiens translocase of outer mitochondrial membrane 34 (TOMM34) OS=Homo sapiens PE=2 SV=1 - [Q6ZPD0_HUMAN]	4.40	3	1	1	182	20.3	8.60
C9JVC3	Uncharacterized protein OS=Homo sapiens GN=ZNF717 PE=4 SV=1 - [C9JVC3_HUMAN]	1.74	3	1	1	864	100.4	8.78
Q71RD7	PP10897 OS=Homo sapiens PE=2 SV=1 - [Q71RD7_HUMAN]	15.87	1	1	1	189	21.3	7.25
B3KWD2	cDNA FLJ42797 fis, clone BRAWH3008697, highly similar to Homo sapiens pleckstrin homology domain containing, family H member 1, mRNA OS=Homo sapiens PE=2 SV=1 - [B3KWD2_HUMAN]	12.23	3	1	1	229	25.3	8.46
O94911	ATP-binding cassette sub-family A member 8 OS=Homo sapiens GN=ABCA8 PE=1 SV=3 - [ABCA8_HUMAN]	0.38	3	1	1	1,581	179.1	7.18
Q09428	ATP-binding cassette sub-family C member 8 OS=Homo sapiens GN=ABCC8 PE=1 SV=6 - [ABCC8_HUMAN]	1.08	1	1	1	1,581	176.9	7.81
Q9H6R3	Acyl-CoA synthetase short-chain family member 3, mitochondrial OS=Homo sapiens GN=ACSS3 PE=2 SV=1 - [ACSS3_HUMAN]	3.35	1	1	1	686	74.7	8.63
O43423	Acidic leucine-rich nuclear phosphoprotein 32 family member C OS=Homo sapiens GN=ANP32C PE=2 SV=1 - [AN32C_HUMAN]	2.56	1	1	1	234	26.7	4.21
Q9H6S1	5-azacytidine-induced protein 2 OS=Homo sapiens GN=AZI2 PE=1 SV=1 - [AZI2_HUMAN]	4.34	1	1	1	392	44.9	6.60
Q14004	Cyclin-dependent kinase 13 OS=Homo sapiens GN=CDK13 PE=1 SV=2 - [CDK13_HUMAN]	1.19	1	1	1	1,512	164.8	9.69
Q15828	Cystatin-M OS=Homo sapiens GN=CST6 PE=1 SV=1 - [CYTM_HUMAN]	13.42	2	1	1	149	16.5	8.09
Q9UHL4	Dipeptidyl peptidase 2 OS=Homo sapiens GN=DPP7 PE=1 SV=3 - [DPP2_HUMAN]	5.49	1	1	1	492	54.3	6.32
Q96L91	E1A-binding protein p400 OS=Homo sapiens GN=EP400 PE=1 SV=4 - [EP400_HUMAN]	0.19	1	1	1	3,159	343.3	9.19
Q5T0W9	Protein FAM83B OS=Homo sapiens GN=FAM83B PE=1 SV=1 - [FA83B_HUMAN]	0.79	1	1	1	1,011	114.7	8.97
O75015	Low affinity immunoglobulin gamma Fc region receptor III-B OS=Homo sapiens GN=FCGR3B PE=1 SV=2 - [FCG3B_HUMAN]	3.43	4	1	1	233	26.2	6.71
Q9BSK4	Protein fem-1 homolog A OS=Homo sapiens GN=FEM1A PE=1 SV=1 - [FEM1A_HUMAN]	1.20	1	1	1	669	73.6	6.07
Q7Z4P5	Growth/differentiation factor 7 OS=Homo sapiens GN=GDF7 PE=2 SV=2 - [GDF7_HUMAN]	3.33	2	1	1	450	46.9	9.80
Q96GX5	Serine/threonine-protein kinase greatwall OS=Homo sapiens GN=MASTL PE=1 SV=1 - [GWL_HUMAN]	1.14	1	1	1	879	97.3	5.99
Q9NP08	Homeobox protein HMX1 OS=Homo sapiens GN=HMX1 PE=2 SV=2 - [HMX1_HUMAN]	2.87	1	1	1	348	36.1	6.65
P01814	Ig heavy chain V-II region OU OS=Homo sapiens PE=1 SV=1 - [HV201_HUMAN]	5.56	2	1	1	126	14.3	9.83
Q9BZV3	Interphotoreceptor matrix proteoglycan 2 OS=Homo sapiens GN=IMPG2 PE=1 SV=3 - [IMPG2_HUMAN]	0.48	1	1	1	1,241	138.5	4.61
Q2LD37	Uncharacterized protein KIAA1109 OS=Homo sapiens GN=KIAA1109 PE=1 SV=2 - [K1109_HUMAN]	0.26	1	1	1	5,005	555.1	6.58
Q9UFC0	Leucine-rich repeat and WD repeat-containing protein 1 OS=Homo sapiens GN=LRWD1 PE=1 SV=2 - [LRWD1_HUMAN]	2.63	1	1	1	647	70.8	7.21
P0C7V9	Putative methyltransferase-like protein 15P1 OS=Homo sapiens GN=METTL15P1 PE=5 SV=1 - [ME15P_HUMAN]	13.68	1	1	1	234	26.7	5.96
Q3V5L5	Alpha-1,6-mannosylglycoprotein 6-beta-N-acetylglucosaminyltransferase B OS=Homo sapiens GN=MGAT5B PE=2 SV=2 - [MGT5B_HUMAN]	1.26	1	1	1	792	89.5	8.35
O15091	Mitochondrial ribonuclease P protein 3 OS=Homo sapiens GN=KIAA0391 PE=1 SV=2 - [MRRP3_HUMAN]	2.92	1	1	1	583	67.3	8.78
Q9NQR4	Omega-amidase NIT2 OS=Homo sapiens GN=NIT2 PE=1 SV=1 - [NIT2_HUMAN]	6.52	1	1	1	276	30.6	7.21
Q6ZRI0	Otogelin OS=Homo sapiens GN=OTOG PE=2 SV=3 - [OTOG_HUMAN]	1.03	1	1	1	2,925	314.6	5.91
O43295	SLIT-ROBO Rho GTPase-activating protein 3 OS=Homo sapiens GN=SRGAP3 PE=1 SV=3 - [SRGP2_HUMAN]	1.91	1	1	1	1,099	124.4	6.68
Q9BX26	Synaptonemal complex protein 2 OS=Homo sapiens GN=SYCP2 PE=2 SV=2 - [SYCP2_HUMAN]	0.85	1	1	1	1,530	175.5	8.85
A6NKL6	Transmembrane protein 200C OS=Homo sapiens GN=TMEM200C PE=2 SV=2 - [T200C_HUMAN]	1.13	1	1	1	621	63.9	10.08
Q6ZRN7	Putative uncharacterized protein FLJ46214 OS=Homo sapiens PE=2 SV=1 - [YP029_HUMAN]	7.21	1	1	1	208	21.1	11.78
P24278	Zinc finger and BTB domain-containing protein 25 OS=Homo sapiens GN=ZBTB25 PE=1 SV=2 - [ZBT25_HUMAN]	1.38	1	1	1	435	49.0	6.55
B7Z685	Guanylate cyclase 1, soluble, beta 3, isoform CRA_c OS=Homo sapiens GN=GUCY1B3 PE=2 SV=1 - [B7Z685_HUMAN]	1.45	8	1	1	551	62.7	5.58
Q8WTZ0	Hyaluronan synthase 3 OS=Homo sapiens GN=HAS3 PE=2 SV=1 - [Q8WTZ0_HUMAN]	9.96	3	1	1	281	31.2	5.99
B3KVZ3	Centromere protein H, isoform CRA_b OS=Homo sapiens GN=CENPH PE=2 SV=1 - [B3KVZ3_HUMAN]	3.51	2	1	1	228	26.2	4.93
B4DSZ2	cDNA FLJ50653, highly similar to Rattus norvegicus CUG triplet repeat, RNA binding protein 2 (Cugbp2), mRNA OS=Homo sapiens PE=2 SV=1 - [B4DSZ2_HUMAN]	5.03	9	1	1	159	18.0	7.75
B5MDA4	HCG22147, isoform CRA_d OS=Homo sapiens GN=LRRC68 PE=4 SV=1 - [B5MDA4_HUMAN]	6.25	2	1	1	608	66.3	4.97
B0QYH2	HscB iron-sulfur cluster co-chaperone homolog (E. coli) OS=Homo sapiens GN=HSCB PE=4 SV=1 - [B0QYH2_HUMAN]	13.99	2	1	1	143	16.5	9.57
B3KQS8	Matrix metallopeptidase 11 (Stromelysin 3), isoform CRA_b OS=Homo sapiens GN=MMP11 PE=2 SV=1 - [B3KQS8_HUMAN]	4.80	3	1	1	354	38.8	6.37
A0N4Z5	HCG2039777 (Fragment) OS=Homo sapiens GN=Tcr-alpha PE=4 SV=1 - [A0N4Z5_HUMAN]	42.11	5	1	1	19	2.1	9.99
A6XGL4	C17orf87 protein OS=Homo sapiens GN=UNQ5783 PE=2 SV=1 - [A6XGL4_HUMAN]	11.59	2	1	1	138	15.7	4.86
D3DU30	Intercellular adhesion molecule 2, isoform CRA_b OS=Homo sapiens GN=ICAM2 PE=4 SV=1 - [D3DU30_HUMAN]	5.88	4	1	1	255	27.8	6.89
D6CHE9	Proteinase 3 OS=Homo sapiens GN=PRTN3 PE=2 SV=1 - [D6CHE9_HUMAN]	5.58	2	1	1	215	23.6	8.16
Q9H5M2	CDNA: FLJ23306 fis, clone HEP11541 OS=Homo sapiens GN=FOSL2 PE=2 SV=1 - [Q9H5M2_HUMAN]	16.39	1	1	1	122	12.9	5.59
Q53EL5	Zinc finger, BED domain containing 3 variant (Fragment) OS=Homo sapiens GN=ZBED3 PE=2 SV=1 - [Q53EL5_HUMAN]	2.99	2	1	1	234	25.1	8.31
D3K175	Solute carrier family 4 sodium bicarbonate cotransporter member 7 truncated type 1b variant OS=Homo sapiens PE=2 SV=1 - [D3K175_HUMAN]	4.28	7	1	1	467	52.7	6.06
E5RGE1	Uncharacterized protein OS=Homo sapiens GN=YWHAZ PE=4 SV=1 - [E5RGE1_HUMAN]	17.31	8	1	1	52	5.9	4.78
E2QRK7	Uncharacterized protein OS=Homo sapiens GN=TJAP1 PE=4 SV=1 - [E2QRK7_HUMAN]	3.11	8	1	1	193	22.8	7.03
E9PKK9	Uncharacterized protein OS=Homo sapiens GN=DKK3 PE=4 SV=1 - [E9PKK9_HUMAN]	10.61	11	1	1	132	14.3	4.70
Q7KZ00	Endothelin receptor subtype B1 (Fragment) OS=Homo sapiens GN=ETB1 PE=2 SV=1 - [Q7KZ00_HUMAN]	35.29	1	1	1	17	2.1	9.70
Q9UL82	Myosin-reactive immunoglobulin light chain variable region (Fragment) OS=Homo sapiens PE=2 SV=1 - [Q9UL82_HUMAN]	10.28	1	1	1	107	11.4	6.60
B4E3I0	cDNA FLJ55017, highly similar to Caldesmon OS=Homo sapiens PE=2 SV=1 - [B4E3I0_HUMAN]	3.85	10	1	1	312	35.4	9.52
B4E3I2	cDNA FLJ55294, highly similar to Jumonji/ARID domain-containing protein 1C OS=Homo sapiens PE=2 SV=1 - [B4E3I2_HUMAN]	1.16	4	1	1	1,379	154.9	5.58
B7Z5K2	cDNA FLJ54289, highly similar to Neural cell adhesion molecule 2 OS=Homo sapiens PE=2 SV=1 - [B7Z5K2_HUMAN]	3.60	3	1	1	695	77.2	5.90
C9J8R9	Uncharacterized protein OS=Homo sapiens GN=SKIL PE=4 SV=1 - [C9J8R9_HUMAN]	13.01	3	1	1	123	13.3	7.20
B4DV06	cDNA FLJ55904, highly similar to Homo sapiens leucine rich repeat and coiled-coil domain containing 1 (LRRCC1), mRNA (Fragment) OS=Homo sapiens PE=2 SV=1 - [B4DV06_HUMAN]	1.60	4	1	1	751	87.4	6.24
Q3SXP2	VMO1 protein OS=Homo sapiens GN=VMO1 PE=2 SV=1 - [Q3SXP2_HUMAN]	17.88	2	1	1	151	16.1	5.08
B7Z952	cDNA FLJ54827, highly similar to Alpha-parvin OS=Homo sapiens PE=2 SV=1 - [B7Z952_HUMAN]	6.55	2	1	1	290	32.7	6.27
B4E2R9	cDNA FLJ52054, highly similar to Alcohol dehydrogenase 1B (EC 1.1.1.1) OS=Homo sapiens PE=2 SV=1 - [B4E2R9_HUMAN]	7.72	1	1	1	298	31.4	8.03
Q29RW5	ADAMTS8 protein (Fragment) OS=Homo sapiens GN=ADAMTS8 PE=2 SV=1 - [Q29RW5_HUMAN]	3.99	1	1	1	676	74.1	6.68
B4DSV7	cDNA FLJ60143, highly similar to Homo sapiens dystrophin, transcript variant Dp140bc, mRNA OS=Homo sapiens PE=2 SV=1 - [B4DSV7_HUMAN]	3.81	14	1	1	525	59.7	7.12
C9JM01	Uncharacterized protein OS=Homo sapiens GN=FKBP6 PE=4 SV=1 - [C9JM01_HUMAN]	3.17	4	1	1	252	28.5	7.24
Q8N6V2	UBASH3A protein OS=Homo sapiens PE=2 SV=1 - [Q8N6V2_HUMAN]	4.43	2	1	1	451	50.4	7.46
E7EMC7	Uncharacterized protein OS=Homo sapiens GN=SQSTM1 PE=4 SV=1 - [E7EMC7_HUMAN]	2.91	1	1	1	378	41.0	7.52
Q68DS9	Putative uncharacterized protein DKFZp686O23186 OS=Homo sapiens GN=DKFZp686O23186 PE=2 SV=1 - [Q68DS9_HUMAN]	9.70	5	1	1	134	14.3	8.48
A1L4E9	Zinc finger protein 572 OS=Homo sapiens GN=ZNF572 PE=2 SV=1 - [A1L4E9_HUMAN]	5.10	2	1	1	529	61.3	7.93
Q0VGA5	SARS protein OS=Homo sapiens GN=SARS PE=2 SV=1 - [Q0VGA5_HUMAN]	1.57	4	1	1	511	58.4	6.43
C9JCN8	Uncharacterized protein OS=Homo sapiens GN=FOSL2 PE=3 SV=1 - [C9JCN8_HUMAN]	5.94	5	1	1	202	22.2	8.78
C9JBN1	Uncharacterized protein OS=Homo sapiens GN=TBL1XR1 PE=4 SV=1 - [C9JBN1_HUMAN]	47.37	2	1	1	76	7.9	4.78
B4DGV8	cDNA FLJ54286, highly similar to Mus musculus membrane-associated ring finger (C3HC4) 5 (March5), mRNA OS=Homo sapiens PE=2 SV=1 - [B4DGV8_HUMAN]	13.79	1	1	1	174	19.4	9.54
B4DHU4	cDNA FLJ56970, highly similar to Endothelin-converting enzyme 2 (EC 3.4.24.71) OS=Homo sapiens PE=2 SV=1 - [B4DHU4_HUMAN]	3.71	3	1	1	485	55.3	5.08
A8MPQ1	Uncharacterized protein OS=Homo sapiens GN=HIF3A PE=4 SV=2 - [A8MPQ1_HUMAN]	2.95	7	1	1	576	62.6	5.96
B3KSD8	cDNA FLJ36057 fis, clone TESTI2018475, highly similar to LAMININ ALPHA-1 CHAIN OS=Homo sapiens PE=2 SV=1 - [B3KSD8_HUMAN]	3.70	1	1	1	405	44.0	8.63
Q8NCE6	cDNA FLJ90299 fis, clone NT2RP2000514, highly similar to Homo sapiens roundabout 2 (robo2) mRNA OS=Homo sapiens PE=2 SV=1 - [Q8NCE6_HUMAN]	2.98	5	1	1	570	62.1	7.11
Q7Z5X3	EIF3L protein OS=Homo sapiens GN=EIF3L PE=2 SV=2 - [Q7Z5X3_HUMAN]	8.51	9	1	1	188	22.3	8.51
Q5DVP9	Rhesus blood group CcEe antigene (Fragment) OS=Homo sapiens GN=RHCE PE=2 SV=1 - [Q5DVP9_HUMAN]	42.00	1	1	1	50	5.2	9.00
Q53S35	Putative uncharacterized protein SLC4A5 (Fragment) OS=Homo sapiens GN=SLC4A5 PE=2 SV=1 - [Q53S35_HUMAN]	2.66	3	1	1	714	78.6	8.31
B7ZAY9	cDNA, FLJ79353, highly similar to Cysteine protease ATG4D (EC 3.4.22.-) OS=Homo sapiens PE=2 SV=1 - [B7ZAY9_HUMAN]	3.21	3	1	1	280	30.9	10.10
B9DI81	Phospholipase C, eta 2 OS=Homo sapiens GN=PLCH2 PE=4 SV=1 - [B9DI81_HUMAN]	1.89	4	1	1	1,058	115.4	7.99
Q0ZCH6	Immunglobulin heavy chain variable region (Fragment) OS=Homo sapiens PE=4 SV=1 - [Q0ZCH6_HUMAN]	6.87	1	1	1	131	14.3	6.55
A8K1F4	cDNA FLJ78094, highly similar to Homo sapiens myeloid leukemia factor 2, mRNA OS=Homo sapiens PE=2 SV=1 - [A8K1F4_HUMAN]	10.89	2	1	1	248	28.1	6.90
A8K5N3	cDNA FLJ77545, highly similar to Homo sapiens zinc finger, DHHC-type containing 13, transcript variant 1, mRNA OS=Homo sapiens PE=2 SV=1 - [A8K5N3_HUMAN]	3.38	3	1	1	622	70.8	8.06
Q9H4N4	Homo sapiens clone CDABP0082 mRNA sequence OS=Homo sapiens PE=2 SV=1 - [Q9H4N4_HUMAN]	21.70	6	1	1	106	13.2	8.65
Q5JUB0	Vacuolar protein sorting 16 homolog (S. cerevisiae) (Fragment) OS=Homo sapiens GN=VPS16 PE=2 SV=1 - [Q5JUB0_HUMAN]	7.05	2	1	1	227	25.1	6.62
B4DWJ6	cDNA FLJ60359, highly similar to Ubiquitin carboxyl-terminal hydrolase 3 (EC 3.1.2.15) OS=Homo sapiens PE=2 SV=1 - [B4DWJ6_HUMAN]	5.90	8	1	1	271	30.9	7.46
B4DRN5	cDNA FLJ59398, highly similar to Latent-transforming growth factor beta-binding protein 3 OS=Homo sapiens PE=2 SV=1 - [B4DRN5_HUMAN]	1.87	4	1	1	1,018	109.2	5.63
B4E1R0	cDNA FLJ56201, highly similar to YY1-associated protein 1 OS=Homo sapiens PE=2 SV=1 - [B4E1R0_HUMAN]	2.01	5	1	1	596	65.3	8.57
C9JM93	Uncharacterized protein OS=Homo sapiens GN=RAD9B PE=4 SV=1 - [C9JM93_HUMAN]	16.46	5	1	1	79	9.5	8.76
E7EQB5	Uncharacterized protein OS=Homo sapiens GN=LHCGR PE=4 SV=1 - [E7EQB5_HUMAN]	1.85	2	1	1	325	36.2	8.73
Q8N7P9	cDNA FLJ40515 fis, clone TESTI2046552 OS=Homo sapiens PE=2 SV=1 - [Q8N7P9_HUMAN]	3.68	5	1	1	163	17.6	9.50
B7Z6N3	cDNA FLJ57598, highly similar to Homo sapiens golgi phosphoprotein 3-like (GOLPH3L), mRNA OS=Homo sapiens PE=2 SV=1 - [B7Z6N3_HUMAN]	6.22	1	1	1	241	27.6	5.19
B7Z1U0	cDNA FLJ58754, highly similar to Homo sapiens cyclin M4 (CNNM4), mRNA OS=Homo sapiens PE=2 SV=1 - [B7Z1U0_HUMAN]	2.66	3	1	1	263	29.5	9.03
Q8TEL2	FLJ00181 protein (Fragment) OS=Homo sapiens GN=FLJ00181 PE=2 SV=1 - [Q8TEL2_HUMAN]	1.63	3	1	1	368	41.5	7.96
D3YTG0	Uncharacterized protein OS=Homo sapiens GN=SEZ6 PE=4 SV=1 - [D3YTG0_HUMAN]	2.01	2	1	1	993	107.3	5.33
E9PKF5	Uncharacterized protein OS=Homo sapiens GN=ROM1 PE=4 SV=1 - [E9PKF5_HUMAN]	31.03	3	1	1	58	6.1	8.69
E5RG39	Uncharacterized protein OS=Homo sapiens GN=ZNF696 PE=4 SV=1 - [E5RG39_HUMAN]	5.39	2	1	1	204	21.0	7.71
B7ZBC9	Katanin p60 (ATPase-containing) subunit A 1 (Fragment) OS=Homo sapiens GN=KATNA1 PE=4 SV=1 - [B7ZBC9_HUMAN]	11.37	4	1	1	211	24.3	8.76
B8ZZI9	Uncharacterized protein OS=Homo sapiens GN=AFTPH PE=4 SV=1 - [B8ZZI9_HUMAN]	2.99	3	1	1	568	61.5	4.54
C9J1B5	Uncharacterized protein OS=Homo sapiens GN=GOLGA6B PE=4 SV=1 - [C9J1B5_HUMAN]	1.60	6	1	1	561	65.5	6.29
Q5JSZ8	KIAA0515 (Fragment) OS=Homo sapiens GN=KIAA0515 PE=2 SV=1 - [Q5JSZ8_HUMAN]	2.90	2	1	1	587	63.2	6.73
B4E0S6	cDNA FLJ55635, highly similar to pre-mRNA-splicing factorATP-dependent RNA helicase DHX15 (EC 3.6.1.-) OS=Homo sapiens PE=2 SV=1 - [B4E0S6_HUMAN]	1.66	2	1	1	784	89.5	7.46
C9IYF0	Uncharacterized protein OS=Homo sapiens GN=FBXO11 PE=4 SV=1 - [C9IYF0_HUMAN]	14.29	2	1	1	140	15.9	8.44
Q9P0P1	HSPC237 OS=Homo sapiens PE=2 SV=1 - [Q9P0P1_HUMAN]	3.55	4	1	1	169	18.3	7.87
Q8NFP3	Gag protein OS=Homo sapiens PE=4 SV=1 - [Q8NFP3_HUMAN]	3.70	1	1	1	622	71.7	8.84
Q59HA1	Golgin 97 variant (Fragment) OS=Homo sapiens PE=2 SV=1 - [Q59HA1_HUMAN]	2.50	3	1	1	400	46.5	4.97
Q6FI97	BAF53A protein OS=Homo sapiens GN=BAF53A PE=2 SV=1 - [Q6FI97_HUMAN]	4.43	1	1	1	429	47.4	5.72
O95074	Bax epsilon (Fragment) OS=Homo sapiens PE=2 SV=1 - [O95074_HUMAN]	30.00	1	1	1	40	4.6	10.27
Q2VYF0	Mitochondrial NADH oxidoreductase-like protein OS=Homo sapiens PE=2 SV=1 - [Q2VYF0_HUMAN]	8.04	2	1	1	112	12.5	9.31
A4D2J8	KIAA1068 protein OS=Homo sapiens GN=KIAA1068 PE=4 SV=1 - [A4D2J8_HUMAN]	10.00	1	1	1	220	24.7	5.35
B0QZ34	AT rich interactive domain 4B (RBP1-like) (Fragment) OS=Homo sapiens GN=ARID4B PE=4 SV=1 - [B0QZ34_HUMAN]	1.28	2	1	1	548	60.9	6.10
B3KUG6	cDNA FLJ39849 fis, clone SPLEN2014711, highly similar to Homo sapiens hect domain and RLD 6 (HERC6), transcript variant 1, mRNA OS=Homo sapiens PE=2 SV=1 - [B3KUG6_HUMAN]	1.03	2	1	1	584	68.0	7.84
Q7Z3N1	Putative uncharacterized protein DKFZp686A04130 OS=Homo sapiens GN=DKFZp686A04130 PE=2 SV=1 - [Q7Z3N1_HUMAN]	2.50	1	1	1	681	75.4	6.77
B0FTY2	NudC-like protein OS=Homo sapiens PE=2 SV=1 - [B0FTY2_HUMAN]	1.66	12	1	1	361	40.8	5.20
E9PJV1	Uncharacterized protein OS=Homo sapiens GN=GLYATL1 PE=4 SV=1 - [E9PJV1_HUMAN]	10.29	6	1	1	68	7.9	6.79
B1APG1	Protein kinase, cAMP-dependent, catalytic, beta (Fragment) OS=Homo sapiens GN=PRKACB PE=4 SV=1 - [B1APG1_HUMAN]	12.69	12	1	1	197	23.1	9.23
B4DDG9	cDNA FLJ53856, highly similar to 5-aminolevulinate synthase, nonspecific, mitochondrial (EC 2.3.1.37) OS=Homo sapiens PE=2 SV=1 - [B4DDG9_HUMAN]	2.27	3	1	1	440	48.8	8.35
Q9BTG7	SPHK1 protein (Fragment) OS=Homo sapiens GN=SPHK1 PE=2 SV=2 - [Q9BTG7_HUMAN]	5.92	5	1	1	287	31.7	6.79
Q16408	UDP-N-acetylglucosamine: alpha-6-D-mannoside beta-1,6-N-acetylglucosaminyltransferase V|GlcNAc transferase V protein (Fragment) OS=Homo sapiens PE=4 SV=1 - [Q16408_HUMAN]	84.21	1	1	1	19	2.1	6.46
Q3KZ26	Dombrock blood group carrier molecule (Fragment) OS=Homo sapiens GN=DO PE=2 SV=1 - [Q3KZ26_HUMAN]	48.28	11	1	1	29	3.3	9.74
Q9UBJ2	ATP-binding cassette sub-family D member 2 OS=Homo sapiens GN=ABCD2 PE=1 SV=1 - [ABCD2_HUMAN]	2.03	1	1	1	740	83.2	8.92
Q9P0P8	Uncharacterized protein C6orf203 OS=Homo sapiens GN=C6orf203 PE=1 SV=1 - [CF203_HUMAN]	5.42	1	1	1	240	27.9	9.29
Q9NXG0	Centlein OS=Homo sapiens GN=CNTLN PE=2 SV=5 - [CNTLN_HUMAN]	2.21	2	1	1	1,405	161.5	8.15
Q17RW2	Collagen alpha-1(XXIV) chain OS=Homo sapiens GN=COL24A1 PE=2 SV=2 - [COOA1_HUMAN]	1.28	1	1	1	1,714	175.4	8.27
Q8N144	Gap junction delta-3 protein OS=Homo sapiens GN=GJD3 PE=1 SV=1 - [CXD3_HUMAN]	6.12	2	1	1	294	31.9	8.54
Q9Y295	Developmentally-regulated GTP-binding protein 1 OS=Homo sapiens GN=DRG1 PE=1 SV=1 - [DRG1_HUMAN]	3.00	1	1	1	367	40.5	8.90
P30040	Endoplasmic reticulum resident protein 29 OS=Homo sapiens GN=ERP29 PE=1 SV=4 - [ERP29_HUMAN]	3.83	1	1	1	261	29.0	7.31
Q8TAL6	Fin bud initiation factor homolog OS=Homo sapiens GN=FIBIN PE=2 SV=1 - [FIBIN_HUMAN]	8.53	1	1	1	211	24.3	5.49
Q5SZK8	FRAS1-related extracellular matrix protein 2 OS=Homo sapiens GN=FREM2 PE=1 SV=2 - [FREM2_HUMAN]	1.07	1	1	1	3,169	350.9	5.03
Q5SYB0	FERM and PDZ domain-containing protein 1 OS=Homo sapiens GN=FRMPD1 PE=1 SV=1 - [FRPD1_HUMAN]	0.57	1	1	1	1,578	173.3	5.25
O14610	Guanine nucleotide-binding protein G(I)/G(S)/G(O) subunit gamma-T2 OS=Homo sapiens GN=GNGT2 PE=2 SV=1 - [GBGT2_HUMAN]	11.59	2	1	1	69	7.7	6.68
Q9NVX0	HAUS augmin-like complex subunit 2 OS=Homo sapiens GN=HAUS2 PE=1 SV=1 - [HAUS2_HUMAN]	3.40	1	1	1	235	26.9	7.88
Q9NU23	LYR motif-containing protein 2 OS=Homo sapiens GN=LYRM2 PE=1 SV=1 - [LYRM2_HUMAN]	22.73	1	1	1	88	10.4	10.46
A6NNC1	Putative POM121-like protein 1-like OS=Homo sapiens PE=5 SV=3 - [P12LL_HUMAN]	2.12	1	1	1	897	94.0	10.26
Q86VP3	Phosphofurin acidic cluster sorting protein 2 OS=Homo sapiens GN=PACS2 PE=1 SV=3 - [PACS2_HUMAN]	1.01	2	1	1	889	97.6	6.60
Q16822	Phosphoenolpyruvate carboxykinase [GTP], mitochondrial OS=Homo sapiens GN=PCK2 PE=1 SV=3 - [PCKGM_HUMAN]	2.34	2	1	1	640	70.7	7.62
P35251	Replication factor C subunit 1 OS=Homo sapiens GN=RFC1 PE=1 SV=4 - [RFC1_HUMAN]	1.57	1	1	1	1,148	128.2	9.36
A2VEC9	SCO-spondin OS=Homo sapiens GN=SSPO PE=2 SV=1 - [SSPO_HUMAN]	0.17	1	1	1	5,147	547.1	6.02
Q8TCJ2	Dolichyl-diphosphooligosaccharide--protein glycosyltransferase subunit STT3B OS=Homo sapiens GN=STT3B PE=1 SV=1 - [STT3B_HUMAN]	0.85	1	1	1	826	93.6	8.91
A6NJI9	Leucine-rich repeat-containing protein ENSP00000371558 OS=Homo sapiens PE=2 SV=2 - [YG043_HUMAN]	2.09	1	1	1	287	33.6	8.76
Q9Y6Q3	Zinc finger protein 37 homolog OS=Homo sapiens GN=ZFP37 PE=2 SV=3 - [ZFP37_HUMAN]	2.86	1	1	1	630	71.2	9.09
Q99592	Zinc finger protein 238 OS=Homo sapiens GN=ZNF238 PE=1 SV=1 - [ZN238_HUMAN]	2.49	1	1	1	522	58.3	5.69
Q96N95	Zinc finger protein 396 OS=Homo sapiens GN=ZNF396 PE=1 SV=2 - [ZN396_HUMAN]	5.67	1	1	1	335	38.6	8.05
A8K8V0	Zinc finger protein 785 OS=Homo sapiens GN=ZNF785 PE=2 SV=1 - [ZN785_HUMAN]	4.69	1	1	1	405	46.1	9.01
Q3KNS6	Zinc finger protein 829 OS=Homo sapiens GN=ZNF829 PE=2 SV=1 - [ZN829_HUMAN]	2.55	1	1	1	432	50.1	7.93
Q6ZTB9	Putative zinc finger protein 833 OS=Homo sapiens GN=ZNF833P PE=5 SV=1 - [ZN833_HUMAN]	3.21	1	1	1	187	21.7	8.60
Q9HCJ5	Zinc finger SWIM domain-containing protein 6 OS=Homo sapiens GN=ZSWIM6 PE=2 SV=2 - [ZSWM6_HUMAN]	0.49	1	1	1	1,215	133.4	7.36
Q3LSM7	NEDD4L variant OS=Homo sapiens GN=NEDD4L PE=2 SV=1 - [Q3LSM7_HUMAN]	2.04	4	1	1	834	96.2	6.04
Q5VZY9	Doublecortin and CaM kinase-like 1 OS=Homo sapiens GN=DCAMKL1 PE=2 SV=1 - [Q5VZY9_HUMAN]	1.65	6	1	1	363	40.4	9.67
Q5SQM6	Nudix (Nucleoside diphosphate linked moiety X)-type motif 13 OS=Homo sapiens GN=NUDT13 PE=2 SV=1 - [Q5SQM6_HUMAN]	2.55	3	1	1	235	26.9	9.09
B1AJP6	Glypican 3 (Fragment) OS=Homo sapiens GN=GPC3 PE=3 SV=1 - [B1AJP6_HUMAN]	2.75	6	1	1	255	29.0	6.86
Q7Z2H1	Oligophrenin 1 (Fragment) OS=Homo sapiens GN=OPHN1 PE=2 SV=1 - [Q7Z2H1_HUMAN]	35.85	3	1	1	53	6.0	5.71
Q96I65	EIF4G1 protein OS=Homo sapiens GN=EIF4G1 PE=2 SV=1 - [Q96I65_HUMAN]	1.55	18	1	1	645	72.2	6.34
Q0VD99	GLS2 protein (Fragment) OS=Homo sapiens GN=GLS2 PE=2 SV=1 - [Q0VD99_HUMAN]	2.36	5	1	1	254	28.0	8.95
B7Z601	cDNA FLJ57187, highly similar to Glycerol-3-phosphate dehydrogenase, mitochondrial (EC 1.1.99.5) OS=Homo sapiens PE=2 SV=1 - [B7Z601_HUMAN]	1.20	4	1	1	500	55.7	6.20
B4DIT7	cDNA FLJ58187, highly similar to Protein-glutamine gamma-glutamyltransferase 2(EC 2.3.2.13) OS=Homo sapiens PE=2 SV=1 - [B4DIT7_HUMAN]	1.16	3	1	1	606	68.6	5.30
B4DKR7	cDNA FLJ50563 OS=Homo sapiens PE=4 SV=1 - [B4DKR7_HUMAN]	7.62	3	1	1	105	12.0	6.04
E7EP58	Uncharacterized protein OS=Homo sapiens GN=ADAMTS7 PE=4 SV=1 - [E7EP58_HUMAN]	2.17	5	1	1	460	50.7	7.69
A5PLK7	RCC2 protein (Fragment) OS=Homo sapiens GN=RCC2 PE=2 SV=1 - [A5PLK7_HUMAN]	3.50	2	1	1	457	49.6	8.69
B2R5U7	cDNA, FLJ92633, highly similar to Homo sapiens CCAAT-box-binding transcription factor (CBF2), mRNA OS=Homo sapiens PE=2 SV=1 - [B2R5U7_HUMAN]	1.10	2	1	1	998	114.1	5.36
A4UCT9	N-myc-interactor (Fragment) OS=Homo sapiens PE=2 SV=1 - [A4UCT9_HUMAN]	6.67	5	1	1	120	13.8	4.96
D9J0A2	Pitchfork OS=Homo sapiens PE=2 SV=1 - [D9J0A2_HUMAN]	3.14	4	1	1	191	21.9	9.89
A0PJJ5	SRP72 protein (Fragment) OS=Homo sapiens GN=SRP72 PE=2 SV=1 - [A0PJJ5_HUMAN]	1.07	6	1	1	559	62.6	8.32
B7Z4Y1	cDNA FLJ58031, highly similar to Tuftelin OS=Homo sapiens PE=2 SV=1 - [B7Z4Y1_HUMAN]	3.18	1	1	1	409	46.4	6.13
Q5JC44	KLHL9 protein OS=Homo sapiens PE=2 SV=1 - [Q5JC44_HUMAN]	3.08	1	1	1	617	69.4	6.35
Q96P23	Myofibrillogenesis regulator MR-2 OS=Homo sapiens PE=2 SV=1 - [Q96P23_HUMAN]	4.93	1	1	1	142	15.6	10.27
Q9UKZ7	Transcription cofactor vestigial-like protein 3. OS=Homo sapiens PE=2 SV=1 - [Q9UKZ7_HUMAN]	3.83	1	1	1	392	45.2	8.24
O95226	Voltage-gated L-type calcium channel alpha-1 subunit OS=Homo sapiens GN=CACNA1F PE=2 SV=1 - [O95226_HUMAN]	0.68	2	1	1	1,912	213.9	5.92
D7RX09	Desmoplakin Ia OS=Homo sapiens PE=2 SV=1 - [D7RX09_HUMAN]	0.33	2	1	1	2,428	278.7	6.95
B4DM46	cDNA FLJ58909 OS=Homo sapiens PE=2 SV=1 - [B4DM46_HUMAN]	3.11	2	1	1	225	21.9	12.18
D6RF16	Uncharacterized protein OS=Homo sapiens GN=GAK PE=4 SV=1 - [D6RF16_HUMAN]	46.94	10	1	1	49	5.1	5.11
B3KVJ5	cDNA FLJ16648 fis, clone TESTI4035508, highly similar to Structural maintenance of chromosomes 4-like 1 protein OS=Homo sapiens PE=2 SV=1 - [B3KVJ5_HUMAN]	1.93	4	1	1	883	101.0	7.08
B9EIS6	Olfactory receptor, family 4, subfamily K, member 5 OS=Homo sapiens GN=OR4K5 PE=2 SV=1 - [B9EIS6_HUMAN]	2.79	1	1	1	323	36.2	8.44
B7Z5N7	cDNA FLJ58612, highly similar to Sec1 family domain-containing protein 1 OS=Homo sapiens PE=2 SV=1 - [B7Z5N7_HUMAN]	2.19	6	1	1	457	51.5	6.96
B3KMK3	cDNA FLJ11241 fis, clone PLACE1008603, highly similar to Nuclear pore complex protein Nup155 OS=Homo sapiens PE=2 SV=1 - [B3KMK3_HUMAN]	1.57	4	1	1	382	44.0	6.40
B3KSJ1	cDNA FLJ36398 fis, clone THYMU2009592, highly similar to Homo sapiens nuclear prelamin A recognition factor (NARF), transcript variant 1, mRNA OS=Homo sapiens PE=2 SV=1 - [B3KSJ1_HUMAN]	4.23	5	1	1	189	21.6	7.27
C9JH18	Uncharacterized protein OS=Homo sapiens GN=GIGYF2 PE=4 SV=1 - [C9JH18_HUMAN]	17.45	5	1	1	149	16.8	9.79
C9K017	Uncharacterized protein OS=Homo sapiens PE=3 SV=1 - [C9K017_HUMAN]	4.61	2	1	1	217	24.6	5.99
Q1M186	HMGA2d OS=Homo sapiens GN=HMGA2 PE=2 SV=1 - [Q1M186_HUMAN]	6.52	1	1	1	92	10.1	11.88
B9EK39	TANC1 protein OS=Homo sapiens GN=TANC1 PE=2 SV=1 - [B9EK39_HUMAN]	0.58	2	1	1	1,390	152.1	8.34
B4DWQ5	cDNA FLJ51655, highly similar to Actin-like protein 2 OS=Homo sapiens PE=2 SV=1 - [B4DWQ5_HUMAN]	2.30	1	1	1	305	34.5	5.72
B7Z8R2	cDNA FLJ55874, highly similar to Homo sapiens pentatricopeptide repeat domain 2 (PTCD2), mRNA OS=Homo sapiens PE=2 SV=1 - [B7Z8R2_HUMAN]	9.94	2	1	1	161	18.3	9.17
D6REX6	Uncharacterized protein OS=Homo sapiens GN=FAM153A PE=4 SV=1 - [D6REX6_HUMAN]	4.65	10	1	1	129	14.7	8.12
Q16316	Steroid 18-hydroxylase (Fragment) OS=Homo sapiens GN=CYP11B2 PE=2 SV=1 - [Q16316_HUMAN]	3.95	2	1	1	228	25.6	6.87
B6E949	Glycosyltransferase A (Fragment) OS=Homo sapiens GN=ABO PE=4 SV=1 - [B6E949_HUMAN]	20.00	5	1	1	30	3.7	7.52
B4DQD7	cDNA FLJ51043, highly similar to Oviduct-specific glycoprotein OS=Homo sapiens PE=2 SV=1 - [B4DQD7_HUMAN]	1.23	5	1	1	486	53.2	8.21
B4DNL7	cDNA FLJ60737, highly similar to Cysteine desulfurase, mitochondrial (EC 2.8.1.7) OS=Homo sapiens PE=2 SV=1 - [B4DNL7_HUMAN]	10.98	6	1	1	255	28.2	7.49
E5RG19	Uncharacterized protein OS=Homo sapiens GN=TMEM30A PE=4 SV=1 - [E5RG19_HUMAN]	22.08	4	1	1	77	8.8	5.34
C9JY10	Uncharacterized protein OS=Homo sapiens GN=PDIA5 PE=4 SV=1 - [C9JY10_HUMAN]	10.94	3	1	1	64	7.3	6.29
Q8TBJ6	ST6GALNAC1 protein OS=Homo sapiens GN=ST6GALNAC1 PE=2 SV=1 - [Q8TBJ6_HUMAN]	4.67	1	1	1	471	52.1	10.04
B4DYP7	cDNA FLJ55435, highly similar to Gamma-tubulin complex component 3 OS=Homo sapiens PE=2 SV=1 - [B4DYP7_HUMAN]	2.01	2	1	1	897	102.5	8.22
B3KUL6	cDNA FLJ40185 fis, clone TESTI2018565, highly similar to Homo sapiens AN1, ubiquitin-like, homolog (Xenopus laevis) (ANUBL1), mRNA OS=Homo sapiens PE=2 SV=1 - [B3KUL6_HUMAN]	1.31	5	1	1	609	66.5	8.56
B9A6M9	TBC1 domain family, member 30 OS=Homo sapiens GN=TBC1D30 PE=2 SV=1 - [B9A6M9_HUMAN]	1.05	2	1	1	761	84.6	8.75
B7Z5I6	cDNA FLJ56516, highly similar to Nuclear pore complex protein Nup88 OS=Homo sapiens PE=2 SV=1 - [B7Z5I6_HUMAN]	4.13	3	1	1	557	61.4	5.11
B3KQG9	cDNA FLJ90444 fis, clone NT2RP3001159, highly similar to Homo sapiens fatty acid desaturase 2 (FADS2), mRNA OS=Homo sapiens PE=2 SV=1 - [B3KQG9_HUMAN]	5.71	1	1	1	210	25.3	8.87
B3KM85	cDNA FLJ10518 fis, clone NT2RP2000814, highly similar to Abnormal spindle-like microcephaly-associated protein (Fragment) OS=Homo sapiens PE=2 SV=1 - [B3KM85_HUMAN]	3.23	5	1	1	526	62.6	10.07
B3KMD9	cDNA FLJ10758 fis, clone NT2RP3004594, highly similar to WD repeat and HMG-box DNA-binding protein 1 OS=Homo sapiens PE=2 SV=1 - [B3KMD9_HUMAN]	2.20	6	1	1	364	40.9	7.75
B7Z641	cDNA FLJ54439, highly similar to Vacuolar proton translocating ATPase 116 kDa subunit a isoform 1 OS=Homo sapiens PE=2 SV=1 - [B7Z641_HUMAN]	0.76	7	1	1	788	90.4	6.83
P31937	3-hydroxyisobutyrate dehydrogenase, mitochondrial OS=Homo sapiens GN=HIBADH PE=1 SV=2 - [3HIDH_HUMAN]	5.65	1	1	1	336	35.3	8.13
Q8N1D0	Beckwith-Wiedemann syndrome chromosomal region 1 candidate gene B protein OS=Homo sapiens GN=SLC22A18AS PE=2 SV=2 - [BWR1B_HUMAN]	6.72	1	1	1	253	27.2	9.79
Q8NFW1	Collagen alpha-1(XXII) chain OS=Homo sapiens GN=COL22A1 PE=1 SV=2 - [COMA1_HUMAN]	1.11	1	1	1	1,626	161.0	7.23
Q86XI8	Uncharacterized protein C19orf68 OS=Homo sapiens GN=C19orf68 PE=1 SV=2 - [CS068_HUMAN]	1.59	1	1	1	627	70.0	8.57
A2CJ06	Dystrotelin OS=Homo sapiens GN=DYTN PE=2 SV=1 - [DYTN_HUMAN]	2.77	1	1	1	578	65.3	9.00
P42126	Enoyl-CoA delta isomerase 1, mitochondrial OS=Homo sapiens GN=ECI1 PE=1 SV=1 - [ECI1_HUMAN]	2.65	1	1	1	302	32.8	8.54
P83110	Probable serine protease HTRA3 OS=Homo sapiens GN=HTRA3 PE=1 SV=2 - [HTRA3_HUMAN]	3.09	1	1	1	453	48.6	7.09
Q8N9C0	Immunoglobulin superfamily member 22 OS=Homo sapiens GN=IGSF22 PE=1 SV=2 - [IGS22_HUMAN]	1.44	2	1	1	903	100.3	7.12
Q6UWE0	E3 ubiquitin-protein ligase LRSAM1 OS=Homo sapiens GN=LRSAM1 PE=1 SV=1 - [LRSM1_HUMAN]	1.24	1	1	1	723	83.5	5.94
A7E2Y1	Myosin-7B OS=Homo sapiens GN=MYH7B PE=2 SV=3 - [MYH7B_HUMAN]	0.98	1	1	1	1,941	221.3	6.01
Q86W25	NACHT, LRR and PYD domains-containing protein 13 OS=Homo sapiens GN=NLRP13 PE=1 SV=2 - [NAL13_HUMAN]	1.63	1	1	1	1,043	118.8	5.66
P46531	Neurogenic locus notch homolog protein 1 OS=Homo sapiens GN=NOTCH1 PE=1 SV=4 - [NOTC1_HUMAN]	1.21	1	1	1	2,555	272.3	5.12
Q8NGC2	Olfactory receptor 4E2 OS=Homo sapiens GN=OR4E2 PE=2 SV=1 - [OR4E2_HUMAN]	2.88	1	1	1	313	35.4	7.97
Q8N807	Protein disulfide-isomerase-like protein of the testis OS=Homo sapiens GN=PDILT PE=1 SV=2 - [PDILT_HUMAN]	1.20	1	1	1	584	66.6	6.86
Q9Y4D7	Plexin-D1 OS=Homo sapiens GN=PLXND1 PE=1 SV=3 - [PLXD1_HUMAN]	1.35	1	1	1	1,925	211.9	7.15
P98175	RNA-binding protein 10 OS=Homo sapiens GN=RBM10 PE=1 SV=3 - [RBM10_HUMAN]	1.51	2	1	1	930	103.5	5.97
P61576	HERV-K_5q13.3 provirus Rec protein OS=Homo sapiens PE=1 SV=1 - [REC9_HUMAN]	15.24	1	1	1	105	11.7	9.92
P62851	40S ribosomal protein S25 OS=Homo sapiens GN=RPS25 PE=1 SV=1 - [RS25_HUMAN]	7.20	1	1	1	125	13.7	10.11
O95977	Sphingosine 1-phosphate receptor 4 OS=Homo sapiens GN=S1PR4 PE=1 SV=1 - [S1PR4_HUMAN]	5.73	1	1	1	384	41.6	9.96
Q9H5K3	Protein kinase-like protein SgK196 OS=Homo sapiens GN=SGK196 PE=2 SV=1 - [SG196_HUMAN]	1.71	1	1	1	350	40.0	6.10
Q9UHP9	Small muscular protein OS=Homo sapiens GN=SMPX PE=2 SV=3 - [SMPX_HUMAN]	7.95	1	1	1	88	9.6	9.16
Q9BQP9	Short palate, lung and nasal epithelium carcinoma-associated protein 3 OS=Homo sapiens GN=SPLUNC3 PE=2 SV=3 - [SPLC3_HUMAN]	7.87	1	1	1	254	28.4	6.65
Q8WUH2	Transforming growth factor-beta receptor-associated protein 1 OS=Homo sapiens GN=TGFBRAP1 PE=1 SV=1 - [TGFA1_HUMAN]	0.81	1	1	1	860	97.1	6.55
Q8NB66	Protein unc-13 homolog C OS=Homo sapiens GN=UNC13C PE=1 SV=3 - [UN13C_HUMAN]	0.50	1	1	1	2,214	250.8	5.92
O75132	Zinc finger BED domain-containing protein 4 OS=Homo sapiens GN=ZBED4 PE=1 SV=2 - [ZBED4_HUMAN]	2.22	1	1	1	1,171	130.2	6.83
B2R8X8	SLAM family member 6, isoform CRA_c OS=Homo sapiens GN=SLAMF6 PE=2 SV=1 - [B2R8X8_HUMAN]	6.65	2	1	1	331	37.3	6.79
Q5STZ7	ATP-binding cassette sub-family F (GCN20) member 1 OS=Homo sapiens GN=ABCF1 PE=2 SV=2 - [Q5STZ7_HUMAN]	6.72	6	1	1	238	27.2	5.68
B9A012	Myosin VIIA, isoform CRA_a OS=Homo sapiens GN=MYO7A PE=4 SV=1 - [B9A012_HUMAN]	0.59	3	1	1	1,178	135.7	8.63
Q7KYX9	PFKM protein OS=Homo sapiens GN=PFKM PE=2 SV=1 - [Q7KYX9_HUMAN]	2.58	5	1	1	465	51.0	8.82
A7E2F7	CAP-GLY domain containing linker protein 2 OS=Homo sapiens GN=CLIP2 PE=2 SV=1 - [A7E2F7_HUMAN]	0.89	2	1	1	1,011	111.7	6.76
B7Z979	cDNA FLJ54248, highly similar to Homo sapiens armadillo repeat containing 8 (ARMC8), transcript variant 2, mRNA OS=Homo sapiens PE=2 SV=1 - [B7Z979_HUMAN]	2.14	7	1	1	468	52.7	6.42
E5RJ19	Uncharacterized protein OS=Homo sapiens PE=4 SV=1 - [E5RJ19_HUMAN]	21.78	1	1	1	101	11.3	10.20
B3KV94	Jumonji, AT rich interactive domain 1B (RBP2-like), isoform CRA_a (Fragment) OS=Homo sapiens GN=JARID1B PE=2 SV=1 - [B3KV94_HUMAN]	1.18	2	1	1	1,275	145.0	6.99
A6NP15	Proteasome (Prosome, macropain) 26S subunit, non-ATPase, 12, isoform CRA_c OS=Homo sapiens GN=PSMD12 PE=2 SV=1 - [A6NP15_HUMAN]	1.38	3	1	1	436	50.5	7.88
Q5VVQ3	6-phosphofructo-2-kinase/fructose-2, 6-biphosphatase 2, isoform CRA_b OS=Homo sapiens GN=PFKFB2 PE=2 SV=1 - [Q5VVQ3_HUMAN]	4.88	2	1	1	471	54.4	7.56
D3DS14	Putative uncharacterized protein FLJ10357 OS=Homo sapiens GN=FLJ10357 PE=4 SV=1 - [D3DS14_HUMAN]	1.32	2	1	1	1,519	164.5	6.15
F1CIE7	ATP synthase F0 subunit 6 OS=Homo sapiens GN=ATP6 PE=4 SV=1 - [F1CIE7_HUMAN]	17.26	1	1	1	226	24.8	10.29
C9JLZ4	Uncharacterized protein OS=Homo sapiens GN=INO80D PE=4 SV=1 - [C9JLZ4_HUMAN]	1.59	2	1	1	439	49.6	9.17
D6RGZ3	Uncharacterized protein OS=Homo sapiens PE=4 SV=1 - [D6RGZ3_HUMAN]	2.92	9	1	1	651	73.8	6.29
Q71UD5	Uroporphyrinogen decarboxylase (Fragment) OS=Homo sapiens GN=UROD PE=2 SV=1 - [Q71UD5_HUMAN]	20.00	1	1	1	100	11.1	9.99
A8MY76	Uncharacterized protein OS=Homo sapiens GN=C14orf21 PE=4 SV=1 - [A8MY76_HUMAN]	5.42	3	1	1	535	58.1	6.73
E7EP39	Uncharacterized protein OS=Homo sapiens GN=CEP110 PE=4 SV=1 - [E7EP39_HUMAN]	1.03	5	1	1	875	101.9	5.62
B0UZE7	HLA-B associated transcript 3 (Fragment) OS=Homo sapiens GN=BAT3 PE=4 SV=1 - [B0UZE7_HUMAN]	12.59	7	1	1	135	15.4	9.52
B4DZZ1	cDNA FLJ57836, highly similar to Myb-binding protein 1A OS=Homo sapiens PE=2 SV=1 - [B4DZZ1_HUMAN]	1.00	4	1	1	603	67.5	9.72
B4DS99	cDNA FLJ51291, highly similar to FYVE, RhoGEF and PH domain-containing protein 1 OS=Homo sapiens PE=2 SV=1 - [B4DS99_HUMAN]	1.14	7	1	1	525	59.8	5.83
B3KR38	cDNA FLJ33624 fis, clone BRAMY2021962, highly similar to Homo sapiens diacylglycerol lipase beta OS=Homo sapiens PE=2 SV=1 - [B3KR38_HUMAN]	2.75	7	1	1	218	24.1	7.74
C9JTD3	Uncharacterized protein OS=Homo sapiens GN=STARD7 PE=4 SV=1 - [C9JTD3_HUMAN]	10.20	4	1	1	147	17.8	7.66
D6RA29	Uncharacterized protein OS=Homo sapiens GN=SPARCL1 PE=4 SV=1 - [D6RA29_HUMAN]	10.83	9	1	1	120	12.9	4.75
B4E2P6	cDNA FLJ50366, highly similar to Zinc finger protein 398 OS=Homo sapiens PE=2 SV=1 - [B4E2P6_HUMAN]	2.37	3	1	1	337	37.5	8.43
B3KQ98	cDNA FLJ90011 fis, clone HEMBA1000443, highly similar to Gastric cancer antigen Zg14 OS=Homo sapiens PE=4 SV=1 - [B3KQ98_HUMAN]	12.62	1	1	1	103	12.6	9.73
Q6ZP86	cDNA FLJ26299 fis, clone DMC07412 OS=Homo sapiens PE=2 SV=1 - [Q6ZP86_HUMAN]	10.69	8	1	1	159	17.8	8.12
E7EMC4	Uncharacterized protein OS=Homo sapiens GN=DDHD1 PE=4 SV=1 - [E7EMC4_HUMAN]	5.25	2	1	1	743	82.9	5.78
B3KV41	cDNA FLJ16101 fis, clone TESTI2014780, highly similar to Homo sapiens minichromosome maintenance deficient domain containing 1 (MCMDC1), mRNA OS=Homo sapiens PE=2 SV=1 - [B3KV41_HUMAN]	1.53	4	1	1	391	44.0	5.76
C9JRC4	Uncharacterized protein OS=Homo sapiens GN=PPP1R7 PE=4 SV=1 - [C9JRC4_HUMAN]	6.52	6	1	1	184	21.1	4.64
Q6PKE0	SON protein (Fragment) OS=Homo sapiens GN=SON PE=2 SV=1 - [Q6PKE0_HUMAN]	0.51	5	1	1	1,365	146.7	4.82
Q68CV8	Putative uncharacterized protein DKFZp762N1113 (Fragment) OS=Homo sapiens GN=DKFZp762N1113 PE=2 SV=1 - [Q68CV8_HUMAN]	7.17	4	1	1	237	27.6	4.94
B7Z6C7	cDNA FLJ54546, highly similar to Homo sapiens sarcolemma associated protein (SLMAP), mRNA OS=Homo sapiens PE=2 SV=1 - [B7Z6C7_HUMAN]	2.70	5	1	1	296	34.2	5.55
B2RDL6	cDNA, FLJ96669, highly similar to Homo sapiens secreted protein, acidic, cysteine-rich (osteonectin)(SPARC), mRNA OS=Homo sapiens PE=2 SV=1 - [B2RDL6_HUMAN]	5.28	1	1	1	303	34.6	4.87
Q7Z4C7	MSTP128 OS=Homo sapiens PE=1 SV=1 - [Q7Z4C7_HUMAN]	5.72	8	1	1	297	35.6	5.41
Q59FZ6	SWI/SNF-related matrix-associated actin-dependent regulator of chromatin a4 variant (Fragment) OS=Homo sapiens PE=2 SV=2 - [Q59FZ6_HUMAN]	2.15	9	1	1	1,165	129.6	9.16
Q96NG1	cDNA FLJ30950 fis, clone HCASM1000061 OS=Homo sapiens PE=2 SV=1 - [Q96NG1_HUMAN]	5.43	8	1	1	184	20.5	5.59
A8K8F9	cDNA FLJ75064, highly similar to Homo sapiens phospholipase C, delta 1 (PLCD1), mRNA OS=Homo sapiens PE=2 SV=1 - [A8K8F9_HUMAN]	1.32	5	1	1	756	85.6	6.70
B2R5C1	cDNA, FLJ92414, highly similar to Homo sapiens zinc finger protein 32 (KOX 30) (ZNF32), mRNA OS=Homo sapiens PE=2 SV=1 - [B2R5C1_HUMAN]	2.93	2	1	1	273	31.0	9.35
Q09LL8	DCLRE1A (Fragment) OS=Homo sapiens PE=2 SV=1 - [Q09LL8_HUMAN]	2.02	2	1	1	496	55.1	6.61
B7Z8J1	cDNA FLJ55657 OS=Homo sapiens PE=2 SV=1 - [B7Z8J1_HUMAN]	3.87	1	1	1	155	16.7	5.20
E7ER99	Uncharacterized protein OS=Homo sapiens GN=OBSL1 PE=4 SV=1 - [E7ER99_HUMAN]	4.30	4	1	1	442	47.4	5.14
B4DWN1	cDNA FLJ52285, highly similar to Vesicular integral-membrane protein VIP36 OS=Homo sapiens PE=2 SV=1 - [B4DWN1_HUMAN]	6.67	5	1	1	285	32.6	6.54
Q15098	APS protein (Fragment) OS=Homo sapiens GN=APS PE=2 SV=1 - [Q15098_HUMAN]	9.09	6	1	1	176	19.1	6.11
C9JI87	Uncharacterized protein OS=Homo sapiens GN=VDAC1 PE=4 SV=1 - [C9JI87_HUMAN]	10.33	3	1	1	184	20.4	7.33
B4DVE8	cDNA FLJ51334, highly similar to Ubiquitin carboxyl-terminal hydrolase 35 (EC 3.1.2.15) OS=Homo sapiens PE=2 SV=1 - [B4DVE8_HUMAN]	3.11	6	1	1	450	50.5	4.92
B3KP10	cDNA FLJ30907 fis, clone FEBRA2006092, highly similar to Homo sapiens DIX domain containing 1 (DIXDC1), mRNA OS=Homo sapiens PE=2 SV=1 - [B3KP10_HUMAN]	11.85	1	1	1	211	23.9	7.08
Q68VJ5	Polypeptide N-acetylgalactosaminyltransferase 5 (Fragment) OS=Homo sapiens GN=GALNT5 PE=2 SV=1 - [Q68VJ5_HUMAN]	7.10	2	1	1	169	19.6	8.72
Q05BU1	PKN3 protein (Fragment) OS=Homo sapiens GN=PKN3 PE=2 SV=1 - [Q05BU1_HUMAN]	3.33	2	1	1	300	33.2	9.98
B2R8R5	cDNA, FLJ94025, highly similar to Homo sapiens tripartite motif-containing 28 (TRIM28), mRNA OS=Homo sapiens PE=2 SV=1 - [B2R8R5_HUMAN]	0.96	2	1	1	835	88.5	5.77
B4DG65	cDNA FLJ52105, highly similar to PDZ domain-containing RING finger protein 4 OS=Homo sapiens PE=2 SV=1 - [B4DG65_HUMAN]	0.77	3	1	1	778	89.0	5.19
E7EPA6	Uncharacterized protein OS=Homo sapiens GN=LAMB1 PE=4 SV=1 - [E7EPA6_HUMAN]	1.29	2	1	1	850	95.1	5.44
E5RHC6	Uncharacterized protein OS=Homo sapiens GN=WDR67 PE=4 SV=1 - [E5RHC6_HUMAN]	13.01	1	1	1	146	16.7	10.26
Q53SQ6	Putative uncharacterized protein PTD004 (Fragment) OS=Homo sapiens GN=PTD004 PE=2 SV=1 - [Q53SQ6_HUMAN]	4.23	2	1	1	213	24.8	9.19
A2VCT1	LCOR protein (Fragment) OS=Homo sapiens GN=LCOR PE=2 SV=1 - [A2VCT1_HUMAN]	5.60	2	1	1	268	28.6	9.35
B4DR36	cDNA FLJ56414, highly similar to Homo sapiens proline-, glutamic acid-, leucine-rich protein 1 (PELP1), mRNA OS=Homo sapiens PE=2 SV=1 - [B4DR36_HUMAN]	0.76	1	1	1	1,180	124.8	4.41
B7Z7S2	cDNA FLJ61722, highly similar to Nance-Horan syndrome protein (Fragment) OS=Homo sapiens PE=2 SV=1 - [B7Z7S2_HUMAN]	1.39	5	1	1	1,153	125.5	7.28
C9JSZ3	Uncharacterized protein OS=Homo sapiens GN=IL17RC PE=4 SV=1 - [C9JSZ3_HUMAN]	2.42	1	1	1	330	36.6	5.85
E9PQV6	Uncharacterized protein OS=Homo sapiens GN=CBARA1 PE=4 SV=1 - [E9PQV6_HUMAN]	5.12	2	1	1	410	47.0	9.80
Q4VXL2	Transforming, acidic coiled-coil containing protein 2 OS=Homo sapiens GN=TACC2 PE=2 SV=1 - [Q4VXL2_HUMAN]	0.71	6	1	1	2,675	281.1	4.79
A6NCR3	Uncharacterized protein OS=Homo sapiens GN=SYNPO2L PE=4 SV=1 - [A6NCR3_HUMAN]	3.32	2	1	1	602	62.8	7.27
B5MDS4	Uncharacterized protein OS=Homo sapiens GN=ATHL1 PE=4 SV=1 - [B5MDS4_HUMAN]	5.88	2	1	1	323	36.3	4.87
E7EQX2	Uncharacterized protein OS=Homo sapiens GN=HECW1 PE=4 SV=1 - [E7EQX2_HUMAN]	7.51	2	1	1	346	38.8	9.58
Q96JS0	Tubby homologue (Fragment) OS=Homo sapiens GN=TUB PE=2 SV=1 - [Q96JS0_HUMAN]	3.49	5	1	1	373	40.4	9.23
A0PJ80	USP47 protein (Fragment) OS=Homo sapiens GN=USP47 PE=2 SV=1 - [A0PJ80_HUMAN]	2.11	3	1	1	331	38.4	5.58
B3KM88	cDNA FLJ10527 fis, clone NT2RP2000932, highly similar to Ankyrin repeat and MYND domain-containing protein 2 OS=Homo sapiens PE=2 SV=1 - [B3KM88_HUMAN]	5.90	3	1	1	441	49.3	6.25
E7EU99	Uncharacterized protein OS=Homo sapiens GN=CGREF1 PE=4 SV=1 - [E7EU99_HUMAN]	16.51	1	1	1	109	11.6	5.35
B4DTY1	cDNA FLJ51091, highly similar to 65 kDa Yes-associated protein OS=Homo sapiens PE=2 SV=1 - [B4DTY1_HUMAN]	5.21	6	1	1	326	36.2	4.81
C9JFU2	Uncharacterized protein OS=Homo sapiens GN=CHD6 PE=4 SV=1 - [C9JFU2_HUMAN]	2.07	2	1	1	822	94.5	7.91
A8MRS9	Uncharacterized protein OS=Homo sapiens GN=GPATCH4 PE=4 SV=1 - [A8MRS9_HUMAN]	7.27	1	1	1	330	37.4	9.00
B4DPD3	cDNA FLJ58602, highly similar to Chloride channel protein ClC-Ka OS=Homo sapiens PE=2 SV=1 - [B4DPD3_HUMAN]	2.80	5	1	1	644	70.5	7.50
C9J840	Uncharacterized protein OS=Homo sapiens GN=MAP4K4 PE=4 SV=1 - [C9J840_HUMAN]	1.82	9	1	1	987	113.5	6.80
D6RFK8	Uncharacterized protein OS=Homo sapiens GN=CASP12 PE=3 SV=1 - [D6RFK8_HUMAN]	8.17	3	1	1	257	29.4	6.21
A8KNJ4	MHC class I antigen OS=Homo sapiens GN=HLA-B PE=3 SV=1 - [A8KNJ4_HUMAN]	11.48	194	1	1	122	14.3	9.13
O43419	Intestinal mucin (Fragment) OS=Homo sapiens GN=MUC3 PE=2 SV=1 - [O43419_HUMAN]	2.38	1	1	1	589	60.5	6.80
C9JWY5	Uncharacterized protein OS=Homo sapiens GN=GPR17 PE=4 SV=1 - [C9JWY5_HUMAN]	13.24	3	1	1	136	15.0	7.42
C9JCM2	Uncharacterized protein OS=Homo sapiens GN=ZSCAN30 PE=4 SV=1 - [C9JCM2_HUMAN]	8.60	2	1	1	221	25.5	4.51
C9JUZ6	Uncharacterized protein OS=Homo sapiens GN=TMEM39A PE=4 SV=1 - [C9JUZ6_HUMAN]	25.71	3	1	1	140	15.5	9.38
Q5T911	Mediator complex subunit 4 (Fragment) OS=Homo sapiens GN=MED4 PE=2 SV=1 - [Q5T911_HUMAN]	4.20	3	1	1	238	27.1	5.63
Q16293	Clone MM1 product (Fragment) OS=Homo sapiens GN=MM1 PE=2 SV=1 - [Q16293_HUMAN]	5.16	1	1	1	349	38.3	7.09
B4DTI4	cDNA FLJ50668, highly similar to AP-2 complex subunit mu-1 OS=Homo sapiens PE=2 SV=1 - [B4DTI4_HUMAN]	5.90	8	1	1	305	34.8	9.54
Q3KQZ2	SYNGR2 protein OS=Homo sapiens GN=SYNGR2 PE=2 SV=1 - [Q3KQZ2_HUMAN]	2.55	1	1	1	275	30.4	9.20
B3KU60	cDNA FLJ39235 fis, clone OCBBF2007829, highly similar to Mus musculus fatso protein OS=Homo sapiens PE=2 SV=1 - [B3KU60_HUMAN]	1.98	2	1	1	505	58.3	5.22
B4DST3	cDNA FLJ56487, highly similar to Methionine synthase (EC 2.1.1.13) OS=Homo sapiens PE=2 SV=1 - [B4DST3_HUMAN]	1.79	4	1	1	1,118	124.0	5.57
A8K3J6	cDNA FLJ76260, highly similar to Homo sapiens T-box 5 (TBX5), transcript variant 1, mRNA OS=Homo sapiens PE=2 SV=1 - [A8K3J6_HUMAN]	3.86	2	1	1	518	57.7	7.66
B4DY68	cDNA FLJ51145, highly similar to Acrosin-binding protein OS=Homo sapiens PE=2 SV=1 - [B4DY68_HUMAN]	5.10	4	1	1	510	57.6	5.27
B7Z9G1	cDNA, FLJ78825 OS=Homo sapiens PE=4 SV=1 - [B7Z9G1_HUMAN]	22.89	1	1	1	83	9.3	9.60
Q5CZ68	Putative uncharacterized protein DKFZp686D20108 (Fragment) OS=Homo sapiens GN=DKFZp686D20108 PE=2 SV=1 - [Q5CZ68_HUMAN]	3.51	1	1	1	484	53.9	8.15
Q6ZSX8	cDNA FLJ45139 fis, clone BRAWH3039623 OS=Homo sapiens PE=2 SV=1 - [Q6ZSX8_HUMAN]	4.41	1	1	1	136	15.5	10.07
C9JL81	Uncharacterized protein OS=Homo sapiens GN=IQCD PE=4 SV=1 - [C9JL81_HUMAN]	2.71	2	1	1	369	42.6	7.93
C9J0A1	Uncharacterized protein OS=Homo sapiens GN=ARHGAP17 PE=4 SV=1 - [C9J0A1_HUMAN]	6.39	5	1	1	219	24.8	6.43
O95092	CLN3 protein (Fragment) OS=Homo sapiens GN=CLN3 PE=2 SV=1 - [O95092_HUMAN]	20.48	17	1	1	83	9.4	4.50
Q96KC0	cDNA FLJ14373 fis, clone HEMBA1001595, highly similar to SEPTIN 2 HOMOLOG OS=Homo sapiens PE=2 SV=1 - [Q96KC0_HUMAN]	2.68	17	1	1	261	30.6	6.67
Q59EB4	LATS, large tumor suppressor, homolog 2 variant (Fragment) OS=Homo sapiens PE=2 SV=1 - [Q59EB4_HUMAN]	0.76	2	1	1	924	101.4	9.22
B4DXK2	cDNA FLJ59097 OS=Homo sapiens PE=2 SV=1 - [B4DXK2_HUMAN]	3.24	4	1	1	216	24.2	8.88
B4E3H3	cDNA FLJ57018, highly similar to 1-phosphatidylinositol-4,5-bisphosphatephosphodiesterase gamma 2 (EC 3.1.4.11) OS=Homo sapiens PE=2 SV=1 - [B4E3H3_HUMAN]	0.80	2	1	1	1,132	132.7	6.51
Q9UK61	Uncharacterized protein C3orf63 OS=Homo sapiens GN=C3orf63 PE=1 SV=3 - [CC063_HUMAN]	0.84	1	1	1	1,670	188.9	5.80
Q0P6D6	Coiled-coil domain-containing protein 15 OS=Homo sapiens GN=CCDC15 PE=2 SV=2 - [CCD15_HUMAN]	0.74	2	1	1	951	110.4	6.47
Q8N9M1	Uncharacterized protein C19orf47 OS=Homo sapiens GN=C19orf47 PE=1 SV=1 - [CS047_HUMAN]	4.50	1	1	1	422	44.7	10.11
P48165	Gap junction alpha-8 protein OS=Homo sapiens GN=GJA8 PE=1 SV=3 - [CXA8_HUMAN]	7.16	1	1	1	433	48.2	5.27
Q92935	Exostosin-like 1 OS=Homo sapiens GN=EXTL1 PE=1 SV=2 - [EXTL1_HUMAN]	1.04	2	1	1	676	74.6	8.19
Q86YR5	G-protein-signaling modulator 1 OS=Homo sapiens GN=GPSM1 PE=1 SV=2 - [GPSM1_HUMAN]	1.48	1	1	1	675	74.5	6.54
Q8NA54	IQ and ubiquitin-like domain-containing protein OS=Homo sapiens GN=IQUB PE=1 SV=2 - [IQUB_HUMAN]	2.28	1	1	1	791	92.5	6.67
Q7Z418	Potassium channel subfamily K member 18 OS=Homo sapiens GN=KCNK18 PE=1 SV=1 - [KCNKI_HUMAN]	1.56	7	1	1	384	43.6	6.99
Q9H9A6	Leucine-rich repeat-containing protein 40 OS=Homo sapiens GN=LRRC40 PE=1 SV=1 - [LRC40_HUMAN]	1.50	1	1	1	602	68.2	6.43
Q9HCI5	Melanoma-associated antigen E1 OS=Homo sapiens GN=MAGEE1 PE=2 SV=2 - [MAGE1_HUMAN]	1.78	1	1	1	957	103.2	5.33
Q8N119	Matrix metalloproteinase-21 OS=Homo sapiens GN=MMP21 PE=2 SV=2 - [MMP21_HUMAN]	3.51	1	1	1	569	65.0	9.11
Q5JR59	Microtubule-associated tumor suppressor candidate 2 OS=Homo sapiens GN=MTUS2 PE=1 SV=3 - [MTUS2_HUMAN]	1.75	1	1	1	1,369	150.1	6.68
O95631	Netrin-1 OS=Homo sapiens GN=NTN1 PE=1 SV=2 - [NET1_HUMAN]	3.31	1	1	1	604	67.7	8.76
Q8NGE5	Olfactory receptor 10A7 OS=Homo sapiens GN=OR10A7 PE=2 SV=1 - [O10A7_HUMAN]	2.85	1	1	1	316	35.7	8.46
A3KFT3	Olfactory receptor 2M5 OS=Homo sapiens GN=OR2M5 PE=2 SV=1 - [OR2M5_HUMAN]	10.26	1	1	1	312	35.1	8.22
A6NMV5	PRAME family member 23 OS=Homo sapiens GN=PRAMEF23 PE=3 SV=1 - [PRA23_HUMAN]	5.25	3	1	1	476	54.8	8.05
P01111	GTPase NRas OS=Homo sapiens GN=NRAS PE=1 SV=1 - [RASN_HUMAN]	6.35	2	1	1	189	21.2	5.17
Q6ZSC3	RNA-binding protein 43 OS=Homo sapiens GN=RBM43 PE=2 SV=1 - [RBM43_HUMAN]	2.24	1	1	1	357	40.6	9.66
Q8NE28	Protein kinase-like protein SgK071 OS=Homo sapiens GN=SGK071 PE=2 SV=4 - [SGK71_HUMAN]	1.91	1	1	1	680	75.6	5.36
A8MU46	Smoothelin-like protein 1 OS=Homo sapiens GN=SMTNL1 PE=1 SV=1 - [SMTL1_HUMAN]	1.97	3	1	1	457	48.9	4.75
Q7Z7C7	Stimulated by retinoic acid gene 8 protein homolog OS=Homo sapiens GN=STRA8 PE=2 SV=1 - [STRA8_HUMAN]	3.64	1	1	1	330	36.9	5.06
O95258	Brain mitochondrial carrier protein 1 OS=Homo sapiens GN=SLC25A14 PE=2 SV=1 - [UCP5_HUMAN]	3.08	1	1	1	325	36.2	9.64
Q96NJ1	Uncharacterized protein FLJ30774 OS=Homo sapiens PE=1 SV=1 - [YI001_HUMAN]	7.14	1	1	1	140	14.3	11.49
B4DG90	cDNA FLJ55199, highly similar to LIM-only protein 3 OS=Homo sapiens PE=2 SV=1 - [B4DG90_HUMAN]	10.43	1	1	1	163	18.4	8.32
B4DUJ4	Casein kinase 2, beta polypeptide, isoform CRA_c OS=Homo sapiens GN=CSNK2B PE=4 SV=1 - [B4DUJ4_HUMAN]	5.45	4	1	1	110	12.8	4.21
E1P5F6	Cell division cycle 40 homolog (Yeast), isoform CRA_d OS=Homo sapiens GN=CDC40 PE=4 SV=1 - [E1P5F6_HUMAN]	4.71	1	1	1	340	39.3	8.31
Q96S40	Excision repair cross-complementing rodent repair deficiency, complementation group 1 (Includes overlapping antisense sequence), isoform CRA_b OS=Homo sapiens GN=ERCC1 PE=2 SV=1 - [Q96S40_HUMAN]	3.30	4	1	1	273	30.0	6.24
Q5TBQ7	TBC1 domain family, member 2 OS=Homo sapiens GN=TBC1D2 PE=2 SV=1 - [Q5TBQ7_HUMAN]	1.13	4	1	1	710	81.6	7.21
Q147X6	ASB16 protein OS=Homo sapiens GN=ASB16 PE=2 SV=1 - [Q147X6_HUMAN]	2.93	3	1	1	273	29.6	7.20
A5PL12	Astrotactin 1 OS=Homo sapiens GN=ASTN1 PE=2 SV=1 - [A5PL12_HUMAN]	1.48	6	1	1	1,216	135.0	5.14
C9J228	Uncharacterized protein OS=Homo sapiens GN=PLCH1 PE=4 SV=1 - [C9J228_HUMAN]	0.81	2	1	1	991	113.2	7.34
Q12916	Sel-1-like protein OS=Homo sapiens GN=SEL1L PE=2 SV=1 - [Q12916_HUMAN]	7.37	3	1	1	95	10.7	8.24
A0JNW0	SBNO2 protein OS=Homo sapiens GN=SBNO2 PE=4 SV=1 - [A0JNW0_HUMAN]	15.63	4	1	1	64	7.0	6.49
Q5QPM0	RNA binding protein, autoantigenic (HnRNP-associated with lethal yellow homolog (Mouse)) (Fragment) OS=Homo sapiens GN=RALY PE=2 SV=1 - [Q5QPM0_HUMAN]	5.81	7	1	1	172	18.7	10.02
B4E0J7	cDNA FLJ52380, highly similar to RNA polymerase I-specific transcriptioninitiation factor RRN3 OS=Homo sapiens PE=2 SV=1 - [B4E0J7_HUMAN]	4.05	7	1	1	469	53.3	5.26
C9JHB0	Uncharacterized protein OS=Homo sapiens GN=ITPK1 PE=4 SV=1 - [C9JHB0_HUMAN]	5.28	4	1	1	322	35.9	4.93
E9PP83	Uncharacterized protein OS=Homo sapiens GN=TRIM68 PE=4 SV=1 - [E9PP83_HUMAN]	4.93	2	1	1	142	16.8	7.99
B2R805	cDNA, FLJ93684, highly similar to Homo sapiens mab-21-like 1 (C. elegans) (MAB21L1), mRNA OS=Homo sapiens PE=2 SV=1 - [B2R805_HUMAN]	2.51	2	1	1	359	41.0	8.73
B4DTV4	cDNA FLJ50215, highly similar to Myosin Ia OS=Homo sapiens PE=2 SV=1 - [B4DTV4_HUMAN]	1.25	3	1	1	881	100.2	9.51
E9PR90	Uncharacterized protein OS=Homo sapiens GN=LAYN PE=4 SV=1 - [E9PR90_HUMAN]	13.43	4	1	1	134	15.1	7.06
B4E3V3	cDNA FLJ60227, highly similar to Peroxisome proliferator-activated receptor delta OS=Homo sapiens PE=2 SV=1 - [B4E3V3_HUMAN]	2.33	5	1	1	343	38.8	6.39
B7Z824	cDNA FLJ52407, highly similar to Crumbs homolog 1 OS=Homo sapiens PE=2 SV=1 - [B7Z824_HUMAN]	2.03	5	1	1	887	98.0	5.22
Q5T7E4	GTP binding protein 2 (Fragment) OS=Homo sapiens GN=GTPBP2 PE=2 SV=1 - [Q5T7E4_HUMAN]	5.88	2	1	1	153	16.7	8.66
Q4ZG84	Putative uncharacterized protein LRP2 (Fragment) OS=Homo sapiens GN=LRP2 PE=2 SV=1 - [Q4ZG84_HUMAN]	0.54	4	1	1	3,881	435.5	5.14
A7KBT6	B cell maturation antigen transcript variant 5 (Fragment) OS=Homo sapiens GN=BCMA PE=2 SV=1 - [A7KBT6_HUMAN]	17.42	4	1	1	132	14.6	8.28
D7EZH4	SNF2LT OS=Homo sapiens PE=2 SV=1 - [D7EZH4_HUMAN]	1.03	6	1	1	776	89.3	8.12
B4DJD1	cDNA FLJ54933, highly similar to Homo sapiens bone marrow protein BM039 (BM039), mRNA OS=Homo sapiens PE=2 SV=1 - [B4DJD1_HUMAN]	5.64	4	1	1	319	37.1	9.17
B4DEF1	cDNA FLJ53828, highly similar to Guanine nucleotide-binding protein-like 1 OS=Homo sapiens PE=2 SV=1 - [B4DEF1_HUMAN]	6.11	13	1	1	262	28.7	4.69
Q68DH9	Putative uncharacterized protein DKFZp686M088 OS=Homo sapiens GN=DKFZp686M088 PE=2 SV=1 - [Q68DH9_HUMAN]	1.49	1	1	1	1,205	133.8	6.49
A8K6A5	cDNA FLJ77742, highly similar to Homo sapiens integrin, alpha 5 (fibronectin receptor, alpha polypeptide), mRNA OS=Homo sapiens PE=2 SV=1 - [A8K6A5_HUMAN]	1.05	3	1	1	1,049	114.4	5.71
B4DZR4	cDNA FLJ58653 OS=Homo sapiens PE=2 SV=1 - [B4DZR4_HUMAN]	4.00	4	1	1	800	92.3	7.99
Q5SYT8	Novel protein similar to Pre-B cell enhancing factor (PBEF) (Fragment) OS=Homo sapiens GN=RP11-92J19.4 PE=1 SV=1 - [Q5SYT8_HUMAN]	3.81	2	1	1	472	53.4	7.80
Q6NVU7	ZP1 protein OS=Homo sapiens GN=ZP1 PE=2 SV=1 - [Q6NVU7_HUMAN]	4.55	2	1	1	330	36.2	6.89
B2RC35	cDNA, FLJ95833 OS=Homo sapiens PE=2 SV=1 - [B2RC35_HUMAN]	6.55	2	1	1	275	32.3	8.37
Q9NNX4	Hemochromatosis protein (Fragment) OS=Homo sapiens PE=2 SV=1 - [Q9NNX4_HUMAN]	1.75	1	1	1	342	40.0	9.31
B2R9P6	cDNA, FLJ94498, highly similar to Homo sapiens guanylate cyclase activator 1A (retina) (GUCA1A),mRNA OS=Homo sapiens PE=2 SV=1 - [B2R9P6_HUMAN]	4.98	3	1	1	201	22.8	4.48
B4DNK0	cDNA FLJ55582, highly similar to Homo sapiens DPH1 homolog (DPH1), mRNA OS=Homo sapiens PE=2 SV=1 - [B4DNK0_HUMAN]	5.74	2	1	1	453	50.2	8.31
B4E189	cDNA FLJ51440, highly similar to Condensin complex subunit 2 OS=Homo sapiens PE=2 SV=1 - [B4E189_HUMAN]	1.16	5	1	1	605	67.5	4.87
Q562Z4	Actin-like protein (Fragment) OS=Homo sapiens GN=ACT PE=2 SV=1 - [Q562Z4_HUMAN]	19.42	1	1	1	103	11.5	7.58
B4DMY1	cDNA FLJ60310 OS=Homo sapiens PE=2 SV=1 - [B4DMY1_HUMAN]	4.29	4	1	1	350	38.3	4.82
C5HZ26	Galectin-16 OS=Homo sapiens GN=LGALS16 PE=2 SV=1 - [C5HZ26_HUMAN]	16.20	1	1	1	142	16.6	6.07
B4DW71	cDNA FLJ58836, highly similar to Ancient ubiquitous protein 1 OS=Homo sapiens PE=2 SV=1 - [B4DW71_HUMAN]	4.70	6	1	1	149	16.4	6.37
B7Z6I7	cDNA FLJ55423, highly similar to Homo sapiens Nik related kinase (NRK), mRNA OS=Homo sapiens PE=2 SV=1 - [B7Z6I7_HUMAN]	5.71	1	1	1	140	16.2	8.63
E7EQB0	Ubiquitin carboxyl-terminal hydrolase OS=Homo sapiens GN=USP42 PE=3 SV=1 - [E7EQB0_HUMAN]	0.64	4	1	1	1,245	137.2	9.28
E9PFM7	Uncharacterized protein OS=Homo sapiens GN=PARP9 PE=4 SV=1 - [E9PFM7_HUMAN]	3.24	3	1	1	710	80.2	8.06
C9JIA1	Uncharacterized protein OS=Homo sapiens GN=SLFN12 PE=4 SV=1 - [C9JIA1_HUMAN]	20.29	3	1	1	69	7.7	6.61
Q6Q3U3	HBV pre-S2 trans-regulated protein 4 OS=Homo sapiens PE=4 SV=1 - [Q6Q3U3_HUMAN]	24.10	1	1	1	83	9.0	9.10
Q4W5S4	Putative uncharacterized protein GRID2 (Fragment) OS=Homo sapiens GN=GRID2 PE=2 SV=1 - [Q4W5S4_HUMAN]	37.25	1	1	1	51	5.7	4.20
B2RBZ0	cDNA, FLJ95773, highly similar to Homo sapiens tripartite motif-containing 7 (TRIM7), mRNA OS=Homo sapiens PE=2 SV=1 - [B2RBZ0_HUMAN]	2.71	10	1	1	221	23.7	6.93
D6RGH1	Uncharacterized protein OS=Homo sapiens PE=4 SV=1 - [D6RGH1_HUMAN]	5.13	1	1	1	156	17.5	8.19
E9PAK5	Uncharacterized protein OS=Homo sapiens GN=DENND4B PE=4 SV=1 - [E9PAK5_HUMAN]	0.99	2	1	1	1,218	133.3	6.73
Q6ZNE1	cDNA FLJ16186 fis, clone BRTHA2007060, moderately similar to EUKARYOTIC TRANSLATION INITIATION FACTOR 3 SUBUNIT 10 OS=Homo sapiens PE=1 SV=1 - [Q6ZNE1_HUMAN]	0.73	1	1	1	957	111.7	9.33
B4DIC3	cDNA FLJ57841, highly similar to Centaurin-gamma 2 OS=Homo sapiens PE=2 SV=1 - [B4DIC3_HUMAN]	4.44	6	1	1	248	27.6	6.62
F1JTL6	Epidermal growth factor receptor (Fragment) OS=Homo sapiens GN=EGFR PE=2 SV=1 - [F1JTL6_HUMAN]	8.09	13	1	1	136	15.0	7.50
D6R9F4	Uncharacterized protein OS=Homo sapiens GN=IBTK PE=4 SV=1 - [D6R9F4_HUMAN]	6.30	6	1	1	238	26.3	8.09
C9JYJ6	Uncharacterized protein OS=Homo sapiens GN=FILIP1L PE=4 SV=1 - [C9JYJ6_HUMAN]	0.72	3	1	1	837	96.2	6.28
B2RBQ1	cDNA, FLJ95628, Homo sapiens ring finger protein 12 (RNF12), mRNA OS=Homo sapiens PE=2 SV=1 - [B2RBQ1_HUMAN]	1.24	2	1	1	483	52.6	9.47
Q59GI3	I-kappa-B-related protein variant (Fragment) OS=Homo sapiens PE=2 SV=1 - [Q59GI3_HUMAN]	1.56	2	1	1	897	97.0	6.18
C9JMM4	Uncharacterized protein OS=Homo sapiens GN=FASTKD1 PE=4 SV=1 - [C9JMM4_HUMAN]	18.27	7	1	1	104	11.9	8.18
Q8NH29	Seven transmembrane helix receptor OS=Homo sapiens PE=3 SV=1 - [Q8NH29_HUMAN]	3.15	1	1	1	222	25.4	7.88
B4E035	cDNA FLJ56186 OS=Homo sapiens PE=2 SV=1 - [B4E035_HUMAN]	3.62	3	1	1	387	41.8	10.17
A6NC57	Ankyrin repeat domain-containing protein 62 OS=Homo sapiens GN=ANKRD62 PE=2 SV=4 - [ANR62_HUMAN]	1.20	1	1	1	917	106.4	6.67
Q9C0F0	Putative Polycomb group protein ASXL3 OS=Homo sapiens GN=ASXL3 PE=2 SV=3 - [ASXL3_HUMAN]	0.36	1	1	1	2,248	241.8	6.14
P55201	Peregrin OS=Homo sapiens GN=BRPF1 PE=1 SV=2 - [BRPF1_HUMAN]	1.48	1	1	1	1,214	137.4	7.93
P02745	Complement C1q subcomponent subunit A OS=Homo sapiens GN=C1QA PE=1 SV=2 - [C1QA_HUMAN]	4.08	1	1	1	245	26.0	9.11
P55291	Cadherin-15 OS=Homo sapiens GN=CDH15 PE=1 SV=1 - [CAD15_HUMAN]	1.11	1	1	1	814	88.9	4.98
Q08AD1	Calmodulin-regulated spectrin-associated protein 2 OS=Homo sapiens GN=CAMSAP1L1 PE=1 SV=3 - [CAMP2_HUMAN]	1.07	1	1	1	1,489	168.0	6.80
Q9BXL7	Caspase recruitment domain-containing protein 11 OS=Homo sapiens GN=CARD11 PE=1 SV=3 - [CAR11_HUMAN]	2.69	2	1	1	1,154	133.2	6.09
Q96IY4	Carboxypeptidase B2 OS=Homo sapiens GN=CPB2 PE=1 SV=2 - [CBPB2_HUMAN]	4.73	1	1	1	423	48.4	7.71
Q8IVM0	Coiled-coil domain-containing protein 50 OS=Homo sapiens GN=CCDC50 PE=1 SV=1 - [CCD50_HUMAN]	2.61	1	1	1	306	35.8	6.65
Q8N4T4	Vav-like protein C9orf100 OS=Homo sapiens GN=C9orf100 PE=2 SV=1 - [CI100_HUMAN]	1.79	1	1	1	335	38.3	9.64
P04632	Calpain small subunit 1 OS=Homo sapiens GN=CAPNS1 PE=1 SV=1 - [CPNS1_HUMAN]	8.96	1	1	1	268	28.3	5.20
Q8N8A6	ATP-dependent RNA helicase DDX51 OS=Homo sapiens GN=DDX51 PE=1 SV=3 - [DDX51_HUMAN]	1.20	1	1	1	666	72.4	8.16
Q14147	Probable ATP-dependent RNA helicase DHX34 OS=Homo sapiens GN=DHX34 PE=1 SV=2 - [DHX34_HUMAN]	0.70	1	1	1	1,143	128.0	7.56
Q7L775	EPM2A-interacting protein 1 OS=Homo sapiens GN=EPM2AIP1 PE=1 SV=1 - [EPMIP_HUMAN]	2.64	1	1	1	607	70.3	6.11
Q08E93	Protein FAM27E3 OS=Homo sapiens GN=FAM27E3 PE=1 SV=1 - [F27E3_HUMAN]	12.39	3	1	1	113	13.5	11.88
Q7Z6J2	General receptor for phosphoinositides 1-associated scaffold protein OS=Homo sapiens GN=GRASP PE=1 SV=1 - [GRASP_HUMAN]	7.85	1	1	1	395	42.6	8.84
P48741	Putative heat shock 70 kDa protein 7 OS=Homo sapiens GN=HSPA7 PE=5 SV=2 - [HSP77_HUMAN]	2.45	1	1	1	367	40.2	7.87
P09565	Putative insulin-like growth factor 2-associated protein OS=Homo sapiens GN=GIG44 PE=4 SV=2 - [IG2R_HUMAN]	17.70	1	1	1	113	12.1	9.41
Q8NEH6	Meiosis-specific nuclear structural protein 1 OS=Homo sapiens GN=MNS1 PE=2 SV=2 - [MNS1_HUMAN]	3.43	1	1	1	495	60.5	7.12
O60682	Musculin OS=Homo sapiens GN=MSC PE=1 SV=2 - [MUSC_HUMAN]	3.40	1	1	1	206	22.1	9.09
Q9Y5G4	Protocadherin gamma-A9 OS=Homo sapiens GN=PCDHGA9 PE=2 SV=1 - [PCDG9_HUMAN]	1.82	1	1	1	932	101.6	5.05
P02776	Platelet factor 4 OS=Homo sapiens GN=PF4 PE=1 SV=2 - [PLF4_HUMAN]	8.91	2	1	1	101	10.8	8.62
Q16821	Protein phosphatase 1 regulatory subunit 3A OS=Homo sapiens GN=PPP1R3A PE=1 SV=3 - [PPR3A_HUMAN]	0.62	1	1	1	1,122	125.7	5.00
Q8N0V3	Putative ribosome-binding factor A, mitochondrial OS=Homo sapiens GN=RBFA PE=1 SV=3 - [RBFA_HUMAN]	2.04	1	1	1	343	38.3	7.85
P62877	E3 ubiquitin-protein ligase RBX1 OS=Homo sapiens GN=RBX1 PE=1 SV=1 - [RBX1_HUMAN]	5.56	1	1	1	108	12.3	6.96
P12271	Retinaldehyde-binding protein 1 OS=Homo sapiens GN=RLBP1 PE=1 SV=2 - [RLBP1_HUMAN]	10.09	1	1	1	317	36.5	5.05
Q96KN7	X-linked retinitis pigmentosa GTPase regulator-interacting protein 1 OS=Homo sapiens GN=RPGRIP1 PE=1 SV=2 - [RPGR1_HUMAN]	0.54	1	1	1	1,286	146.6	5.68
Q12981	Vesicle transport protein SEC20 OS=Homo sapiens GN=BNIP1 PE=1 SV=3 - [SEC20_HUMAN]	2.63	1	1	1	228	26.1	8.95
O75764	Transcription elongation factor A protein 3 OS=Homo sapiens GN=TCEA3 PE=2 SV=2 - [TCEA3_HUMAN]	4.31	1	1	1	348	38.9	9.19
P20061	Transcobalamin-1 OS=Homo sapiens GN=TCN1 PE=1 SV=2 - [TCO1_HUMAN]	1.85	1	1	1	433	48.2	5.03
Q96RU7	Tribbles homolog 3 OS=Homo sapiens GN=TRIB3 PE=1 SV=2 - [TRIB3_HUMAN]	5.59	1	1	1	358	39.6	8.09
Q9UNY4	Transcription termination factor 2 OS=Homo sapiens GN=TTF2 PE=1 SV=2 - [TTF2_HUMAN]	1.55	1	1	1	1,162	129.5	8.37
A6NNY8	Ubiquitin carboxyl-terminal hydrolase 27 OS=Homo sapiens GN=USP27X PE=2 SV=3 - [UBP27_HUMAN]	1.83	1	1	1	438	49.6	7.18
P55851	Mitochondrial uncoupling protein 2 OS=Homo sapiens GN=UCP2 PE=1 SV=1 - [UCP2_HUMAN]	2.59	1	1	1	309	33.2	9.70
Q8TBY9	WD repeat-containing protein 66 OS=Homo sapiens GN=WDR66 PE=1 SV=2 - [WDR66_HUMAN]	1.31	1	1	1	1,149	129.9	5.08
Q96MM3	Zinc finger protein 42 homolog OS=Homo sapiens GN=ZFP42 PE=1 SV=2 - [ZFP42_HUMAN]	5.81	1	1	1	310	34.8	8.90
Q9UK13	Zinc finger protein 221 OS=Homo sapiens GN=ZNF221 PE=2 SV=3 - [ZN221_HUMAN]	1.94	1	1	1	617	71.1	8.43
B4DFL4	cDNA FLJ50470, moderately similar to Rattus norvegicus breast carcinoma amplified sequence 1 (Bcas1), mRNA OS=Homo sapiens PE=2 SV=1 - [B4DFL4_HUMAN]	3.60	7	1	1	250	26.9	9.66
Q96NB5	Chromosome 3 open reading frame 33, isoform CRA_b OS=Homo sapiens GN=C3orf33 PE=2 SV=1 - [Q96NB5_HUMAN]	3.59	2	1	1	251	29.2	9.91
Q86YU0	Carbonic anhydrase VII short form OS=Homo sapiens GN=CA7 PE=2 SV=1 - [Q86YU0_HUMAN]	4.33	2	1	1	208	23.4	8.29
B4DGX8	Ret proto-oncogene (Multiple endocrine neoplasia and medullary thyroid carcinoma 1, Hirschsprung disease), isoform CRA_c OS=Homo sapiens GN=RET PE=2 SV=1 - [B4DGX8_HUMAN]	2.08	3	1	1	818	90.7	6.49
D3DUP2	WNK lysine deficient protein kinase 1, isoform CRA_d OS=Homo sapiens GN=WNK1 PE=4 SV=1 - [D3DUP2_HUMAN]	0.28	3	1	1	2,107	222.6	6.43
D3DX86	Putative uncharacterized protein MGC3101 OS=Homo sapiens GN=MGC3101 PE=4 SV=1 - [D3DX86_HUMAN]	22.37	2	1	1	76	8.6	4.53
E9PF14	Uncharacterized protein OS=Homo sapiens GN=CDK5RAP1 PE=4 SV=1 - [E9PF14_HUMAN]	3.06	6	1	1	327	36.7	6.79
A6NLR7	Uncharacterized protein OS=Homo sapiens GN=CD200 PE=4 SV=2 - [A6NLR7_HUMAN]	8.21	2	1	1	195	21.9	8.34
B4DM00	cDNA FLJ54367, highly similar to Amyloid beta A4 protein (APP) (ABPP)(Alzheimer disease amyloid protein homolog) OS=Homo sapiens PE=2 SV=1 - [B4DM00_HUMAN]	1.33	16	1	1	528	60.0	6.09
Q0VAL7	C21orf58 protein OS=Homo sapiens GN=C21orf58 PE=2 SV=1 - [Q0VAL7_HUMAN]	4.32	2	1	1	162	17.7	10.08
A5JQJ6	Factor VIII (Fragment) OS=Homo sapiens GN=F8 PE=4 SV=1 - [A5JQJ6_HUMAN]	25.93	4	1	1	54	6.2	9.70
B3KUW3	cDNA FLJ40751 fis, clone TRACH2000702, highly similar to Coiled-coil domain-containing protein 46 OS=Homo sapiens PE=2 SV=1 - [B3KUW3_HUMAN]	1.16	4	1	1	519	62.0	6.01
Q96CK3	CAD protein (Fragment) OS=Homo sapiens GN=CAD PE=2 SV=2 - [Q96CK3_HUMAN]	0.82	3	1	1	851	93.4	7.66
Q86Y85	Potassium voltage-gated channel, subfamily G, member 1 OS=Homo sapiens GN=KCNG1 PE=2 SV=1 - [Q86Y85_HUMAN]	4.09	2	1	1	513	57.8	6.46
Q9UFB4	Putative uncharacterized protein DKFZp434B1917 (Fragment) OS=Homo sapiens GN=DKFZp434B1917 PE=2 SV=1 - [Q9UFB4_HUMAN]	1.91	2	1	1	366	43.4	9.80
B3KUS6	cDNA FLJ40512 fis, clone TESTI2046439, highly similar to Serine/threonine-protein kinase MAK (EC 2.7.11.22) OS=Homo sapiens PE=2 SV=1 - [B3KUS6_HUMAN]	2.78	3	1	1	288	33.6	8.91
Q5T1M3	Solute carrier family 31 (Copper transporters), member 1 OS=Homo sapiens GN=SLC31A1 PE=2 SV=1 - [Q5T1M3_HUMAN]	10.64	1	1	1	188	21.0	7.15
B1ANH6	Guanylate kinase 1 OS=Homo sapiens GN=GUK1 PE=4 SV=1 - [B1ANH6_HUMAN]	7.44	1	1	1	242	26.3	8.92
B3KPN9	cDNA FLJ32007 fis, clone NT2RP7009481, highly similar to Homo sapiens dispatched homolog 1 (Drosophila) (DISP1), mRNA OS=Homo sapiens PE=2 SV=1 - [B3KPN9_HUMAN]	0.50	2	1	1	1,203	135.2	6.79
E9PE50	Uncharacterized protein OS=Homo sapiens GN=NLRP1 PE=4 SV=1 - [E9PE50_HUMAN]	1.31	2	1	1	1,375	154.8	6.96
B2R6L0	cDNA, FLJ93005, highly similar to Homo sapiens tubulin, beta polypeptide (TUBB), mRNA OS=Homo sapiens PE=2 SV=1 - [B2R6L0_HUMAN]	3.37	1	1	1	445	49.9	4.89
B4DDI3	cDNA FLJ59836, highly similar to Cytochrome P450 11A1, mitochondrial(EC 1.14.15.6) OS=Homo sapiens PE=2 SV=1 - [B4DDI3_HUMAN]	6.94	6	1	1	144	16.4	9.31
B3KXP1	cDNA FLJ45802 fis, clone NT2RI3001967, highly similar to Ankyrin repeat and SAM domain-containing protein 6 OS=Homo sapiens PE=2 SV=1 - [B3KXP1_HUMAN]	1.04	2	1	1	676	72.5	8.97
B7Z5N1	cDNA FLJ60828, highly similar to Protein-cysteine N-palmitoyltransferase HHAT(EC 2.3.1.-) OS=Homo sapiens PE=2 SV=1 - [B7Z5N1_HUMAN]	1.86	6	1	1	430	49.6	8.25
Q86YM6	HOMER1F OS=Homo sapiens GN=HOMER1 PE=2 SV=1 - [Q86YM6_HUMAN]	3.33	4	1	1	180	20.8	5.01
Q6LCG8	Catenin-4 (Fragment) OS=Homo sapiens PE=2 SV=1 - [Q6LCG8_HUMAN]	2.11	3	1	1	617	68.0	9.29
B3KQZ4	cDNA FLJ33327 fis, clone BNGH42009025, weakly similar to Homo sapiens t-complex 11(TCP11), mRNA OS=Homo sapiens PE=2 SV=1 - [B3KQZ4_HUMAN]	3.34	2	1	1	509	57.0	5.59
E7EN42	Uncharacterized protein OS=Homo sapiens GN=FAM57A PE=4 SV=1 - [E7EN42_HUMAN]	2.12	2	1	1	330	37.2	11.08
Q6AW96	Putative uncharacterized protein DKFZp686A20205 OS=Homo sapiens GN=DKFZp686A20205 PE=2 SV=1 - [Q6AW96_HUMAN]	0.87	1	1	1	923	101.4	8.06
B4DN28	cDNA FLJ54639, highly similar to Integrin alpha-8 (Fragment) OS=Homo sapiens PE=2 SV=1 - [B4DN28_HUMAN]	0.76	2	1	1	1,048	115.9	5.68
A8MWQ3	Uncharacterized protein OS=Homo sapiens GN=CIT PE=4 SV=1 - [A8MWQ3_HUMAN]	0.99	3	1	1	1,618	185.7	6.71
B4DVW4	cDNA FLJ51116, highly similar to Interleukin-10 receptor alpha chain OS=Homo sapiens PE=2 SV=1 - [B4DVW4_HUMAN]	3.73	2	1	1	429	46.3	5.12
B4DVP9	cDNA FLJ60005, moderately similar to Homo sapiens pleckstrin homology domain containing, family M member 1, mRNA OS=Homo sapiens PE=2 SV=1 - [B4DVP9_HUMAN]	10.53	3	1	1	171	19.0	5.97
Q9H918	cDNA FLJ13079 fis, clone NT2RP3002004, moderately similar to H.sapiens FAST kinase OS=Homo sapiens PE=2 SV=1 - [Q9H918_HUMAN]	6.92	7	1	1	159	16.9	9.55
B7Z800	cDNA FLJ57673, highly similar to Adenosine kinase (EC 2.7.1.20) OS=Homo sapiens PE=2 SV=1 - [B7Z800_HUMAN]	1.83	1	1	1	327	36.6	6.79
E5RHS5	Uncharacterized protein OS=Homo sapiens GN=EIF3E PE=4 SV=1 - [E5RHS5_HUMAN]	8.85	6	1	1	226	26.6	6.06
D6REC6	Uncharacterized protein OS=Homo sapiens GN=CDK7 PE=4 SV=1 - [D6REC6_HUMAN]	10.07	4	1	1	149	17.0	5.27
Q86TY2	Full-length cDNA clone CS0DI035YL21 of Placenta of Homo sapiens (human) (Fragment) OS=Homo sapiens PE=2 SV=1 - [Q86TY2_HUMAN]	5.98	6	1	1	301	33.2	5.52
A8K5X7	cDNA FLJ76854 OS=Homo sapiens PE=2 SV=1 - [A8K5X7_HUMAN]	3.67	2	1	1	409	46.8	9.11
B4DWD2	cDNA FLJ58634, weakly similar to Mus musculus microtubule-associated protein 7 domain containing 1 (Mtap7d1), mRNA OS=Homo sapiens PE=2 SV=1 - [B4DWD2_HUMAN]	1.86	2	1	1	858	96.7	9.32
D6R919	Uncharacterized protein OS=Homo sapiens GN=SH3BP2 PE=4 SV=1 - [D6R919_HUMAN]	5.50	9	1	1	109	12.3	10.24
E5RJX4	Uncharacterized protein OS=Homo sapiens GN=PPP2CB PE=4 SV=1 - [E5RJX4_HUMAN]	17.02	5	1	1	47	5.6	4.65
Q59EL6	Ankyrin repeat and SOCS box-containing protein 3 isoform a variant (Fragment) OS=Homo sapiens PE=2 SV=1 - [Q59EL6_HUMAN]	5.59	6	1	1	340	37.2	5.86
A8K9F8	cDNA FLJ78036, highly similar to Homo sapiens caspase recruitment domain family, member 12, mRNA OS=Homo sapiens PE=2 SV=1 - [A8K9F8_HUMAN]	0.59	3	1	1	1,024	116.1	6.73
Q53HK3	Eukaryotic translation initiation factor 2, subunit 3 gamma, 52kDa variant (Fragment) OS=Homo sapiens PE=2 SV=1 - [Q53HK3_HUMAN]	3.39	1	1	1	472	51.1	8.40
Q5VZB4	SLIT-ROBO Rho GTPase activating protein 2 (Fragment) OS=Homo sapiens GN=SRGAP2 PE=2 SV=1 - [Q5VZB4_HUMAN]	3.56	7	1	1	450	50.7	5.29
C9JTX5	Uncharacterized protein OS=Homo sapiens GN=ACTB PE=3 SV=1 - [C9JTX5_HUMAN]	13.75	32	1	1	80	8.5	5.35
B2R7Q9	cDNA, FLJ93562 OS=Homo sapiens PE=2 SV=1 - [B2R7Q9_HUMAN]	3.50	2	1	1	543	59.6	4.82
A2RRC9	IQ motif containing GTPase activating protein 3 OS=Homo sapiens GN=IQGAP3 PE=2 SV=1 - [A2RRC9_HUMAN]	0.61	2	1	1	1,631	184.4	7.49
B4DI12	cDNA FLJ56014, highly similar to Homo sapiens kinase D-interacting substance of 220 kDa (KIDINS220), mRNA OS=Homo sapiens PE=2 SV=1 - [B4DI12_HUMAN]	3.58	4	1	1	587	64.6	5.50
E7ESG1	Uncharacterized protein OS=Homo sapiens GN=DNASE1L1 PE=4 SV=1 - [E7ESG1_HUMAN]	14.52	6	1	1	62	6.8	8.02
E9PJG4	Uncharacterized protein OS=Homo sapiens GN=FDFT1 PE=4 SV=1 - [E9PJG4_HUMAN]	7.33	3	1	1	150	17.7	7.80
A8K591	cDNA FLJ77127, highly similar to Homo sapiens forkhead box M1 (FOXM1), transcript variant 3, mRNA OS=Homo sapiens PE=2 SV=1 - [A8K591_HUMAN]	0.80	2	1	1	747	82.6	8.35
Q5VVP7	C-reactive protein, pentraxin-related OS=Homo sapiens GN=CRP PE=2 SV=1 - [Q5VVP7_HUMAN]	5.88	2	1	1	102	11.6	8.46
B8ZZJ3	Uncharacterized protein OS=Homo sapiens GN=ALMS1 PE=4 SV=1 - [B8ZZJ3_HUMAN]	0.15	3	1	1	4,125	456.0	6.29
B8ZZ29	Phosphatidylinositol glycan anchor biosynthesis, class Q (Fragment) OS=Homo sapiens GN=PIGQ PE=4 SV=1 - [B8ZZ29_HUMAN]	14.29	9	1	1	112	12.5	7.72
Q71V38	Dihydroxyacetone phosphate acyltransferase (Fragment) OS=Homo sapiens PE=2 SV=1 - [Q71V38_HUMAN]	1.83	9	1	1	383	42.9	5.97
Q6ZTI3	cDNA FLJ44621 fis, clone BRACE2016896, highly similar to Lysyl-tRNA synthetase (EC 6.1.1.6) OS=Homo sapiens PE=2 SV=1 - [Q6ZTI3_HUMAN]	2.13	3	1	1	423	48.4	5.38
Q6NYC5	MAP2 protein (Fragment) OS=Homo sapiens GN=MAP2 PE=2 SV=1 - [Q6NYC5_HUMAN]	1.79	2	1	1	1,064	116.3	4.67
C9K0E7	Uncharacterized protein OS=Homo sapiens GN=FAM115A PE=4 SV=1 - [C9K0E7_HUMAN]	12.33	4	1	1	146	15.4	4.50
Q8WYP1	Ribosomal protein L5 (Fragment) OS=Homo sapiens GN=RPL5 PE=2 SV=1 - [Q8WYP1_HUMAN]	11.29	4	1	1	62	7.4	10.33
A8K2M7	cDNA FLJ77039, highly similar to Homo sapiens c-myc promoter binding protein (MYCPBP), mRNA (Fragment) OS=Homo sapiens PE=2 SV=1 - [A8K2M7_HUMAN]	1.08	1	1	1	927	104.8	8.29
B4DUL7	cDNA FLJ58283, highly similar to Mus musculus leiomodin 2 (cardiac) (Lmod2), mRNA OS=Homo sapiens PE=2 SV=1 - [B4DUL7_HUMAN]	1.61	5	1	1	497	56.0	8.63
E2J9M0	Truncated protein tyrosine phosphatase non-receptor type 14 OS=Homo sapiens GN=PTPN14 PE=2 SV=1 - [E2J9M0_HUMAN]	3.79	5	1	1	211	24.8	6.92
Q86WC9	PG1 protein (Fragment) OS=Homo sapiens PE=2 SV=1 - [Q86WC9_HUMAN]	18.89	1	1	1	90	10.5	10.76
C9K0H7	Uncharacterized protein OS=Homo sapiens GN=RYR3 PE=4 SV=1 - [C9K0H7_HUMAN]	0.41	4	1	1	3,857	435.4	6.34
E7EUA2	Uncharacterized protein OS=Homo sapiens GN=SHANK2 PE=4 SV=1 - [E7EUA2_HUMAN]	1.28	5	1	1	1,253	134.7	5.60
A6NL68	Uncharacterized protein OS=Homo sapiens GN=SLC2A1 PE=3 SV=1 - [A6NL68_HUMAN]	2.99	1	1	1	234	25.2	9.80
A2MYC8	V5-2 protein (Fragment) OS=Homo sapiens GN=V5-2 PE=1 SV=1 - [A2MYC8_HUMAN]	9.62	2	1	1	104	11.0	7.28
B3KQB8	cDNA FLJ90160 fis, clone HEMBB1002661, highly similar to Hairy/enhancer-of-split related with YRPW motif 1 OS=Homo sapiens PE=2 SV=1 - [B3KQB8_HUMAN]	9.21	4	1	1	304	32.6	8.95
B7Z6Y2	cDNA FLJ54942, highly similar to Homo sapiens bridging integrator 1 (BIN1), transcript variant 10, mRNA OS=Homo sapiens PE=2 SV=1 - [B7Z6Y2_HUMAN]	2.60	5	1	1	385	43.1	5.33
B3KQK5	cDNA FLJ90621 fis, clone PLACE1002547, highly similar to Mitochondrial proteins import receptor OS=Homo sapiens PE=2 SV=1 - [B3KQK5_HUMAN]	6.76	3	1	1	281	31.6	5.57
A2J1N7	Rheumatoid factor RF-ET10 (Fragment) OS=Homo sapiens PE=2 SV=1 - [A2J1N7_HUMAN]	7.29	1	1	1	96	10.5	9.36
B4DDT8	cDNA FLJ54587, highly similar to Splicing factor, arginine/serine-rich 16 OS=Homo sapiens PE=2 SV=1 - [B4DDT8_HUMAN]	1.47	4	1	1	612	70.2	10.59
B5A940	Soluble MET variant 12 OS=Homo sapiens GN=MET PE=2 SV=1 - [B5A940_HUMAN]	2.21	1	1	1	408	46.5	6.93
B4DXH5	cDNA FLJ58435, highly similar to Myosin-5B OS=Homo sapiens PE=2 SV=1 - [B4DXH5_HUMAN]	1.68	3	1	1	537	62.1	6.14
B4DJF2	cDNA FLJ51975, moderately similar to 14-3-3 protein epsilon (14-3-3E) OS=Homo sapiens PE=4 SV=1 - [B4DJF2_HUMAN]	15.96	3	1	1	94	10.9	5.34
C0KZE1	Family with sequence similarity 33 member A (Fragment) OS=Homo sapiens GN=FAM33A PE=2 SV=1 - [C0KZE1_HUMAN]	20.93	2	1	1	43	4.5	5.78
B3KSR5	cDNA FLJ36828 fis, clone ASTRO2009333, highly similar to Homo sapiens debranching enzyme homolog 1 (S. cerevisiae) (DBR1), mRNA OS=Homo sapiens PE=2 SV=1 - [B3KSR5_HUMAN]	5.16	2	1	1	310	34.8	4.59
E9PCV4	Uncharacterized protein OS=Homo sapiens GN=SPHK2 PE=4 SV=1 - [E9PCV4_HUMAN]	2.44	1	1	1	616	64.8	7.71
Q4FEB5	Chymase 1 preproprotein transcript I (Fragment) OS=Homo sapiens GN=CMA1 PE=2 SV=1 - [Q4FEB5_HUMAN]	6.12	2	1	1	147	16.3	9.07
B7Z7Z9	cDNA FLJ58712, highly similar to Mus musculus myosin, heavy polypeptide 9, non-muscle (Myh9), transcript variant 1, mRNA OS=Homo sapiens PE=2 SV=1 - [B7Z7Z9_HUMAN]	1.61	2	1	1	498	56.3	5.71
Q8N863	cDNA FLJ39940 fis, clone SPLEN2022522 OS=Homo sapiens PE=2 SV=1 - [Q8N863_HUMAN]	10.86	1	1	1	175	18.2	7.25
P20933	N(4)-(beta-N-acetylglucosaminyl)-L-asparaginase OS=Homo sapiens GN=AGA PE=1 SV=2 - [ASPG_HUMAN]	2.31	1	1	1	346	37.2	6.28
Q9NPF2	Carbohydrate sulfotransferase 11 OS=Homo sapiens GN=CHST11 PE=1 SV=1 - [CHSTB_HUMAN]	5.40	1	1	1	352	41.5	8.85
P53420	Collagen alpha-4(IV) chain OS=Homo sapiens GN=COL4A4 PE=1 SV=3 - [CO4A4_HUMAN]	1.42	1	1	1	1,690	163.9	8.62
Q8IV53	DENN domain-containing protein 1C OS=Homo sapiens GN=DENND1C PE=1 SV=1 - [DEN1C_HUMAN]	1.37	1	1	1	801	87.0	5.67
Q96N67	Dedicator of cytokinesis protein 7 OS=Homo sapiens GN=DOCK7 PE=1 SV=4 - [DOCK7_HUMAN]	0.56	1	1	1	2,140	242.4	6.80
Q9P2D6	Protein FAM135A OS=Homo sapiens GN=FAM135A PE=1 SV=2 - [F135A_HUMAN]	0.46	1	1	1	1,515	169.7	5.39
Q8ND71	GTPase IMAP family member 8 OS=Homo sapiens GN=GIMAP8 PE=1 SV=2 - [GIMA8_HUMAN]	1.20	1	1	1	665	74.8	8.34
P40197	Platelet glycoprotein V OS=Homo sapiens GN=GP5 PE=1 SV=1 - [GPV_HUMAN]	2.68	1	1	1	560	60.9	9.63
P54257	Huntingtin-associated protein 1 OS=Homo sapiens GN=HAP1 PE=1 SV=3 - [HAP1_HUMAN]	1.34	1	1	1	671	75.5	4.70
P01815	Ig heavy chain V-II region COR OS=Homo sapiens PE=1 SV=1 - [HV202_HUMAN]	7.50	1	1	1	120	13.2	6.57
Q9P2K6	Kelch domain-containing protein 5 OS=Homo sapiens GN=KLHDC5 PE=1 SV=2 - [KLDC5_HUMAN]	1.39	1	1	1	505	56.8	5.74
P59901	Leukocyte immunoglobulin-like receptor subfamily A member 4 OS=Homo sapiens GN=LILRA4 PE=1 SV=2 - [LIRA4_HUMAN]	3.01	1	1	1	499	55.1	8.07
Q02978	Mitochondrial 2-oxoglutarate/malate carrier protein OS=Homo sapiens GN=SLC25A11 PE=1 SV=3 - [M2OM_HUMAN]	7.64	1	1	1	314	34.0	9.91
P35410	Mas-related G-protein coupled receptor MRG OS=Homo sapiens GN=MAS1L PE=2 SV=1 - [MAS1L_HUMAN]	1.85	2	1	1	378	42.4	8.32
Q9NU22	Midasin OS=Homo sapiens GN=MDN1 PE=1 SV=2 - [MDN1_HUMAN]	0.16	1	1	1	5,596	632.4	5.68
Q8N1T3	Myosin-Ih OS=Homo sapiens GN=MYO1H PE=2 SV=2 - [MYO1H_HUMAN]	0.68	1	1	1	1,032	119.0	9.09
P17568	NADH dehydrogenase [ubiquinone] 1 beta subcomplex subunit 7 OS=Homo sapiens GN=NDUFB7 PE=1 SV=4 - [NDUB7_HUMAN]	5.11	1	1	1	137	16.4	8.92
Q96SB3	Neurabin-2 OS=Homo sapiens GN=PPP1R9B PE=1 SV=2 - [NEB2_HUMAN]	3.31	2	1	1	815	89.1	4.97
Q8WWZ8	Oncoprotein-induced transcript 3 protein OS=Homo sapiens GN=OIT3 PE=1 SV=2 - [OIT3_HUMAN]	2.20	1	1	1	545	60.0	5.58
P27986	Phosphatidylinositol 3-kinase regulatory subunit alpha OS=Homo sapiens GN=PIK3R1 PE=1 SV=2 - [P85A_HUMAN]	1.52	1	1	1	724	83.5	6.16
Q9NWQ8	Phosphoprotein associated with glycosphingolipid-enriched microdomains 1 OS=Homo sapiens GN=PAG1 PE=1 SV=2 - [PAG1_HUMAN]	1.62	1	1	1	432	47.0	4.65
Q9Y5G2	Protocadherin gamma-B2 OS=Homo sapiens GN=PCDHGB2 PE=2 SV=1 - [PCDGE_HUMAN]	0.75	1	1	1	931	100.8	5.07
Q9UF12	Probable proline dehydrogenase 2 OS=Homo sapiens GN=PRODH2 PE=2 SV=1 - [PROD2_HUMAN]	1.31	2	1	1	536	58.8	8.60
Q7RTS3	Pancreas transcription factor 1 subunit alpha OS=Homo sapiens GN=PTF1A PE=1 SV=1 - [PTF1A_HUMAN]	1.83	1	1	1	328	34.9	5.25
A4D1S5	Ras-related protein Rab-19 OS=Homo sapiens GN=RAB19 PE=2 SV=2 - [RAB19_HUMAN]	5.07	1	1	1	217	24.4	6.52
A6NED2	RCC1 domain-containing protein 1 OS=Homo sapiens GN=RCCD1 PE=1 SV=1 - [RCCD1_HUMAN]	5.05	1	1	1	376	40.1	5.27
Q86YS3	Rab11 family-interacting protein 4 OS=Homo sapiens GN=RAB11FIP4 PE=1 SV=1 - [RFIP4_HUMAN]	1.10	1	1	1	637	71.9	4.87
Q15287	RNA-binding protein with serine-rich domain 1 OS=Homo sapiens GN=RNPS1 PE=1 SV=1 - [RNPS1_HUMAN]	2.62	1	1	1	305	34.2	11.84
P55017	Solute carrier family 12 member 3 OS=Homo sapiens GN=SLC12A3 PE=1 SV=3 - [S12A3_HUMAN]	0.98	1	1	1	1,021	113.1	7.88
Q63ZE4	Solute carrier family 22 member 10 OS=Homo sapiens GN=SLC22A10 PE=2 SV=2 - [S22AA_HUMAN]	1.66	1	1	1	541	60.2	8.56
O75094	Slit homolog 3 protein OS=Homo sapiens GN=SLIT3 PE=2 SV=3 - [SLIT3_HUMAN]	0.59	1	1	1	1,523	167.6	7.65
Q9UHJ3	Scm-like with four MBT domains protein 1 OS=Homo sapiens GN=SFMBT1 PE=1 SV=2 - [SMBT1_HUMAN]	1.27	1	1	1	866	98.1	6.21
Q9BXP5	Serrate RNA effector molecule homolog OS=Homo sapiens GN=SRRT PE=1 SV=1 - [SRRT_HUMAN]	1.71	1	1	1	876	100.6	5.96
P24821	Tenascin OS=Homo sapiens GN=TNC PE=1 SV=3 - [TENA_HUMAN]	1.14	2	1	1	2,201	240.7	4.89
Q16473	Putative tenascin-XA OS=Homo sapiens GN=TNXA PE=5 SV=2 - [TENXA_HUMAN]	4.18	11	1	1	311	33.7	5.50
Q8N6V9	Testis-expressed sequence 9 protein OS=Homo sapiens GN=TEX9 PE=2 SV=1 - [TEX9_HUMAN]	2.05	1	1	1	391	44.8	6.60
Q7Z2Z1	Treslin OS=Homo sapiens GN=TICRR PE=1 SV=2 - [TICRR_HUMAN]	0.37	1	1	1	1,910	210.7	8.78
Q6ZQQ6	WD repeat-containing protein 87 OS=Homo sapiens GN=WDR87 PE=2 SV=3 - [WDR87_HUMAN]	0.24	2	1	1	2,873	333.0	7.28
Q5T619	Zinc finger protein 648 OS=Homo sapiens GN=ZNF648 PE=1 SV=1 - [ZN648_HUMAN]	2.11	1	1	1	568	62.3	8.62
B1AL55	EDAR-associated death domain (Fragment) OS=Homo sapiens GN=EDARADD PE=4 SV=1 - [B1AL55_HUMAN]	11.90	3	1	1	84	9.3	4.22
B0QYC4	Caspase recruitment domain family, member 10 (Fragment) OS=Homo sapiens GN=CARD10 PE=4 SV=1 - [B0QYC4_HUMAN]	1.67	4	1	1	480	53.1	7.58
B1AL01	Bone morphogenetic protein 7 (Osteogenic protein 1) (Fragment) OS=Homo sapiens GN=BMP7 PE=3 SV=1 - [B1AL01_HUMAN]	2.36	5	1	1	296	34.1	7.81
B4E1A2	Gamma-aminobutyric acid (GABA) A receptor, alpha 5, isoform CRA_a OS=Homo sapiens GN=GABRA5 PE=2 SV=1 - [B4E1A2_HUMAN]	1.72	3	1	1	348	39.0	9.47
Q7Z2I3	L1 cell adhesion molecule (Fragment) OS=Homo sapiens GN=L1CAM PE=2 SV=1 - [Q7Z2I3_HUMAN]	9.86	6	1	1	71	7.8	4.72
Q6ZV91	HCG1647356, isoform CRA_b OS=Homo sapiens GN=hCG_1647356 PE=2 SV=1 - [Q6ZV91_HUMAN]	12.16	1	1	1	148	15.3	9.64
D3DUT9	CD163 antigen-like 1, isoform CRA_a OS=Homo sapiens GN=CD163L1 PE=4 SV=1 - [D3DUT9_HUMAN]	1.32	5	1	1	1,061	116.2	6.00
D3DS02	HCG2044783, isoform CRA_a OS=Homo sapiens GN=hCG_2044783 PE=4 SV=1 - [D3DS02_HUMAN]	3.72	1	1	1	188	21.1	11.80
D6RAF2	Uncharacterized protein OS=Homo sapiens GN=PAPD4 PE=4 SV=1 - [D6RAF2_HUMAN]	2.49	2	1	1	441	51.0	9.23
E7EW21	Uncharacterized protein OS=Homo sapiens GN=SHF PE=4 SV=1 - [E7EW21_HUMAN]	3.30	6	1	1	212	23.4	4.70
C9JBA5	Uncharacterized protein OS=Homo sapiens GN=IGSF11 PE=4 SV=1 - [C9JBA5_HUMAN]	1.74	3	1	1	403	43.4	7.11
Q59GF0	Thyroglobulin variant (Fragment) OS=Homo sapiens PE=2 SV=1 - [Q59GF0_HUMAN]	0.44	1	1	1	1,574	173.0	6.05
Q5NV70	V1-11 protein (Fragment) OS=Homo sapiens GN=V1-11 PE=4 SV=1 - [Q5NV70_HUMAN]	16.49	2	1	1	97	10.3	4.59
B4DSM3	cDNA FLJ56943, highly similar to Protocadherin-11 X-linked (Fragment) OS=Homo sapiens PE=2 SV=1 - [B4DSM3_HUMAN]	1.56	6	1	1	897	98.9	4.87
B2R9T9	cDNA, FLJ94551 OS=Homo sapiens PE=2 SV=1 - [B2R9T9_HUMAN]	2.47	6	1	1	243	26.2	10.67
E7ESA4	Uncharacterized protein OS=Homo sapiens GN=CNKSR2 PE=4 SV=1 - [E7ESA4_HUMAN]	0.94	3	1	1	848	96.2	6.61
E9PRQ2	Uncharacterized protein OS=Homo sapiens GN=MAP3K11 PE=4 SV=1 - [E9PRQ2_HUMAN]	20.72	1	1	1	111	12.3	4.92
Q8TD77	TAP1 (Fragment) OS=Homo sapiens GN=TAP1 PE=4 SV=1 - [Q8TD77_HUMAN]	28.89	1	1	1	45	4.6	6.46
B4DGK0	cDNA FLJ55052, highly similar to Ubiquitin protein ligase Praja1 (EC 6.3.2.-) OS=Homo sapiens PE=2 SV=1 - [B4DGK0_HUMAN]	1.61	1	1	1	558	61.3	4.89
B3KU02	cDNA FLJ39019 fis, clone NT2RP7002738, highly similar to Homo sapiens sorting nexin 26 (SNX26), mRNA OS=Homo sapiens PE=2 SV=1 - [B3KU02_HUMAN]	1.42	2	1	1	1,126	120.9	9.20
P78512	Dual specificity protein phosphatase homolog hMKP-R (Fragment) OS=Homo sapiens PE=2 SV=1 - [P78512_HUMAN]	42.65	3	1	1	68	7.6	7.28
E5RFS1	Uncharacterized protein OS=Homo sapiens GN=COPS5 PE=4 SV=1 - [E5RFS1_HUMAN]	52.63	4	1	1	57	6.3	5.19
B9EGA3	SMARCD2 protein OS=Homo sapiens GN=SMARCD2 PE=2 SV=1 - [B9EGA3_HUMAN]	4.05	3	1	1	494	55.8	9.58
Q69YU9	Putative uncharacterized protein DKFZp761I2114 (Fragment) OS=Homo sapiens GN=DKFZp761I2114 PE=4 SV=1 - [Q69YU9_HUMAN]	16.30	1	1	1	92	10.3	4.34
Q69YS5	Zinc finger protein 41 homolog (Zfp-41). (Fragment) OS=Homo sapiens GN=DKFZp761O1618 PE=2 SV=1 - [Q69YS5_HUMAN]	4.63	1	1	1	540	58.6	9.23
B7ZKP4	ASTN2 protein OS=Homo sapiens GN=ASTN2 PE=2 SV=1 - [B7ZKP4_HUMAN]	1.87	8	1	1	375	42.2	5.71
C9JYI7	Uncharacterized protein OS=Homo sapiens GN=SMARCD3 PE=4 SV=1 - [C9JYI7_HUMAN]	24.46	4	1	1	139	15.1	11.46
Q6L640	ABO glycosyltransferase (Fragment) OS=Homo sapiens GN=ABO PE=2 SV=1 - [Q6L640_HUMAN]	10.48	1	1	1	229	26.8	7.96
B3KY11	cDNA FLJ46571 fis, clone THYMU3041428, highly similar to Probable ATP-dependent RNA helicase DDX23 (EC 3.6.1.-) OS=Homo sapiens PE=2 SV=1 - [B3KY11_HUMAN]	1.13	3	1	1	800	93.2	9.50
O95333	Putative uncharacterized protein (Fragment) OS=Homo sapiens PE=2 SV=1 - [O95333_HUMAN]	6.14	5	1	1	114	12.7	6.05
B7Z4X9	cDNA FLJ53632, highly similar to SAP30-binding protein OS=Homo sapiens PE=2 SV=1 - [B7Z4X9_HUMAN]	7.74	2	1	1	168	18.7	4.35
A8K5G6	cDNA FLJ78646, highly similar to Homo sapiens polymerase (DNA directed), gamma (POLG), mRNA OS=Homo sapiens PE=2 SV=1 - [A8K5G6_HUMAN]	2.10	5	1	1	1,239	139.5	6.84
B4DYX1	cDNA FLJ57672, highly similar to Homo sapiens MORC family CW-type zinc finger 1 (MORC1), mRNA OS=Homo sapiens PE=2 SV=1 - [B4DYX1_HUMAN]	0.73	3	1	1	963	110.5	7.75
P78328	Sperm-associated antigen 10 (Protein HP47). (Fragment) OS=Homo sapiens GN=SPAG10 PE=2 SV=1 - [P78328_HUMAN]	15.38	1	1	1	78	8.7	7.05
Q5NV88	V1-22 protein (Fragment) OS=Homo sapiens GN=V1-22 PE=4 SV=1 - [Q5NV88_HUMAN]	8.16	2	1	1	98	10.6	4.78
D6RI04	Uncharacterized protein OS=Homo sapiens GN=SMUG1 PE=4 SV=1 - [D6RI04_HUMAN]	9.43	1	1	1	159	17.6	4.84
A6NLT1	Uncharacterized protein OS=Homo sapiens GN=MAN1C1 PE=4 SV=3 - [A6NLT1_HUMAN]	2.21	6	1	1	362	40.7	6.24
B7Z5T8	cDNA FLJ58750, highly similar to Carnitine O-palmitoyltransferase I, muscle isoform (EC 2.3.1.21) OS=Homo sapiens PE=2 SV=1 - [B7Z5T8_HUMAN]	1.01	6	1	1	691	78.8	9.22
Q4G0K7	GPR116 protein (Fragment) OS=Homo sapiens GN=GPR116 PE=2 SV=1 - [Q4G0K7_HUMAN]	0.98	6	1	1	717	79.1	7.96
Q59GB7	Solute carrier family 12 (Sodium/potassium/chloride transporters), member 2 variant (Fragment) OS=Homo sapiens PE=2 SV=1 - [Q59GB7_HUMAN]	8.20	1	1	1	122	14.0	9.67
Q9UDQ3	Calcium channel; match to P54289 (PID:g1705852) (Fragment) OS=Homo sapiens GN=WUGSC:H_DJ0560O14.1 PE=2 SV=1 - [Q9UDQ3_HUMAN]	1.34	4	1	1	745	84.3	5.31
E9PL61	Uncharacterized protein OS=Homo sapiens GN=DEPDC1 PE=4 SV=1 - [E9PL61_HUMAN]	26.47	1	1	1	34	3.8	8.98
Q5JWR0	RanBP-type and C3HC4-type zinc finger containing 1 (Fragment) OS=Homo sapiens GN=RBCK1 PE=2 SV=2 - [Q5JWR0_HUMAN]	3.15	5	1	1	254	28.3	5.53
B4DNU0	cDNA FLJ58492, highly similar to TFIIH basal transcription factor complex p52 subunit OS=Homo sapiens PE=2 SV=1 - [B4DNU0_HUMAN]	2.85	3	1	1	386	43.3	8.76
B7ZLP8	TARSL2 protein OS=Homo sapiens GN=TARSL2 PE=2 SV=1 - [B7ZLP8_HUMAN]	1.31	2	1	1	766	88.7	5.81
C9JE82	Uncharacterized protein OS=Homo sapiens GN=CACNA2D2 PE=4 SV=1 - [C9JE82_HUMAN]	0.79	5	1	1	1,144	129.1	5.74
C9IZP8	Uncharacterized protein OS=Homo sapiens GN=C1S PE=4 SV=1 - [C9IZP8_HUMAN]	16.50	1	1	1	103	11.8	4.20
Q8WUM7	KANK1 protein (Fragment) OS=Homo sapiens GN=KANK1 PE=2 SV=1 - [Q8WUM7_HUMAN]	1.28	8	1	1	784	84.1	4.83
A9XA84	Alpha-helix coiled-coil rod homologue (Fragment) OS=Homo sapiens GN=HCR PE=4 SV=1 - [A9XA84_HUMAN]	16.24	4	1	1	117	13.3	5.19
A8K6X9	cDNA FLJ76427, highly similar to Homo sapiens SH2 domain binding protein 1 (tetratricopeptide repeat containing) (SH2BP1), mRNA OS=Homo sapiens PE=2 SV=1 - [A8K6X9_HUMAN]	0.51	2	1	1	1,173	133.4	6.77
B4DS56	cDNA FLJ58937 OS=Homo sapiens PE=2 SV=1 - [B4DS56_HUMAN]	17.34	1	1	1	173	17.9	9.80
B4DYT2	cDNA FLJ60197 OS=Homo sapiens PE=2 SV=1 - [B4DYT2_HUMAN]	4.12	1	1	1	243	27.3	8.43
B4DXS8	cDNA FLJ50911, moderately similar to Homo sapiens hepatoma derived growth factor-like 1 (HDGFL1), mRNA OS=Homo sapiens PE=2 SV=1 - [B4DXS8_HUMAN]	6.25	2	1	1	176	18.9	4.40
B4E006	cDNA FLJ53119, highly similar to ADP-ribosyl cyclase 1 (EC 3.2.2.5) OS=Homo sapiens PE=2 SV=1 - [B4E006_HUMAN]	5.00	3	1	1	160	17.9	8.97
Q5SPY4	ATP-binding cassette, sub-family A (ABC1), member 2 (Fragment) OS=Homo sapiens GN=ABCA2 PE=2 SV=1 - [Q5SPY4_HUMAN]	14.63	4	1	1	123	13.7	9.73
B3KPD3	cDNA FLJ31633 fis, clone NT2RI2003407, highly similar to Inner centromere protein (Fragment) OS=Homo sapiens PE=2 SV=1 - [B3KPD3_HUMAN]	0.93	2	1	1	752	86.7	9.52
B4DXK3	cDNA FLJ58959, highly similar to Ataxin-7-like protein 1 OS=Homo sapiens PE=2 SV=1 - [B4DXK3_HUMAN]	1.98	5	1	1	303	32.3	9.83
Q05C17	BTAF1 protein (Fragment) OS=Homo sapiens GN=BTAF1 PE=2 SV=1 - [Q05C17_HUMAN]	2.50	2	1	1	560	62.9	6.04
E7EXB3	Uncharacterized protein OS=Homo sapiens GN=BHMT PE=4 SV=1 - [E7EXB3_HUMAN]	4.11	3	1	1	365	40.5	7.15
C9JP63	Uncharacterized protein OS=Homo sapiens GN=ITSN2 PE=4 SV=1 - [C9JP63_HUMAN]	1.02	11	1	1	591	67.0	9.31
Q59G20	Beta isoform of regulatory subunit B56, protein phosphatase 2A variant (Fragment) OS=Homo sapiens PE=2 SV=1 - [Q59G20_HUMAN]	1.90	1	1	1	369	41.2	6.68
Q4G118	LOC649305 protein (Fragment) OS=Homo sapiens GN=LOC649305 PE=2 SV=1 - [Q4G118_HUMAN]	3.19	1	1	1	188	19.9	11.02
A0JD09	HADV14S1 (Fragment) OS=Homo sapiens GN=hADV14S1 PE=4 SV=1 - [A0JD09_HUMAN]	6.84	1	1	1	117	13.0	5.06
D3JV41	Thrombocidin-2 antimicrobial variant (Fragment) OS=Homo sapiens PE=4 SV=1 - [D3JV41_HUMAN]	10.32	2	1	1	126	13.7	9.00
Q5VW31	Nuclear factor I/B OS=Homo sapiens GN=NFIB PE=2 SV=1 - [Q5VW31_HUMAN]	5.36	9	1	1	168	18.3	9.01
A4D211	Similar to mesenchymal stem cell protein DSC92; neurite outgrowth associated protein OS=Homo sapiens GN=LOC402245 PE=4 SV=1 - [A4D211_HUMAN]	20.63	1	1	1	126	13.8	8.75
E9PQT1	Uncharacterized protein OS=Homo sapiens GN=FGD4 PE=4 SV=1 - [E9PQT1_HUMAN]	9.66	6	1	1	207	22.3	4.79
C9JUP5	Uncharacterized protein OS=Homo sapiens GN=MYO1B PE=4 SV=1 - [C9JUP5_HUMAN]	9.40	7	1	1	149	16.8	5.17
Q9H7M5	FLJ00045 protein (Fragment) OS=Homo sapiens GN=FLJ00045 PE=2 SV=1 - [Q9H7M5_HUMAN]	2.21	7	1	1	272	31.5	5.43
A8K1N8	cDNA FLJ78365 OS=Homo sapiens PE=2 SV=1 - [A8K1N8_HUMAN]	0.81	2	1	1	740	87.0	5.38
B4E178	cDNA FLJ52386, highly similar to Bardet-Biedl syndrome 4 protein OS=Homo sapiens PE=2 SV=1 - [B4E178_HUMAN]	1.73	7	1	1	347	38.3	6.87
B3KQU2	cDNA PSEC0179 fis, clone OVARC1001209, highly similar to Protein disulfide-isomerase A2 OS=Homo sapiens PE=2 SV=1 - [B3KQU2_HUMAN]	3.24	1	1	1	494	53.8	5.10
B4DRF5	cDNA FLJ60335, highly similar to Homo sapiens IQ motif containing C (IQCC), mRNA OS=Homo sapiens PE=2 SV=1 - [B4DRF5_HUMAN]	2.19	2	1	1	366	41.4	8.62
C9JUF6	Uncharacterized protein OS=Homo sapiens GN=SLITRK6 PE=4 SV=1 - [C9JUF6_HUMAN]	1.52	2	1	1	395	43.8	7.11
E9PND4	Uncharacterized protein OS=Homo sapiens GN=HLA-DOB PE=4 SV=1 - [E9PND4_HUMAN]	6.49	6	1	1	154	17.4	5.60
B2RTS2	ARAP1 protein OS=Homo sapiens GN=ARAP1 PE=2 SV=1 - [B2RTS2_HUMAN]	1.50	4	1	1	1,133	127.6	6.14
Q9UH66	7h3 protein (Fragment) OS=Homo sapiens PE=2 SV=1 - [Q9UH66_HUMAN]	3.33	1	1	1	450	48.9	7.71
Q9BSC0	CEBPB protein (Fragment) OS=Homo sapiens GN=CEBPB PE=2 SV=2 - [Q9BSC0_HUMAN]	4.38	9	1	1	137	14.9	9.80
B0QYM9	Parvin, gamma (Fragment) OS=Homo sapiens GN=PARVG PE=4 SV=1 - [B0QYM9_HUMAN]	53.85	4	1	1	26	3.0	4.08
Q495S5	EMILIN3 protein OS=Homo sapiens GN=EMILIN3 PE=2 SV=1 - [Q495S5_HUMAN]	6.72	2	1	1	372	39.6	5.15
Q6P1M9	Armadillo repeat-containing X-linked protein 5 OS=Homo sapiens GN=ARMCX5 PE=2 SV=1 - [ARMX5_HUMAN]	7.89	1	1	1	558	62.3	8.73
Q8N0T5	Putative uncharacterized protein C2orf58 OS=Homo sapiens GN=C2orf58 PE=5 SV=1 - [CB058_HUMAN]	19.01	1	1	1	142	15.8	9.09
Q8NFR7	Coiled-coil domain-containing protein 148 OS=Homo sapiens GN=CCDC148 PE=2 SV=2 - [CC148_HUMAN]	1.35	2	1	1	591	71.0	8.87
Q9P232	Contactin-3 OS=Homo sapiens GN=CNTN3 PE=1 SV=3 - [CNTN3_HUMAN]	1.07	1	1	1	1,028	112.8	6.30
O75128	Protein cordon-bleu OS=Homo sapiens GN=COBL PE=1 SV=2 - [COBL_HUMAN]	0.63	1	1	1	1,261	135.5	7.75
P24903	Cytochrome P450 2F1 OS=Homo sapiens GN=CYP2F1 PE=1 SV=2 - [CP2F1_HUMAN]	1.22	1	1	1	491	55.5	7.37
P51808	Dynein light chain Tctex-type 3 OS=Homo sapiens GN=DYNLT3 PE=1 SV=1 - [DYLT3_HUMAN]	14.66	2	1	1	116	13.1	5.66
P49411	Elongation factor Tu, mitochondrial OS=Homo sapiens GN=TUFM PE=1 SV=2 - [EFTU_HUMAN]	1.55	1	1	1	452	49.5	7.61
Q6UWV6	Ectonucleotide pyrophosphatase/phosphodiesterase family member 7 OS=Homo sapiens GN=ENPP7 PE=1 SV=3 - [ENPP7_HUMAN]	4.59	1	1	1	458	51.5	6.89
Q9BXW9	Fanconi anemia group D2 protein OS=Homo sapiens GN=FANCD2 PE=1 SV=1 - [FACD2_HUMAN]	1.02	1	1	1	1,471	166.4	6.24
Q5D862	Filaggrin-2 OS=Homo sapiens GN=FLG2 PE=1 SV=1 - [FILA2_HUMAN]	0.50	1	1	1	2,391	247.9	8.31
Q8IZF6	Probable G-protein coupled receptor 112 OS=Homo sapiens GN=GPR112 PE=2 SV=2 - [GP112_HUMAN]	0.26	1	1	1	3,080	333.2	6.21
Q92917	G patch domain and KOW motifs-containing protein OS=Homo sapiens GN=GPKOW PE=1 SV=2 - [GPKOW_HUMAN]	1.26	1	1	1	476	52.2	6.15
P60893	Probable G-protein coupled receptor 85 OS=Homo sapiens GN=GPR85 PE=2 SV=1 - [GPR85_HUMAN]	2.97	2	1	1	370	42.0	9.66
O60269	G protein-regulated inducer of neurite outgrowth 2 OS=Homo sapiens GN=GPRIN2 PE=1 SV=2 - [GRIN2_HUMAN]	1.53	1	1	1	458	47.4	6.74
Q9ULI3	Protein HEG homolog 1 OS=Homo sapiens GN=HEG1 PE=1 SV=3 - [HEG1_HUMAN]	0.72	1	1	1	1,381	147.4	6.18
Q6UWX4	HHIP-like protein 2 OS=Homo sapiens GN=HHIPL2 PE=2 SV=1 - [HIPL2_HUMAN]	1.24	1	1	1	724	80.7	9.01
Q7LGA3	Heparan sulfate 2-O-sulfotransferase 1 OS=Homo sapiens GN=HS2ST1 PE=1 SV=1 - [HS2ST_HUMAN]	1.69	1	1	1	356	41.9	8.69
Q15323	Keratin, type I cuticular Ha1 OS=Homo sapiens GN=KRT31 PE=1 SV=3 - [K1H1_HUMAN]	5.05	1	1	1	416	47.2	4.88
P01611	Ig kappa chain V-I region Wes OS=Homo sapiens PE=1 SV=1 - [KV119_HUMAN]	16.67	1	1	1	108	11.6	7.28
Q9P2V4	Leucine-rich repeat, immunoglobulin-like domain and transmembrane domain-containing protein 1 OS=Homo sapiens GN=LRIT1 PE=1 SV=1 - [LRIT1_HUMAN]	1.77	1	1	1	623	68.0	8.54
Q96EZ8	Microspherule protein 1 OS=Homo sapiens GN=MCRS1 PE=1 SV=1 - [MCRS1_HUMAN]	2.60	1	1	1	462	51.8	9.38
Q16819	Meprin A subunit alpha OS=Homo sapiens GN=MEP1A PE=1 SV=2 - [MEP1A_HUMAN]	2.14	2	1	1	746	84.4	5.72
P09238	Stromelysin-2 OS=Homo sapiens GN=MMP10 PE=1 SV=1 - [MMP10_HUMAN]	5.25	1	1	1	476	54.1	5.80
Q9Y5G7	Protocadherin gamma-A6 OS=Homo sapiens GN=PCDHGA6 PE=2 SV=1 - [PCDG6_HUMAN]	0.97	2	1	1	932	100.8	4.88
O76038	Secretagogin OS=Homo sapiens GN=SCGN PE=1 SV=2 - [SEGN_HUMAN]	3.62	1	1	1	276	32.0	5.41
Q8N196	Homeobox protein SIX5 OS=Homo sapiens GN=SIX5 PE=1 SV=3 - [SIX5_HUMAN]	1.35	1	1	1	739	74.5	4.96
Q9BVQ7	Spermatogenesis-associated protein 5-like protein 1 OS=Homo sapiens GN=SPATA5L1 PE=1 SV=2 - [SPA5L_HUMAN]	1.06	1	1	1	753	80.7	8.09
Q7L8C5	Synaptotagmin-13 OS=Homo sapiens GN=SYT13 PE=1 SV=1 - [SYT13_HUMAN]	2.58	1	1	1	426	46.9	7.66
A8MW99	UPF0623 protein OS=Homo sapiens PE=3 SV=1 - [U623_HUMAN]	2.33	1	1	1	386	44.2	6.16
P55089	Urocortin OS=Homo sapiens GN=UCN PE=1 SV=1 - [UCN1_HUMAN]	5.65	3	1	1	124	13.5	11.71
O75385	Serine/threonine-protein kinase ULK1 OS=Homo sapiens GN=ULK1 PE=1 SV=2 - [ULK1_HUMAN]	0.67	1	1	1	1,050	112.6	8.79
Q96A05	V-type proton ATPase subunit E 2 OS=Homo sapiens GN=ATP6V1E2 PE=1 SV=1 - [VATE2_HUMAN]	3.98	1	1	1	226	26.1	8.76
Q9C0J8	WD repeat-containing protein 33 OS=Homo sapiens GN=WDR33 PE=1 SV=2 - [WDR33_HUMAN]	2.47	1	1	1	1,336	145.8	9.17
Q8IZH2	5'-3' exoribonuclease 1 OS=Homo sapiens GN=XRN1 PE=1 SV=1 - [XRN1_HUMAN]	0.41	1	1	1	1,706	194.0	7.21
Q9BX82	Zinc finger protein 471 OS=Homo sapiens GN=ZNF471 PE=2 SV=1 - [ZN471_HUMAN]	0.96	1	1	1	626	73.0	8.56
Q6ZR52	Zinc finger protein 493 OS=Homo sapiens GN=ZNF493 PE=2 SV=3 - [ZN493_HUMAN]	1.55	2	1	1	646	75.3	9.31
E5KLK2	Mitochondrial dynamin-like 120 kDa protein OS=Homo sapiens GN=OPA1 PE=3 SV=1 - [E5KLK2_HUMAN]	0.87	12	1	1	924	107.5	7.99
E5KN59	Peptidyl-prolyl cis-trans isomerase D OS=Homo sapiens PE=4 SV=1 - [E5KN59_HUMAN]	2.43	3	1	1	370	40.7	7.21
B2RPC0	C6orf138 protein OS=Homo sapiens GN=C6orf138 PE=2 SV=1 - [B2RPC0_HUMAN]	2.16	3	1	1	510	58.6	7.64
A0N4X5	HCG2039756 (Fragment) OS=Homo sapiens GN=Tcr-alpha PE=4 SV=1 - [A0N4X5_HUMAN]	42.11	1	1	1	19	2.2	8.84
D3DS91	A kinase (PRKA) anchor protein 6, isoform CRA_b OS=Homo sapiens GN=AKAP6 PE=4 SV=1 - [D3DS91_HUMAN]	0.34	3	1	1	2,077	229.3	5.10
B0QZK4	Heterochromatin protein 1, binding protein 3 (Fragment) OS=Homo sapiens GN=HP1BP3 PE=3 SV=1 - [B0QZK4_HUMAN]	3.95	4	1	1	253	28.5	9.88
B7ZB84	Like-glycosyltransferase (Fragment) OS=Homo sapiens GN=LARGE PE=4 SV=1 - [B7ZB84_HUMAN]	3.99	6	1	1	276	32.4	5.74
D3DU86	TNF receptor-associated factor 7, isoform CRA_c OS=Homo sapiens GN=TRAF7 PE=4 SV=1 - [D3DU86_HUMAN]	1.05	6	1	1	570	63.9	6.95
Q6U836	Epithelial cell transforming 2 OS=Homo sapiens GN=ECT2 PE=2 SV=1 - [Q6U836_HUMAN]	1.59	3	1	1	882	99.9	7.61
E5RFY2	Uncharacterized protein OS=Homo sapiens GN=KIAA0146 PE=4 SV=1 - [E5RFY2_HUMAN]	8.60	8	1	1	93	10.3	9.00
C9J3Y5	Uncharacterized protein OS=Homo sapiens GN=FAM72D PE=4 SV=1 - [C9J3Y5_HUMAN]	4.03	1	1	1	149	16.5	8.19
O19731	MHC HLA-DR-beta cell surface glycoprotein (Fragment) OS=Homo sapiens GN=HLA-DRB PE=4 SV=1 - [O19731_HUMAN]	82.76	1	1	1	29	2.9	8.00
B4DYR8	cDNA FLJ56473, highly similar to Zinc finger and BTB domain-containing protein 38 OS=Homo sapiens PE=2 SV=1 - [B4DYR8_HUMAN]	0.75	1	1	1	1,196	134.2	8.03
B7WP93	Uncharacterized protein OS=Homo sapiens GN=DNAH10 PE=4 SV=2 - [B7WP93_HUMAN]	1.62	3	1	1	617	70.1	6.04
E9PEZ6	Uncharacterized protein OS=Homo sapiens GN=WDR52 PE=4 SV=1 - [E9PEZ6_HUMAN]	1.50	4	1	1	401	47.5	5.68
Q6IBJ0	ORC3L protein OS=Homo sapiens GN=ORC3L PE=2 SV=1 - [Q6IBJ0_HUMAN]	2.84	6	1	1	387	45.3	7.94
B4DPQ5	cDNA FLJ51521, highly similar to Cytochrome P450 3A4 (EC 1.14.13.67) OS=Homo sapiens PE=2 SV=1 - [B4DPQ5_HUMAN]	4.95	11	1	1	323	37.1	6.93
A4PET2	AlkB, alkylation repair homolog 2 isoform 1 OS=Homo sapiens GN=ALKBH2 PE=2 SV=1 - [A4PET2_HUMAN]	5.73	1	1	1	157	17.1	6.57
A4D255	Putative uncharacterized protein MGC26484 OS=Homo sapiens GN=MGC26484 PE=4 SV=1 - [A4D255_HUMAN]	2.69	1	1	1	520	59.4	8.40
Q8NF05	FLJ00399 protein (Fragment) OS=Homo sapiens GN=FLJ00399 PE=2 SV=1 - [Q8NF05_HUMAN]	5.45	1	1	1	165	18.0	11.75
Q96BE0	Putative uncharacterized protein (Fragment) OS=Homo sapiens PE=2 SV=1 - [Q96BE0_HUMAN]	2.60	1	1	1	269	29.3	4.88
B3KMZ6	cDNA FLJ13058 fis, clone NT2RP3001587, highly similar to Ubiquitin-like 1-activating enzyme E1B OS=Homo sapiens PE=2 SV=1 - [B3KMZ6_HUMAN]	3.28	1	1	1	640	71.1	5.29
B3KUK8	cDNA FLJ40107 fis, clone TESTI2006677 OS=Homo sapiens PE=2 SV=1 - [B3KUK8_HUMAN]	4.49	2	1	1	356	40.6	8.40
Q59GS5	Paxillin variant (Fragment) OS=Homo sapiens PE=2 SV=1 - [Q59GS5_HUMAN]	1.68	1	1	1	713	75.5	5.10
Q6MZK0	Putative uncharacterized protein DKFZp686F02202 OS=Homo sapiens GN=DKFZp686F02202 PE=2 SV=1 - [Q6MZK0_HUMAN]	4.84	5	1	1	351	38.3	4.54
B7Z7G7	cDNA FLJ52026, highly similar to Vacuolar protein sorting-associated protein 45 OS=Homo sapiens PE=2 SV=1 - [B7Z7G7_HUMAN]	3.03	7	1	1	198	23.0	6.06
B4DPP1	cDNA FLJ54605, highly similar to UDP-glucuronosyltransferase 2B10 (EC 2.4.1.17) OS=Homo sapiens PE=2 SV=1 - [B4DPP1_HUMAN]	3.38	4	1	1	444	50.7	9.20
E9PRI1	Uncharacterized protein OS=Homo sapiens GN=MUS81 PE=4 SV=1 - [E9PRI1_HUMAN]	1.68	4	1	1	476	52.3	8.84
C9JDC4	Uncharacterized protein OS=Homo sapiens GN=DHX30 PE=4 SV=1 - [C9JDC4_HUMAN]	3.04	1	1	1	362	39.2	9.04
Q4VB96	EPB42 protein OS=Homo sapiens GN=EPB42 PE=2 SV=1 - [Q4VB96_HUMAN]	2.51	4	1	1	358	39.5	6.54
B0AZV7	cDNA, FLJ79547, highly similar to T-cell surface glycoprotein CD4 OS=Homo sapiens PE=2 SV=1 - [B0AZV7_HUMAN]	2.87	3	1	1	279	31.2	9.79
B3KVI8	cDNA FLJ16604 fis, clone TESTI4008097, highly similar to Polycomb group protein ASXL1 OS=Homo sapiens PE=2 SV=1 - [B3KVI8_HUMAN]	0.68	6	1	1	1,462	157.9	5.96
B4E0R8	cDNA FLJ60210 OS=Homo sapiens PE=2 SV=1 - [B4E0R8_HUMAN]	3.47	1	1	1	202	23.1	7.97
C9JWB5	Uncharacterized protein OS=Homo sapiens PE=4 SV=1 - [C9JWB5_HUMAN]	4.79	1	1	1	188	19.6	10.52
A5D8T4	TNFRSF8 protein (Fragment) OS=Homo sapiens GN=TNFRSF8 PE=2 SV=1 - [A5D8T4_HUMAN]	3.21	3	1	1	187	20.4	5.20
B3KX42	cDNA FLJ44712 fis, clone BRACE3019941, highly similar to Intraflagellar transport 88 homolog OS=Homo sapiens PE=2 SV=1 - [B3KX42_HUMAN]	2.14	3	1	1	795	90.7	8.56
E5RI61	Uncharacterized protein OS=Homo sapiens GN=KIAA0802 PE=4 SV=1 - [E5RI61_HUMAN]	0.58	3	1	1	1,545	173.2	5.95
B4DKW4	cDNA FLJ58541, highly similar to WD repeat protein 23 OS=Homo sapiens PE=2 SV=1 - [B4DKW4_HUMAN]	3.87	4	1	1	413	47.0	7.50
B3KRB3	cDNA FLJ33967 fis, clone DFNES2000913, highly similar to Synapsin-2 OS=Homo sapiens PE=2 SV=1 - [B3KRB3_HUMAN]	7.42	6	1	1	256	27.4	8.28
B4DH92	cDNA FLJ58118, highly similar to Leucine-rich repeat-containing G-protein coupled receptor 5 OS=Homo sapiens PE=2 SV=1 - [B4DH92_HUMAN]	2.57	4	1	1	777	85.7	6.99
C9JUI2	Protein Wnt OS=Homo sapiens GN=WNT2 PE=3 SV=1 - [C9JUI2_HUMAN]	8.50	1	1	1	153	17.6	8.43
B5A955	Soluble CSF1R variant 1 OS=Homo sapiens GN=CSF1R PE=2 SV=1 - [B5A955_HUMAN]	5.56	8	1	1	306	33.2	7.75
Q8NF76	FLJ00287 protein (Fragment) OS=Homo sapiens GN=FLJ00287 PE=2 SV=1 - [Q8NF76_HUMAN]	12.90	1	1	1	186	20.2	8.37
Q8WZ16	Putative uncharacterized protein OS=Homo sapiens PE=2 SV=1 - [Q8WZ16_HUMAN]	6.63	1	1	1	166	18.2	9.36
B4DVQ2	cDNA FLJ60909, highly similar to Proline-rich protein 8 OS=Homo sapiens PE=2 SV=1 - [B4DVQ2_HUMAN]	1.87	2	1	1	854	93.9	10.84
D5HR47	PUM1 fusion protein OS=Homo sapiens PE=2 SV=1 - [D5HR47_HUMAN]	3.68	7	1	1	516	54.6	5.24
Q9Y4Z9	FANCA protein (Fragment) OS=Homo sapiens GN=FANCA PE=4 SV=1 - [Q9Y4Z9_HUMAN]	93.33	1	1	1	15	1.6	8.03
A1XP52	Catecholamine-regulated protein 40 OS=Homo sapiens PE=2 SV=1 - [A1XP52_HUMAN]	4.86	5	1	1	350	38.1	5.39
A6NNS3	Uncharacterized protein OS=Homo sapiens GN=DOC2B PE=4 SV=2 - [A6NNS3_HUMAN]	5.84	2	1	1	308	33.9	7.43
B5MC62	Uncharacterized protein OS=Homo sapiens GN=AGR3 PE=4 SV=1 - [B5MC62_HUMAN]	3.68	1	1	1	163	18.7	7.90
C9JGZ0	Uncharacterized protein OS=Homo sapiens GN=BRIP1 PE=4 SV=1 - [C9JGZ0_HUMAN]	0.80	2	1	1	994	112.4	8.21
B4DW40	cDNA FLJ53498, highly similar to Homo sapiens G protein-coupled receptor 114 (GPR114), mRNA OS=Homo sapiens PE=2 SV=1 - [B4DW40_HUMAN]	3.04	4	1	1	296	32.7	8.65
Q59GI5	Dynamin 1 isoform 2 variant (Fragment) OS=Homo sapiens PE=2 SV=1 - [Q59GI5_HUMAN]	3.00	5	1	1	600	68.8	7.68
B4DSG5	cDNA FLJ56149, highly similar to Tax1-binding protein 1 OS=Homo sapiens PE=2 SV=1 - [B4DSG5_HUMAN]	0.94	3	1	1	747	86.1	5.39
Q15057	Arf-GAP with coiled-coil, ANK repeat and PH domain-containing protein 2 OS=Homo sapiens GN=ACAP2 PE=1 SV=3 - [ACAP2_HUMAN]	3.60	1	1	1	778	88.0	6.80
Q86TB3	Alpha-protein kinase 2 OS=Homo sapiens GN=ALPK2 PE=1 SV=3 - [ALPK2_HUMAN]	1.34	1	1	1	2,170	236.9	5.24
Q9BZ19	Ankyrin repeat domain-containing protein 60 OS=Homo sapiens GN=ANKRD60 PE=2 SV=3 - [ANR60_HUMAN]	4.64	1	1	1	345	37.6	8.95
Q9UBL0	cAMP-regulated phosphoprotein 21 OS=Homo sapiens GN=ARPP21 PE=1 SV=2 - [ARP21_HUMAN]	2.09	1	1	1	812	89.1	6.95
P48047	ATP synthase subunit O, mitochondrial OS=Homo sapiens GN=ATP5O PE=1 SV=1 - [ATPO_HUMAN]	5.16	2	1	1	213	23.3	9.96
Q494V2	Coiled-coil domain-containing protein 37 OS=Homo sapiens GN=CCDC37 PE=1 SV=1 - [CCD37_HUMAN]	1.96	1	1	1	611	71.1	7.11
Q7Z4R8	UPF0669 protein C6orf120 OS=Homo sapiens GN=C6orf120 PE=1 SV=1 - [CF120_HUMAN]	3.14	2	1	1	191	20.8	4.84
C9J069	Uncharacterized protein C9orf172 OS=Homo sapiens GN=C9orf172 PE=3 SV=1 - [CI172_HUMAN]	0.61	1	1	1	976	106.6	9.03
Q9BXX0	EMILIN-2 OS=Homo sapiens GN=EMILIN2 PE=1 SV=3 - [EMIL2_HUMAN]	0.66	1	1	1	1,053	115.6	6.46
Q49AJ0	Protein FAM135B OS=Homo sapiens GN=FAM135B PE=2 SV=2 - [F135B_HUMAN]	0.50	1	1	1	1,406	155.7	5.86
Q9NVF7	F-box only protein 28 OS=Homo sapiens GN=FBXO28 PE=1 SV=1 - [FBX28_HUMAN]	1.63	3	1	1	368	41.1	9.55
Q15485	Ficolin-2 OS=Homo sapiens GN=FCN2 PE=1 SV=2 - [FCN2_HUMAN]	4.47	2	1	1	313	34.0	6.77
Q2WGJ9	Fer-1-like protein 6 OS=Homo sapiens GN=FER1L6 PE=2 SV=2 - [FR1L6_HUMAN]	0.59	1	1	1	1,857	209.2	6.38
Q9UF56	F-box/LRR-repeat protein 17 OS=Homo sapiens GN=FBXL17 PE=2 SV=3 - [FXL17_HUMAN]	1.43	1	1	1	701	75.6	8.07
Q7Z3F1	Integral membrane protein GPR155 OS=Homo sapiens GN=GPR155 PE=1 SV=2 - [GP155_HUMAN]	3.45	1	1	1	870	96.9	6.84
O60741	Potassium/sodium hyperpolarization-activated cyclic nucleotide-gated channel 1 OS=Homo sapiens GN=HCN1 PE=2 SV=3 - [HCN1_HUMAN]	1.80	2	1	1	890	98.7	8.40
P05204	Non-histone chromosomal protein HMG-17 OS=Homo sapiens GN=HMGN2 PE=1 SV=3 - [HMGN2_HUMAN]	8.89	1	1	1	90	9.4	9.99
Q96N76	Urocanate hydratase OS=Homo sapiens GN=UROC1 PE=1 SV=1 - [HUTU_HUMAN]	1.48	3	1	1	676	74.8	6.79
P01771	Ig heavy chain V-III region HIL OS=Homo sapiens PE=1 SV=1 - [HV310_HUMAN]	9.09	1	1	1	121	13.6	9.36
Q8N201	Integrator complex subunit 1 OS=Homo sapiens GN=INTS1 PE=1 SV=2 - [INT1_HUMAN]	0.41	3	1	1	2,190	244.1	6.13
Q8NFU5	Inositol polyphosphate multikinase OS=Homo sapiens GN=IPMK PE=1 SV=1 - [IPMK_HUMAN]	5.29	1	1	1	416	47.2	7.64
Q9NYS0	NF-kappa-B inhibitor-interacting Ras-like protein 1 OS=Homo sapiens GN=NKIRAS1 PE=1 SV=1 - [KBRS1_HUMAN]	4.69	1	1	1	192	21.6	6.35
Q96KK3	Potassium voltage-gated channel subfamily S member 1 OS=Homo sapiens GN=KCNS1 PE=2 SV=2 - [KCNS1_HUMAN]	2.85	1	1	1	526	58.3	7.15
Q9BYZ2	L-lactate dehydrogenase A-like 6B OS=Homo sapiens GN=LDHAL6B PE=1 SV=3 - [LDH6B_HUMAN]	7.09	1	1	1	381	41.9	8.65
Q96KR4	Leishmanolysin-like peptidase OS=Homo sapiens GN=LMLN PE=2 SV=2 - [LMLN_HUMAN]	1.22	3	1	1	655	73.5	6.93
P01708	Ig lambda chain V-II region BUR OS=Homo sapiens PE=1 SV=1 - [LV205_HUMAN]	10.09	1	1	1	109	11.5	7.87
Q05BQ5	MBT domain-containing protein 1 OS=Homo sapiens GN=MBTD1 PE=1 SV=2 - [MBTD1_HUMAN]	0.96	2	1	1	628	70.5	7.77
Q8NGZ3	Olfactory receptor 13G1 OS=Homo sapiens GN=OR13G1 PE=2 SV=1 - [O13G1_HUMAN]	10.10	1	1	1	307	34.6	8.22
Q8NGL2	Olfactory receptor 5L1 OS=Homo sapiens GN=OR5L1 PE=2 SV=1 - [OR5L1_HUMAN]	5.47	1	1	1	311	34.5	7.96
Q8NGG0	Olfactory receptor 8J3 OS=Homo sapiens GN=OR8J3 PE=2 SV=1 - [OR8J3_HUMAN]	2.54	1	1	1	315	35.5	8.29
Q8NH50	Olfactory receptor 8K5 OS=Homo sapiens GN=OR8K5 PE=2 SV=1 - [OR8K5_HUMAN]	5.54	1	1	1	307	35.2	6.96
Q9Y2H5	Pleckstrin homology domain-containing family A member 6 OS=Homo sapiens GN=PLEKHA6 PE=1 SV=4 - [PKHA6_HUMAN]	0.86	2	1	1	1,048	117.1	9.10
Q69YJ1	Putative pleckstrin homology domain-containing family M member 1P OS=Homo sapiens GN=PLEKHM1P PE=5 SV=1 - [PKHMP_HUMAN]	2.31	1	1	1	520	58.9	6.33
Q9UQ72	Pregnancy-specific beta-1-glycoprotein 11 OS=Homo sapiens GN=PSG11 PE=2 SV=3 - [PSG11_HUMAN]	9.85	1	1	1	335	37.1	7.14
Q63HN8	RING finger protein 213 OS=Homo sapiens GN=RNF213 PE=1 SV=2 - [RN213_HUMAN]	0.82	2	1	1	3,280	373.7	6.92
Q9UJJ7	RNA pseudouridylate synthase domain-containing protein 1 OS=Homo sapiens GN=RPUSD1 PE=2 SV=1 - [RUSD1_HUMAN]	2.24	1	1	1	312	34.7	7.05
O75934	Pre-mRNA-splicing factor SPF27 OS=Homo sapiens GN=BCAS2 PE=1 SV=1 - [SPF27_HUMAN]	2.67	2	1	1	225	26.1	5.66
Q7L7X3	Serine/threonine-protein kinase TAO1 OS=Homo sapiens GN=TAOK1 PE=1 SV=1 - [TAOK1_HUMAN]	1.50	1	1	1	1,001	116.0	7.65
Q13472	DNA topoisomerase 3-alpha OS=Homo sapiens GN=TOP3A PE=1 SV=1 - [TOP3A_HUMAN]	0.70	1	1	1	1,001	112.3	8.34
Q07283	Trichohyalin OS=Homo sapiens GN=TCHH PE=1 SV=2 - [TRHY_HUMAN]	1.75	1	1	1	1,943	253.8	5.78
O60858	E3 ubiquitin-protein ligase TRIM13 OS=Homo sapiens GN=TRIM13 PE=1 SV=2 - [TRI13_HUMAN]	2.21	1	1	1	407	47.0	6.01
O00507	Probable ubiquitin carboxyl-terminal hydrolase FAF-Y OS=Homo sapiens GN=USP9Y PE=1 SV=2 - [USP9Y_HUMAN]	0.23	1	1	1	2,555	290.9	5.86
P47989	Xanthine dehydrogenase/oxidase OS=Homo sapiens GN=XDH PE=1 SV=4 - [XDH_HUMAN]	1.13	1	1	1	1,333	146.3	7.66
P15822	Zinc finger protein 40 OS=Homo sapiens GN=HIVEP1 PE=1 SV=3 - [ZEP1_HUMAN]	0.81	1	1	1	2,718	296.7	7.84
Q7Z3T8	Zinc finger FYVE domain-containing protein 16 OS=Homo sapiens GN=ZFYVE16 PE=1 SV=3 - [ZFY16_HUMAN]	1.23	1	1	1	1,539	168.8	4.82
Q8N9Z0	Zinc finger protein 610 OS=Homo sapiens GN=ZNF610 PE=2 SV=2 - [ZN610_HUMAN]	3.46	1	1	1	462	53.5	9.20
P51504	Zinc finger protein 80 OS=Homo sapiens GN=ZNF80 PE=2 SV=2 - [ZNF80_HUMAN]	5.13	1	1	1	273	31.2	8.73
B8ZZ67	SMT3 suppressor of mif two 3 homolog 1 (Yeast), isoform CRA_b OS=Homo sapiens GN=SUMO1 PE=4 SV=1 - [B8ZZ67_HUMAN]	24.19	4	1	1	62	7.2	5.73
A6NCP9	Retinol binding protein 4, plasma, isoform CRA_b OS=Homo sapiens GN=RBP4 PE=3 SV=2 - [A6NCP9_HUMAN]	5.03	3	1	1	199	23.0	6.09
B4DE08	Mediator complex subunit 20mediator complex subunit 20mediator complex subunit 20Trf (TATA binding protein-related factor)-proximal homolog (Drosophila) OS=Homo sapiens GN=TRFP PE=2 SV=1 - [B4DE08_HUMAN]	29.27	4	1	1	123	13.5	7.17
Q5T6V7	Chromosome 9 open reading frame 64 OS=Homo sapiens GN=C9orf64 PE=2 SV=1 - [Q5T6V7_HUMAN]	3.00	2	1	1	200	23.3	6.30
B3KW33	Oxysterol-binding protein OS=Homo sapiens GN=OSBPL9 PE=2 SV=1 - [B3KW33_HUMAN]	4.61	8	1	1	521	58.5	6.39
Q14CE9	SALL1 protein OS=Homo sapiens GN=SALL1 PE=2 SV=1 - [Q14CE9_HUMAN]	10.88	4	1	1	147	15.6	8.59
B3KUT5	HCG1645996, isoform CRA_b OS=Homo sapiens GN=hCG_1645996 PE=2 SV=1 - [B3KUT5_HUMAN]	4.97	1	1	1	181	20.4	10.36
D3DW55	Coiled-coil domain containing 33, isoform CRA_c OS=Homo sapiens GN=CCDC33 PE=4 SV=1 - [D3DW55_HUMAN]	4.39	4	1	1	205	22.7	8.29
Q2M272	Protocadherin gamma subfamily C, 5, isoform 2 OS=Homo sapiens GN=PCDHGC5 PE=2 SV=1 - [Q2M272_HUMAN]	1.82	2	1	1	878	95.0	5.15
B4DIG5	BRF1 homolog, subunit of RNA polymerase III transcription initiation factor IIIB (S. cerevisiae), isoform CRA_e OS=Homo sapiens GN=BRF1 PE=2 SV=1 - [B4DIG5_HUMAN]	1.25	3	1	1	562	61.8	5.27
D3DP46	Signal peptidase complex subunit 3 homolog (S. cerevisiae), isoform CRA_a OS=Homo sapiens GN=SPCS3 PE=4 SV=1 - [D3DP46_HUMAN]	3.89	2	1	1	180	20.3	8.97
E9PKX1	Uncharacterized protein OS=Homo sapiens GN=ZCCHC11 PE=4 SV=1 - [E9PKX1_HUMAN]	0.77	2	1	1	906	103.1	6.79
E9PMP4	Uncharacterized protein OS=Homo sapiens GN=CCDC67 PE=4 SV=1 - [E9PMP4_HUMAN]	31.13	6	1	1	106	12.3	5.26
C1PHC3	CD44 molecule (Fragment) OS=Homo sapiens GN=CD44 PE=4 SV=1 - [C1PHC3_HUMAN]	17.78	14	1	1	45	5.0	8.57
A2A2F9	Chromosome 20 open reading frame 3 (Fragment) OS=Homo sapiens GN=C20orf3 PE=4 SV=1 - [A2A2F9_HUMAN]	3.68	2	1	1	408	45.3	5.66
B3KYB1	cDNA FLJ16760 fis, clone BRACE3050764, highly similar to Cell division protein kinase 10 (EC 2.7.11.22) OS=Homo sapiens PE=2 SV=1 - [B3KYB1_HUMAN]	2.24	3	1	1	313	35.6	8.68
B4DYR1	cDNA FLJ51576, highly similar to Jagged-1 OS=Homo sapiens PE=2 SV=1 - [B4DYR1_HUMAN]	1.42	4	1	1	1,059	116.5	5.73
B3KN02	cDNA FLJ13106 fis, clone NT2RP3002455, highly similar to DnaJ homolog subfamily C member 13 OS=Homo sapiens PE=2 SV=1 - [B3KN02_HUMAN]	1.80	3	1	1	890	99.2	6.01
D2Y6Y4	ATP synthase subunit 6 (Fragment) OS=Homo sapiens GN=ATP6 PE=4 SV=1 - [D2Y6Y4_HUMAN]	50.00	1	1	1	44	4.7	11.17
A8K8N3	cDNA FLJ78740, highly similar to Homo sapiens sperm associated antigen 5 (SPAG5), mRNA OS=Homo sapiens PE=2 SV=1 - [A8K8N3_HUMAN]	1.42	2	1	1	1,193	134.3	5.00
Q9H2G3	CTCL tumor antigen se20-7 (Fragment) OS=Homo sapiens PE=2 SV=1 - [Q9H2G3_HUMAN]	1.77	1	1	1	623	73.8	5.71
C9J8D8	Uncharacterized protein OS=Homo sapiens GN=SPEG PE=4 SV=1 - [C9J8D8_HUMAN]	2.34	4	1	1	299	32.4	10.48
Q5T2S9	Novel protein (Fragment) OS=Homo sapiens GN=RP11-691I13.1 PE=2 SV=1 - [Q5T2S9_HUMAN]	2.57	2	1	1	389	44.3	9.03
B4E0Y2	cDNA FLJ58219, highly similar to TBC1 domain family member 1 OS=Homo sapiens PE=2 SV=1 - [B4E0Y2_HUMAN]	1.73	5	1	1	463	51.8	6.68
C9IYV3	Uncharacterized protein OS=Homo sapiens GN=GUCA1B PE=4 SV=1 - [C9IYV3_HUMAN]	5.23	1	1	1	172	19.9	4.63
C9JY21	Uncharacterized protein OS=Homo sapiens GN=CPA1 PE=4 SV=1 - [C9JY21_HUMAN]	11.27	2	1	1	142	16.4	4.94
C9JA91	Uncharacterized protein OS=Homo sapiens GN=TACC3 PE=4 SV=1 - [C9JA91_HUMAN]	3.03	6	1	1	231	24.8	4.92
Q9BZK2	CTLA-4 V domain region (Fragment) OS=Homo sapiens PE=2 SV=1 - [Q9BZK2_HUMAN]	39.13	5	1	1	115	12.3	4.94
B7Z8U4	cDNA FLJ52387, highly similar to Kelch-like protein 3 OS=Homo sapiens PE=2 SV=1 - [B7Z8U4_HUMAN]	6.00	12	1	1	200	22.7	7.21
B7Z879	cDNA FLJ54956, highly similar to Vacuolar protein sorting 11 OS=Homo sapiens PE=2 SV=1 - [B7Z879_HUMAN]	2.36	4	1	1	931	107.3	7.52
E9PCP1	Uncharacterized protein OS=Homo sapiens GN=SLC4A3 PE=4 SV=1 - [E9PCP1_HUMAN]	7.05	3	1	1	227	25.6	6.74
Q59EE7	Pro-alpha-1 type V collagen variant (Fragment) OS=Homo sapiens PE=2 SV=1 - [Q59EE7_HUMAN]	1.40	3	1	1	1,792	178.4	5.00
B4E2H7	cDNA FLJ59819, highly similar to Homo sapiens nephronectin (NPNT), mRNA OS=Homo sapiens PE=2 SV=1 - [B4E2H7_HUMAN]	2.43	10	1	1	536	58.6	8.57
C9J7C5	Uncharacterized protein OS=Homo sapiens GN=LIPT1 PE=4 SV=1 - [C9J7C5_HUMAN]	20.51	5	1	1	39	4.3	9.20
E9PMF7	Uncharacterized protein OS=Homo sapiens GN=PTPRC PE=4 SV=1 - [E9PMF7_HUMAN]	10.96	2	1	1	146	15.9	5.00
Q6P6D5	MYT1 protein OS=Homo sapiens GN=MYT1 PE=2 SV=1 - [Q6P6D5_HUMAN]	1.52	3	1	1	591	64.6	6.84
Q9HAL2	cDNA FLJ11413 fis, clone HEMBA1000908 OS=Homo sapiens PE=2 SV=1 - [Q9HAL2_HUMAN]	10.56	1	1	1	161	17.5	9.26
B2RUU3	Dedicator of cytokinesis 1 OS=Homo sapiens GN=DOCK1 PE=2 SV=1 - [B2RUU3_HUMAN]	0.48	3	1	1	1,865	215.2	7.49
B4DG25	cDNA FLJ52157, highly similar to Probable G-protein coupled receptor 12 OS=Homo sapiens PE=2 SV=1 - [B4DG25_HUMAN]	9.71	4	1	1	175	19.6	8.56
C9J154	Uncharacterized protein OS=Homo sapiens GN=VEZT PE=4 SV=1 - [C9J154_HUMAN]	0.77	2	1	1	779	88.6	5.29
Q53SG8	Putative uncharacterized protein FLJ22527 (Fragment) OS=Homo sapiens GN=FLJ22527 PE=2 SV=1 - [Q53SG8_HUMAN]	9.05	9	1	1	199	22.8	9.92
B4DN09	cDNA FLJ55858, highly similar to Homo sapiens formin 2 (FMN2), mRNA OS=Homo sapiens PE=2 SV=1 - [B4DN09_HUMAN]	2.56	1	1	1	351	40.6	7.12
B4E3U4	cDNA FLJ59107, moderately similar to Heterogeneous nuclear ribonucleoprotein G OS=Homo sapiens PE=2 SV=1 - [B4E3U4_HUMAN]	9.18	1	1	1	196	22.2	5.26
E7EMA7	Uncharacterized protein OS=Homo sapiens GN=PDPR PE=3 SV=1 - [E7EMA7_HUMAN]	2.41	3	1	1	582	66.3	5.66
C9JJ39	Uncharacterized protein OS=Homo sapiens GN=STARD3NL PE=4 SV=1 - [C9JJ39_HUMAN]	13.37	5	1	1	172	19.8	6.52
B4DIK9	cDNA FLJ55877 OS=Homo sapiens PE=2 SV=1 - [B4DIK9_HUMAN]	4.87	1	1	1	226	23.9	9.76
Q5NV92	V5-6 protein (Fragment) OS=Homo sapiens GN=IGLV4-69 PE=4 SV=1 - [Q5NV92_HUMAN]	10.10	2	1	1	99	10.6	6.27
B4DUC2	cDNA FLJ54483, highly similar to Serine/threonine-protein kinase tousled-like 2 (EC 2.7.11.1) OS=Homo sapiens PE=2 SV=1 - [B4DUC2_HUMAN]	2.92	3	1	1	718	82.4	8.18
B4DGB9	cDNA FLJ57363 OS=Homo sapiens PE=2 SV=1 - [B4DGB9_HUMAN]	11.60	1	1	1	181	20.3	9.04
B3KMU4	cDNA FLJ12640 fis, clone NT2RM4001940, highly similar to Timeless homolog OS=Homo sapiens PE=2 SV=1 - [B3KMU4_HUMAN]	2.29	2	1	1	481	54.5	5.08
A6NLR2	Uncharacterized protein OS=Homo sapiens GN=MAN1A2 PE=4 SV=2 - [A6NLR2_HUMAN]	2.78	2	1	1	611	69.6	8.12
Q59FA5	Transgelin variant (Fragment) OS=Homo sapiens PE=2 SV=1 - [Q59FA5_HUMAN]	16.07	2	1	1	112	12.3	7.40
Q08E77	UTP14, U3 small nucleolar ribonucleoprotein, homolog C (Yeast) OS=Homo sapiens GN=UTP14C PE=2 SV=1 - [Q08E77_HUMAN]	2.22	2	1	1	766	87.1	7.30
Q05BJ6	CEP290 protein OS=Homo sapiens GN=CEP290 PE=2 SV=1 - [Q05BJ6_HUMAN]	1.99	2	1	1	805	94.2	5.16
A6NE97	Uncharacterized protein OS=Homo sapiens GN=CYP2A7 PE=3 SV=2 - [A6NE97_HUMAN]	1.58	5	1	1	443	50.6	8.65
Q7M4S4	Granulocyte inhibitory protein OS=Homo sapiens PE=1 SV=1 - [Q7M4S4_HUMAN]	90.00	1	1	1	20	2.0	4.50
Q9H9Z0	cDNA FLJ12463 fis, clone NT2RM1000772, weakly similar to VEGETATIBLE INCOMPATIBILITY PROTEIN HET-E-1 OS=Homo sapiens PE=2 SV=1 - [Q9H9Z0_HUMAN]	0.93	10	1	1	864	98.3	7.27
A1A4T9	ZNF662 protein OS=Homo sapiens GN=ZNF662 PE=2 SV=1 - [A1A4T9_HUMAN]	6.14	3	1	1	391	44.8	7.85
Q5TCY7	Vasoactive intestinal peptide (Fragment) OS=Homo sapiens GN=VIP PE=2 SV=1 - [Q5TCY7_HUMAN]	18.64	3	1	1	118	13.4	6.58
A0PJC9	TPR protein (Fragment) OS=Homo sapiens GN=TPR PE=2 SV=1 - [A0PJC9_HUMAN]	2.76	6	1	1	471	53.8	5.60
A8K8U8	cDNA FLJ75856, highly similar to Homo sapiens A kinase (PRKA) anchor protein 4 (AKAP4), transcript variant 1, mRNA OS=Homo sapiens PE=2 SV=1 - [A8K8U8_HUMAN]	2.22	2	1	1	854	94.4	7.06
A6NF12	Tyrosine-protein kinase receptor OS=Homo sapiens GN=NTRK1 PE=3 SV=1 - [A6NF12_HUMAN]	1.45	3	1	1	760	83.9	6.73
D6RD92	Uncharacterized protein OS=Homo sapiens GN=TSC22D3 PE=4 SV=1 - [D6RD92_HUMAN]	32.00	9	1	1	25	3.1	5.10
B4DR99	cDNA FLJ59140 OS=Homo sapiens PE=2 SV=1 - [B4DR99_HUMAN]	6.50	3	1	1	200	23.3	6.39
E9PF99	Uncharacterized protein OS=Homo sapiens GN=CEP192 PE=4 SV=1 - [E9PF99_HUMAN]	0.32	1	1	1	2,537	278.9	5.48
B4DQI3	cDNA FLJ60796, highly similar to Alpha-1,2-mannosyltransferase ALG9 (EC 2.4.1.-) OS=Homo sapiens PE=2 SV=1 - [B4DQI3_HUMAN]	9.09	4	1	1	440	50.2	7.75
B4DPH8	cDNA FLJ55125 OS=Homo sapiens PE=2 SV=1 - [B4DPH8_HUMAN]	2.62	2	1	1	229	25.6	5.30
B4DDF8	cDNA FLJ51786, highly similar to Retinal dehydrogenase 1 (EC 1.2.1.36) OS=Homo sapiens PE=2 SV=1 - [B4DDF8_HUMAN]	4.50	2	1	1	422	46.0	7.59
Q8NCJ2	cDNA FLJ90221 fis, clone NT2RM1000462, highly similar to Cat eye syndrome critical region protein 1precursor OS=Homo sapiens PE=2 SV=1 - [Q8NCJ2_HUMAN]	2.96	5	1	1	270	30.6	6.76
Q8TD12	Decay-accelerating factor 3 (Fragment) OS=Homo sapiens PE=2 SV=1 - [Q8TD12_HUMAN]	3.40	13	1	1	265	29.4	8.44
E5RK24	Uncharacterized protein OS=Homo sapiens GN=GTF2E2 PE=4 SV=1 - [E5RK24_HUMAN]	16.44	4	1	1	73	8.0	9.82
B3KU41	cDNA FLJ39170 fis, clone OCBBF2003028, highly similar to Rattus norvegicus basic leucine zipper and W2 domains 1 (Bzw1), mRNA OS=Homo sapiens PE=2 SV=1 - [B3KU41_HUMAN]	4.27	8	1	1	211	24.5	8.95
Q59FN4	LATS homolog 1 variant (Fragment) OS=Homo sapiens PE=2 SV=1 - [Q59FN4_HUMAN]	4.46	2	1	1	830	92.3	9.45
B4DPG5	cDNA FLJ55902 OS=Homo sapiens PE=2 SV=1 - [B4DPG5_HUMAN]	1.59	1	1	1	566	62.4	5.92
Q6ZW66	cDNA FLJ41543 fis, clone CERVX2002006 OS=Homo sapiens PE=2 SV=1 - [Q6ZW66_HUMAN]	5.51	1	1	1	127	14.8	10.17
E9PLX2	Uncharacterized protein OS=Homo sapiens GN=RNF141 PE=4 SV=1 - [E9PLX2_HUMAN]	20.56	3	1	1	180	19.8	5.38
E9PMB2	Uncharacterized protein OS=Homo sapiens GN=GPA33 PE=4 SV=1 - [E9PMB2_HUMAN]	16.05	1	1	1	81	9.1	10.02
Q7Z2U6	ZNF701 protein (Fragment) OS=Homo sapiens GN=ZNF701 PE=2 SV=1 - [Q7Z2U6_HUMAN]	4.64	4	1	1	151	17.5	8.79
B3KUI5	cDNA FLJ39968 fis, clone SPLEN2027780, highly similar to Hyaluronidase-1 (EC 3.2.1.35) OS=Homo sapiens PE=2 SV=1 - [B3KUI5_HUMAN]	3.68	2	1	1	435	48.3	6.96
Q6IQ32	ADNP homeobox protein 2 OS=Homo sapiens GN=ADNP2 PE=2 SV=1 - [ADNP2_HUMAN]	0.88	1	1	1	1,131	122.8	9.16
Q9NZD4	Alpha-hemoglobin-stabilizing protein OS=Homo sapiens GN=AHSP PE=1 SV=1 - [AHSP_HUMAN]	9.80	1	1	1	102	11.8	5.00
Q92843	Bcl-2-like protein 2 OS=Homo sapiens GN=BCL2L2 PE=1 SV=2 - [B2CL2_HUMAN]	4.15	1	1	1	193	20.7	5.34
Q13867	Bleomycin hydrolase OS=Homo sapiens GN=BLMH PE=1 SV=1 - [BLMH_HUMAN]	3.30	1	1	1	455	52.5	6.27
Q96EH3	Uncharacterized protein C7orf30 OS=Homo sapiens GN=C7orf30 PE=2 SV=1 - [CG030_HUMAN]	2.99	1	1	1	234	26.2	5.49
O75122	CLIP-associating protein 2 OS=Homo sapiens GN=CLASP2 PE=1 SV=2 - [CLAP2_HUMAN]	1.93	2	1	1	1,294	141.0	8.47
A6NMZ7	Collagen alpha-6(VI) chain OS=Homo sapiens GN=COL6A6 PE=1 SV=2 - [CO6A6_HUMAN]	0.40	1	1	1	2,263	247.0	6.89
Q6ZTR5	Uncharacterized protein CXorf22 OS=Homo sapiens GN=CXorf22 PE=2 SV=3 - [CX022_HUMAN]	1.84	1	1	1	976	110.3	8.16
Q86TM3	Probable ATP-dependent RNA helicase DDX53 OS=Homo sapiens GN=DDX53 PE=1 SV=3 - [DDX53_HUMAN]	1.11	1	1	1	631	71.1	9.07
Q8TCP9	Protein FAM200A OS=Homo sapiens GN=FAM200A PE=2 SV=1 - [F200A_HUMAN]	2.09	1	1	1	573	66.2	6.21
Q9BQS8	FYVE and coiled-coil domain-containing protein 1 OS=Homo sapiens GN=FYCO1 PE=1 SV=3 - [FYCO1_HUMAN]	0.88	2	1	1	1,478	166.9	4.92
Q4ZG55	Protein GREB1 OS=Homo sapiens GN=GREB1 PE=2 SV=1 - [GREB1_HUMAN]	0.72	1	1	1	1,949	216.3	6.95
P52333	Tyrosine-protein kinase JAK3 OS=Homo sapiens GN=JAK3 PE=1 SV=2 - [JAK3_HUMAN]	1.42	1	1	1	1,124	125.0	7.18
Q9UPV7	Protein KIAA1045 OS=Homo sapiens GN=KIAA1045 PE=1 SV=2 - [K1045_HUMAN]	2.50	1	1	1	400	45.2	5.67
P09848	Lactase-phlorizin hydrolase OS=Homo sapiens GN=LCT PE=1 SV=3 - [LPH_HUMAN]	0.42	1	1	1	1,927	218.4	6.34
Q6NSJ5	Leucine-rich repeat-containing protein 8E OS=Homo sapiens GN=LRRC8E PE=2 SV=2 - [LRC8E_HUMAN]	1.38	1	1	1	796	90.2	6.96
O94898	Leucine-rich repeats and immunoglobulin-like domains protein 2 OS=Homo sapiens GN=LRIG2 PE=1 SV=3 - [LRIG2_HUMAN]	1.50	1	1	1	1,065	118.9	5.55
O75197	Low-density lipoprotein receptor-related protein 5 OS=Homo sapiens GN=LRP5 PE=1 SV=2 - [LRP5_HUMAN]	0.43	1	1	1	1,615	179.0	5.34
A4FU01	Myotubularin-related protein 11 OS=Homo sapiens GN=MTMR11 PE=2 SV=2 - [MTMRB_HUMAN]	1.69	1	1	1	709	79.5	7.03
Q9BZQ8	Protein Niban OS=Homo sapiens GN=FAM129A PE=1 SV=1 - [NIBAN_HUMAN]	1.08	1	1	1	928	103.1	4.78
O95989	Diphosphoinositol polyphosphate phosphohydrolase 1 OS=Homo sapiens GN=NUDT3 PE=1 SV=1 - [NUDT3_HUMAN]	5.81	1	1	1	172	19.5	6.34
Q9H792	Pseudopodium-enriched atypical kinase 1 OS=Homo sapiens GN=PEAK1 PE=1 SV=4 - [PEAK1_HUMAN]	0.92	1	1	1	1,746	193.0	6.89
Q7Z443	Polycystic kidney disease protein 1-like 3 OS=Homo sapiens GN=PKD1L3 PE=1 SV=1 - [PK1L3_HUMAN]	0.81	1	1	1	1,732	195.8	8.48
O75051	Plexin-A2 OS=Homo sapiens GN=PLXNA2 PE=1 SV=4 - [PLXA2_HUMAN]	0.79	1	1	1	1,894	211.0	6.48
Q9NQV5	PR domain-containing protein 11 OS=Homo sapiens GN=PRDM11 PE=1 SV=2 - [PRD11_HUMAN]	1.17	1	1	1	511	57.8	6.18
Q2KHN1	RING finger protein 151 OS=Homo sapiens GN=RNF151 PE=2 SV=1 - [RN151_HUMAN]	6.53	1	1	1	245	27.4	8.69
Q96C34	RUN domain-containing protein 1 OS=Homo sapiens GN=RUNDC1 PE=1 SV=3 - [RUND1_HUMAN]	2.94	1	1	1	613	67.6	6.19
Q92529	SHC-transforming protein 3 OS=Homo sapiens GN=SHC3 PE=1 SV=1 - [SHC3_HUMAN]	2.69	1	1	1	594	64.0	8.32
Q8NB90	Spermatogenesis-associated protein 5 OS=Homo sapiens GN=SPATA5 PE=1 SV=3 - [SPAT5_HUMAN]	1.34	1	1	1	893	97.8	5.66
Q7Z699	Sprouty-related, EVH1 domain-containing protein 1 OS=Homo sapiens GN=SPRED1 PE=1 SV=2 - [SPRE1_HUMAN]	2.03	1	1	1	444	50.4	6.54
Q9NS56	E3 ubiquitin-protein ligase Topors OS=Homo sapiens GN=TOPORS PE=1 SV=1 - [TOPRS_HUMAN]	0.57	1	1	1	1,045	119.1	9.51
Q9UMW8	Ubl carboxyl-terminal hydrolase 18 OS=Homo sapiens GN=USP18 PE=1 SV=1 - [UBP18_HUMAN]	5.38	1	1	1	372	43.0	7.80
A8MWX3	Putative WAS protein family homolog 4 OS=Homo sapiens GN=WASH4P PE=5 SV=1 - [WASH4_HUMAN]	2.10	1	1	1	477	51.6	6.67
A6NF79	Zinc finger protein 734 OS=Homo sapiens GN=ZNF734 PE=3 SV=3 - [ZN734_HUMAN]	3.40	1	1	1	559	64.8	9.29
E9PDB0	Uncharacterized protein OS=Homo sapiens GN=WDR49 PE=4 SV=1 - [E9PDB0_HUMAN]	1.54	2	1	1	518	58.8	6.58
Q5VVX1	Parkinson disease (Autosomal recessive, juvenile) 2, parkin OS=Homo sapiens GN=PARK2 PE=2 SV=1 - [Q5VVX1_HUMAN]	8.39	7	1	1	274	30.6	6.74
B8ZZZ7	DNA polymerase-transactivated protein 6, isoform CRA_b OS=Homo sapiens GN=SPATS2L PE=4 SV=1 - [B8ZZZ7_HUMAN]	4.02	4	1	1	498	54.9	9.73
D6RB33	DCN1, defective in cullin neddylation 1, domain containing 4 (S. cerevisiae), isoform CRA_e OS=Homo sapiens GN=DCUN1D4 PE=4 SV=1 - [D6RB33_HUMAN]	12.59	5	1	1	143	16.3	6.28
E1B6X3	Chromosome 17 open reading frame 62, isoform CRA_g OS=Homo sapiens GN=C17orf62 PE=4 SV=1 - [E1B6X3_HUMAN]	10.40	2	1	1	173	19.4	6.80
C0H5X6	PEX7 protein OS=Homo sapiens GN=PEX7 PE=2 SV=1 - [C0H5X6_HUMAN]	5.71	2	1	1	280	30.9	6.33
C9JAJ5	Putative uncharacterized protein LOC349136 OS=Homo sapiens GN=WDR86 PE=4 SV=1 - [C9JAJ5_HUMAN]	6.14	1	1	1	277	29.0	8.66
B4DR51	HCG2036867 OS=Homo sapiens GN=hCG_2036867 PE=2 SV=1 - [B4DR51_HUMAN]	1.53	1	1	1	590	68.5	9.01
B2RWN4	TUBGCP6 protein OS=Homo sapiens GN=TUBGCP6 PE=2 SV=1 - [B2RWN4_HUMAN]	0.99	2	1	1	1,811	199.4	6.25
B2RAN2	cDNA, FLJ95014, highly similar to Homo sapiens vanin 1 (VNN1), mRNA OS=Homo sapiens PE=2 SV=1 - [B2RAN2_HUMAN]	1.75	2	1	1	513	57.0	5.57
Q59EX8	Low density lipoprotein receptor-related protein 6 variant (Fragment) OS=Homo sapiens PE=2 SV=1 - [Q59EX8_HUMAN]	0.74	3	1	1	1,477	165.3	5.38
Q05DA9	SFRS17A protein (Fragment) OS=Homo sapiens GN=SFRS17A PE=2 SV=1 - [Q05DA9_HUMAN]	2.05	2	1	1	292	33.5	8.34
A2IDA3	N-methylpurine-DNA glycosylase (Fragment) OS=Homo sapiens GN=MPG PE=3 SV=1 - [A2IDA3_HUMAN]	3.19	3	1	1	251	27.3	9.03
B4DP13	cDNA FLJ56607 OS=Homo sapiens PE=2 SV=1 - [B4DP13_HUMAN]	1.62	2	1	1	431	49.5	6.74
B2R8K8	cDNA, FLJ93949, highly similar to Homo sapiens NIMA (never in mitosis gene a)-related kinase 7 (NEK7), mRNA OS=Homo sapiens PE=2 SV=1 - [B2R8K8_HUMAN]	1.99	2	1	1	302	34.5	8.25
B2RA57	cDNA, FLJ94708 OS=Homo sapiens PE=2 SV=1 - [B2RA57_HUMAN]	3.23	4	1	1	279	31.4	8.16
B4DLD1	cDNA FLJ57342, highly similar to Laminin gamma-2 chain OS=Homo sapiens PE=2 SV=1 - [B4DLD1_HUMAN]	1.98	1	1	1	1,010	110.6	6.99
Q05200	C-Ha-ras protein (Fragment) OS=Homo sapiens GN=c-Ha-ras PE=4 SV=1 - [Q05200_HUMAN]	21.92	1	1	1	73	7.5	11.93
A0AUJ7	OR5F1 protein (Fragment) OS=Homo sapiens GN=OR5F1 PE=2 SV=1 - [A0AUJ7_HUMAN]	2.88	2	1	1	313	35.0	8.62
B3KMU0	cDNA FLJ12607 fis, clone NT2RM4001489, highly similar to SAPS domain family member 2 OS=Homo sapiens PE=2 SV=1 - [B3KMU0_HUMAN]	3.86	3	1	1	492	51.5	4.74
B4DT40	cDNA FLJ57586 OS=Homo sapiens PE=2 SV=1 - [B4DT40_HUMAN]	12.02	4	1	1	183	20.7	9.26
D6RJA8	Uncharacterized protein OS=Homo sapiens GN=ACAD9 PE=4 SV=1 - [D6RJA8_HUMAN]	9.68	2	1	1	93	10.6	11.85
E5RFG6	Uncharacterized protein OS=Homo sapiens GN=WDR67 PE=4 SV=1 - [E5RFG6_HUMAN]	5.65	4	1	1	248	28.1	8.70
E9PL46	Uncharacterized protein OS=Homo sapiens GN=FBXO3 PE=4 SV=1 - [E9PL46_HUMAN]	4.65	5	1	1	129	15.2	8.32
E7EQT4	Uncharacterized protein OS=Homo sapiens GN=ACIN1 PE=4 SV=1 - [E7EQT4_HUMAN]	0.73	4	1	1	1,094	122.2	5.62
B4DHX9	cDNA FLJ50769, highly similar to Kallikrein-7 (EC 3.4.21.-) OS=Homo sapiens PE=2 SV=1 - [B4DHX9_HUMAN]	6.43	3	1	1	140	15.2	8.54
Q59H55	Protein tyrosine phosphatase, non-receptor type 13 isoform 2 variant (Fragment) OS=Homo sapiens PE=2 SV=1 - [Q59H55_HUMAN]	0.62	2	1	1	2,434	271.3	6.52
Q53F79	PAP associated domain containing protein 5 variant (Fragment) OS=Homo sapiens PE=2 SV=1 - [Q53F79_HUMAN]	3.22	4	1	1	466	51.8	8.63
B4DGZ0	cDNA FLJ61246, highly similar to Protein MICAL-2 OS=Homo sapiens PE=2 SV=1 - [B4DGZ0_HUMAN]	1.45	2	1	1	1,103	124.1	8.56
B4DF49	cDNA FLJ52535, highly similar to Homo sapiens lysosomal-associated membrane protein 2 (LAMP2), transcript variant LAMP2B, mRNA OS=Homo sapiens PE=2 SV=1 - [B4DF49_HUMAN]	6.79	6	1	1	265	29.2	7.91
E9PB56	Uncharacterized protein OS=Homo sapiens GN=DOK7 PE=4 SV=1 - [E9PB56_HUMAN]	5.88	2	1	1	255	27.5	8.79
B3KY56	cDNA FLJ46898 fis, clone UTERU3022168, highly similar to Protein FAM62A OS=Homo sapiens PE=2 SV=1 - [B3KY56_HUMAN]	2.08	3	1	1	1,058	117.3	5.90
A8K0Z6	cDNA FLJ76212 OS=Homo sapiens PE=2 SV=1 - [A8K0Z6_HUMAN]	2.42	3	1	1	289	32.3	8.41
B3KXZ8	cDNA FLJ46469 fis, clone THYMU3023107, highly similar to Transmembrane channel-like protein 8 OS=Homo sapiens PE=2 SV=1 - [B3KXZ8_HUMAN]	2.06	3	1	1	340	37.7	8.76
B9EK54	RTL1 protein OS=Homo sapiens GN=RTL1 PE=2 SV=1 - [B9EK54_HUMAN]	1.62	3	1	1	1,358	155.0	5.22
Q6FH17	ARF6 protein OS=Homo sapiens GN=ARF6 PE=2 SV=1 - [Q6FH17_HUMAN]	8.00	1	1	1	175	20.1	8.70
B3KTG2	cDNA FLJ38193 fis, clone FCBBF1000280, highly similar to Homo sapiens palladin, cytoskeletal associated protein (PALLD), mRNA OS=Homo sapiens PE=2 SV=1 - [B3KTG2_HUMAN]	1.16	4	1	1	777	85.8	6.89
E9PIP8	Uncharacterized protein OS=Homo sapiens GN=NOS1AP PE=4 SV=1 - [E9PIP8_HUMAN]	23.46	4	1	1	81	8.6	5.71
E9PKW0	Uncharacterized protein OS=Homo sapiens GN=IFT46 PE=4 SV=1 - [E9PKW0_HUMAN]	10.19	6	1	1	108	12.3	4.13
C9J941	Uncharacterized protein OS=Homo sapiens GN=GOLM1 PE=4 SV=1 - [C9J941_HUMAN]	11.25	4	1	1	80	9.0	9.82
Q75MP1	Putative uncharacterized protein PRKAR2B (Fragment) OS=Homo sapiens GN=PRKAR2B PE=2 SV=1 - [Q75MP1_HUMAN]	5.23	4	1	1	306	34.1	4.86
Q5FBW8	Postmeiotic segregation increased 2 nirs variant 5 OS=Homo sapiens GN=PMS2 PE=2 SV=1 - [Q5FBW8_HUMAN]	2.12	3	1	1	756	84.6	8.03
C9JPS5	Uncharacterized protein OS=Homo sapiens GN=SLC39A5 PE=4 SV=1 - [C9JPS5_HUMAN]	3.85	4	1	1	156	16.4	6.42
B4E3C4	cDNA FLJ52848, highly similar to ATP-dependent RNA helicase DDX3X (EC 3.6.1.-) OS=Homo sapiens PE=2 SV=1 - [B4E3C4_HUMAN]	8.12	1	1	1	308	33.8	8.70
E9PKF7	Uncharacterized protein OS=Homo sapiens GN=ANGPTL5 PE=4 SV=1 - [E9PKF7_HUMAN]	4.69	2	1	1	192	21.9	4.98
Q6ZSZ4	cDNA FLJ45103 fis, clone BRAWH3032571, moderately similar to Chromodomain helicase-DNA-binding protein 4 OS=Homo sapiens PE=2 SV=1 - [Q6ZSZ4_HUMAN]	0.65	6	1	1	1,225	141.1	8.10
Q5HY46	Tafazzin (Cardiomyopathy, dilated 3A (X-linked); endocardial fibroelastosis 2; Barth syndrome) (Fragment) OS=Homo sapiens GN=TAZ PE=2 SV=1 - [Q5HY46_HUMAN]	5.29	5	1	1	170	19.1	7.80
A4D137	Similar to Six transmembrane epithelial antigen of prostate OS=Homo sapiens GN=MGC87042 PE=4 SV=1 - [A4D137_HUMAN]	3.56	1	1	1	450	50.3	8.37
C9JEF4	Uncharacterized protein OS=Homo sapiens GN=HDAC6 PE=4 SV=1 - [C9JEF4_HUMAN]	29.41	14	1	1	51	5.6	11.72
B4DUJ3	cDNA FLJ52895, highly similar to Carbonic anhydrase 3 (EC 4.2.1.1) OS=Homo sapiens PE=2 SV=1 - [B4DUJ3_HUMAN]	5.96	4	1	1	235	26.8	8.31
C9JX13	Uncharacterized protein OS=Homo sapiens GN=UBE2Q2 PE=4 SV=1 - [C9JX13_HUMAN]	2.55	2	1	1	314	36.1	4.84
D6RD63	Uncharacterized protein OS=Homo sapiens GN=COPS4 PE=4 SV=1 - [D6RD63_HUMAN]	6.55	7	1	1	275	31.4	6.54
E7EV53	Uncharacterized protein OS=Homo sapiens GN=GABRA6 PE=3 SV=1 - [E7EV53_HUMAN]	3.16	2	1	1	443	49.7	8.41
B5MEF5	Uncharacterized protein OS=Homo sapiens GN=SNED1 PE=4 SV=2 - [B5MEF5_HUMAN]	0.51	2	1	1	1,380	148.6	7.03
B4DYK2	cDNA FLJ56270, highly similar to Homo sapiens pleckstrin homology domain containing, family G (with RhoGef domain) member 2, mRNA OS=Homo sapiens PE=2 SV=1 - [B4DYK2_HUMAN]	0.81	3	1	1	1,237	133.3	5.40
B7Z5Y4	cDNA FLJ55381, highly similar to Choline transporter-like protein 5 OS=Homo sapiens PE=2 SV=1 - [B7Z5Y4_HUMAN]	1.82	3	1	1	713	80.8	8.65
D6RER7	Uncharacterized protein OS=Homo sapiens GN=FYB PE=4 SV=1 - [D6RER7_HUMAN]	8.70	8	1	1	115	12.0	10.14
B2RC50	cDNA, FLJ95853, highly similar to Homo sapiens exosome component Rrp46 (RRP46), mRNA OS=Homo sapiens PE=2 SV=1 - [B2RC50_HUMAN]	6.38	2	1	1	235	25.4	8.18
C9JTR3	Uncharacterized protein OS=Homo sapiens GN=RTTN PE=4 SV=1 - [C9JTR3_HUMAN]	1.07	2	1	1	1,593	178.8	6.37
B4DRP5	cDNA FLJ54718, highly similar to Alpha-actinin-1 OS=Homo sapiens PE=2 SV=1 - [B4DRP5_HUMAN]	2.35	16	1	1	255	28.8	8.38
B3KVK0	cDNA FLJ16660 fis, clone TESTI4042440, moderately similar to Mus musculus axotrophin (Axot) OS=Homo sapiens PE=2 SV=1 - [B3KVK0_HUMAN]	2.38	2	1	1	799	89.8	7.21
B3KNJ2	cDNA FLJ14679 fis, clone NT2RP2004232, highly similar to Serine/threonine-protein kinase D3 (EC 2.7.11.13) OS=Homo sapiens PE=2 SV=1 - [B3KNJ2_HUMAN]	2.22	1	1	1	631	72.1	6.54
A8K0G2	cDNA FLJ78222 OS=Homo sapiens PE=2 SV=1 - [A8K0G2_HUMAN]	3.34	5	1	1	419	46.8	5.35
B9ZVD2	Uncharacterized protein OS=Homo sapiens GN=EYS PE=4 SV=2 - [B9ZVD2_HUMAN]	0.82	2	1	1	3,165	350.6	5.78
C9JIN0	Uncharacterized protein OS=Homo sapiens GN=ZNF555 PE=4 SV=1 - [C9JIN0_HUMAN]	1.47	3	1	1	543	63.0	9.03
E9PHG3	Uncharacterized protein OS=Homo sapiens GN=SMYD1 PE=4 SV=1 - [E9PHG3_HUMAN]	1.47	4	1	1	477	55.0	7.03
A6NHA5	Uncharacterized protein OS=Homo sapiens GN=CLEC4D PE=4 SV=1 - [A6NHA5_HUMAN]	9.32	2	1	1	161	18.2	7.88
E7EWN9	Uncharacterized protein OS=Homo sapiens GN=DSCR3 PE=4 SV=1 - [E7EWN9_HUMAN]	4.35	2	1	1	253	28.3	9.07
Q5JWQ4	Neuropilin 1 (Fragment) OS=Homo sapiens GN=NRP1 PE=2 SV=1 - [Q5JWQ4_HUMAN]	5.51	15	1	1	236	26.7	9.36
Q569I6	ZNF644 protein (Fragment) OS=Homo sapiens GN=ZNF644 PE=2 SV=1 - [Q569I6_HUMAN]	2.64	3	1	1	721	80.6	8.18
B3KSM2	cDNA FLJ36596 fis, clone TRACH2014371, highly similar to Leucine-rich repeat-containing protein 61 OS=Homo sapiens PE=2 SV=1 - [B3KSM2_HUMAN]	8.11	2	1	1	259	28.0	4.77
Q86TX6	Full-length cDNA clone CS0DI006YN07 of Placenta of Homo sapiens (human) OS=Homo sapiens PE=4 SV=1 - [Q86TX6_HUMAN]	25.58	1	1	1	43	5.2	11.05
C9JDX0	Uncharacterized protein OS=Homo sapiens GN=C2orf55 PE=4 SV=1 - [C9JDX0_HUMAN]	24.32	3	1	1	37	4.0	4.44
E9PGR4	Uncharacterized protein OS=Homo sapiens GN=BTN2A1 PE=4 SV=1 - [E9PGR4_HUMAN]	6.06	7	1	1	330	37.2	7.37
Q75MZ8	IRF1 (Fragment) OS=Homo sapiens PE=2 SV=1 - [Q75MZ8_HUMAN]	36.59	2	1	1	41	4.5	4.91
A8K5W7	cDNA FLJ75180, highly similar to Homo sapiens mitochondrial isoleucine tRNA synthetase, mRNA OS=Homo sapiens PE=2 SV=1 - [A8K5W7_HUMAN]	0.96	2	1	1	940	105.9	6.42
B2RE64	cDNA, FLJ93772, highly similar to Homo sapiens vesicle transport through interaction with t-SNAREs homolog 1B (yeast) (VTI1B), mRNA OS=Homo sapiens PE=2 SV=1 - [B2RE64_HUMAN]	5.60	3	1	1	232	26.7	9.04
Q6P6D1	C2CD2 protein OS=Homo sapiens GN=C2CD2 PE=2 SV=1 - [Q6P6D1_HUMAN]	4.64	3	1	1	388	41.7	8.35
A8KAD2	cDNA FLJ77477, highly similar to Homo sapiens zinc finger protein 484 (ZNF484), transcript variant 2, mRNA OS=Homo sapiens PE=2 SV=1 - [A8KAD2_HUMAN]	0.74	4	1	1	816	93.9	8.40
B2R4I8	cDNA, FLJ92106, highly similar to Homo sapiens adaptor-related protein complex 3, sigma 1 subunit(AP3S1), mRNA OS=Homo sapiens PE=2 SV=1 - [B2R4I8_HUMAN]	5.18	2	1	1	193	21.7	5.39
B3KWW9	cDNA FLJ44046 fis, clone TESTI4030505 OS=Homo sapiens PE=2 SV=1 - [B3KWW9_HUMAN]	1.25	2	1	1	1,116	126.8	7.24
B9EGN4	ZNF594 protein OS=Homo sapiens GN=ZNF594 PE=2 SV=1 - [B9EGN4_HUMAN]	1.24	3	1	1	807	93.9	8.72
C9JKR8	Uncharacterized protein OS=Homo sapiens GN=MSL3 PE=4 SV=1 - [C9JKR8_HUMAN]	7.14	6	1	1	224	26.0	7.84
E9PFR5	Uncharacterized protein OS=Homo sapiens GN=FAT1 PE=4 SV=1 - [E9PFR5_HUMAN]	0.52	2	1	1	4,588	506.0	5.00
B9A019	Uncharacterized protein OS=Homo sapiens PE=4 SV=1 - [B9A019_HUMAN]	24.24	2	1	1	99	11.4	6.23
A1YR24	MORC family CW-type zinc finger protein 4 OS=Homo sapiens GN=MORC4 PE=2 SV=1 - [A1YR24_HUMAN]	1.39	5	1	1	648	74.3	8.29
B3KWH2	cDNA FLJ43085 fis, clone BRTHA3018656, highly similar to Homo sapiens solute carrier family 41, member 1 (SLC41A1), mRNA OS=Homo sapiens PE=2 SV=1 - [B3KWH2_HUMAN]	4.41	2	1	1	363	39.3	6.60
E9PQ27	Uncharacterized protein OS=Homo sapiens GN=ILK PE=4 SV=1 - [E9PQ27_HUMAN]	45.00	1	1	1	40	4.6	4.44
E9PFW8	Uncharacterized protein OS=Homo sapiens GN=SQSTM1 PE=4 SV=1 - [E9PFW8_HUMAN]	7.78	1	1	1	167	18.4	8.48
B7Z7B0	cDNA FLJ52791, highly similar to Cleavage and polyadenylation specificityfactor 30 kDa subunit OS=Homo sapiens PE=2 SV=1 - [B7Z7B0_HUMAN]	9.42	3	1	1	191	21.9	8.56
B4E2M1	cDNA FLJ58478, highly similar to Glypican-6 OS=Homo sapiens PE=2 SV=1 - [B4E2M1_HUMAN]	6.07	2	1	1	428	48.5	6.84
C9JJ42	Uncharacterized protein OS=Homo sapiens GN=NAB1 PE=4 SV=1 - [C9JJ42_HUMAN]	27.38	6	1	1	84	9.2	9.66
E9PJ83	Uncharacterized protein OS=Homo sapiens GN=PTPRJ PE=4 SV=1 - [E9PJ83_HUMAN]	10.20	5	1	1	147	15.0	6.06
B4DED1	cDNA FLJ53505, highly similar to Chaperone-activity of bc1 complex-like, mitochondrial OS=Homo sapiens PE=2 SV=1 - [B4DED1_HUMAN]	5.10	6	1	1	314	35.5	9.17
B7ZME5	MYBPHL protein OS=Homo sapiens GN=MYBPHL PE=2 SV=1 - [B7ZME5_HUMAN]	4.23	2	1	1	331	36.2	8.48
O75230	H53_GS1 (Fragment) OS=Homo sapiens PE=4 SV=1 - [O75230_HUMAN]	4.84	1	1	1	537	58.0	8.25
A5HM68	Putative reverse transcriptase (Fragment) OS=Homo sapiens GN=pol PE=2 SV=1 - [A5HM68_HUMAN]	35.29	6	1	1	17	1.9	4.50
B4DH39	cDNA FLJ57028, highly similar to Long-chain-fatty-acid--CoA ligase 4 (EC 6.2.1.3) OS=Homo sapiens PE=2 SV=1 - [B4DH39_HUMAN]	2.31	3	1	1	650	72.1	8.22
P18859	ATP synthase-coupling factor 6, mitochondrial OS=Homo sapiens GN=ATP5J PE=1 SV=1 - [ATP5J_HUMAN]	13.89	2	1	1	108	12.6	9.52
P0C7T5	Ataxin-1-like OS=Homo sapiens GN=ATXN1L PE=1 SV=1 - [ATX1L_HUMAN]	1.60	1	1	1	689	73.3	6.60
Q9BXH1	Bcl-2-binding component 3 OS=Homo sapiens GN=BBC3 PE=1 SV=1 - [BBC3_HUMAN]	3.63	2	1	1	193	20.5	8.78
Q8N143	B-cell CLL/lymphoma 6 member B protein OS=Homo sapiens GN=BCL6B PE=2 SV=2 - [BCL6B_HUMAN]	3.13	2	1	1	479	51.5	8.97
P11586	C-1-tetrahydrofolate synthase, cytoplasmic OS=Homo sapiens GN=MTHFD1 PE=1 SV=3 - [C1TC_HUMAN]	1.07	2	1	1	935	101.5	7.30
Q8TAB7	Putative coiled-coil domain-containing protein 26 OS=Homo sapiens GN=CCDC26 PE=5 SV=1 - [CCD26_HUMAN]	8.26	1	1	1	109	13.2	9.45
Q6P1X6	UPF0598 protein C8orf82 OS=Homo sapiens GN=C8orf82 PE=1 SV=2 - [CH082_HUMAN]	3.70	1	1	1	216	23.9	9.14
Q7L1S5	Carbohydrate sulfotransferase 9 OS=Homo sapiens GN=CHST9 PE=2 SV=2 - [CHST9_HUMAN]	5.42	1	1	1	443	52.0	9.38
Q9H2I8	Leucine-rich repeat-containing protein C10orf11 OS=Homo sapiens GN=C10orf11 PE=1 SV=1 - [CJ011_HUMAN]	3.03	2	1	1	198	22.6	6.70
Q5T742	Uncharacterized protein C10orf25 OS=Homo sapiens GN=C10orf25 PE=2 SV=3 - [CJ025_HUMAN]	9.02	1	1	1	122	14.4	10.23
A6PW82	Putative uncharacterized protein CXorf30 OS=Homo sapiens GN=CXorf30 PE=2 SV=2 - [CX030_HUMAN]	1.42	2	1	1	633	72.0	6.09
Q9BVC3	Sister chromatid cohesion protein DCC1 OS=Homo sapiens GN=DSCC1 PE=1 SV=2 - [DCC1_HUMAN]	4.58	1	1	1	393	44.8	5.17
Q9BTC0	Death-inducer obliterator 1 OS=Homo sapiens GN=DIDO1 PE=1 SV=5 - [DIDO1_HUMAN]	0.94	1	1	1	2,240	243.7	7.88
P51530	DNA2-like helicase OS=Homo sapiens GN=DNA2 PE=1 SV=3 - [DNA2L_HUMAN]	0.94	1	1	1	1,060	120.3	7.74
Q8N350	Protein Dos OS=Homo sapiens GN=DOS PE=1 SV=2 - [DOS_HUMAN]	1.10	1	1	1	725	75.8	6.61
Q8N9I9	Protein deltex-3 OS=Homo sapiens GN=DTX3 PE=2 SV=2 - [DTX3_HUMAN]	2.02	1	1	1	347	38.0	8.73
Q66K89	Transcription factor E4F1 OS=Homo sapiens GN=E4F1 PE=1 SV=2 - [E4F1_HUMAN]	1.53	1	1	1	784	83.4	6.34
Q6ZMW3	Echinoderm microtubule-associated protein-like 6 OS=Homo sapiens GN=EML6 PE=2 SV=2 - [EMAL6_HUMAN]	0.72	1	1	1	1,958	217.8	7.44
Q6ZQQ2	Protein FAM75D1 OS=Homo sapiens GN=FAM75D1 PE=1 SV=1 - [F75D1_HUMAN]	1.40	1	1	1	1,576	175.5	8.85
O60427	Fatty acid desaturase 1 OS=Homo sapiens GN=FADS1 PE=1 SV=1 - [FADS1_HUMAN]	1.35	2	1	1	444	51.9	8.87
Q6P3S6	F-box only protein 42 OS=Homo sapiens GN=FBXO42 PE=1 SV=1 - [FBX42_HUMAN]	1.67	1	1	1	717	77.8	7.43
Q06210	Glucosamine--fructose-6-phosphate aminotransferase [isomerizing] 1 OS=Homo sapiens GN=GFPT1 PE=1 SV=3 - [GFPT1_HUMAN]	2.29	1	1	1	699	78.8	7.11
Q02539	Histone H1.1 OS=Homo sapiens GN=HIST1H1A PE=1 SV=3 - [H11_HUMAN]	8.84	1	1	1	215	21.8	10.99
Q7Z745	HEAT repeat-containing protein 7B2 OS=Homo sapiens GN=HEATR7B2 PE=2 SV=3 - [HTRB2_HUMAN]	0.44	2	1	1	1,585	180.7	6.28
Q68E01	Integrator complex subunit 3 OS=Homo sapiens GN=INTS3 PE=1 SV=1 - [INT3_HUMAN]	2.11	1	1	1	1,043	118.0	5.80
Q9UHH9	Inositol hexakisphosphate kinase 2 OS=Homo sapiens GN=IP6K2 PE=2 SV=2 - [IP6K2_HUMAN]	2.35	2	1	1	426	49.2	6.83
Q6UXX5	Inter-alpha-trypsin inhibitor heavy chain H5-like protein OS=Homo sapiens GN=ITIH5L PE=2 SV=1 - [ITH5L_HUMAN]	1.45	1	1	1	1,313	143.1	9.01
Q9UPP5	Uncharacterized protein KIAA1107 OS=Homo sapiens GN=KIAA1107 PE=1 SV=2 - [K1107_HUMAN]	0.50	1	1	1	1,409	155.6	6.19
P46013	Antigen KI-67 OS=Homo sapiens GN=MKI67 PE=1 SV=2 - [KI67_HUMAN]	0.40	1	1	1	3,256	358.5	9.45
P05771	Protein kinase C beta type OS=Homo sapiens GN=PRKCB PE=1 SV=4 - [KPCB_HUMAN]	2.38	1	1	1	671	76.8	7.01
P01703	Ig lambda chain V-I region NEWM OS=Homo sapiens PE=1 SV=1 - [LV105_HUMAN]	10.68	1	1	1	103	10.9	9.29
Q92918	Mitogen-activated protein kinase kinase kinase kinase 1 OS=Homo sapiens GN=MAP4K1 PE=1 SV=1 - [M4K1_HUMAN]	1.92	1	1	1	833	91.2	8.34
Q71F56	Mediator of RNA polymerase II transcription subunit 13-like OS=Homo sapiens GN=MED13L PE=1 SV=1 - [MD13L_HUMAN]	1.27	1	1	1	2,210	242.4	6.04
O14686	Histone-lysine N-methyltransferase MLL2 OS=Homo sapiens GN=MLL2 PE=1 SV=2 - [MLL2_HUMAN]	0.33	1	1	1	5,537	593.0	5.58
P51511	Matrix metalloproteinase-15 OS=Homo sapiens GN=MMP15 PE=1 SV=1 - [MMP15_HUMAN]	1.05	1	1	1	669	75.8	7.46
Q96JP2	Putative myosin-XVB OS=Homo sapiens GN=MYO15B PE=5 SV=2 - [MY15B_HUMAN]	1.57	1	1	1	1,530	167.0	8.41
P52179	Myomesin-1 OS=Homo sapiens GN=MYOM1 PE=1 SV=2 - [MYOM1_HUMAN]	0.42	1	1	1	1,685	187.5	6.93
Q9UPR5	Sodium/calcium exchanger 2 OS=Homo sapiens GN=SLC8A2 PE=2 SV=2 - [NAC2_HUMAN]	2.28	1	1	1	921	100.3	5.15
Q9UKK3	Poly [ADP-ribose] polymerase 4 OS=Homo sapiens GN=PARP4 PE=1 SV=3 - [PARP4_HUMAN]	0.93	1	1	1	1,724	192.5	5.66
Q9Y5H1	Protocadherin gamma-A2 OS=Homo sapiens GN=PCDHGA2 PE=2 SV=1 - [PCDG2_HUMAN]	0.97	1	1	1	932	101.4	5.01
P11309	Proto-oncogene serine/threonine-protein kinase pim-1 OS=Homo sapiens GN=PIM1 PE=1 SV=3 - [PIM1_HUMAN]	4.21	1	1	1	404	45.4	7.01
O14939	Phospholipase D2 OS=Homo sapiens GN=PLD2 PE=1 SV=2 - [PLD2_HUMAN]	0.75	1	1	1	933	105.9	7.64
P23284	Peptidyl-prolyl cis-trans isomerase B OS=Homo sapiens GN=PPIB PE=1 SV=2 - [PPIB_HUMAN]	4.17	1	1	1	216	23.7	9.41
Q09MP3	RAD51-associated protein 2 OS=Homo sapiens GN=RAD51AP2 PE=1 SV=1 - [R51A2_HUMAN]	1.47	1	1	1	1,159	133.8	7.34
Q6PCD5	E3 ubiquitin-protein ligase RFWD3 OS=Homo sapiens GN=RFWD3 PE=1 SV=3 - [RFWD3_HUMAN]	3.23	1	1	1	774	85.0	6.46
Q8WYP3	Ras and Rab interactor 2 OS=Homo sapiens GN=RIN2 PE=1 SV=1 - [RIN2_HUMAN]	1.34	2	1	1	895	100.1	6.58
Q7Z6J9	tRNA-splicing endonuclease subunit Sen54 OS=Homo sapiens GN=TSEN54 PE=1 SV=3 - [SEN54_HUMAN]	3.42	1	1	1	526	58.8	7.91
Q6UWI4	Protein shisa-2 homolog OS=Homo sapiens GN=SHISA2 PE=1 SV=1 - [SHSA2_HUMAN]	5.42	1	1	1	295	31.4	7.68
B8ZZ34	Putative protein shisa-8 OS=Homo sapiens GN=SHISA8 PE=5 SV=2 - [SHSA8_HUMAN]	4.27	1	1	1	492	51.4	10.83
Q9NXL6	SID1 transmembrane family member 1 OS=Homo sapiens GN=SIDT1 PE=2 SV=2 - [SIDT1_HUMAN]	2.30	1	1	1	827	93.8	7.30
Q8IY92	Structure-specific endonuclease subunit SLX4 OS=Homo sapiens GN=SLX4 PE=1 SV=3 - [SLX4_HUMAN]	0.49	1	1	1	1,834	199.9	6.06
Q9P2F5	Storkhead-box protein 2 OS=Homo sapiens GN=STOX2 PE=2 SV=2 - [STOX2_HUMAN]	0.86	1	1	1	926	102.6	8.40
Q24JP5	Transmembrane protein 132A OS=Homo sapiens GN=TMEM132A PE=2 SV=1 - [T132A_HUMAN]	1.96	1	1	1	1,023	110.0	5.62
Q12962	Transcription initiation factor TFIID subunit 10 OS=Homo sapiens GN=TAF10 PE=1 SV=1 - [TAF10_HUMAN]	4.59	1	1	1	218	21.7	6.57
Q9Y5Q9	General transcription factor 3C polypeptide 3 OS=Homo sapiens GN=GTF3C3 PE=1 SV=1 - [TF3C3_HUMAN]	2.48	1	1	1	886	101.2	5.07
Q6UXN7	TOMM20-like protein 1 OS=Homo sapiens GN=TOMM20L PE=2 SV=1 - [TO20L_HUMAN]	11.18	1	1	1	152	17.7	8.59
Q8TD43	Transient receptor potential cation channel subfamily M member 4 OS=Homo sapiens GN=TRPM4 PE=1 SV=1 - [TRPM4_HUMAN]	1.89	1	1	1	1,214	134.2	8.15
Q9HBA0	Transient receptor potential cation channel subfamily V member 4 OS=Homo sapiens GN=TRPV4 PE=1 SV=2 - [TRPV4_HUMAN]	1.26	1	1	1	871	98.2	7.77
A6NEE1	UPF0639 protein OS=Homo sapiens PE=3 SV=2 - [U639_HUMAN]	1.98	2	1	1	506	59.1	6.44
Q9Y493	Zonadhesin OS=Homo sapiens GN=ZAN PE=2 SV=4 - [ZAN_HUMAN]	0.25	1	1	1	2,812	305.5	6.11
Q8N7K0	Zinc finger protein 433 OS=Homo sapiens GN=ZNF433 PE=2 SV=1 - [ZN433_HUMAN]	4.31	1	1	1	673	77.2	9.20
Q2MD44	B-cell linker protein (Fragment) OS=Homo sapiens GN=BASH PE=2 SV=1 - [Q2MD44_HUMAN]	14.63	19	1	1	41	4.8	10.21
B4DX48	Transporter OS=Homo sapiens GN=SLC6A2 PE=2 SV=1 - [B4DX48_HUMAN]	1.95	3	1	1	512	57.3	6.74
Q96LV9	Transient receptor potential cation channel, subfamily M, member 6 OS=Homo sapiens GN=TRPM6 PE=2 SV=1 - [Q96LV9_HUMAN]	2.97	3	1	1	303	34.3	8.82
B7ZBA8	Novel protein OS=Homo sapiens GN=CCDC154 PE=4 SV=1 - [B7ZBA8_HUMAN]	2.11	2	1	1	522	59.4	9.26
A2A2C6	Amine oxidase (Flavin containing) domain 1 OS=Homo sapiens GN=AOF1 PE=4 SV=1 - [A2A2C6_HUMAN]	2.20	3	1	1	590	65.7	8.51
A2AB25	PHD finger protein 1 (Fragment) OS=Homo sapiens GN=PHF1 PE=4 SV=1 - [A2AB25_HUMAN]	13.26	1	1	1	181	18.6	10.93
C3W5P5	TLR9 (Fragment) OS=Homo sapiens GN=TLR9 PE=4 SV=1 - [C3W5P5_HUMAN]	0.87	6	1	1	1,031	115.7	8.19
B4DMI6	WDR45-like, isoform CRA_f OS=Homo sapiens GN=WDR45L PE=2 SV=1 - [B4DMI6_HUMAN]	9.81	2	1	1	316	35.2	7.81
B1AK81	Phosphatidylinositol glycan anchor biosynthesis, class K OS=Homo sapiens GN=PIGK PE=4 SV=1 - [B1AK81_HUMAN]	5.65	4	1	1	301	34.3	5.81
B4DZX7	Thioredoxin domain containing, isoform CRA_b OS=Homo sapiens GN=TXNDC PE=2 SV=1 - [B4DZX7_HUMAN]	5.10	2	1	1	196	22.4	5.71
Q5TBP5	TAF4 RNA polymerase II, TATA box binding protein (TBP)-associated factor, 135kDa (Fragment) OS=Homo sapiens GN=TAF4 PE=2 SV=1 - [Q5TBP5_HUMAN]	3.06	2	1	1	949	97.3	10.13
Q5J7V3	Cell migration-inducing protein 20 OS=Homo sapiens PE=4 SV=1 - [Q5J7V3_HUMAN]	9.38	11	1	1	64	7.4	8.57
Q96C71	GNAI2 protein OS=Homo sapiens GN=GNAI2 PE=2 SV=1 - [Q96C71_HUMAN]	3.10	1	1	1	355	40.5	5.54
Q6NXE4	BRD4 protein (Fragment) OS=Homo sapiens GN=BRD4 PE=2 SV=1 - [Q6NXE4_HUMAN]	1.82	7	1	1	548	60.8	8.75
A5PKX5	MAN2A1 protein OS=Homo sapiens GN=MAN2A1 PE=2 SV=1 - [A5PKX5_HUMAN]	1.22	2	1	1	1,143	131.0	7.58
A6NM42	Uncharacterized protein OS=Homo sapiens GN=SERINC4 PE=4 SV=1 - [A6NM42_HUMAN]	2.11	1	1	1	426	46.1	9.28
D3YTH6	Uncharacterized protein OS=Homo sapiens GN=FAM22G PE=4 SV=1 - [D3YTH6_HUMAN]	2.54	5	1	1	590	61.8	8.35
C9JZJ2	Uncharacterized protein OS=Homo sapiens GN=BRCC3 PE=4 SV=1 - [C9JZJ2_HUMAN]	8.90	1	1	1	292	33.4	6.19
B4DXD5	cDNA FLJ51556, highly similar to AF4/FMR2 family member 2 OS=Homo sapiens PE=2 SV=1 - [B4DXD5_HUMAN]	1.79	3	1	1	952	105.1	9.28
B7Z260	cDNA FLJ53468, highly similar to Protein FAM5C OS=Homo sapiens PE=2 SV=1 - [B7Z260_HUMAN]	1.96	1	1	1	664	76.2	7.02
Q53T35	Putative uncharacterized protein PTHR2 (Fragment) OS=Homo sapiens GN=PTHR2 PE=2 SV=1 - [Q53T35_HUMAN]	4.28	4	1	1	327	37.0	6.83
Q5T1V5	DEAD (Asp-Glu-Ala-Asp) box polypeptide 59 (Fragment) OS=Homo sapiens GN=DDX59 PE=2 SV=1 - [Q5T1V5_HUMAN]	9.94	3	1	1	161	17.7	5.31
E7ERT1	Uncharacterized protein OS=Homo sapiens GN=ZNF586 PE=4 SV=1 - [E7ERT1_HUMAN]	3.49	2	1	1	258	27.8	9.42
A2IDC7	Mitochondrial ribosomal protein L28 (Fragment) OS=Homo sapiens GN=MRPL28 PE=4 SV=1 - [A2IDC7_HUMAN]	9.79	5	1	1	194	23.1	9.36
C9JNR0	Uncharacterized protein OS=Homo sapiens GN=IL1R2 PE=4 SV=1 - [C9JNR0_HUMAN]	8.30	4	1	1	229	26.3	7.40
Q6ZUQ1	cDNA FLJ43465 fis, clone OCBBF2036476 OS=Homo sapiens PE=2 SV=1 - [Q6ZUQ1_HUMAN]	5.86	1	1	1	256	27.2	9.94
B4DL88	cDNA FLJ52323, highly similar to Mastermind-like protein 3 (Mam-3) OS=Homo sapiens PE=2 SV=1 - [B4DL88_HUMAN]	1.80	3	1	1	445	47.3	9.52
B7Z698	cDNA FLJ50420, highly similar to Transmembrane protein 106A OS=Homo sapiens PE=2 SV=1 - [B7Z698_HUMAN]	3.27	3	1	1	214	23.8	6.77
A4F3V9	Cytochrome P450 1A1 OS=Homo sapiens GN=CYP1A1 PE=2 SV=1 - [A4F3V9_HUMAN]	9.55	6	1	1	157	18.4	7.59
B7Z255	cDNA FLJ59500, highly similar to Glycogen (starch) synthase, muscle (EC 2.4.1.11) OS=Homo sapiens PE=2 SV=1 - [B7Z255_HUMAN]	4.05	7	1	1	370	42.1	6.21
B0AZU3	cDNA, FLJ79533, highly similar to Bcl-2-associated transcription factor 1 OS=Homo sapiens PE=2 SV=1 - [B0AZU3_HUMAN]	7.97	3	1	1	138	16.2	9.63
Q6QNA5	Von Willebrand factor-cleaving protease (Fragment) OS=Homo sapiens GN=ADAMTS13 PE=2 SV=1 - [Q6QNA5_HUMAN]	43.48	1	1	1	23	2.6	11.69
Q5H8Y7	L(3)mbt-like (Drosophila) (Fragment) OS=Homo sapiens GN=L3MBTL PE=2 SV=1 - [Q5H8Y7_HUMAN]	6.25	1	1	1	288	32.0	7.03
B2R769	cDNA, FLJ93308, highly similar to Homo sapiens leukocyte-derived arginine aminopeptidase (LRAP), mRNA OS=Homo sapiens PE=2 SV=1 - [B2R769_HUMAN]	0.94	2	1	1	960	110.4	6.77
B4DUD8	cDNA FLJ57597 OS=Homo sapiens PE=2 SV=1 - [B4DUD8_HUMAN]	1.39	4	1	1	789	89.0	6.68
C6GLX4	Putative uncharacterized protein (Fragment) OS=Homo sapiens PE=4 SV=1 - [C6GLX4_HUMAN]	8.89	1	1	1	180	20.6	8.84
B7Z1I0	cDNA FLJ53825, highly similar to Integrin-linked protein kinase 1 (EC 2.7.11.1) OS=Homo sapiens PE=2 SV=1 - [B7Z1I0_HUMAN]	2.20	3	1	1	318	36.4	9.41
B2RCT7	cDNA, FLJ96279, highly similar to Homo sapiens solute carrier family 2 (facilitated glucosetransporter), member 10 (SLC2A10), mRNA OS=Homo sapiens PE=2 SV=1 - [B2RCT7_HUMAN]	1.48	3	1	1	541	56.8	8.59
A0A575	V_segment translation product (Fragment) OS=Homo sapiens GN=TCRBV22S1A2N1T PE=4 SV=1 - [A0A575_HUMAN]	18.10	1	1	1	116	13.5	4.84
Q59H94	Gamma filamin variant (Fragment) OS=Homo sapiens PE=1 SV=1 - [Q59H94_HUMAN]	0.52	2	1	1	1,342	142.7	6.46
E9PLH5	Uncharacterized protein OS=Homo sapiens GN=ST5 PE=4 SV=1 - [E9PLH5_HUMAN]	2.44	1	1	1	328	37.6	6.99
E9PQ61	Uncharacterized protein OS=Homo sapiens GN=ZC3H11A PE=4 SV=1 - [E9PQ61_HUMAN]	1.07	3	1	1	652	72.5	8.63
Q96JJ8	KIAA1829 protein (Fragment) OS=Homo sapiens GN=KIAA1829 PE=2 SV=1 - [Q96JJ8_HUMAN]	2.33	2	1	1	557	64.7	8.16
E9PAQ0	Uncharacterized protein OS=Homo sapiens GN=TASP1 PE=4 SV=1 - [E9PAQ0_HUMAN]	4.79	2	1	1	397	42.1	8.13
A3KFI1	Neuroblastoma, suppression of tumorigenicity 1 (Fragment) OS=Homo sapiens GN=NBL1 PE=4 SV=1 - [A3KFI1_HUMAN]	7.81	1	1	1	128	14.0	6.48
B4DF61	cDNA FLJ53309, highly similar to Methionyl-tRNA synthetase (EC 6.1.1.10) OS=Homo sapiens PE=2 SV=1 - [B4DF61_HUMAN]	2.05	3	1	1	635	71.8	7.14
Q9NSI4	PRED42 protein (Fragment) OS=Homo sapiens GN=PRED42 PE=4 SV=1 - [Q9NSI4_HUMAN]	5.54	1	1	1	325	35.5	7.62
E9PHM5	Uncharacterized protein OS=Homo sapiens GN=CACNA1H PE=4 SV=1 - [E9PHM5_HUMAN]	1.11	2	1	1	2,347	258.4	7.46
B4E1P1	cDNA FLJ51451, highly similar to Semaphorin-3E OS=Homo sapiens PE=2 SV=1 - [B4E1P1_HUMAN]	1.26	3	1	1	715	82.4	7.43
B2RD71	cDNA, FLJ96486, highly similar to Homo sapiens DEAH (Asp-Glu-Ala-His) box polypeptide 32 (DHX32), mRNA OS=Homo sapiens PE=2 SV=1 - [B2RD71_HUMAN]	1.08	2	1	1	743	84.4	4.97
B7Z2B7	cDNA FLJ60493 OS=Homo sapiens PE=2 SV=1 - [B7Z2B7_HUMAN]	2.99	6	1	1	201	22.1	5.08
B2RAK1	cDNA, FLJ94965, highly similar to Homo sapiens leucyl/cystinyl aminopeptidase (LNPEP), mRNA OS=Homo sapiens PE=2 SV=1 - [B2RAK1_HUMAN]	1.37	2	1	1	1,025	117.3	5.73
B3KTP7	cDNA FLJ38566 fis, clone HCHON2005118, highly similar to Collagen alpha-1(XV) chain OS=Homo sapiens PE=2 SV=1 - [B3KTP7_HUMAN]	2.01	2	1	1	745	75.3	4.31
B2R9J0	cDNA, FLJ94418, highly similar to Homo sapiens inositol 1,4,5-trisphosphate 3-kinase B (ITPKB), mRNA OS=Homo sapiens PE=2 SV=1 - [B2R9J0_HUMAN]	1.69	4	1	1	472	53.5	6.49
B3KRA9	cDNA FLJ33948 fis, clone CTONG2018379, highly similar to Hexokinase-1 (EC 2.7.1.1) OS=Homo sapiens PE=2 SV=1 - [B3KRA9_HUMAN]	1.24	8	1	1	566	63.3	7.91
D6RFF5	Uncharacterized protein OS=Homo sapiens GN=ANKRD37 PE=4 SV=1 - [D6RFF5_HUMAN]	38.64	4	1	1	44	4.5	5.00
E9PB65	Uncharacterized protein OS=Homo sapiens GN=SCOC PE=4 SV=1 - [E9PB65_HUMAN]	12.98	1	1	1	131	14.8	9.99
Q6ZW19	cDNA FLJ41761 fis, clone IMR322003675 OS=Homo sapiens PE=2 SV=1 - [Q6ZW19_HUMAN]	3.10	1	1	1	258	27.2	12.10
A0AV47	Topoisomerase (DNA) II binding protein 1 OS=Homo sapiens GN=TOPBP1 PE=2 SV=2 - [A0AV47_HUMAN]	0.63	3	1	1	1,435	160.5	6.99
B7Z356	cDNA FLJ50157, highly similar to Brain-specific angiogenesis inhibitor 3 OS=Homo sapiens PE=2 SV=1 - [B7Z356_HUMAN]	0.96	2	1	1	728	81.7	7.55
Q8NAG4	cDNA FLJ35392 fis, clone SKNSH2000716 OS=Homo sapiens PE=2 SV=1 - [Q8NAG4_HUMAN]	10.74	1	1	1	149	16.1	8.60
E5RIN8	Uncharacterized protein OS=Homo sapiens GN=INTS8 PE=4 SV=2 - [E5RIN8_HUMAN]	11.76	4	1	1	204	22.7	8.10
E7EN27	Uncharacterized protein OS=Homo sapiens GN=GNRHR2 PE=4 SV=1 - [E7EN27_HUMAN]	2.05	2	1	1	292	32.5	9.26
B5MDP1	Uncharacterized protein OS=Homo sapiens GN=C9orf102 PE=4 SV=1 - [B5MDP1_HUMAN]	0.99	2	1	1	607	69.4	9.14
B4DSR3	cDNA FLJ52893, highly similar to Homo sapiens DDHD domain containing 2 (DDHD2), mRNA OS=Homo sapiens PE=2 SV=1 - [B4DSR3_HUMAN]	2.95	3	1	1	271	30.7	5.38
B0QYM2	Chromosome 22 open reading frame 23 (Fragment) OS=Homo sapiens GN=C22orf23 PE=4 SV=1 - [B0QYM2_HUMAN]	5.83	3	1	1	120	13.6	10.23
D6RAI2	Uncharacterized protein OS=Homo sapiens GN=MAPKBP1 PE=4 SV=1 - [D6RAI2_HUMAN]	15.94	5	1	1	69	7.7	9.28
B7Z7P0	cDNA FLJ51394, highly similar to Ubiquitin conjugation factor E4 A OS=Homo sapiens PE=2 SV=1 - [B7Z7P0_HUMAN]	2.79	2	1	1	538	62.0	5.68
B4E0B7	Condensin complex subunit 1 OS=Homo sapiens PE=2 SV=1 - [B4E0B7_HUMAN]	1.25	4	1	1	1,356	152.1	6.74
B4DW07	cDNA FLJ50032, highly similar to Homo sapiens spectrin domain with coiled-coils 1 (SPECC1), transcript variant, mRNA OS=Homo sapiens PE=2 SV=1 - [B4DW07_HUMAN]	2.21	6	1	1	408	45.7	8.34
B7Z5C2	cDNA FLJ52972, weakly similar to Homo sapiens abhydrolase domain containing 3 (ABHD3), mRNA OS=Homo sapiens PE=2 SV=1 - [B7Z5C2_HUMAN]	13.24	1	1	1	136	15.5	9.80
D6RGF3	Uncharacterized protein OS=Homo sapiens GN=FAM13C PE=4 SV=1 - [D6RGF3_HUMAN]	2.05	3	1	1	488	55.0	7.36
Q8NHB3	Seven transmembrane helix receptor OS=Homo sapiens PE=2 SV=1 - [Q8NHB3_HUMAN]	2.81	2	1	1	249	28.3	8.37
Q8N882	cDNA FLJ39841 fis, clone SPLEN2014210, moderately similar to Rattus norvegicus alpha D integrin mRNA OS=Homo sapiens PE=2 SV=1 - [Q8N882_HUMAN]	4.33	4	1	1	231	24.8	9.42
B7Z6C3	cDNA FLJ56151, highly similar to Platelet glycoprotein 4 OS=Homo sapiens PE=2 SV=1 - [B7Z6C3_HUMAN]	1.52	11	1	1	396	44.7	8.22
B4E3F1	cDNA FLJ61573, highly similar to Protein LAP2 OS=Homo sapiens PE=2 SV=1 - [B4E3F1_HUMAN]	0.97	7	1	1	1,346	151.1	5.41
A6NMK2	Uncharacterized protein OS=Homo sapiens GN=HORMAD1 PE=4 SV=2 - [A6NMK2_HUMAN]	2.54	2	1	1	394	45.2	5.80
A4VCI6	HIP1 protein (Fragment) OS=Homo sapiens GN=HIP1 PE=2 SV=1 - [A4VCI6_HUMAN]	1.74	5	1	1	517	57.7	4.77
C9JYC0	Uncharacterized protein OS=Homo sapiens GN=C3orf78 PE=4 SV=1 - [C9JYC0_HUMAN]	43.24	1	1	1	37	4.4	8.66
E9PPA7	Uncharacterized protein OS=Homo sapiens GN=PKD1L2 PE=4 SV=1 - [E9PPA7_HUMAN]	0.64	4	1	1	1,095	120.9	5.57
A2A492	BCL2/adenovirus E1B 19kD interacting protein like OS=Homo sapiens GN=BNIPL PE=4 SV=1 - [A2A492_HUMAN]	5.63	2	1	1	355	39.5	5.41
B7WP88	Uncharacterized protein OS=Homo sapiens GN=TRANK1 PE=4 SV=2 - [B7WP88_HUMAN]	1.09	2	1	1	2,375	273.3	6.64
B3KPL4	cDNA FLJ31915 fis, clone NT2RP7004911, highly similar to Zinc finger protein 336 OS=Homo sapiens PE=2 SV=1 - [B3KPL4_HUMAN]	1.41	2	1	1	711	80.4	7.93
Q9HBB2	Iron regulatory protein 1 OS=Homo sapiens GN=IRP1 PE=2 SV=1 - [Q9HBB2_HUMAN]	0.89	2	1	1	790	87.0	6.55
Q6LDR0	Triiodothyronine receptor (Fragment) OS=Homo sapiens PE=2 SV=1 - [Q6LDR0_HUMAN]	12.00	6	1	1	75	9.0	6.79
B4E1Y9	cDNA FLJ54321, highly similar to MAM domain-containing protein 2 OS=Homo sapiens PE=2 SV=1 - [B4E1Y9_HUMAN]	1.90	3	1	1	580	65.1	5.06
B4DI95	cDNA FLJ52955, moderately similar to Homo sapiens solute carrier family 25 (mitochondrial carrier; phosphate carrier), member 24 (SLC25A24), transcript variant 2, mRNA OS=Homo sapiens PE=2 SV=1 - [B4DI95_HUMAN]	3.93	3	1	1	229	25.0	10.10
B2R6Y6	cDNA, FLJ93181 OS=Homo sapiens PE=2 SV=1 - [B2R6Y6_HUMAN]	3.32	2	1	1	241	27.4	6.98
B3KR30	cDNA FLJ33557 fis, clone BRAMY2009489, highly similar to Homo sapiens SPHK1 (sphingosine kinase type 1) interacting protein (SKIP), mRNA OS=Homo sapiens PE=2 SV=1 - [B3KR30_HUMAN]	2.56	2	1	1	702	77.6	5.38
B4E011	cDNA FLJ51422, highly similar to Interleukin-9 receptor OS=Homo sapiens PE=2 SV=1 - [B4E011_HUMAN]	9.42	2	1	1	329	36.7	6.15
B4DWS1	cDNA FLJ51125, highly similar to Alcohol dehydrogenase class 4 mu/sigma chain (EC 1.1.1.1) OS=Homo sapiens PE=2 SV=1 - [B4DWS1_HUMAN]	3.42	4	1	1	234	24.9	6.76
A8K1E9	cDNA FLJ78497 OS=Homo sapiens PE=2 SV=1 - [A8K1E9_HUMAN]	1.64	3	1	1	489	54.2	7.11
B2RAH7	cDNA, FLJ94921, highly similar to Homo sapiens prolyl endopeptidase (PREP), mRNA OS=Homo sapiens PE=2 SV=1 - [B2RAH7_HUMAN]	0.99	3	1	1	710	80.7	5.86
Q9HDA3	Glycine decarboxylase P-protein (Fragment) OS=Homo sapiens PE=2 SV=2 - [Q9HDA3_HUMAN]	6.34	2	1	1	331	36.5	6.52
Q5VXM9	Brix domain containing 1 (Fragment) OS=Homo sapiens GN=BXDC1 PE=2 SV=1 - [Q5VXM9_HUMAN]	8.17	4	1	1	208	24.1	9.47
C9K0K8	Uncharacterized protein OS=Homo sapiens GN=FAM82A1 PE=4 SV=1 - [C9K0K8_HUMAN]	3.79	4	1	1	211	24.8	7.58
B4DXV6	cDNA FLJ60193, highly similar to Melanoma-derived growth regulatory protein OS=Homo sapiens PE=2 SV=1 - [B4DXV6_HUMAN]	15.27	2	1	1	131	14.3	8.98
A8K2X6	cDNA FLJ77840, highly similar to Homo sapiens solute carrier family 43, member 3 (SLC43A3), mRNA OS=Homo sapiens PE=2 SV=1 - [A8K2X6_HUMAN]	4.89	5	1	1	491	54.5	8.53
B4DY78	cDNA FLJ59827, highly similar to Homo sapiens translin-associated factor X interacting protein 1 (TSNAXIP1), mRNA OS=Homo sapiens PE=2 SV=1 - [B4DY78_HUMAN]	5.14	4	1	1	350	40.6	4.98
C9JT72	Uncharacterized protein OS=Homo sapiens GN=PMFBP1 PE=4 SV=1 - [C9JT72_HUMAN]	1.63	4	1	1	797	92.7	6.28
E7ET94	Uncharacterized protein OS=Homo sapiens GN=ABP1 PE=3 SV=1 - [E7ET94_HUMAN]	11.40	9	1	1	193	22.9	9.80
B4DFM6	cDNA FLJ50472, highly similar to Homo sapiens HECT domain and ankyrin repeat containing, E3 ubiquitin protein ligase 1 (HACE1), mRNA OS=Homo sapiens PE=2 SV=1 - [B4DFM6_HUMAN]	3.27	5	1	1	398	45.2	5.08
E9PKB9	Uncharacterized protein OS=Homo sapiens GN=SNX19 PE=4 SV=1 - [E9PKB9_HUMAN]	1.87	2	1	1	803	87.3	5.08
Q59GL0	Rearranged L-myc fusion sequence variant (Fragment) OS=Homo sapiens PE=2 SV=1 - [Q59GL0_HUMAN]	0.87	2	1	1	1,608	184.6	7.12
A4D152	Similar to Neuronal protein 3.1 (P311 protein) OS=Homo sapiens GN=LOC392642 PE=4 SV=1 - [A4D152_HUMAN]	19.85	1	1	1	136	15.5	9.61
B4DRV7	cDNA FLJ51539, highly similar to Vitamin D3 receptor OS=Homo sapiens PE=2 SV=1 - [B4DRV7_HUMAN]	2.53	3	1	1	395	44.7	6.52
B4DYE3	cDNA FLJ58091, highly similar to Chloride channel protein 2 OS=Homo sapiens PE=2 SV=1 - [B4DYE3_HUMAN]	3.11	6	1	1	450	49.9	9.22
B3KXE7	cDNA FLJ45304 fis, clone BRHIP3003984, highly similar to IkappaB kinase complex-associated protein OS=Homo sapiens PE=2 SV=1 - [B3KXE7_HUMAN]	0.81	6	1	1	983	111.4	6.27
B4DZ23	cDNA FLJ50302, highly similar to Probable G-protein coupled receptor 176 OS=Homo sapiens PE=2 SV=1 - [B4DZ23_HUMAN]	4.04	2	1	1	470	52.3	8.79
B7Z2U6	cDNA FLJ59304, highly similar to Cadherin-12 OS=Homo sapiens PE=2 SV=1 - [B7Z2U6_HUMAN]	2.65	1	1	1	754	83.9	4.79
C9JGI7	Uncharacterized protein OS=Homo sapiens GN=ESYT2 PE=4 SV=1 - [C9JGI7_HUMAN]	3.04	2	1	1	527	59.4	8.00
C9JYZ0	Uncharacterized protein OS=Homo sapiens GN=ACY1 PE=4 SV=1 - [C9JYZ0_HUMAN]	5.26	8	1	1	228	25.8	6.37
B4DUP2	cDNA FLJ56155, highly similar to UTP--glucose-1-phosphate uridylyltransferase 2 (EC 2.7.7.9) OS=Homo sapiens PE=2 SV=1 - [B4DUP2_HUMAN]	2.71	2	1	1	517	57.8	8.13
A7E243	CACNA2D4 protein OS=Homo sapiens GN=CACNA2D4 PE=2 SV=1 - [A7E243_HUMAN]	1.33	3	1	1	601	68.0	5.68
D6RAH8	Uncharacterized protein OS=Homo sapiens GN=MARVELD2 PE=4 SV=1 - [D6RAH8_HUMAN]	4.83	6	1	1	145	16.3	5.94
Q9H715	CDNA: FLJ21558 fis, clone COL06372 OS=Homo sapiens PE=2 SV=1 - [Q9H715_HUMAN]	13.04	1	1	1	138	15.3	9.20
O14572	WUGSC:H_248O15.1 protein (Fragment) OS=Homo sapiens GN=WUGSC:H_248O15.1 PE=2 SV=1 - [O14572_HUMAN]	0.43	2	1	1	1,849	206.7	6.25
Q9H051	Putative uncharacterized protein DKFZp547M202 (Fragment) OS=Homo sapiens GN=DKFZp547M202 PE=2 SV=1 - [Q9H051_HUMAN]	7.26	10	1	1	124	14.3	8.97
B3KX25	cDNA FLJ44528 fis, clone UTERU3003523, highly similar to Dedicator of cytokinesis protein 9 OS=Homo sapiens PE=2 SV=1 - [B3KX25_HUMAN]	0.72	10	1	1	1,253	142.6	7.71
Q59GJ9	Acetyl-CoA carboxylase 2 variant (Fragment) OS=Homo sapiens PE=2 SV=1 - [Q59GJ9_HUMAN]	0.36	3	1	1	1,689	192.2	6.60
C9J137	Uncharacterized protein OS=Homo sapiens GN=SH3BP4 PE=4 SV=1 - [C9J137_HUMAN]	1.63	3	1	1	552	60.3	5.71
B7Z1E8	cDNA FLJ56847, highly similar to Segment polarity protein dishevelled homologDVL-1 OS=Homo sapiens PE=2 SV=1 - [B7Z1E8_HUMAN]	1.45	1	1	1	619	66.8	7.40
B3KQH4	cDNA FLJ90464 fis, clone NT2RP3002281, highly similar to RNA-binding protein 12 OS=Homo sapiens PE=2 SV=1 - [B3KQH4_HUMAN]	1.58	4	1	1	568	59.1	8.51
B2RBW9	cDNA, FLJ95746, highly similar to Homo sapiens inhibin, beta C (INHBC), mRNA OS=Homo sapiens PE=2 SV=1 - [B2RBW9_HUMAN]	2.27	2	1	1	352	38.2	7.11
Q8NEX1	Ells2 (Fragment) OS=Homo sapiens PE=2 SV=1 - [Q8NEX1_HUMAN]	10.56	2	1	1	180	18.8	6.27
Q59HG5	Zinc finger protein 192 variant (Fragment) OS=Homo sapiens PE=2 SV=1 - [Q59HG5_HUMAN]	2.27	2	1	1	441	49.8	8.59
B7WPP2	Uncharacterized protein OS=Homo sapiens GN=FHAD1 PE=4 SV=1 - [B7WPP2_HUMAN]	3.22	1	1	1	466	53.7	9.20
B3KSR7	cDNA FLJ36831 fis, clone ASTRO2010615, highly similar to Calpain-5 (EC 3.4.22.-) OS=Homo sapiens PE=2 SV=1 - [B3KSR7_HUMAN]	1.80	5	1	1	557	64.0	7.25
B3KU89	cDNA FLJ39376 fis, clone PERIC1000025, highly similar to DNA topoisomerase 3-beta-1 OS=Homo sapiens PE=2 SV=1 - [B3KU89_HUMAN]	4.00	3	1	1	275	30.9	7.99
B4DTH5	cDNA FLJ55592, weakly similar to Sel-1 homolog OS=Homo sapiens PE=2 SV=1 - [B4DTH5_HUMAN]	1.11	4	1	1	539	60.7	5.99
B7ZKW0	ATP1B4 protein OS=Homo sapiens GN=ATP1B4 PE=2 SV=1 - [B7ZKW0_HUMAN]	4.46	4	1	1	314	36.7	4.74
O94929	Actin-binding LIM protein 3 OS=Homo sapiens GN=ABLIM3 PE=1 SV=3 - [ABLM3_HUMAN]	3.51	1	1	1	683	77.8	8.54
Q3I5F7	Putative acyl-coenzyme A thioesterase 6 OS=Homo sapiens GN=ACOT6 PE=1 SV=1 - [ACOT6_HUMAN]	2.90	1	1	1	207	23.0	8.63
O15085	Rho guanine nucleotide exchange factor 11 OS=Homo sapiens GN=ARHGEF11 PE=1 SV=1 - [ARHGB_HUMAN]	1.18	2	1	1	1,522	167.6	5.50
Q8NCU7	C2 calcium-dependent domain-containing protein 4A OS=Homo sapiens GN=C2CD4A PE=2 SV=2 - [C2C4A_HUMAN]	3.52	1	1	1	369	39.7	11.28
Q4AC94	C2 domain-containing protein 3 OS=Homo sapiens GN=C2CD3 PE=1 SV=4 - [C2CD3_HUMAN]	0.42	1	1	1	2,353	260.2	7.12
Q5RHP9	Uncharacterized protein C1orf173 OS=Homo sapiens GN=C1orf173 PE=2 SV=1 - [CA173_HUMAN]	1.18	1	1	1	1,530	168.4	4.88
Q96MW1	Coiled-coil domain-containing protein 43 OS=Homo sapiens GN=CCDC43 PE=1 SV=2 - [CCD43_HUMAN]	3.13	2	1	1	224	25.2	4.92
Q6ZRK6	Coiled-coil domain-containing protein 73 OS=Homo sapiens GN=CCDC73 PE=1 SV=2 - [CCD73_HUMAN]	1.39	1	1	1	1,079	124.1	5.58
Q86XR8	Centrosomal protein of 57 kDa OS=Homo sapiens GN=CEP57 PE=1 SV=2 - [CEP57_HUMAN]	4.60	1	1	1	500	57.1	9.31
Q9H8Q6	Putative uncharacterized protein C15orf34 OS=Homo sapiens GN=C15orf34 PE=5 SV=1 - [CO034_HUMAN]	11.51	1	1	1	139	16.1	8.87
A6NKP2	Putative short-chain dehydrogenase/reductase family 42E member 2 OS=Homo sapiens GN=SDR42E2 PE=3 SV=3 - [D42E2_HUMAN]	5.69	1	1	1	422	46.8	9.63
Q6ZS02	Putative GED domain-containing protein DNM1P46 OS=Homo sapiens GN=DNM1P46 PE=5 SV=1 - [DMP46_HUMAN]	7.27	1	1	1	220	23.7	8.24
Q9UBS3	DnaJ homolog subfamily B member 9 OS=Homo sapiens GN=DNAJB9 PE=1 SV=1 - [DNJB9_HUMAN]	4.48	1	1	1	223	25.5	8.27
Q9C0G6	Dynein heavy chain 6, axonemal OS=Homo sapiens GN=DNAH6 PE=1 SV=3 - [DYH6_HUMAN]	0.38	1	1	1	4,158	475.7	6.00
P08246	Neutrophil elastase OS=Homo sapiens GN=ELANE PE=1 SV=1 - [ELNE_HUMAN]	10.49	1	1	1	267	28.5	9.35
Q9UNN8	Endothelial protein C receptor OS=Homo sapiens GN=PROCR PE=1 SV=1 - [EPCR_HUMAN]	3.78	1	1	1	238	26.7	7.18
Q2M3D2	Exocyst complex component 3-like protein 2 OS=Homo sapiens GN=EXOC3L2 PE=2 SV=1 - [EX3L2_HUMAN]	2.93	1	1	1	409	45.8	7.71
Q8IYI6	Exocyst complex component 8 OS=Homo sapiens GN=EXOC8 PE=1 SV=2 - [EXOC8_HUMAN]	0.83	1	1	1	725	81.7	5.49
A8MYZ0	Protein FAM188B2 OS=Homo sapiens GN=FAM188B2 PE=3 SV=2 - [F1882_HUMAN]	3.06	1	1	1	360	41.0	7.91
Q9UK73	Protein fem-1 homolog B OS=Homo sapiens GN=FEM1B PE=1 SV=1 - [FEM1B_HUMAN]	1.44	1	1	1	627	70.2	6.61
O15552	Free fatty acid receptor 2 OS=Homo sapiens GN=FFAR2 PE=2 SV=1 - [FFAR2_HUMAN]	8.48	1	1	1	330	37.1	9.39
Q8IX07	Zinc finger protein ZFPM1 OS=Homo sapiens GN=ZFPM1 PE=1 SV=2 - [FOG1_HUMAN]	3.68	1	1	1	1,006	104.8	7.87
P05362	Intercellular adhesion molecule 1 OS=Homo sapiens GN=ICAM1 PE=1 SV=2 - [ICAM1_HUMAN]	1.88	2	1	1	532	57.8	7.99
Q8WYK2	Jun dimerization protein 2 OS=Homo sapiens GN=JDP2 PE=1 SV=1 - [JDP2_HUMAN]	3.68	1	1	1	163	18.7	9.23
O60303	Uncharacterized protein KIAA0556 OS=Homo sapiens GN=KIAA0556 PE=1 SV=4 - [K0556_HUMAN]	1.05	1	1	1	1,618	180.8	5.87
P23352	Anosmin-1 OS=Homo sapiens GN=KAL1 PE=1 SV=3 - [KALM_HUMAN]	1.76	1	1	1	680	76.1	9.16
Q6UW63	KDEL motif-containing protein 1 OS=Homo sapiens GN=KDELC1 PE=1 SV=1 - [KDEL1_HUMAN]	3.78	1	1	1	502	58.0	7.71
O94910	Latrophilin-1 OS=Homo sapiens GN=LPHN1 PE=1 SV=1 - [LPHN1_HUMAN]	0.95	1	1	1	1,474	162.6	6.60
Q9HAR2	Latrophilin-3 OS=Homo sapiens GN=LPHN3 PE=1 SV=2 - [LPHN3_HUMAN]	1.31	1	1	1	1,447	161.7	6.44
Q9BZ81	Melanoma-associated antigen B5 OS=Homo sapiens GN=MAGEB5 PE=2 SV=2 - [MAGB5_HUMAN]	5.09	1	1	1	275	31.9	7.65
Q5JRA6	Melanoma inhibitory activity protein 3 OS=Homo sapiens GN=MIA3 PE=1 SV=1 - [MIA3_HUMAN]	0.37	1	1	1	1,907	213.6	4.84
Q8NEZ4	Histone-lysine N-methyltransferase MLL3 OS=Homo sapiens GN=MLL3 PE=1 SV=3 - [MLL3_HUMAN]	0.18	1	1	1	4,911	541.0	6.49
P13533	Myosin-6 OS=Homo sapiens GN=MYH6 PE=1 SV=5 - [MYH6_HUMAN]	0.41	2	1	1	1,939	223.6	5.73
Q9ULB1	Neurexin-1-alpha OS=Homo sapiens GN=NRXN1 PE=1 SV=1 - [NRX1A_HUMAN]	1.29	4	1	1	1,477	161.8	5.90
Q8NGS8	Olfactory receptor 13C5 OS=Homo sapiens GN=OR13C5 PE=2 SV=1 - [O13C5_HUMAN]	2.20	1	1	1	318	35.8	8.22
Q8NGH5	Olfactory receptor 56A1 OS=Homo sapiens GN=OR56A1 PE=2 SV=3 - [O56A1_HUMAN]	3.14	1	1	1	318	35.8	8.72
Q96PE5	Opalin OS=Homo sapiens GN=OPALIN PE=2 SV=1 - [OPALI_HUMAN]	15.60	1	1	1	141	15.7	6.18
Q6IE36	Ovostatin homolog 2 OS=Homo sapiens GN=OVOS2 PE=2 SV=2 - [OVOS2_HUMAN]	1.19	1	1	1	1,432	161.1	5.27
Q29RF7	Sister chromatid cohesion protein PDS5 homolog A OS=Homo sapiens GN=PDS5A PE=1 SV=1 - [PDS5A_HUMAN]	0.52	1	1	1	1,337	150.7	7.91
Q96FA3	Protein pellino homolog 1 OS=Homo sapiens GN=PELI1 PE=1 SV=2 - [PELI1_HUMAN]	4.78	1	1	1	418	46.3	8.03
Q92692	Poliovirus receptor-related protein 2 OS=Homo sapiens GN=PVRL2 PE=1 SV=1 - [PVRL2_HUMAN]	3.72	1	1	1	538	57.7	4.82
A1KZ92	Peroxidasin-like protein OS=Homo sapiens GN=PXDNL PE=2 SV=3 - [PXDNL_HUMAN]	1.44	1	1	1	1,463	163.6	7.43
Q12829	Ras-related protein Rab-40B OS=Homo sapiens GN=RAB40B PE=2 SV=1 - [RB40B_HUMAN]	3.96	2	1	1	278	30.9	9.61
O43865	Putative adenosylhomocysteinase 2 OS=Homo sapiens GN=AHCYL1 PE=1 SV=2 - [SAHH2_HUMAN]	3.21	1	1	1	530	58.9	6.89
Q6KCM7	Calcium-binding mitochondrial carrier protein SCaMC-2 OS=Homo sapiens GN=SLC25A25 PE=1 SV=1 - [SCMC2_HUMAN]	1.92	1	1	1	469	52.6	8.35
Q6ZSJ9	Protein shisa-6 homolog OS=Homo sapiens GN=SHISA6 PE=2 SV=2 - [SHSA6_HUMAN]	4.60	1	1	1	500	55.7	9.35
O43173	Sia-alpha-2,3-Gal-beta-1,4-GlcNAc-R:alpha 2,8-sialyltransferase OS=Homo sapiens GN=ST8SIA3 PE=2 SV=3 - [SIA8C_HUMAN]	2.37	2	1	1	380	43.9	9.52
Q96Q15	Serine/threonine-protein kinase SMG1 OS=Homo sapiens GN=SMG1 PE=1 SV=3 - [SMG1_HUMAN]	0.38	1	1	1	3,661	410.2	6.46
Q86XE0	Sorting nexin-32 OS=Homo sapiens GN=SNX32 PE=2 SV=1 - [SNX32_HUMAN]	1.74	1	1	1	403	46.4	7.09
O75558	Syntaxin-11 OS=Homo sapiens GN=STX11 PE=1 SV=1 - [STX11_HUMAN]	3.14	1	1	1	287	33.2	6.55
C9J1S8	Tripartite motif-containing protein 49-like protein 1 OS=Homo sapiens GN=TRIM49L1 PE=2 SV=1 - [T49L1_HUMAN]	1.33	1	1	1	452	52.5	8.37
Q8IZX4	Transcription initiation factor TFIID subunit 1-like OS=Homo sapiens GN=TAF1L PE=1 SV=1 - [TAF1L_HUMAN]	1.26	1	1	1	1,826	207.2	5.40
Q6P1X5	Transcription initiation factor TFIID subunit 2 OS=Homo sapiens GN=TAF2 PE=1 SV=3 - [TAF2_HUMAN]	1.17	1	1	1	1,199	136.9	8.19
O43151	Methylcytosine dioxygenase TET3 OS=Homo sapiens GN=TET3 PE=2 SV=3 - [TET3_HUMAN]	1.02	1	1	1	1,660	179.2	7.34
Q92973	Transportin-1 OS=Homo sapiens GN=TNPO1 PE=1 SV=2 - [TNPO1_HUMAN]	1.00	1	1	1	898	102.3	4.98
Q8IWV7	E3 ubiquitin-protein ligase UBR1 OS=Homo sapiens GN=UBR1 PE=1 SV=1 - [UBR1_HUMAN]	1.26	1	1	1	1,749	200.1	6.01
A8MXK9	Uncharacterized protein ENSP00000382033 OS=Homo sapiens PE=2 SV=2 - [YQ018_HUMAN]	12.65	1	1	1	166	18.0	6.52
Q9H6K5	Putative uncharacterized protein FLJ22184 OS=Homo sapiens PE=1 SV=1 - [YS027_HUMAN]	1.95	1	1	1	616	60.0	4.91
Q6AHZ1	Zinc finger protein 518A OS=Homo sapiens GN=ZNF518A PE=1 SV=2 - [Z518A_HUMAN]	1.48	1	1	1	1,483	166.7	9.28
A4D1E1	Zinc finger protein 804B OS=Homo sapiens GN=ZNF804B PE=1 SV=2 - [Z804B_HUMAN]	0.52	1	1	1	1,349	152.5	8.54
Q96IT1	Zinc finger protein 496 OS=Homo sapiens GN=ZNF496 PE=1 SV=1 - [ZN496_HUMAN]	1.53	2	1	1	587	66.9	5.69
B3KVF8	HCG27198, isoform CRA_a OS=Homo sapiens GN=hCG_27198 PE=2 SV=1 - [B3KVF8_HUMAN]	6.32	7	1	1	285	32.6	4.89
Q8N9G1	SEC16 homolog A (S. cerevisiae) OS=Homo sapiens GN=SEC16A PE=2 SV=1 - [Q8N9G1_HUMAN]	2.34	7	1	1	385	40.8	7.64
C9J8U2	Nicotinate phosphoribosyltransferase domain containing 1, isoform CRA_e OS=Homo sapiens GN=NAPRT1 PE=4 SV=1 - [C9J8U2_HUMAN]	2.04	3	1	1	490	52.1	5.33
Q4VB33	APOBEC1 protein (Fragment) OS=Homo sapiens GN=APOBEC1 PE=2 SV=1 - [Q4VB33_HUMAN]	4.48	2	1	1	223	26.8	8.63
Q8WVX6	WAPAL protein OS=Homo sapiens GN=WAPAL PE=2 SV=1 - [Q8WVX6_HUMAN]	3.48	4	1	1	402	45.5	5.08
A4D0W7	Protein Wnt OS=Homo sapiens GN=WNT16 PE=3 SV=1 - [A4D0W7_HUMAN]	2.25	2	1	1	355	39.8	8.66
B9VJ61	Toll-like receptor 5 OS=Homo sapiens GN=TLR5 PE=4 SV=1 - [B9VJ61_HUMAN]	0.70	22	1	1	858	97.7	6.74
B1ANC9	Coactivator associated arginine methyltransferase 1-like (Fragment) OS=Homo sapiens GN=CARM1L PE=4 SV=1 - [B1ANC9_HUMAN]	9.35	1	1	1	385	43.7	6.07
Q96JS4	Cerebral protein-3 OS=Homo sapiens GN=hucep-3 PE=2 SV=1 - [Q96JS4_HUMAN]	13.25	1	1	1	166	18.0	9.00
A6NDF3	Uncharacterized protein OS=Homo sapiens GN=CXorf26 PE=4 SV=2 - [A6NDF3_HUMAN]	2.59	2	1	1	232	26.6	9.36
A6NLQ3	Uncharacterized protein OS=Homo sapiens GN=BPTF PE=4 SV=3 - [A6NLQ3_HUMAN]	4.17	1	1	1	408	46.2	10.13
E9PAK3	Uncharacterized protein OS=Homo sapiens PE=4 SV=1 - [E9PAK3_HUMAN]	9.09	2	1	1	99	11.2	12.13
D6RAS8	Uncharacterized protein OS=Homo sapiens GN=IRX4 PE=4 SV=1 - [D6RAS8_HUMAN]	3.24	1	1	1	216	22.9	8.90
D6RF05	Uncharacterized protein OS=Homo sapiens GN=HARS PE=4 SV=1 - [D6RF05_HUMAN]	20.00	10	1	1	60	6.7	9.41
O14750	Reverse transcriptase (Fragment) OS=Homo sapiens GN=pol PE=4 SV=1 - [O14750_HUMAN]	18.52	1	1	1	81	9.2	9.58
B4DZN7	cDNA FLJ61501, highly similar to SID1 transmembrane family member 2 OS=Homo sapiens PE=2 SV=1 - [B4DZN7_HUMAN]	0.82	4	1	1	733	83.0	7.17
B7Z2E0	cDNA FLJ54649, highly similar to Chondroitin sulfate proteoglycan 5 OS=Homo sapiens PE=2 SV=1 - [B7Z2E0_HUMAN]	3.27	2	1	1	428	46.6	4.64
Q658M3	Putative uncharacterized protein DKFZp666H126 (Fragment) OS=Homo sapiens GN=DKFZp666H126 PE=2 SV=1 - [Q658M3_HUMAN]	5.30	6	1	1	396	45.5	7.97
B4DUR9	cDNA FLJ56675, highly similar to Activator of 90 kDa heat shock protein ATPase homolog 1 OS=Homo sapiens PE=2 SV=1 - [B4DUR9_HUMAN]	6.94	2	1	1	288	32.3	5.72
Q5JYW1	Forkhead-associated (FHA) phosphopeptide binding domain 1 OS=Homo sapiens GN=FHAD1 PE=2 SV=1 - [Q5JYW1_HUMAN]	3.59	5	1	1	251	28.6	7.74
B3KWG5	cDNA FLJ43038 fis, clone BRTHA3002955, highly similar to Receptor tyrosine-protein kinase erbB-3 (EC 2.7.10.1) OS=Homo sapiens PE=2 SV=1 - [B3KWG5_HUMAN]	4.76	6	1	1	462	50.6	5.01
E7ENR7	Uncharacterized protein OS=Homo sapiens GN=MYO18B PE=4 SV=1 - [E7ENR7_HUMAN]	0.43	4	1	1	2,080	233.6	6.73
E5RHD3	Uncharacterized protein OS=Homo sapiens GN=KHDRBS3 PE=4 SV=1 - [E5RHD3_HUMAN]	5.04	17	1	1	119	13.8	5.05
B4DJE7	cDNA FLJ52595, highly similar to Medium-chain specific acyl-CoA dehydrogenase, mitochondrial (EC 1.3.99.3) OS=Homo sapiens PE=2 SV=1 - [B4DJE7_HUMAN]	7.76	8	1	1	232	25.6	9.00
B4DF57	cDNA FLJ56569, highly similar to ERO1-like protein beta (EC 1.8.4.-) OS=Homo sapiens PE=2 SV=1 - [B4DF57_HUMAN]	19.44	3	1	1	144	16.1	8.16
Q7Z617	Archease-like protein isoform ABAC OS=Homo sapiens GN=ARCH PE=2 SV=1 - [Q7Z617_HUMAN]	19.74	2	1	1	76	8.9	8.47
Q59FX7	Microsomal NAD+-dependent retinol dehydrogenase 4 variant (Fragment) OS=Homo sapiens PE=2 SV=1 - [Q59FX7_HUMAN]	5.70	1	1	1	193	21.7	7.56
D6RBD9	Uncharacterized protein OS=Homo sapiens GN=TXNDC15 PE=4 SV=1 - [D6RBD9_HUMAN]	12.26	6	1	1	155	16.9	4.41
E9PHV5	Uncharacterized protein OS=Homo sapiens GN=SSFA2 PE=4 SV=1 - [E9PHV5_HUMAN]	0.73	2	1	1	1,237	136.1	5.20
Q6ZSU2	cDNA FLJ45207 fis, clone BRCAN2010665, highly similar to Channel associated protein of synapse-110 OS=Homo sapiens PE=2 SV=1 - [Q6ZSU2_HUMAN]	1.63	8	1	1	552	59.3	6.57
O60420	Protein tyrosine phosphatase gamma (Fragment) OS=Homo sapiens GN=PTPRG PE=2 SV=1 - [O60420_HUMAN]	4.93	3	1	1	142	15.9	5.27
Q75ME3	Putative uncharacterized protein WBSCR22 OS=Homo sapiens GN=WBSCR22 PE=2 SV=1 - [Q75ME3_HUMAN]	10.80	3	1	1	250	28.2	8.41
B4DRS4	cDNA FLJ60139, highly similar to Homo sapiens HIV TAT specific factor 1 (HTATSF1), mRNA OS=Homo sapiens PE=2 SV=1 - [B4DRS4_HUMAN]	2.36	1	1	1	721	82.0	4.42
B4E3W2	cDNA FLJ61092, highly similar to Fanconi anemia group C protein OS=Homo sapiens PE=4 SV=1 - [B4E3W2_HUMAN]	13.04	3	1	1	115	13.5	4.81
C7FDR0	MHC class I antigen (Fragment) OS=Homo sapiens GN=HLA-B PE=3 SV=1 - [C7FDR0_HUMAN]	7.73	1	1	1	181	21.0	6.35
B4DNL3	cDNA FLJ53145, highly similar to Ankycorbin (Fragment) OS=Homo sapiens PE=2 SV=1 - [B4DNL3_HUMAN]	1.52	4	1	1	659	74.5	5.10
E9PMF5	Uncharacterized protein OS=Homo sapiens GN=CHIA PE=4 SV=1 - [E9PMF5_HUMAN]	9.90	1	1	1	101	11.6	8.79
E5RHA3	Uncharacterized protein OS=Homo sapiens GN=ERICH1 PE=4 SV=1 - [E5RHA3_HUMAN]	12.17	3	1	1	345	38.3	4.86
A6NLX9	Uncharacterized protein OS=Homo sapiens GN=SPOCD1 PE=4 SV=3 - [A6NLX9_HUMAN]	3.68	2	1	1	544	55.8	5.21
B5A927	Soluble VEGFR3 variant 2 OS=Homo sapiens GN=VEGFR3 PE=2 SV=1 - [B5A927_HUMAN]	6.10	7	1	1	295	33.0	5.03
E7EMX9	Uncharacterized protein OS=Homo sapiens GN=AFF3 PE=4 SV=1 - [E7EMX9_HUMAN]	1.03	7	1	1	780	84.0	6.68
Q9BRJ0	HECTD1 protein (Fragment) OS=Homo sapiens GN=HECTD1 PE=2 SV=2 - [Q9BRJ0_HUMAN]	15.70	7	1	1	121	13.4	8.28
Q8NBW0	cDNA FLJ90715 fis, clone PLACE1008469 OS=Homo sapiens PE=2 SV=1 - [Q8NBW0_HUMAN]	3.94	5	1	1	330	36.9	8.81
Q64FX9	AKNA transcript C2 (Fragment) OS=Homo sapiens GN=AKNA PE=2 SV=1 - [Q64FX9_HUMAN]	2.29	4	1	1	612	67.3	6.42
B4DSU2	cDNA FLJ50854, highly similar to Homo sapiens solute carrier family 30 (zinc transporter), member 9 (SLC30A9), mRNA OS=Homo sapiens PE=2 SV=1 - [B4DSU2_HUMAN]	3.53	3	1	1	397	44.5	7.46
Q6V1P8	Protocadherin protein (Fragment) OS=Homo sapiens GN=PCDHJ PE=2 SV=1 - [Q6V1P8_HUMAN]	2.99	4	1	1	702	75.9	4.88
Q562X7	Actin-like protein (Fragment) OS=Homo sapiens GN=ACT PE=2 SV=1 - [Q562X7_HUMAN]	29.13	1	1	1	103	11.6	6.24
C9JIG6	Uncharacterized protein OS=Homo sapiens GN=SORBS2 PE=4 SV=1 - [C9JIG6_HUMAN]	4.57	13	1	1	372	41.5	8.98
E9PFP9	Uncharacterized protein OS=Homo sapiens GN=ATM PE=4 SV=1 - [E9PFP9_HUMAN]	0.41	2	1	1	1,708	195.9	6.77
B2RA70	cDNA, FLJ94729, highly similar to Homo sapiens v-yes-1 Yamaguchi sarcoma viral oncogene homolog 1(YES1), mRNA OS=Homo sapiens PE=2 SV=1 - [B2RA70_HUMAN]	3.68	2	1	1	543	60.8	6.74
B4DFG1	cDNA FLJ60470, highly similar to N-terminal acetyltransferase complex ARD1 subunit homolog A (EC 2.3.1.88) OS=Homo sapiens PE=4 SV=1 - [B4DFG1_HUMAN]	6.78	1	1	1	118	13.8	8.78
B4DW39	cDNA FLJ60915, highly similar to Fc receptor-like protein 5 OS=Homo sapiens PE=2 SV=1 - [B4DW39_HUMAN]	1.64	4	1	1	611	67.6	7.24
B7Z2Z6	cDNA FLJ57307, highly similar to Alkaline phytoceramidase (EC 3.5.1.-) OS=Homo sapiens PE=2 SV=1 - [B7Z2Z6_HUMAN]	8.86	2	1	1	158	18.6	8.00
B4DMS0	cDNA FLJ57852, highly similar to Cysteine-rich protein 2-binding protein OS=Homo sapiens PE=2 SV=1 - [B4DMS0_HUMAN]	2.69	3	1	1	595	67.5	8.59
Q5G1L5	N-terminally extended type 3 canonical transient receptor potential channel OS=Homo sapiens PE=2 SV=1 - [Q5G1L5_HUMAN]	0.98	1	1	1	921	105.5	6.65
A2BF27	Major histocompatibility complex class I C (Fragment) OS=Homo sapiens GN=HLA-C PE=3 SV=1 - [A2BF27_HUMAN]	5.77	4	1	1	260	29.8	8.90
E5RGP3	Uncharacterized protein OS=Homo sapiens GN=PLEKHA2 PE=4 SV=1 - [E5RGP3_HUMAN]	6.09	7	1	1	115	13.2	8.28
C9JJW1	Uncharacterized protein OS=Homo sapiens GN=FAM179A PE=4 SV=1 - [C9JJW1_HUMAN]	8.93	5	1	1	112	12.2	7.91
E7ETR0	Uncharacterized protein OS=Homo sapiens GN=RUVBL1 PE=4 SV=1 - [E7ETR0_HUMAN]	6.35	3	1	1	315	34.8	5.76
E5RG32	Glutathione peroxidase OS=Homo sapiens GN=GPX3 PE=3 SV=1 - [E5RG32_HUMAN]	10.07	2	1	1	139	15.1	5.41
Q24JQ5	ISLR2 protein OS=Homo sapiens GN=ISLR2 PE=2 SV=1 - [Q24JQ5_HUMAN]	4.49	1	1	1	334	36.2	5.16
Q59GU9	Dual oxidase 2 variant (Fragment) OS=Homo sapiens PE=2 SV=1 - [Q59GU9_HUMAN]	1.45	2	1	1	550	62.0	5.62
B4DZD5	cDNA FLJ60991, highly similar to FKBP12-rapamycin complex-associated protein OS=Homo sapiens PE=2 SV=1 - [B4DZD5_HUMAN]	0.71	3	1	1	1,404	157.6	7.77
B4E2U1	cDNA FLJ61558, highly similar to Methyl-CpG-binding domain protein 5 OS=Homo sapiens PE=2 SV=1 - [B4E2U1_HUMAN]	1.01	3	1	1	1,086	114.6	9.31
B4DMF0	Non-lysosomal glucosylceramidase OS=Homo sapiens PE=2 SV=1 - [B4DMF0_HUMAN]	2.25	3	1	1	755	85.3	5.74
B2R9D2	cDNA, FLJ94338, highly similar to Homo sapiens EGF, latrophilin and seven transmembrane domain containing 1 (ELTD1), mRNA OS=Homo sapiens PE=2 SV=1 - [B2R9D2_HUMAN]	2.81	2	1	1	606	68.6	8.02
Q6ZUW4	cDNA FLJ43276 fis, clone KIDNE2011532, moderately similar to Homo sapiens melanoma-associated chondroitin sulfate proteoglycan 4 OS=Homo sapiens PE=2 SV=1 - [Q6ZUW4_HUMAN]	11.17	1	1	1	179	21.0	5.03
B4DRV5	cDNA FLJ60840, highly similar to Nucleoporin p54 OS=Homo sapiens PE=2 SV=1 - [B4DRV5_HUMAN]	4.89	6	1	1	327	37.4	6.81
A5PLL3	MYST3 protein OS=Homo sapiens GN=MYST3 PE=2 SV=1 - [A5PLL3_HUMAN]	0.86	6	1	1	815	93.2	8.94
B4DXY4	cDNA FLJ51730, highly similar to Zinc finger protein 408 OS=Homo sapiens PE=2 SV=1 - [B4DXY4_HUMAN]	1.12	2	1	1	712	77.3	7.47
D6REC1	Uncharacterized protein OS=Homo sapiens GN=SNCAIP PE=4 SV=1 - [D6REC1_HUMAN]	31.88	3	1	1	69	7.7	4.41
B9A063	Uncharacterized protein OS=Homo sapiens GN=MYO7B PE=4 SV=1 - [B9A063_HUMAN]	1.55	6	1	1	969	110.3	8.66
Q53GZ4	Leucine rich repeat containing 5 variant (Fragment) OS=Homo sapiens PE=2 SV=1 - [Q53GZ4_HUMAN]	1.49	5	1	1	671	76.8	7.43
B4DSJ2	cDNA FLJ53526, highly similar to Homo sapiens interferon regulatory factor 2 binding protein 2 (IRF2BP2), mRNA OS=Homo sapiens PE=2 SV=1 - [B4DSJ2_HUMAN]	9.27	1	1	1	259	27.4	8.13
B7ZKR2	Sodium/hydrogen exchanger OS=Homo sapiens GN=SLC9A3 PE=2 SV=1 - [B7ZKR2_HUMAN]	3.03	3	1	1	825	91.9	7.49
B4DRS3	cDNA FLJ54645 OS=Homo sapiens PE=2 SV=1 - [B4DRS3_HUMAN]	3.66	2	1	1	383	42.1	10.10
B4DZX4	cDNA FLJ51420, highly similar to Aflatoxin B1 aldehyde reductase member 2 (EC 1.-.-.-) OS=Homo sapiens PE=2 SV=1 - [B4DZX4_HUMAN]	4.76	1	1	1	168	18.2	7.39
B3KXT6	cDNA FLJ46030 fis, clone SPLEN2028417, highly similar to Homeobox protein HLX1 OS=Homo sapiens PE=2 SV=1 - [B3KXT6_HUMAN]	2.46	2	1	1	488	50.7	8.50
B4DWE0	cDNA FLJ50260, highly similar to Cytochrome b-245 heavy chain OS=Homo sapiens PE=2 SV=1 - [B4DWE0_HUMAN]	1.86	2	1	1	538	61.3	8.43
B7Z372	cDNA FLJ51479, highly similar to Bifunctional UDP-N-acetylglucosamine2-epimerase/N- acetylmannosamine kinase OS=Homo sapiens PE=2 SV=1 - [B7Z372_HUMAN]	2.61	7	1	1	612	66.7	6.86
Q9NRB8	G-protein coupled receptor EDG-7 OS=Homo sapiens PE=2 SV=1 - [Q9NRB8_HUMAN]	2.26	1	1	1	354	40.3	9.48
C9JLA8	Uncharacterized protein OS=Homo sapiens GN=ARHGAP4 PE=4 SV=1 - [C9JLA8_HUMAN]	5.75	8	1	1	174	20.2	7.52
B4DUY0	cDNA FLJ60352, highly similar to Glutaryl-CoA dehydrogenase, mitochondrial (EC 1.3.99.7) OS=Homo sapiens PE=2 SV=1 - [B4DUY0_HUMAN]	5.84	4	1	1	274	29.5	8.92
C9JNV2	Uncharacterized protein OS=Homo sapiens GN=BUD31 PE=4 SV=1 - [C9JNV2_HUMAN]	9.57	1	1	1	115	13.6	8.85
O43180	Spinocerebellar ataxia 7 (Fragment) OS=Homo sapiens GN=SCA7 PE=2 SV=1 - [O43180_HUMAN]	13.95	2	1	1	129	14.1	9.70
Q36734	NADH dehydrogenase subunit 2 (Fragment) OS=Homo sapiens GN=clone 39-1 PE=2 SV=1 - [Q36734_HUMAN]	29.85	1	1	1	67	7.9	8.57
Q7Z4E6	MSTP094 OS=Homo sapiens GN=MST094 PE=2 SV=1 - [Q7Z4E6_HUMAN]	5.56	4	1	1	108	12.2	6.52
Q59HA9	Glutamate [NMDA] receptor subunit epsilon 2 variant (Fragment) OS=Homo sapiens PE=2 SV=1 - [Q59HA9_HUMAN]	1.50	2	1	1	1,136	127.4	8.19
B4E3P6	cDNA FLJ61089, highly similar to Zinc finger protein 646 OS=Homo sapiens PE=2 SV=1 - [B4E3P6_HUMAN]	1.15	2	1	1	1,305	142.9	6.70
B7Z7S9	cDNA FLJ61724, highly similar to Shugoshin-like 2 OS=Homo sapiens PE=2 SV=1 - [B7Z7S9_HUMAN]	1.27	2	1	1	1,260	143.9	7.59
Q9Y408	Putative uncharacterized protein DKFZp566D133 (Fragment) OS=Homo sapiens GN=GARNL2P PE=2 SV=1 - [Q9Y408_HUMAN]	5.25	4	1	1	381	43.9	6.73
E7EP04	Uncharacterized protein OS=Homo sapiens GN=DNAJC10 PE=4 SV=1 - [E7EP04_HUMAN]	3.64	5	1	1	275	31.4	7.05
E9PRK2	Uncharacterized protein OS=Homo sapiens GN=NARS2 PE=4 SV=1 - [E9PRK2_HUMAN]	3.73	3	1	1	241	27.2	9.03
E9PK12	Uncharacterized protein OS=Homo sapiens GN=FUZ PE=4 SV=1 - [E9PK12_HUMAN]	4.35	2	1	1	368	40.6	5.95
E9PK39	Uncharacterized protein OS=Homo sapiens GN=LRRK1 PE=4 SV=1 - [E9PK39_HUMAN]	2.46	3	1	1	650	72.2	7.36
Q7Z4W6	Preprotachykinin B OS=Homo sapiens PE=4 SV=1 - [Q7Z4W6_HUMAN]	20.37	2	1	1	108	12.1	9.20
Q9H7K8	FLJ00064 protein (Fragment) OS=Homo sapiens GN=FLJ00064 PE=2 SV=1 - [Q9H7K8_HUMAN]	3.99	1	1	1	276	30.4	5.66
A8K8F0	cDNA FLJ76436 OS=Homo sapiens PE=2 SV=1 - [A8K8F0_HUMAN]	6.71	1	1	1	298	33.2	5.60
B3KSY6	cDNA FLJ37308 fis, clone BRAMY2016386, highly similar to Paraplegin (EC 3.4.24.-) OS=Homo sapiens PE=2 SV=1 - [B3KSY6_HUMAN]	4.72	1	1	1	212	23.7	5.33
B6E614	HBV XAg-transactivated protein 11 splice variant 1 OS=Homo sapiens GN=XTP11 PE=2 SV=1 - [B6E614_HUMAN]	3.43	3	1	1	437	49.2	5.22
Q53SE4	Putative uncharacterized protein DGKD (Fragment) OS=Homo sapiens GN=DGKD PE=2 SV=1 - [Q53SE4_HUMAN]	1.37	2	1	1	1,098	121.8	7.21
B4DXU6	cDNA FLJ52174, highly similar to Homo sapiens ASF1 anti-silencing function 1 homolog B (S. cerevisiae) (ASF1B), mRNA OS=Homo sapiens PE=2 SV=1 - [B4DXU6_HUMAN]	10.81	2	1	1	185	20.4	4.55
B4E061	cDNA FLJ51170, highly similar to DNA topoisomerase I, mitochondrial (EC 5.99.1.2) OS=Homo sapiens PE=2 SV=1 - [B4E061_HUMAN]	6.92	2	1	1	477	53.4	8.78
Q8NB64	cDNA FLJ34177 fis, clone FCBBF3016451, highly similar to RETINAL-CADHERIN OS=Homo sapiens PE=2 SV=1 - [Q8NB64_HUMAN]	1.09	4	1	1	824	90.2	4.91
B5MC89	Uncharacterized protein OS=Homo sapiens GN=THADA PE=4 SV=1 - [B5MC89_HUMAN]	1.15	10	1	1	870	98.4	6.87
C9JSN6	Microtubule-associated protein OS=Homo sapiens PE=4 SV=1 - [C9JSN6_HUMAN]	1.06	2	1	1	758	78.9	6.71
E7EV12	Uncharacterized protein OS=Homo sapiens GN=IPO5 PE=4 SV=1 - [E7EV12_HUMAN]	11.27	1	1	1	142	15.7	4.82
Q5T0C9	SCL/TAL1 interrupting locus (Fragment) OS=Homo sapiens GN=STIL PE=2 SV=1 - [Q5T0C9_HUMAN]	2.86	1	1	1	280	31.9	6.38
B3KTW8	cDNA FLJ38884 fis, clone MESAN2017152, highly similar to Homo sapiens PH domain-containing protein (pp9099), mRNA OS=Homo sapiens PE=2 SV=1 - [B3KTW8_HUMAN]	3.59	3	1	1	223	25.0	8.63
A8K8H7	cDNA FLJ77598 OS=Homo sapiens PE=2 SV=1 - [A8K8H7_HUMAN]	1.25	2	1	1	559	61.8	9.22
B4DIL4	cDNA FLJ50166, highly similar to Dedicator of cytokinesis protein 6 OS=Homo sapiens PE=2 SV=1 - [B4DIL4_HUMAN]	1.23	4	1	1	1,386	154.4	6.87
A8K8X0	cDNA FLJ75187, highly similar to Homo sapiens nap1 P120 OS=Homo sapiens PE=2 SV=1 - [A8K8X0_HUMAN]	4.12	1	1	1	972	112.1	6.70
B3KXV2	cDNA FLJ46110 fis, clone TESTI2033905, highly similar to Homo sapiens MYCBP associated protein (MYCBPAP), mRNA OS=Homo sapiens PE=2 SV=1 - [B3KXV2_HUMAN]	1.94	4	1	1	774	88.8	6.87
B7ZLB7	C5orf44 protein (Fragment) OS=Homo sapiens GN=C5orf44 PE=2 SV=1 - [B7ZLB7_HUMAN]	5.50	3	1	1	400	44.6	5.73
B5MCM0	Uncharacterized protein OS=Homo sapiens GN=TEAD3 PE=4 SV=1 - [B5MCM0_HUMAN]	5.87	8	1	1	375	41.9	9.33
Q8N274	cDNA FLJ33834 fis, clone CTONG2004264, moderately similar to NEUROBLAST DIFFERENTIATION ASSOCIATED PROTEIN AHNAK OS=Homo sapiens PE=2 SV=1 - [Q8N274_HUMAN]	1.14	2	1	1	791	86.0	6.05
Q3MS94	Dominant-negative kinase-deficient Brutons tyrosine kinase isoform 4 (Fragment) OS=Homo sapiens GN=BTK kinase deficient isoform 4 PE=2 SV=1 - [Q3MS94_HUMAN]	10.66	4	1	1	197	22.8	7.43
A8K0T4	cDNA FLJ78233 OS=Homo sapiens PE=4 SV=1 - [A8K0T4_HUMAN]	24.72	2	1	1	89	9.6	4.70
O00179	9-cis-retinol specific dehydrogenase OS=Homo sapiens PE=2 SV=1 - [O00179_HUMAN]	2.52	2	1	1	318	34.8	8.84
Q59G54	Bromodomain adjacent to zinc finger domain, 1A isoform b variant (Fragment) OS=Homo sapiens PE=2 SV=1 - [Q59G54_HUMAN]	1.43	3	1	1	1,188	136.5	5.97
Q86YI3	PSD protein (Fragment) OS=Homo sapiens GN=PSD PE=2 SV=2 - [Q86YI3_HUMAN]	1.19	2	1	1	927	99.7	6.19
C9WES4	MHC class I antigen (Fragment) OS=Homo sapiens GN=HLA-C PE=3 SV=1 - [C9WES4_HUMAN]	7.73	1	1	1	181	21.3	6.60
E9PPC5	Uncharacterized protein OS=Homo sapiens GN=RCOR3 PE=4 SV=1 - [E9PPC5_HUMAN]	40.91	8	1	1	44	4.9	5.87
E5RFR2	Uncharacterized protein OS=Homo sapiens GN=ADCY8 PE=3 SV=1 - [E5RFR2_HUMAN]	3.17	4	1	1	252	28.9	6.52
B0QYL3	Protein interacting with PRKCA 1 (Fragment) OS=Homo sapiens GN=PICK1 PE=4 SV=1 - [B0QYL3_HUMAN]	7.42	5	1	1	229	25.1	7.44
Q96I77	TMC6 protein OS=Homo sapiens GN=TMC6 PE=2 SV=1 - [Q96I77_HUMAN]	3.32	4	1	1	211	23.7	7.93
B4DEW5	cDNA FLJ54049, highly similar to Multimerin-2 OS=Homo sapiens PE=2 SV=1 - [B4DEW5_HUMAN]	2.48	4	1	1	727	80.0	5.74
B3KT81	cDNA FLJ37836 fis, clone BRSSN2010587, weakly similar to Homo sapiens MDN1, midasin homolog (yeast) (MDN1), mRNA OS=Homo sapiens PE=2 SV=1 - [B3KT81_HUMAN]	0.88	3	1	1	1,018	114.7	7.65
B2R6A3	cDNA, FLJ92860, highly similar to Homo sapiens solute carrier family 9 (sodium/hydrogen exchanger), member 3 regulator 1 (SLC9A3R1), mRNA OS=Homo sapiens PE=2 SV=1 - [B2R6A3_HUMAN]	2.23	2	1	1	358	38.9	5.77
B4DV35	cDNA FLJ54280, moderately similar to Deoxyribonuclease-1 (EC 3.1.21.1) OS=Homo sapiens PE=2 SV=1 - [B4DV35_HUMAN]	4.81	3	1	1	187	19.8	7.39
B7WP74	Uncharacterized protein OS=Homo sapiens GN=CWC22 PE=4 SV=1 - [B7WP74_HUMAN]	1.88	3	1	1	745	85.5	5.71
D6RDI8	Uncharacterized protein OS=Homo sapiens GN=ACAD11 PE=4 SV=1 - [D6RDI8_HUMAN]	1.89	2	1	1	476	53.7	6.95
Q4GUF2	ATP synthase protein 8 OS=Homo sapiens GN=ATPase 8 PE=3 SV=1 - [Q4GUF2_HUMAN]	23.53	1	1	1	68	8.0	9.91
C9JRH1	Uncharacterized protein OS=Homo sapiens GN=DHRSX PE=4 SV=1 - [C9JRH1_HUMAN]	14.75	5	1	1	122	13.7	9.28
Q59FW1	Metastasis associated protein variant (Fragment) OS=Homo sapiens PE=2 SV=1 - [Q59FW1_HUMAN]	1.96	4	1	1	511	57.3	9.89
B4DMD2	cDNA FLJ54025, highly similar to Coiled-coil domain-containing protein 45 OS=Homo sapiens PE=2 SV=1 - [B4DMD2_HUMAN]	2.74	5	1	1	657	76.6	9.39
B4DS06	cDNA FLJ59965, highly similar to Metallophosphoesterase domain-containing protein 1 OS=Homo sapiens PE=2 SV=1 - [B4DS06_HUMAN]	3.57	1	1	1	168	19.2	7.40
B4DH24	cDNA FLJ58843, highly similar to Rab GDP dissociation inhibitor alpha OS=Homo sapiens PE=2 SV=1 - [B4DH24_HUMAN]	4.59	4	1	1	196	22.4	5.20
B4E2Y2	cDNA FLJ50370, highly similar to Ubiquitin conjugation factor E4 B OS=Homo sapiens PE=2 SV=1 - [B4E2Y2_HUMAN]	1.80	3	1	1	1,057	121.3	6.14
B4DMA0	cDNA FLJ56008, highly similar to Aldehyde dehydrogenase family 7 member A1 (EC 1.2.1.3) OS=Homo sapiens PE=2 SV=1 - [B4DMA0_HUMAN]	2.19	3	1	1	502	54.0	7.99
C9J871	Uncharacterized protein OS=Homo sapiens GN=ZNF808 PE=4 SV=1 - [C9J871_HUMAN]	12.05	5	1	1	83	9.6	9.17
E9PPZ7	Uncharacterized protein OS=Homo sapiens GN=SSRP1 PE=4 SV=1 - [E9PPZ7_HUMAN]	13.64	4	1	1	154	17.6	5.33
Q4F969	Leukocyte specific transcript 1 OS=Homo sapiens PE=4 SV=1 - [Q4F969_HUMAN]	36.67	1	1	1	90	10.1	5.41
Q59H36	Crumbs homolog 1 isoform II variant (Fragment) OS=Homo sapiens PE=2 SV=1 - [Q59H36_HUMAN]	4.08	1	1	1	441	48.0	4.75
Q96AZ4	Putative uncharacterized protein (Fragment) OS=Homo sapiens PE=2 SV=1 - [Q96AZ4_HUMAN]	15.65	5	1	1	147	15.4	9.89
A8K3R3	cDNA FLJ75284, highly similar to Homo sapiens RAB guanine nucleotide exchange factor (GEF) 1 (RABGEF1), mRNA OS=Homo sapiens PE=2 SV=1 - [A8K3R3_HUMAN]	1.83	5	1	1	491	56.8	7.27
B4DQP2	cDNA FLJ59736, highly similar to Protein-arginine deiminase type-1 (EC 3.5.3.15) OS=Homo sapiens PE=2 SV=1 - [B4DQP2_HUMAN]	14.18	4	1	1	134	15.7	6.99
B4DDU7	cDNA FLJ56797, highly similar to Aladin OS=Homo sapiens PE=2 SV=1 - [B4DDU7_HUMAN]	3.12	1	1	1	513	55.8	7.49
B7Z8T7	cDNA FLJ51752, highly similar to Neuroendocrine convertase 1 (EC 3.4.21.93) OS=Homo sapiens PE=2 SV=1 - [B7Z8T7_HUMAN]	1.42	5	1	1	706	78.9	6.13
Q13139	mRNA clone with similarity to L-glycerol-3-phosphate:NAD oxidoreductase and albumin gene sequences OS=Homo sapiens PE=2 SV=1 - [Q13139_HUMAN]	6.04	1	1	1	331	35.7	9.55
Q59FN1	Integrin beta (Fragment) OS=Homo sapiens PE=2 SV=1 - [Q59FN1_HUMAN]	6.02	4	1	1	166	18.8	6.68
B7Z703	cDNA FLJ59769, highly similar to Homo sapiens pleckstrin homology domain containing, family G (with RhoGef domain) member 5, transcript variant 1, mRNA OS=Homo sapiens PE=2 SV=1 - [B7Z703_HUMAN]	2.72	5	1	1	736	81.3	6.01
Q5JYA3	Methylenetetrahydrofolate dehydrogenase (NADP+ dependent) 1-like (Fragment) OS=Homo sapiens GN=MTHFD1L PE=2 SV=1 - [Q5JYA3_HUMAN]	5.26	5	1	1	114	12.4	4.77
C9JP57	Uncharacterized protein OS=Homo sapiens GN=EFHC2 PE=4 SV=1 - [C9JP57_HUMAN]	2.48	5	1	1	727	85.0	7.01
D6RGY5	Uncharacterized protein OS=Homo sapiens GN=TCP11 PE=4 SV=1 - [D6RGY5_HUMAN]	3.17	14	1	1	221	24.8	5.57
E7ESQ8	Uncharacterized protein OS=Homo sapiens GN=CERCAM PE=4 SV=1 - [E7ESQ8_HUMAN]	1.82	1	1	1	548	62.1	6.49
Q8WZ36	Putative uncharacterized protein OS=Homo sapiens PE=1 SV=1 - [Q8WZ36_HUMAN]	5.66	1	1	1	106	11.9	8.68
Q4W5I3	Putative uncharacterized protein ABCG2 (Fragment) OS=Homo sapiens GN=ABCG2 PE=2 SV=1 - [Q4W5I3_HUMAN]	1.32	2	1	1	607	66.8	8.66
Q5MY58	UURF2 ubiquitin ligase (Fragment) OS=Homo sapiens PE=2 SV=1 - [Q5MY58_HUMAN]	2.55	4	1	1	275	30.2	7.34
Q9NSM6	Putative uncharacterized protein DKFZp761P19121 (Fragment) OS=Homo sapiens GN=DKFZp761P19121 PE=2 SV=1 - [Q9NSM6_HUMAN]	14.12	4	1	1	177	20.5	6.64
B2R6D7	cDNA, FLJ92904, highly similar to Homo sapiens casein kinase 2, alpha 1 polypeptide (CSNK2A1), mRNA OS=Homo sapiens PE=2 SV=1 - [B2R6D7_HUMAN]	4.86	1	1	1	391	45.1	7.75
B4DEK5	cDNA FLJ54596, highly similar to Proactivator polypeptide OS=Homo sapiens PE=2 SV=1 - [B4DEK5_HUMAN]	3.32	8	1	1	452	50.6	4.97
B4DSW6	cDNA FLJ58422, highly similar to HIRA protein OS=Homo sapiens PE=2 SV=1 - [B4DSW6_HUMAN]	1.99	1	1	1	806	88.1	8.31
B3KWG8	cDNA FLJ43069 fis, clone BRTHA3009037, highly similar to Regulator of G-protein signaling 3 OS=Homo sapiens PE=2 SV=1 - [B3KWG8_HUMAN]	0.64	2	1	1	1,088	120.1	6.30
Q6WG77	Antigen MLAA-16 (Fragment) OS=Homo sapiens PE=2 SV=1 - [Q6WG77_HUMAN]	7.37	1	1	1	285	31.3	8.18
E9PJ73	Uncharacterized protein OS=Homo sapiens GN=BATF2 PE=4 SV=1 - [E9PJ73_HUMAN]	23.23	3	1	1	155	17.1	7.43
B4DYF8	cDNA FLJ58479, highly similar to Leucine-rich repeats and calponin homologydomain-containing protein 4 OS=Homo sapiens PE=2 SV=1 - [B4DYF8_HUMAN]	4.68	4	1	1	363	39.2	9.09
C6K4M2	MHC class I antigen (Fragment) OS=Homo sapiens GN=HLA-C PE=3 SV=1 - [C6K4M2_HUMAN]	6.08	1	1	1	181	20.7	5.73
Q92974	Rho guanine nucleotide exchange factor 2 OS=Homo sapiens GN=ARHGEF2 PE=1 SV=4 - [ARHG2_HUMAN]	1.52	3	1	1	986	111.5	7.27
P36575	Arrestin-C OS=Homo sapiens GN=ARR3 PE=1 SV=2 - [ARRC_HUMAN]	4.64	1	1	1	388	42.8	5.77
Q96QE3	ATPase family AAA domain-containing protein 5 OS=Homo sapiens GN=ATAD5 PE=1 SV=4 - [ATAD5_HUMAN]	0.43	1	1	1	1,844	207.4	9.19
O00512	B-cell CLL/lymphoma 9 protein OS=Homo sapiens GN=BCL9 PE=1 SV=4 - [BCL9_HUMAN]	1.54	2	1	1	1,426	149.2	8.91
Q6ZMU1	Putative protein C3P1 OS=Homo sapiens GN=C3P1 PE=5 SV=3 - [C3P1_HUMAN]	3.86	1	1	1	363	40.2	6.79
A6NFE2	Uncharacterized protein C12orf70 OS=Homo sapiens GN=C12orf70 PE=2 SV=2 - [CL070_HUMAN]	4.96	1	1	1	343	39.5	4.86
Q2UY09	Collagen alpha-1(XXVIII) chain OS=Homo sapiens GN=COL28A1 PE=2 SV=2 - [COSA1_HUMAN]	1.51	1	1	1	1,125	116.6	6.40
P86434	Uncharacterized protein C22orf45 OS=Homo sapiens GN=C22orf45 PE=2 SV=1 - [CV045_HUMAN]	11.32	1	1	1	159	17.2	6.65
Q7L576	Cytoplasmic FMR1-interacting protein 1 OS=Homo sapiens GN=CYFIP1 PE=1 SV=1 - [CYFP1_HUMAN]	1.04	3	1	1	1,253	145.1	6.90
O60477	Deleted in bladder cancer protein 1 OS=Homo sapiens GN=DBC1 PE=2 SV=2 - [DBC1_HUMAN]	4.07	1	1	1	761	88.7	8.91
O95886	Disks large-associated protein 3 OS=Homo sapiens GN=DLGAP3 PE=1 SV=3 - [DLGP3_HUMAN]	1.12	1	1	1	979	106.0	8.76
O75937	DnaJ homolog subfamily C member 8 OS=Homo sapiens GN=DNAJC8 PE=1 SV=2 - [DNJC8_HUMAN]	3.95	1	1	1	253	29.8	9.06
Q8TE73	Dynein heavy chain 5, axonemal OS=Homo sapiens GN=DNAH5 PE=1 SV=3 - [DYH5_HUMAN]	0.15	1	1	1	4,624	528.7	6.10
Q86XX4	Extracellular matrix protein FRAS1 OS=Homo sapiens GN=FRAS1 PE=2 SV=1 - [FRAS1_HUMAN]	0.22	2	1	1	4,007	442.6	5.59
Q8N442	Translation factor GUF1, mitochondrial OS=Homo sapiens GN=GUF1 PE=1 SV=1 - [GUF1_HUMAN]	3.44	1	1	1	669	74.3	8.59
Q86W26	NACHT, LRR and PYD domains-containing protein 10 OS=Homo sapiens GN=NLRP10 PE=1 SV=1 - [NAL10_HUMAN]	1.37	1	1	1	655	75.0	7.20
Q9UHQ9	NADH-cytochrome b5 reductase 1 OS=Homo sapiens GN=CYB5R1 PE=1 SV=1 - [NB5R1_HUMAN]	5.90	1	1	1	305	34.1	9.38
Q8NGE3	Olfactory receptor 10P1 OS=Homo sapiens GN=OR10P1 PE=2 SV=1 - [O10P1_HUMAN]	9.58	2	1	1	313	34.7	9.70
Q96HA1	Nuclear envelope pore membrane protein POM 121 OS=Homo sapiens GN=POM121 PE=1 SV=2 - [P121A_HUMAN]	0.72	1	1	1	1,249	127.6	10.56
O60346	PH domain leucine-rich repeat-containing protein phosphatase 1 OS=Homo sapiens GN=PHLPP1 PE=1 SV=3 - [PHLP1_HUMAN]	0.87	1	1	1	1,717	184.6	6.28
Q99638	Cell cycle checkpoint control protein RAD9A OS=Homo sapiens GN=RAD9A PE=1 SV=1 - [RAD9A_HUMAN]	7.42	1	1	1	391	42.5	5.66
Q8TCX5	Rhophilin-1 OS=Homo sapiens GN=RHPN1 PE=2 SV=1 - [RHPN1_HUMAN]	1.44	1	1	1	695	76.2	7.77
Q01973	Tyrosine-protein kinase transmembrane receptor ROR1 OS=Homo sapiens GN=ROR1 PE=2 SV=2 - [ROR1_HUMAN]	1.60	2	1	1	937	104.2	7.17
P81133	Single-minded homolog 1 OS=Homo sapiens GN=SIM1 PE=2 SV=2 - [SIM1_HUMAN]	2.48	1	1	1	766	85.5	7.43
O00241	Signal-regulatory protein beta-1 OS=Homo sapiens GN=SIRPB1 PE=1 SV=5 - [SIRB1_HUMAN]	7.54	1	1	1	398	43.2	6.52
Q03403	Trefoil factor 2 OS=Homo sapiens GN=TFF2 PE=1 SV=2 - [TFF2_HUMAN]	13.18	1	1	1	129	14.3	5.81
Q6ZT12	E3 ubiquitin-protein ligase UBR3 OS=Homo sapiens GN=UBR3 PE=2 SV=2 - [UBR3_HUMAN]	0.79	2	1	1	1,888	212.3	6.10
Q5T4S7	E3 ubiquitin-protein ligase UBR4 OS=Homo sapiens GN=UBR4 PE=1 SV=1 - [UBR4_HUMAN]	0.35	1	1	1	5,183	573.5	6.04
Q8N0Z9	V-set and immunoglobulin domain-containing protein 10 OS=Homo sapiens GN=VSIG10 PE=2 SV=1 - [VSI10_HUMAN]	1.67	1	1	1	540	59.2	4.69
A8MXY4	Zinc finger protein 99 OS=Homo sapiens GN=ZNF99 PE=2 SV=2 - [ZNF99_HUMAN]	0.68	1	1	1	1,036	120.0	9.31
O43149	Zinc finger ZZ-type and EF-hand domain-containing protein 1 OS=Homo sapiens GN=ZZEF1 PE=1 SV=6 - [ZZEF1_HUMAN]	0.24	1	1	1	2,961	330.9	5.95
B4DRI5	cDNA FLJ54307, highly similar to Zinc finger protein 282 OS=Homo sapiens PE=2 SV=1 - [B4DRI5_HUMAN]	4.32	4	1	1	463	51.9	4.70
A9Z1Z8	Glutamate receptor, ionotropic, kainate 3 OS=Homo sapiens GN=GRIK3 PE=2 SV=1 - [A9Z1Z8_HUMAN]	1.95	3	1	1	872	98.7	7.17
Q0VFX3	FLJ44955 protein OS=Homo sapiens GN=FLJ44955 PE=2 SV=1 - [Q0VFX3_HUMAN]	3.48	1	1	1	201	23.3	9.38
E9PBK4	Uncharacterized protein OS=Homo sapiens GN=C14orf102 PE=4 SV=1 - [E9PBK4_HUMAN]	1.29	3	1	1	933	106.8	6.21
A6NCI0	Mitochondrial ribosomal protein L42, isoform CRA_e OS=Homo sapiens GN=MRPL42 PE=4 SV=1 - [A6NCI0_HUMAN]	10.56	1	1	1	142	16.6	6.98
Q5T097	Utrophin OS=Homo sapiens GN=UTRN PE=2 SV=1 - [Q5T097_HUMAN]	1.92	2	1	1	988	113.3	6.96
Q5SWK9	Triadin OS=Homo sapiens GN=TRDN PE=2 SV=1 - [Q5SWK9_HUMAN]	1.53	2	1	1	721	80.7	9.38
D3DNV8	Leprecan-like 1, isoform CRA_b OS=Homo sapiens GN=LEPREL1 PE=4 SV=1 - [D3DNV8_HUMAN]	4.17	3	1	1	527	60.3	5.07
D3DX14	UDP-N-acetyl-alpha-D-galactosamine:polypeptide N-acetylgalactosaminyltransferase-like 5, isoform CRA_d OS=Homo sapiens GN=GALNTL5 PE=4 SV=1 - [D3DX14_HUMAN]	6.15	1	1	1	130	14.9	9.42
Q5JU42	Primase, polypeptide 2A, 58kDa (Fragment) OS=Homo sapiens GN=PRIM2A PE=2 SV=1 - [Q5JU42_HUMAN]	3.82	3	1	1	340	39.8	8.43
Q14663	H-2K binding factor-2 OS=Homo sapiens GN=KBF2 PE=2 SV=1 - [Q14663_HUMAN]	5.71	1	1	1	420	46.1	6.39
A0N4V7	HCG2039797 (Fragment) OS=Homo sapiens GN=Tcr-alpha PE=4 SV=1 - [A0N4V7_HUMAN]	38.10	1	1	1	21	2.2	9.74
Q8TDU0	HCG2044627 OS=Homo sapiens GN=GPCR PE=4 SV=1 - [Q8TDU0_HUMAN]	4.24	1	1	1	401	43.1	5.20
B4DVH1	cDNA FLJ54927, highly similar to VIP36-like protein OS=Homo sapiens PE=2 SV=1 - [B4DVH1_HUMAN]	3.94	6	1	1	203	23.4	7.46
D6RF86	Uncharacterized protein OS=Homo sapiens GN=CDH6 PE=4 SV=1 - [D6RF86_HUMAN]	3.13	2	1	1	608	67.4	4.88
E9PJ07	Uncharacterized protein OS=Homo sapiens GN=C10orf137 PE=4 SV=1 - [E9PJ07_HUMAN]	4.37	2	1	1	343	37.6	6.13
Q6P3S4	CEBPA protein OS=Homo sapiens PE=2 SV=1 - [Q6P3S4_HUMAN]	4.26	2	1	1	141	15.2	10.58
B1AHQ3	Tuftelin interacting protein 11 (Fragment) OS=Homo sapiens GN=TFIP11 PE=4 SV=1 - [B1AHQ3_HUMAN]	3.16	10	1	1	190	21.3	8.05
B7Z563	cDNA FLJ53015, highly similar to Homo sapiens GTP binding protein 3 (GTPBP3), transcript variant IV, mRNA OS=Homo sapiens PE=2 SV=1 - [B7Z563_HUMAN]	4.64	3	1	1	151	15.5	11.37
Q2VPK0	ATG16L2 protein (Fragment) OS=Homo sapiens GN=ATG16L2 PE=2 SV=1 - [Q2VPK0_HUMAN]	14.41	3	1	1	111	12.6	6.32
D6RB32	Uncharacterized protein OS=Homo sapiens GN=F11 PE=4 SV=1 - [D6RB32_HUMAN]	4.32	3	1	1	162	18.4	6.49
B1AKS7	Zinc finger protein 25 OS=Homo sapiens GN=ZNF25 PE=4 SV=1 - [B1AKS7_HUMAN]	2.14	2	1	1	420	49.3	9.01
Q6ZND8	cDNA FLJ16191 fis, clone BRTHA2013610, moderately similar to Arabidopsis thaliana deoxyguanosine kinase -related (At1g72040) mRNA OS=Homo sapiens PE=2 SV=1 - [Q6ZND8_HUMAN]	2.93	1	1	1	512	57.5	8.46
B3KWT9	cDNA FLJ43819 fis, clone TESTI4001925, highly similar to Homo sapiens spermatogenesis associated 20 (SPATA20), mRNA OS=Homo sapiens PE=2 SV=1 - [B3KWT9_HUMAN]	2.21	5	1	1	453	50.2	6.60
B4DWJ5	cDNA FLJ58292, moderately similar to Homo sapiens zinc finger protein 134 (clone pHZ-15) (ZNF134), mRNA OS=Homo sapiens PE=2 SV=1 - [B4DWJ5_HUMAN]	3.98	8	1	1	377	43.1	8.62
B2RC94	cDNA, FLJ95922 OS=Homo sapiens PE=2 SV=1 - [B2RC94_HUMAN]	7.05	2	1	1	227	25.5	8.66
Q5EDC5	Cytochrome p4502C9 (Fragment) OS=Homo sapiens GN=CYP2C9 PE=2 SV=1 - [Q5EDC5_HUMAN]	9.68	15	1	1	62	7.3	8.54
B4DSE6	cDNA FLJ57580, highly similar to Zinc finger protein 485 OS=Homo sapiens PE=2 SV=1 - [B4DSE6_HUMAN]	2.86	3	1	1	350	40.0	9.74
E9PH38	Uncharacterized protein OS=Homo sapiens GN=PPP2R1A PE=4 SV=1 - [E9PH38_HUMAN]	3.34	2	1	1	509	56.8	5.69
C9J0Q4	Uncharacterized protein OS=Homo sapiens GN=RAD18 PE=4 SV=1 - [C9J0Q4_HUMAN]	7.61	3	1	1	92	10.6	7.58
Q6XYC0	LP2209 OS=Homo sapiens PE=4 SV=1 - [Q6XYC0_HUMAN]	14.29	1	1	1	70	7.8	9.99
B4DPQ1	cDNA FLJ55741, moderately similar to Bile acyl-CoA synthetase (EC 6.2.1.7) OS=Homo sapiens PE=2 SV=1 - [B4DPQ1_HUMAN]	2.97	2	1	1	606	66.8	7.87
E7EMA6	Uncharacterized protein OS=Homo sapiens GN=ZYG11B PE=4 SV=1 - [E7EMA6_HUMAN]	2.71	4	1	1	665	75.0	7.03
E9PKK1	Uncharacterized protein OS=Homo sapiens GN=RPS6KB2 PE=4 SV=1 - [E9PKK1_HUMAN]	5.63	1	1	1	160	17.4	7.88
E9PGC6	Uncharacterized protein OS=Homo sapiens GN=PRCC PE=4 SV=1 - [E9PGC6_HUMAN]	4.28	1	1	1	467	50.3	7.55
E7ES48	Uncharacterized protein OS=Homo sapiens GN=P2RX6 PE=4 SV=1 - [E7ES48_HUMAN]	32.50	3	1	1	80	8.8	8.73
D6RCL0	Uncharacterized protein OS=Homo sapiens GN=DCP2 PE=4 SV=1 - [D6RCL0_HUMAN]	8.11	2	1	1	74	8.5	9.01
O43831	NFX.1 (Fragment) OS=Homo sapiens PE=2 SV=1 - [O43831_HUMAN]	15.13	3	1	1	152	16.5	7.62
A4UCR9	Mitochondrial 28S ribosomal protein S34 OS=Homo sapiens PE=2 SV=1 - [A4UCR9_HUMAN]	3.98	3	1	1	176	20.8	10.93
C9JY71	Uncharacterized protein OS=Homo sapiens GN=CC2D2A PE=4 SV=1 - [C9JY71_HUMAN]	0.58	2	1	1	1,561	179.4	6.83
E7EQQ3	Uncharacterized protein OS=Homo sapiens GN=ZNF181 PE=4 SV=1 - [E7EQQ3_HUMAN]	2.44	1	1	1	615	70.5	8.66
C9JS59	Uncharacterized protein OS=Homo sapiens GN=RNF123 PE=4 SV=1 - [C9JS59_HUMAN]	2.64	4	1	1	721	81.5	7.18
Q9P0H6	AD-012 protein OS=Homo sapiens PE=2 SV=1 - [Q9P0H6_HUMAN]	3.60	1	1	1	583	60.7	7.42
B4DRD7	cDNA FLJ54752, highly similar to Poly(rC)-binding protein 2 OS=Homo sapiens PE=2 SV=1 - [B4DRD7_HUMAN]	2.84	10	1	1	282	29.7	7.71
E9PQL1	Uncharacterized protein OS=Homo sapiens GN=FAM76A PE=4 SV=1 - [E9PQL1_HUMAN]	5.56	2	1	1	288	33.1	8.82
B7Z4C0	cDNA FLJ51894, highly similar to Glutamate receptor 3 OS=Homo sapiens PE=2 SV=1 - [B7Z4C0_HUMAN]	2.72	5	1	1	662	76.2	6.80
E7BJU4	Heme oxygenase 1 (Fragment) OS=Homo sapiens GN=HO1 PE=2 SV=1 - [E7BJU4_HUMAN]	14.05	1	1	1	121	14.3	6.96
C1JFL7	Polysystin 1 (Fragment) OS=Homo sapiens GN=PKD1 PE=4 SV=1 - [C1JFL7_HUMAN]	26.92	1	1	1	52	5.4	4.60
Q59G96	Dynamin 2 isoform 4 variant (Fragment) OS=Homo sapiens PE=2 SV=1 - [Q59G96_HUMAN]	3.70	6	1	1	487	55.1	8.65
E5RHN4	Uncharacterized protein OS=Homo sapiens GN=CYP26B1 PE=4 SV=1 - [E5RHN4_HUMAN]	9.88	4	1	1	172	19.5	10.49
C9JR29	Uncharacterized protein OS=Homo sapiens GN=CSMD3 PE=4 SV=1 - [C9JR29_HUMAN]	0.46	3	1	1	3,501	382.8	5.68
E7EMC6	Uncharacterized protein OS=Homo sapiens GN=ANXA6 PE=3 SV=1 - [E7EMC6_HUMAN]	2.12	9	1	1	330	36.9	5.91
E7EQ49	Uncharacterized protein OS=Homo sapiens GN=WDR37 PE=4 SV=1 - [E7EQ49_HUMAN]	4.82	3	1	1	249	27.4	8.95
Q5JTE7	Exportin 5 (Fragment) OS=Homo sapiens GN=XPO5 PE=2 SV=1 - [Q5JTE7_HUMAN]	1.20	5	1	1	666	75.2	5.12
A1A4V4	DRD3 protein OS=Homo sapiens GN=DRD3 PE=2 SV=1 - [A1A4V4_HUMAN]	1.50	1	1	1	400	44.2	8.78
A5D8X4	BTB (POZ) domain containing 7 OS=Homo sapiens GN=BTBD7 PE=2 SV=1 - [A5D8X4_HUMAN]	1.95	2	1	1	410	46.0	5.85
C9J8W0	Uncharacterized protein OS=Homo sapiens GN=LRP3 PE=4 SV=1 - [C9J8W0_HUMAN]	4.21	1	1	1	285	30.6	5.38
E9PHQ9	Uncharacterized protein OS=Homo sapiens GN=COL20A1 PE=4 SV=1 - [E9PHQ9_HUMAN]	0.70	2	1	1	1,279	135.4	7.72
E7EWX9	Uncharacterized protein OS=Homo sapiens GN=TMF1 PE=4 SV=1 - [E7EWX9_HUMAN]	1.13	5	1	1	533	58.4	4.77
B2RCG8	cDNA, FLJ96063, highly similar to Homo sapiens rhophilin, Rho GTPase binding protein 2 (RHPN2), mRNA OS=Homo sapiens PE=2 SV=1 - [B2RCG8_HUMAN]	2.04	3	1	1	686	76.9	6.92
A5H1S6	MLL/MAML2 fusion protein (Fragment) OS=Homo sapiens GN=MLL/MAML2 fusion PE=2 SV=1 - [A5H1S6_HUMAN]	12.93	1	1	1	147	15.8	8.50
E7ENY8	Uncharacterized protein OS=Homo sapiens GN=COL3A1 PE=4 SV=1 - [E7ENY8_HUMAN]	2.06	2	1	1	1,163	111.9	5.88
B7Z7U1	cDNA FLJ52805, moderately similar to Dynein intermediate chain 1, axonemal OS=Homo sapiens PE=2 SV=1 - [B7Z7U1_HUMAN]	10.12	1	1	1	168	19.3	9.26
B7Z7R0	cDNA FLJ50732, highly similar to Guanine nucleotide-binding protein alpha-13 subunit OS=Homo sapiens PE=2 SV=1 - [B7Z7R0_HUMAN]	4.26	4	1	1	282	33.2	6.46
B4DGF2	cDNA FLJ56069 OS=Homo sapiens PE=2 SV=1 - [B4DGF2_HUMAN]	4.91	2	1	1	795	89.7	5.96
Q2HJC8	HDLBP protein (Fragment) OS=Homo sapiens GN=HDLBP PE=2 SV=1 - [Q2HJC8_HUMAN]	1.36	9	1	1	589	65.3	5.67
B3KWK2	cDNA FLJ43211 fis, clone FEBRA2020668 OS=Homo sapiens PE=2 SV=1 - [B3KWK2_HUMAN]	8.86	2	1	1	350	40.1	9.09
Q6P0M3	TTC7A protein (Fragment) OS=Homo sapiens GN=TTC7A PE=2 SV=1 - [Q6P0M3_HUMAN]	1.60	4	1	1	686	76.9	6.27
B7Z369	cDNA FLJ54683, highly similar to Homo sapiens sushi domain containing 4 (SUSD4), transcript variant 1, mRNA OS=Homo sapiens PE=2 SV=1 - [B7Z369_HUMAN]	6.89	1	1	1	421	46.2	4.81
B4DL80	cDNA FLJ57525, highly similar to Cell division cycle protein 27 homolog OS=Homo sapiens PE=2 SV=1 - [B4DL80_HUMAN]	0.92	2	1	1	763	84.8	7.31
E9PMN9	Uncharacterized protein OS=Homo sapiens GN=NAT10 PE=4 SV=1 - [E9PMN9_HUMAN]	4.21	4	1	1	617	69.9	7.65
Q5CZB7	Putative uncharacterized protein DKFZp686L21237 OS=Homo sapiens GN=DKFZp686L21237 PE=2 SV=1 - [Q5CZB7_HUMAN]	6.91	1	1	1	246	28.6	5.82
D6RAK9	Uncharacterized protein OS=Homo sapiens GN=VPRBP PE=4 SV=1 - [D6RAK9_HUMAN]	6.65	2	1	1	376	42.7	4.91
B2RAJ0	cDNA, FLJ94946, highly similar to Homo sapiens cartilage intermediate layer protein 2 (CILP2), mRNA OS=Homo sapiens PE=2 SV=1 - [B2RAJ0_HUMAN]	2.42	2	1	1	1,156	126.2	8.24
A8K9G7	cDNA FLJ75094, highly similar to Homo sapiens neural cell expressed, developmentally down-regulated 9 (NEDD9), transcript variant 1, mRNA OS=Homo sapiens PE=2 SV=1 - [A8K9G7_HUMAN]	0.84	2	1	1	834	92.9	6.70
B3KYB0	cDNA FLJ42691 fis, clone BRAMY3002803, highly similar to Serine/threonine-protein kinase PAK 6 (EC 2.7.11.1) OS=Homo sapiens PE=2 SV=1 - [B3KYB0_HUMAN]	1.57	2	1	1	636	69.6	9.61
B3KQ25	cDNA FLJ32659 fis, clone TESTI1000045, highly similar to Proteasome activator complex subunit 3 OS=Homo sapiens PE=2 SV=1 - [B3KQ25_HUMAN]	4.66	3	1	1	193	22.4	6.80
B2R5R0	cDNA, FLJ92575, Homo sapiens glycoprotein 2 (zymogen granule membrane) (GP2), mRNA OS=Homo sapiens PE=2 SV=1 - [B2R5R0_HUMAN]	1.32	3	1	1	530	58.6	5.12
A8K313	cDNA FLJ78249, highly similar to Homo sapiens RAD51 associated protein 1, mRNA OS=Homo sapiens PE=2 SV=1 - [A8K313_HUMAN]	4.26	2	1	1	352	38.4	9.11
B3KP78	cDNA FLJ31312 fis, clone LIVER1000224, highly similar to Solute carrier organic anion transporter family member 1B3 OS=Homo sapiens PE=2 SV=1 - [B3KP78_HUMAN]	1.42	4	1	1	702	77.2	8.82
Q58F05	NARG1 protein (Fragment) OS=Homo sapiens GN=NARG1 PE=2 SV=1 - [Q58F05_HUMAN]	0.98	4	1	1	614	72.3	8.37
Q9H6X7	cDNA: FLJ21736 fis, clone COLF3353 OS=Homo sapiens PE=2 SV=1 - [Q9H6X7_HUMAN]	15.71	2	1	1	210	24.1	5.27
C9J978	Uncharacterized protein OS=Homo sapiens GN=GPR98 PE=4 SV=1 - [C9J978_HUMAN]	1.02	4	1	1	1,466	162.0	4.67
E7ENZ2	Uncharacterized protein OS=Homo sapiens GN=FCHSD2 PE=4 SV=1 - [E7ENZ2_HUMAN]	3.64	3	1	1	604	67.9	5.21
E7EVK8	Uncharacterized protein OS=Homo sapiens GN=ZNF45 PE=4 SV=1 - [E7EVK8_HUMAN]	1.68	3	1	1	537	61.4	8.95
B7Z2D1	cDNA FLJ59197, moderately similar to Bestrophin-1 OS=Homo sapiens PE=2 SV=1 - [B7Z2D1_HUMAN]	13.67	4	1	1	139	15.3	9.63
Q59FF8	CUB and Sushi multiple domains 1 variant (Fragment) OS=Homo sapiens PE=2 SV=1 - [Q59FF8_HUMAN]	0.67	3	1	1	2,966	323.3	6.04
Q580I4	Putative uncharacterized protein CACNB4 (Fragment) OS=Homo sapiens GN=CACNB4 PE=1 SV=1 - [Q580I4_HUMAN]	7.87	6	1	1	267	30.1	9.14
B4DH23	cDNA FLJ57925, highly similar to Alpha-mannosidase 2C1 (EC 3.2.1.24) OS=Homo sapiens PE=2 SV=1 - [B4DH23_HUMAN]	10.05	5	1	1	209	23.4	5.90
B4DM60	cDNA FLJ51499, highly similar to BR serine/threonine-protein kinase 2 (EC 2.7.11.1) OS=Homo sapiens PE=2 SV=1 - [B4DM60_HUMAN]	4.61	5	1	1	369	40.6	9.66
B4DNP2	cDNA FLJ60739, highly similar to Homo sapiens cancer susceptibility candidate 1 (CASC1), mRNA OS=Homo sapiens PE=2 SV=1 - [B4DNP2_HUMAN]	3.81	3	1	1	657	75.8	5.11
C9JMM2	Uncharacterized protein OS=Homo sapiens GN=PIP4K2B PE=4 SV=1 - [C9JMM2_HUMAN]	5.05	2	1	1	277	31.8	9.20
C9J1I5	Uncharacterized protein OS=Homo sapiens GN=XKR5 PE=4 SV=1 - [C9J1I5_HUMAN]	2.04	2	1	1	685	74.9	6.68
E2QRI9	Uncharacterized protein OS=Homo sapiens GN=AP1S3 PE=4 SV=1 - [E2QRI9_HUMAN]	15.87	4	1	1	63	7.8	10.55
E2QRL0	Uncharacterized protein OS=Homo sapiens GN=KIF26B PE=4 SV=1 - [E2QRL0_HUMAN]	1.70	3	1	1	1,350	143.7	7.99
B9EIJ9	FLJ46481 protein OS=Homo sapiens GN=FLJ46481 PE=2 SV=1 - [B9EIJ9_HUMAN]	3.26	3	1	1	276	30.6	5.73
C9JEA8	Uncharacterized protein OS=Homo sapiens PE=3 SV=1 - [C9JEA8_HUMAN]	1.43	1	1	1	419	46.6	5.66
Q9BS61	KMO protein OS=Homo sapiens PE=2 SV=1 - [Q9BS61_HUMAN]	4.42	1	1	1	407	46.5	8.46
C9J5M6	Uncharacterized protein OS=Homo sapiens GN=SDK2 PE=4 SV=1 - [C9J5M6_HUMAN]	3.51	4	1	1	513	56.2	7.91
C9K018	Uncharacterized protein OS=Homo sapiens PE=4 SV=1 - [C9K018_HUMAN]	13.76	1	1	1	109	11.9	4.03
Q59F56	DJ34F7.7 (Superkiller viralicidic activity 2-like variant) (Fragment) OS=Homo sapiens PE=2 SV=1 - [Q59F56_HUMAN]	4.12	9	1	1	607	67.5	8.75
B4DYH5	cDNA FLJ50094, highly similar to Beta klotho OS=Homo sapiens PE=2 SV=1 - [B4DYH5_HUMAN]	8.88	4	1	1	259	30.3	10.02
B4DHE1	cDNA FLJ61257, highly similar to Ribosomal protein S6 kinase delta-1 (EC 2.7.11.1) OS=Homo sapiens PE=2 SV=1 - [B4DHE1_HUMAN]	1.06	3	1	1	660	72.8	4.84
C9K0I0	Uncharacterized protein OS=Homo sapiens GN=TSKS PE=4 SV=1 - [C9K0I0_HUMAN]	9.95	2	1	1	392	43.1	6.18
C9J9V0	Uncharacterized protein OS=Homo sapiens GN=PRR14L PE=4 SV=1 - [C9J9V0_HUMAN]	15.00	2	1	1	180	19.3	4.68
B9EGR0	C4orf8 protein OS=Homo sapiens GN=C4orf8 PE=2 SV=1 - [B9EGR0_HUMAN]	1.16	4	1	1	1,211	134.2	6.40
C9JI92	Uncharacterized protein OS=Homo sapiens GN=ALS2CR8 PE=4 SV=1 - [C9JI92_HUMAN]	2.57	5	1	1	623	69.4	6.38
D6RBC1	Uncharacterized protein OS=Homo sapiens GN=POC5 PE=4 SV=1 - [D6RBC1_HUMAN]	4.94	3	1	1	162	19.0	7.30
Q6NUN0	Acyl-coenzyme A synthetase ACSM5, mitochondrial OS=Homo sapiens GN=ACSM5 PE=2 SV=2 - [ACSM5_HUMAN]	5.01	1	1	1	579	64.7	8.40
P51828	Adenylate cyclase type 7 OS=Homo sapiens GN=ADCY7 PE=2 SV=1 - [ADCY7_HUMAN]	1.57	1	1	1	1,080	120.2	8.12
Q76KP1	N-acetyl-beta-glucosaminyl-glycoprotein 4-beta-N-acetylgalactosaminyltransferase 1 OS=Homo sapiens GN=B4GALNT4 PE=1 SV=1 - [B4GN4_HUMAN]	0.77	1	1	1	1,039	116.4	6.98
Q8NEQ6	Uncharacterized protein C1orf64 OS=Homo sapiens GN=C1orf64 PE=2 SV=1 - [CA064_HUMAN]	13.02	1	1	1	169	17.6	8.95
Q68DN1	Uncharacterized protein C2orf16 OS=Homo sapiens GN=C2orf16 PE=1 SV=3 - [CB016_HUMAN]	0.91	1	1	1	1,984	224.2	10.08
Q9NRJ2	Putative uncharacterized protein C9orf31 OS=Homo sapiens GN=C9orf31 PE=2 SV=1 - [CI031_HUMAN]	12.27	1	1	1	163	17.6	10.93
P22680	Cholesterol 7-alpha-monooxygenase OS=Homo sapiens GN=CYP7A1 PE=1 SV=2 - [CP7A1_HUMAN]	3.77	1	1	1	504	57.6	8.24
Q7L014	Probable ATP-dependent RNA helicase DDX46 OS=Homo sapiens GN=DDX46 PE=1 SV=2 - [DDX46_HUMAN]	1.84	1	1	1	1,031	117.3	9.29
Q86SG4	Dresden prostate carcinoma protein 2 OS=Homo sapiens GN=HMGN2P46 PE=2 SV=1 - [DPCA2_HUMAN]	4.07	1	1	1	172	20.4	9.83
Q96JB1	Dynein heavy chain 8, axonemal OS=Homo sapiens GN=DNAH8 PE=1 SV=2 - [DYH8_HUMAN]	0.42	1	1	1	4,490	514.3	6.32
Q13347	Eukaryotic translation initiation factor 3 subunit I OS=Homo sapiens GN=EIF3I PE=1 SV=1 - [EIF3I_HUMAN]	3.08	2	1	1	325	36.5	5.64
Q9HCE0	Ectopic P granules protein 5 homolog OS=Homo sapiens GN=EPG5 PE=2 SV=2 - [EPG5_HUMAN]	0.66	1	1	1	2,579	292.3	6.43
Q14677	Clathrin interactor 1 OS=Homo sapiens GN=CLINT1 PE=1 SV=1 - [EPN4_HUMAN]	3.20	2	1	1	625	68.2	6.42
Q9NV70	Exocyst complex component 1 OS=Homo sapiens GN=EXOC1 PE=1 SV=4 - [EXOC1_HUMAN]	1.01	1	1	1	894	101.9	6.61
Q8NB25	Protein FAM184A OS=Homo sapiens GN=FAM184A PE=2 SV=3 - [F184A_HUMAN]	2.11	1	1	1	1,140	132.9	5.83
Q5SY85	Protein FAM201A OS=Homo sapiens GN=FAM201A PE=2 SV=1 - [F201A_HUMAN]	11.61	1	1	1	155	16.5	8.68
Q8NA70	Protein FAM47B OS=Homo sapiens GN=FAM47B PE=2 SV=2 - [FA47B_HUMAN]	3.57	2	1	1	645	73.9	8.73
Q76B58	Protein FAM5C OS=Homo sapiens GN=FAM5C PE=2 SV=1 - [FAM5C_HUMAN]	1.96	1	1	1	766	88.4	7.81
Q5H8C1	FRAS1-related extracellular matrix protein 1 OS=Homo sapiens GN=FREM1 PE=1 SV=3 - [FREM1_HUMAN]	1.33	3	1	1	2,179	244.0	5.90
Q9NXP7	Gypsy retrotransposon integrase-like protein 1 OS=Homo sapiens GN=GIN1 PE=2 SV=3 - [GIN1_HUMAN]	3.26	1	1	1	522	59.8	7.97
Q16478	Glutamate receptor, ionotropic kainate 5 OS=Homo sapiens GN=GRIK5 PE=2 SV=2 - [GRIK5_HUMAN]	3.57	1	1	1	980	109.2	8.21
Q12849	G-rich sequence factor 1 OS=Homo sapiens GN=GRSF1 PE=1 SV=3 - [GRSF1_HUMAN]	4.58	1	1	1	480	53.1	6.19
Q9Y581	Insulin-like peptide INSL6 OS=Homo sapiens GN=INSL6 PE=2 SV=2 - [INSL6_HUMAN]	6.57	1	1	1	213	24.8	9.67
Q92831	Histone acetyltransferase KAT2B OS=Homo sapiens GN=KAT2B PE=1 SV=3 - [KAT2B_HUMAN]	0.96	1	1	1	832	93.0	9.01
Q2M1P5	Kinesin-like protein KIF7 OS=Homo sapiens GN=KIF7 PE=1 SV=2 - [KIF7_HUMAN]	1.56	1	1	1	1,343	150.5	6.79
Q5T749	Keratinocyte proline-rich protein OS=Homo sapiens GN=KPRP PE=1 SV=1 - [KPRP_HUMAN]	1.04	1	1	1	579	64.1	8.27
P25391	Laminin subunit alpha-1 OS=Homo sapiens GN=LAMA1 PE=1 SV=2 - [LAMA1_HUMAN]	0.68	1	1	1	3,075	336.9	6.35
Q8TF66	Leucine-rich repeat-containing protein 15 OS=Homo sapiens GN=LRRC15 PE=1 SV=1 - [LRC15_HUMAN]	4.13	1	1	1	581	64.4	6.71
P30301	Lens fiber major intrinsic protein OS=Homo sapiens GN=MIP PE=1 SV=1 - [MIP_HUMAN]	10.65	1	1	1	263	28.1	8.48
Q86WG5	Myotubularin-related protein 13 OS=Homo sapiens GN=SBF2 PE=1 SV=1 - [MTMRD_HUMAN]	1.03	1	1	1	1,849	208.3	7.06
Q5H9M0	PWWP domain-containing protein MUM1L1 OS=Homo sapiens GN=MUM1L1 PE=2 SV=1 - [MUML1_HUMAN]	3.45	1	1	1	696	79.0	4.97
Q8NFZ4	Neuroligin-2 OS=Homo sapiens GN=NLGN2 PE=1 SV=1 - [NLGN2_HUMAN]	0.72	1	1	1	835	90.8	6.18
P35228	Nitric oxide synthase, inducible OS=Homo sapiens GN=NOS2 PE=1 SV=2 - [NOS2_HUMAN]	1.73	1	1	1	1,153	131.0	7.96
Q9P121	Neurotrimin OS=Homo sapiens GN=NTM PE=1 SV=1 - [NTRI_HUMAN]	5.23	5	1	1	344	37.9	7.81
Q8NGF3	Olfactory receptor 51D1 OS=Homo sapiens GN=OR51D1 PE=2 SV=1 - [O51D1_HUMAN]	3.09	1	1	1	324	35.8	8.66
Q8IV76	PAS domain-containing protein 1 OS=Homo sapiens GN=PASD1 PE=2 SV=1 - [PASD1_HUMAN]	0.78	1	1	1	773	87.4	5.07
Q8NA58	Poly(A)-specific ribonuclease PARN-like domain-containing protein 1 OS=Homo sapiens GN=PNLDC1 PE=2 SV=2 - [PNDC1_HUMAN]	1.54	1	1	1	520	60.1	8.57
Q99496	E3 ubiquitin-protein ligase RING2 OS=Homo sapiens GN=RNF2 PE=1 SV=1 - [RING2_HUMAN]	4.76	1	1	1	336	37.6	6.84
P50443	Sulfate transporter OS=Homo sapiens GN=SLC26A2 PE=1 SV=2 - [S26A2_HUMAN]	2.71	1	1	1	739	81.6	8.38
Q9Y2H2	Phosphatidylinositide phosphatase SAC2 OS=Homo sapiens GN=INPP5F PE=1 SV=3 - [SAC2_HUMAN]	0.71	1	1	1	1,132	128.3	7.02
P37088	Amiloride-sensitive sodium channel subunit alpha OS=Homo sapiens GN=SCNN1A PE=1 SV=1 - [SCNNA_HUMAN]	1.35	5	1	1	669	75.7	7.52
O14796	SH2 domain-containing protein 1B OS=Homo sapiens GN=SH2D1B PE=1 SV=2 - [SH21B_HUMAN]	12.12	1	1	1	132	15.3	8.82
Q96PQ1	Sialic acid-binding Ig-like lectin 12 OS=Homo sapiens GN=SIGLEC12 PE=1 SV=1 - [SIG12_HUMAN]	1.34	1	1	1	595	64.9	6.68
Q7Z6B7	SLIT-ROBO Rho GTPase-activating protein 1 OS=Homo sapiens GN=SRGAP1 PE=1 SV=1 - [SRGP1_HUMAN]	0.83	1	1	1	1,085	124.2	6.83
O43294	Transforming growth factor beta-1-induced transcript 1 protein OS=Homo sapiens GN=TGFB1I1 PE=1 SV=2 - [TGFI1_HUMAN]	3.47	1	1	1	461	49.8	7.03
Q9Y4G6	Talin-2 OS=Homo sapiens GN=TLN2 PE=1 SV=4 - [TLN2_HUMAN]	0.63	1	1	1	2,542	271.4	5.57
Q03169	Tumor necrosis factor alpha-induced protein 2 OS=Homo sapiens GN=TNFAIP2 PE=1 SV=2 - [TNAP2_HUMAN]	1.38	1	1	1	654	72.6	6.46
Q96QT4	Transient receptor potential cation channel subfamily M member 7 OS=Homo sapiens GN=TRPM7 PE=1 SV=1 - [TRPM7_HUMAN]	0.32	1	1	1	1,865	212.6	7.88
Q9BZV1	UBX domain-containing protein 6 OS=Homo sapiens GN=UBXN6 PE=1 SV=1 - [UBXN6_HUMAN]	4.08	1	1	1	441	49.7	6.89
Q5VZL5	Zinc finger MYM-type protein 4 OS=Homo sapiens GN=ZMYM4 PE=1 SV=1 - [ZMYM4_HUMAN]	0.52	1	1	1	1,548	172.7	6.84
Q6ZQV5	Zinc finger protein 788 OS=Homo sapiens GN=ZNF788 PE=2 SV=2 - [ZN788_HUMAN]	1.46	1	1	1	615	71.9	9.55
D7SFF4	ATP synthase protein 8 OS=Homo sapiens GN=ATP8 PE=3 SV=1 - [D7SFF4_HUMAN]	16.18	1	1	1	68	8.0	9.91
Q5TEH8	Protein Wnt OS=Homo sapiens GN=WNT2B PE=2 SV=1 - [Q5TEH8_HUMAN]	2.68	4	1	1	299	33.9	8.90
A6PVK7	Chromosome 9 open reading frame 84 OS=Homo sapiens GN=C9orf84 PE=4 SV=1 - [A6PVK7_HUMAN]	1.02	3	1	1	1,370	157.0	5.50
D6W646	HCG22253, isoform CRA_a OS=Homo sapiens GN=hCG_22253 PE=4 SV=1 - [D6W646_HUMAN]	1.08	2	1	1	1,015	114.0	6.30
Q96DJ8	CDNA FLJ25312 fis, clone SYN01070 OS=Homo sapiens GN=hCG_2039992 PE=2 SV=1 - [Q96DJ8_HUMAN]	21.05	1	1	1	133	14.4	7.78
B3KSQ7	Drebrin 1, isoform CRA_d OS=Homo sapiens GN=DBN1 PE=2 SV=1 - [B3KSQ7_HUMAN]	2.67	1	1	1	599	65.6	4.53
B7ZBE1	Sfi1 homolog, spindle assembly associated (Yeast) (Fragment) OS=Homo sapiens GN=SFI1 PE=4 SV=1 - [B7ZBE1_HUMAN]	1.58	3	1	1	825	96.2	11.03
Q13161	Bone morphogenetic protein receptor, type II (Serine/threonine kinase), isoform CRA_a OS=Homo sapiens GN=BMPR2 PE=2 SV=1 - [Q13161_HUMAN]	4.53	3	1	1	530	59.9	5.66
B1AK94	FGGY carbohydrate kinase domain containing OS=Homo sapiens GN=FGGY PE=2 SV=1 - [B1AK94_HUMAN]	3.46	3	1	1	463	50.0	6.09
B6EBE3	HCG1774694 OS=Homo sapiens GN=HTA PE=4 SV=1 - [B6EBE3_HUMAN]	10.87	1	1	1	92	10.2	10.89
Q5TAD4	Mesoderm induction early response 1 homolog (Xenopus laevis) OS=Homo sapiens GN=MIER1 PE=2 SV=1 - [Q5TAD4_HUMAN]	5.76	2	1	1	486	54.5	4.41
B2RTQ9	FANCM protein OS=Homo sapiens GN=FANCM PE=2 SV=1 - [B2RTQ9_HUMAN]	0.74	2	1	1	2,022	229.1	6.04
A2RUE7	Glutamate receptor, ionotropic, N-methyl-D-aspartate 3A OS=Homo sapiens GN=GRIN3A PE=2 SV=1 - [A2RUE7_HUMAN]	0.99	2	1	1	1,115	125.5	7.77
B3KY49	Family with sequence similarity 11, member A, isoform CRA_a OS=Homo sapiens GN=FAM11A PE=2 SV=1 - [B3KY49_HUMAN]	11.63	1	1	1	172	20.0	6.79
B4DZH1	HCG1999003 OS=Homo sapiens GN=hCG_1999003 PE=2 SV=1 - [B4DZH1_HUMAN]	3.69	7	1	1	217	24.4	9.76
Q05BU7	WASF2 protein (Fragment) OS=Homo sapiens GN=WASF2 PE=2 SV=1 - [Q05BU7_HUMAN]	5.98	3	1	1	184	21.4	9.25
A8MVQ6	Uncharacterized protein OS=Homo sapiens GN=MPHOSPH9 PE=4 SV=1 - [A8MVQ6_HUMAN]	3.32	2	1	1	692	78.1	6.71
Q6ZQZ4	cDNA FLJ46776 fis, clone TRACH3026650, highly similar to Actin cross-linking family protein 7 OS=Homo sapiens PE=2 SV=1 - [Q6ZQZ4_HUMAN]	0.95	3	1	1	1,372	153.6	8.27
B2R4N3	cDNA, FLJ92155, highly similar to Homo sapiens ubiquitin-like 5 (UBL5), mRNA OS=Homo sapiens PE=4 SV=1 - [B2R4N3_HUMAN]	8.22	2	1	1	73	8.5	7.28
B4E045	cDNA FLJ59131, highly similar to Integrin alpha-4 OS=Homo sapiens PE=2 SV=1 - [B4E045_HUMAN]	14.37	7	1	1	174	18.8	5.52
B4DMZ0	cDNA FLJ55227, highly similar to Tyrosine-protein kinase CSK (EC 2.7.10.2) OS=Homo sapiens PE=2 SV=1 - [B4DMZ0_HUMAN]	4.58	6	1	1	371	42.1	6.39
Q59F39	IL2-inducible T-cell kinase variant (Fragment) OS=Homo sapiens PE=2 SV=1 - [Q59F39_HUMAN]	1.86	2	1	1	376	43.8	8.94
Q4VX86	Chromosome 20 open reading frame 166 OS=Homo sapiens GN=C20orf166 PE=2 SV=1 - [Q4VX86_HUMAN]	20.20	2	1	1	99	10.9	6.51
E9PG32	Uncharacterized protein OS=Homo sapiens GN=DNAH12 PE=4 SV=1 - [E9PG32_HUMAN]	0.82	2	1	1	2,316	267.3	5.96
E7ENE6	Uncharacterized protein OS=Homo sapiens GN=SPTBN1 PE=4 SV=1 - [E7ENE6_HUMAN]	2.17	3	1	1	690	80.9	6.68
E7EUR7	Uncharacterized protein OS=Homo sapiens GN=KIAA1530 PE=4 SV=1 - [E7EUR7_HUMAN]	9.76	2	1	1	164	18.3	7.09
B1API7	Nuclear receptor coactivator 4 (Fragment) OS=Homo sapiens GN=NCOA4 PE=4 SV=1 - [B1API7_HUMAN]	5.32	8	1	1	263	29.4	6.90
B4DN62	cDNA FLJ53141, highly similar to Homo sapiens phospholipase C, epsilon 1 (PLCE1), mRNA (Fragment) OS=Homo sapiens PE=2 SV=1 - [B4DN62_HUMAN]	2.43	5	1	1	699	77.7	6.54
D6RGH0	Uncharacterized protein OS=Homo sapiens GN=CDK18 PE=4 SV=1 - [D6RGH0_HUMAN]	18.46	1	1	1	65	7.3	9.57
B4DTE0	cDNA FLJ57587, highly similar to Synapsin-3 OS=Homo sapiens PE=2 SV=1 - [B4DTE0_HUMAN]	4.84	4	1	1	186	19.7	10.07
Q9BYY6	Cannabinoid receptor 1 (Fragment) OS=Homo sapiens GN=CNR1 PE=2 SV=1 - [Q9BYY6_HUMAN]	3.03	3	1	1	330	37.4	8.51
O14854	Zinc finger protein (Fragment) OS=Homo sapiens PE=2 SV=1 - [O14854_HUMAN]	38.46	1	1	1	65	7.2	9.91
Q59FR5	Tumor necrosis factor receptor superfamily, member 21 variant (Fragment) OS=Homo sapiens PE=2 SV=1 - [Q59FR5_HUMAN]	4.64	3	1	1	345	38.9	8.16
B7ZB92	Adenylosuccinate lyase (Fragment) OS=Homo sapiens GN=ADSL PE=4 SV=1 - [B7ZB92_HUMAN]	6.93	6	1	1	101	11.3	5.83
Q6JHX1	Sarco/endoplasmic reticulum Ca2+ ATPase isoform 3f (Fragment) OS=Homo sapiens GN=ATP2A3 PE=2 SV=1 - [Q6JHX1_HUMAN]	7.04	3	1	1	142	15.8	9.19
B7Z6W6	cDNA FLJ52268, highly similar to Intercellular adhesion molecule 3 OS=Homo sapiens PE=2 SV=1 - [B7Z6W6_HUMAN]	11.92	3	1	1	151	16.8	7.06
B1ARF3	Inositol polyphosphate-5-phosphatase, 75kDa OS=Homo sapiens GN=INPP5B PE=4 SV=1 - [B1ARF3_HUMAN]	5.64	2	1	1	319	34.6	5.47
B4DS20	cDNA FLJ60341, highly similar to MAGUK p55 subfamily member 3 OS=Homo sapiens PE=2 SV=1 - [B4DS20_HUMAN]	3.35	2	1	1	388	43.6	6.65
Q7Z6I4	SCAF1 protein OS=Homo sapiens GN=SCAF1 PE=2 SV=1 - [Q7Z6I4_HUMAN]	8.68	2	1	1	219	23.8	5.11
A8K5R2	cDNA FLJ77293, highly similar to Homo sapiens vasohibin 1 (VASH1), mRNA OS=Homo sapiens PE=2 SV=1 - [A8K5R2_HUMAN]	7.12	2	1	1	365	40.9	9.54
B4DSN3	cDNA FLJ60345, highly similar to Protein transport protein Sec24C OS=Homo sapiens PE=2 SV=1 - [B4DSN3_HUMAN]	1.95	2	1	1	975	106.5	6.96
C9JVR1	Uncharacterized protein OS=Homo sapiens GN=TENC1 PE=4 SV=1 - [C9JVR1_HUMAN]	0.57	3	1	1	1,399	151.4	8.41
E9PM16	Uncharacterized protein OS=Homo sapiens GN=ZNF7 PE=4 SV=1 - [E9PM16_HUMAN]	5.22	1	1	1	134	14.4	5.22
Q59G44	Calcium-transporting ATPase 2C1 isoform 1d variant (Fragment) OS=Homo sapiens PE=2 SV=1 - [Q59G44_HUMAN]	2.87	7	1	1	627	68.6	8.09
B2R6Y2	cDNA, FLJ93173, Homo sapiens sulfite oxidase (SUOX), nuclear gene encodingmitochondrial protein, mRNA OS=Homo sapiens PE=2 SV=1 - [B2R6Y2_HUMAN]	1.84	2	1	1	488	53.9	5.58
B9EIL8	Olfactory receptor, family 52, subfamily N, member 2 OS=Homo sapiens GN=OR52N2 PE=2 SV=1 - [B9EIL8_HUMAN]	4.98	2	1	1	321	35.9	8.15
B5MC86	Uncharacterized protein OS=Homo sapiens GN=EHBP1 PE=4 SV=1 - [B5MC86_HUMAN]	2.90	3	1	1	310	35.5	5.20
C9J8T4	Uncharacterized protein OS=Homo sapiens GN=RNF13 PE=4 SV=1 - [C9J8T4_HUMAN]	14.72	4	1	1	163	17.9	4.77
Q7KYM9	ORF protein OS=Homo sapiens GN=ORF PE=2 SV=1 - [Q7KYM9_HUMAN]	2.98	2	1	1	570	59.9	8.41
Q8TEC9	cDNA FLJ23639 fis, clone CAS11535 OS=Homo sapiens PE=2 SV=1 - [Q8TEC9_HUMAN]	2.55	1	1	1	353	39.8	8.16
B7ZLM9	CXorf23 protein (Fragment) OS=Homo sapiens GN=CXorf23 PE=2 SV=1 - [B7ZLM9_HUMAN]	5.14	2	1	1	311	35.8	6.70
O95283	Envelope protein RIC-5 OS=Homo sapiens GN=env PE=4 SV=1 - [O95283_HUMAN]	12.50	2	1	1	152	17.2	6.54
E2QRL3	Uncharacterized protein OS=Homo sapiens GN=C17orf104 PE=4 SV=1 - [E2QRL3_HUMAN]	2.52	2	1	1	754	85.6	8.09
E9PPC4	Uncharacterized protein OS=Homo sapiens GN=RAG1 PE=4 SV=1 - [E9PPC4_HUMAN]	0.86	3	1	1	931	106.1	8.75
B8ZZD5	Uncharacterized protein OS=Homo sapiens PE=4 SV=1 - [B8ZZD5_HUMAN]	21.82	1	1	1	55	6.1	8.88
Q2TSD3	Aging-associated gene 6 protein OS=Homo sapiens PE=2 SV=1 - [Q2TSD3_HUMAN]	2.83	1	1	1	672	76.7	7.05
B3KSJ2	cDNA FLJ36408 fis, clone THYMU2010094, highly similar to Probable phospholipid-transporting ATPase IF (EC 3.6.3.1) OS=Homo sapiens PE=2 SV=1 - [B3KSJ2_HUMAN]	1.22	3	1	1	736	83.4	7.47
B4DJ97	cDNA FLJ53005, highly similar to CAP10-like 46 kDa protein OS=Homo sapiens PE=2 SV=1 - [B4DJ97_HUMAN]	3.43	3	1	1	233	27.5	9.11
B7Z363	cDNA FLJ58775 OS=Homo sapiens PE=2 SV=1 - [B7Z363_HUMAN]	5.53	3	1	1	380	41.8	4.93
B4DKV6	cDNA FLJ60084, highly similar to Pericentrin (Fragment) OS=Homo sapiens PE=2 SV=1 - [B4DKV6_HUMAN]	2.25	2	1	1	888	101.2	5.05
Q8NF61	FLJ00326 protein (Fragment) OS=Homo sapiens GN=FLJ00326 PE=2 SV=1 - [Q8NF61_HUMAN]	7.38	1	1	1	122	13.4	10.49
B4DMJ1	cDNA FLJ52993, highly similar to Heterogeneous nuclear ribonucleoprotein C OS=Homo sapiens PE=2 SV=1 - [B4DMJ1_HUMAN]	17.78	1	1	1	135	14.6	9.69
A6NJC0	Uncharacterized protein OS=Homo sapiens GN=TMCC2 PE=4 SV=2 - [A6NJC0_HUMAN]	2.73	6	1	1	256	28.1	8.88
B4E0X6	Proteasome subunit alpha type OS=Homo sapiens PE=2 SV=1 - [B4E0X6_HUMAN]	17.69	1	1	1	130	14.6	8.51
A8MSQ8	Uncharacterized protein OS=Homo sapiens GN=C2orf55 PE=4 SV=2 - [A8MSQ8_HUMAN]	2.81	2	1	1	605	63.6	5.86
Q53QX9	Putative uncharacterized protein HK2 (Fragment) OS=Homo sapiens GN=HK2 PE=2 SV=1 - [Q53QX9_HUMAN]	3.49	5	1	1	573	63.6	7.06
B4DSL9	cDNA FLJ58748, highly similar to U3 small nucleolar RNA-associated protein 6homolog OS=Homo sapiens PE=2 SV=1 - [B4DSL9_HUMAN]	4.55	4	1	1	330	39.1	6.57
Q96LZ0	cDNA FLJ32975 fis, clone TESTI2013832, moderately similar to Xenopus laevis transketolase mRNA OS=Homo sapiens PE=2 SV=1 - [Q96LZ0_HUMAN]	5.63	3	1	1	320	34.3	6.79
Q5QPL4	Zinc finger protein 643 (Fragment) OS=Homo sapiens GN=ZNF643 PE=2 SV=1 - [Q5QPL4_HUMAN]	13.49	3	1	1	126	14.1	4.82
A8CGI2	Ubiquitin C (Fragment) OS=Homo sapiens GN=UBC PE=2 SV=1 - [A8CGI2_HUMAN]	26.09	13	1	1	23	2.6	4.75
B2RAG4	cDNA, FLJ94902, highly similar to Homo sapiens ring finger protein 103 (RNF103), mRNA OS=Homo sapiens PE=2 SV=1 - [B2RAG4_HUMAN]	2.48	1	1	1	685	79.3	5.68
B3KM27	cDNA FLJ10093 fis, clone HEMBA1002363, highly similar to Structural maintenance of chromosome 2-like 1 protein (Fragment) OS=Homo sapiens PE=2 SV=1 - [B3KM27_HUMAN]	3.89	1	1	1	489	55.9	6.52
Q5T085	Amylase, alpha 1B (Salivary) (Fragment) OS=Homo sapiens GN=AMY1B PE=3 SV=1 - [Q5T085_HUMAN]	14.16	7	1	1	226	25.6	7.31
B7Z954	cDNA FLJ61560, highly similar to Tight junction protein ZO-2 OS=Homo sapiens PE=2 SV=1 - [B7Z954_HUMAN]	1.81	4	1	1	1,108	124.8	8.69
Q8IZ53	C9orf75 protein (Fragment) OS=Homo sapiens GN=C9orf75 PE=2 SV=2 - [Q8IZ53_HUMAN]	6.58	3	1	1	380	41.5	4.82
B3KSE7	cDNA FLJ36081 fis, clone TESTI2019911, highly similar to Disintegrin-like testicular metalloproteinase OS=Homo sapiens PE=2 SV=1 - [B3KSE7_HUMAN]	4.53	1	1	1	331	36.6	7.68
B4DJB5	cDNA FLJ56662, highly similar to Homo sapiens GTP binding protein 6 (GTPBP6), mRNA OS=Homo sapiens PE=2 SV=1 - [B4DJB5_HUMAN]	4.24	3	1	1	354	39.7	9.80
A8K229	cDNA FLJ77897, highly similar to Homo sapiens cripto, FRL-1, cryptic family 1 (CFC1), mRNA OS=Homo sapiens PE=2 SV=1 - [A8K229_HUMAN]	13.90	2	1	1	223	24.6	8.73
D6RC83	Uncharacterized protein OS=Homo sapiens GN=CCDC99 PE=4 SV=1 - [D6RC83_HUMAN]	7.39	3	1	1	230	27.4	5.01
E7EWG2	Uncharacterized protein OS=Homo sapiens GN=TNIP1 PE=4 SV=1 - [E7EWG2_HUMAN]	15.04	12	1	1	133	14.3	4.74
B4DNP0	cDNA FLJ59362, highly similar to Signal transducer and activator of transcription 3 OS=Homo sapiens PE=2 SV=1 - [B4DNP0_HUMAN]	2.08	6	1	1	672	76.1	6.30
B4DJ70	cDNA FLJ58384, highly similar to Natural killer cell receptor 2B4 OS=Homo sapiens PE=2 SV=1 - [B4DJ70_HUMAN]	5.00	3	1	1	140	15.1	8.65
A7E2U9	SLC12A6 protein (Fragment) OS=Homo sapiens GN=SLC12A6 PE=2 SV=1 - [A7E2U9_HUMAN]	13.53	9	1	1	133	15.0	5.34
B4E0A9	cDNA FLJ60381 OS=Homo sapiens PE=2 SV=1 - [B4E0A9_HUMAN]	3.03	1	1	1	330	37.5	6.51
C9JYB8	Uncharacterized protein OS=Homo sapiens GN=CCDC66 PE=4 SV=1 - [C9JYB8_HUMAN]	1.42	2	1	1	633	72.4	7.75
Q6IPM3	ARPC5 protein OS=Homo sapiens GN=ARPC5 PE=4 SV=1 - [Q6IPM3_HUMAN]	27.27	3	1	1	88	9.4	4.63
Q4VBX8	ANKIB1 protein (Fragment) OS=Homo sapiens GN=ANKIB1 PE=2 SV=1 - [Q4VBX8_HUMAN]	1.59	3	1	1	441	48.1	4.68
B4E192	cDNA FLJ54224, highly similar to Glial fibrillary acidic protein, astrocyte OS=Homo sapiens PE=2 SV=1 - [B4E192_HUMAN]	3.69	4	1	1	407	47.3	5.33
B4DE43	cDNA FLJ57948, highly similar to Dual specificity mitogen-activated protein kinase kinase 5 (EC 2.7.12.2) OS=Homo sapiens PE=2 SV=1 - [B4DE43_HUMAN]	3.40	3	1	1	412	46.1	6.25
B7ZLW5	STIL protein OS=Homo sapiens GN=STIL PE=2 SV=1 - [B7ZLW5_HUMAN]	1.18	3	1	1	1,270	141.0	6.47
Q5T3H5	Regulator of G-protein signaling 7 (Fragment) OS=Homo sapiens GN=RGS7 PE=2 SV=1 - [Q5T3H5_HUMAN]	3.07	5	1	1	326	38.1	8.91
B3KP05	cDNA FLJ30874 fis, clone FEBRA2004329, moderately similar to MYOSIN HEAVY CHAIN, CARDIAC MUSCLE ISOFORM OS=Homo sapiens PE=2 SV=1 - [B3KP05_HUMAN]	1.74	2	1	1	975	113.0	5.50
Q0D2I0	PPP1R3F protein (Fragment) OS=Homo sapiens GN=PPP1R3F PE=2 SV=1 - [Q0D2I0_HUMAN]	2.68	7	1	1	336	34.4	4.37
Q86WJ5	Hyperpolarization activated cyclic nucleotide-gated potassium channel OS=Homo sapiens GN=HCN3 PE=2 SV=1 - [Q86WJ5_HUMAN]	0.90	2	1	1	774	86.0	9.76
B3KQE3	cDNA FLJ90319 fis, clone NT2RP2001538, highly similar to Paired amphipathic helix protein Sin3a OS=Homo sapiens PE=2 SV=1 - [B3KQE3_HUMAN]	2.38	2	1	1	378	44.5	6.05
C9JBZ4	Uncharacterized protein OS=Homo sapiens GN=HAUS8 PE=4 SV=1 - [C9JBZ4_HUMAN]	2.44	2	1	1	409	44.8	7.06
B4DTA1	cDNA FLJ60029, highly similar to Keratin, type II cuticular Hb3 OS=Homo sapiens PE=2 SV=1 - [B4DTA1_HUMAN]	5.49	5	1	1	255	29.1	5.63
B7ZW30	AIDA protein (Fragment) OS=Homo sapiens GN=AIDA PE=2 SV=1 - [B7ZW30_HUMAN]	7.00	3	1	1	257	29.1	5.45
Q6ZSQ3	cDNA FLJ45300 fis, clone BRHIP3003688 OS=Homo sapiens PE=2 SV=1 - [Q6ZSQ3_HUMAN]	13.95	1	1	1	129	14.6	8.28
B4DPM1	cDNA FLJ58373, highly similar to Acyl-coenzyme A oxidase 2, peroxisomal (EC 1.17.99.3) OS=Homo sapiens PE=2 SV=1 - [B4DPM1_HUMAN]	4.09	3	1	1	465	52.3	7.18
Q59GD8	Calcium channel, voltage-dependent, L type, alpha 1D subunit variant (Fragment) OS=Homo sapiens PE=2 SV=1 - [Q59GD8_HUMAN]	0.54	3	1	1	1,854	211.3	6.57
C9JEP3	Uncharacterized protein OS=Homo sapiens GN=FAM160B1 PE=4 SV=1 - [C9JEP3_HUMAN]	1.49	1	1	1	738	83.7	5.34
B4DPL4	cDNA FLJ58207 OS=Homo sapiens PE=2 SV=1 - [B4DPL4_HUMAN]	6.88	1	1	1	276	29.0	9.35
B9EIM5	Olfactory receptor, family 13, subfamily F, member 1 OS=Homo sapiens GN=OR13F1 PE=2 SV=1 - [B9EIM5_HUMAN]	2.19	3	1	1	319	35.6	8.63
Q6ZUK0	cDNA FLJ43630 fis, clone SPLEN2030479 OS=Homo sapiens PE=2 SV=1 - [Q6ZUK0_HUMAN]	10.37	1	1	1	164	17.9	9.50
B4DRS0	cDNA FLJ51534, highly similar to Homo sapiens protein phosphatase 1, regulatory (inhibitor) subunit 3C (PPP1R3C), mRNA OS=Homo sapiens PE=2 SV=1 - [B4DRS0_HUMAN]	3.52	5	1	1	199	22.9	6.28
B4DWB2	cDNA FLJ51728, highly similar to G protein-coupled receptor kinase 5 (EC 2.7.11.16) OS=Homo sapiens PE=2 SV=1 - [B4DWB2_HUMAN]	1.65	3	1	1	485	55.6	7.80
Q6MZM7	Putative uncharacterized protein DKFZp686O12165 (Fragment) OS=Homo sapiens GN=DKFZp686O12165 PE=1 SV=1 - [Q6MZM7_HUMAN]	32.19	19	0	46	2,193	240.5	5.30
Q6N084	Putative uncharacterized protein DKFZp686L11144 (Fragment) OS=Homo sapiens GN=DKFZp686L11144 PE=2 SV=1 - [Q6N084_HUMAN]	42.07	21	0	28	1,034	113.3	6.07
B2R6W1	cDNA, FLJ93143, highly similar to Homo sapiens complement component 7 (C7), mRNA OS=Homo sapiens PE=2 SV=1 - [B2R6W1_HUMAN]	41.76	6	0	27	843	93.5	6.48
Q6U2F8	C4A (Fragment) OS=Homo sapiens GN=C4A PE=4 SV=1 - [Q6U2F8_HUMAN]	61.05	18	0	23	534	58.3	6.23
B7Z558	cDNA FLJ51523, highly similar to Inter-alpha-trypsin inhibitor heavy chain H1 OS=Homo sapiens PE=2 SV=1 - [B7Z558_HUMAN]	27.05	1	0	14	769	86.1	6.60
B7Z8B6	cDNA FLJ54395, highly similar to Inter-alpha-trypsin inhibitor heavy chain H1 OS=Homo sapiens PE=2 SV=1 - [B7Z8B6_HUMAN]	27.77	2	0	11	623	69.5	6.16
Q9UL90	Myosin-reactive immunoglobulin heavy chain variable region (Fragment) OS=Homo sapiens PE=2 SV=1 - [Q9UL90_HUMAN]	32.74	26	0	3	113	12.4	8.44
Q5NV62	V3-4 protein (Fragment) OS=Homo sapiens GN=IGLV8-61 PE=4 SV=1 - [Q5NV62_HUMAN]	16.16	1	0	2	99	10.4	4.73
